# Activation of Sirtuin 2 Inhibitors Employing Photoswitchable Geometry and Aqueous Solubility

**DOI:** 10.1002/cmdc.202000148

**Published:** 2020-05-07

**Authors:** Christoph W. Grathwol, Nathalie Wössner, Steven Behnisch‐Cornwell, Lukas Schulig, Lin Zhang, Oliver Einsle, Manfred Jung, Andreas Link

**Affiliations:** ^1^ Institute of Pharmacy University of Greifswald Friedrich-Ludwig-Jahn-Str. 17 17489 Greifswald Germany; ^2^ Institute of Pharmaceutical Sciences University of Freiburg Albertstr. 25 79104 Freiburg, Germany; ^3^ Institute of Biochemistry University of Freiburg Albertstr. 21 79104 Freiburg Germany

**Keywords:** azo dyes, epigenetics, photopharmacology, photoswitches, sirtuins

## Abstract

Because isoenzymes of the experimentally and therapeutically extremely relevant sirtuin family show high similarity, addressing the unique selectivity pocket of sirtuin 2 is a promising strategy towards selective inhibitors. An unrelated approach towards selective inhibition of isoenzymes with varied tissue distribution is targeted drug delivery or spatiotemporal activation by photochemical activation. Azologization of two nicotinamide‐mimicking lead structures was undertaken to combine both approaches and yielded a set of 33 azobenzenes and azopyridines that have been evaluated for their photochemical behaviour and bioactivity. For some compounds, inhibitory activity reached the sub‐micromolar range in their thermodynamically favoured *E* form and could be decreased by photoisomerization to the metastable *Z* form. Besides, derivatization with long‐chain fatty acids yielded potent sirtuin 2 inhibitors, featuring another intriguing aspect of azo‐based photoswitches. In these compounds, switching to the *Z* isomer increased aqueous solubility and thereby enhanced biological activity by up to a factor of 21. The biological activity of two compounds was confirmed by hyperacetylation of sirtuin specific histone proteins in a cell‐based activity assay.

## Graphical abstract

**Switched into action**: Besides fine‐tuning molecular geometry for enzyme inhibition, photoinduced isomerization of azopyridine‐based nicotinamide mimics results in improved aqueous solubility, enforcing the effect on target protein sirtuin 2. The resulting Sirt2 inhibitor could be used to study binding events in cellular systems with spatiotemporal control by two independent molecular and supramolecular effects.

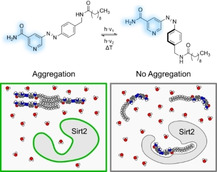

## Introduction

**Figure 1:**
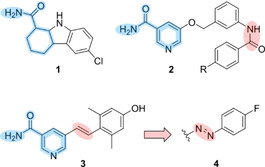
Nicotinamide‐mimicking sirtuin inhibitors: Selisistat (**1**), 5‐[(3‐amidobenzyl)oxy]nicotinamides (**2**) and the structurally related 5‐styrylnicotinamide **3**. Exchange of the amide bond in **2** and the stilbene C,C‐double bond in **3** allows for incorporation of a photoswitchable azo moiety while maintaining the original shape of the parent molecules.

Sirtuins refer to a family of NAD^+^‐dependent lysine deacylases that are highly conserved and found throughout all domains of life. Usually, sirtuins are classified as histone deacetylases (class III HDACs), but also an increasing number of non‐histone proteins were identified as native substrates. In sum, sirtuin‐promoted deacylation is involved in the regulation of various cellular processes such as DNA repair, gene transcription, aging, metabolism, and apoptosis representing an auspicious target for pharmacological intervention.^1^ In human cells, seven sirtuin isotypes (Sirt1–7) have been identified. Sirt1–3 are phylogenetically closely related (class I sirtuins) exhibiting high similarity in their amino acid sequence especially along the catalytic cleft, which comprises the binding sites of the acyl‐lysine substrate and the cofactor NAD^+^.^2^ Nevertheless, these three isotypes show differences in their substrate recognition as well as their enzymatic activity. In addition to a robust deacetylation ability, Sirt2 efficiently binds and cleaves myristoylated peptides, whereas *in vivo* histone decrotonylation has been reported for Sirt3.^3^ During sirtuin‐catalysed protein deacylation, the by‐product nicotinamide functions as an endogenous pan‐sirtuin inhibitor.^4^ Hence, nicotinamide‐mimicking compounds (Figure 1) act as eligible sirtuin inhibitors demonstrated by the potently active Sirt1 inhibitor selisistat (**1**), which was announced to be intended for evaluation in a long term phase III study for the treatment of Huntington's disease.^5^ Furthermore, 5‐[(3‐amidobenzyl)oxy]nicotinamides (**2**) exert robust Sirt2 inhibition evincing promising effects in the therapy of neurodegenerative diseases or cancer.^6^ In our hands, an *in vitro* activity screening of a pooled kinase inhibitor library revealed 5‐styrylnicotinamide **3** as a moderate inhibitor of Sirt2.^7^ Based on this structure, azologization resulted in a reversible photoswitch (**4**) exhibiting comparable inhibitory activity of its stretched out *E* form.^8^, ^9^

Over the past decade, molecular photoswitches have permitted light‐mediated control over a vast scope of biological targets such as ion channels, transporters, GPCRs, and various enzymes.^10^ A common strategy to achieve light‐sensitivity of certain biological target structures is the design of photochromic ligands (PCLs) by insertion of photoswitchable moieties into known bioactive small molecules.^11^ Upon irradiation with appropriate wavelengths, PCLs undergo reversible photoisomerization reactions, accompanied by marked changes in shape and physicochemical properties of the ligand. As the target affinity of a ligand is strongly influenced by its structure and electron distribution, distinct photoisomers often show an altered binding behaviour and consequently exert differential bioactivity.^12^ As a matter of fact, the first PCLs targeting an epigenetic regulator have been photoswitchable sirtuin inhibitors based on a diarylmaleimide photoswitch.^13^ Using an indolyl fulgimide core instead improved the photochemical behaviour of the sirtuin photoswitch under physiological conditions.^14^ Besides, azobenzene‐based photoswitches have been designed for the light‐mediated modulation of human Zn^2+^‐dependent HDACs as well as related bacterial amidohydrolases, offering promising perspectives concerning highly selective antineoplastic and antimicrobial chemotherapy.^15^ Among the known molecular photoswitches, azo dyes hold a prominent role combining a comfortable way of synthesis with finely tuneable photophysical properties. By treatment with UV radiation, (*E*)‐azobenzene segues in a metastable photostationary state (PSS) comprising high amounts of the *Z* isomer followed by slow thermal relaxation of (*Z*)‐azobenzene back to the more stable *E* isomer in the dark. Blue light radiation accelerates *Z*→*E* isomerization, while complete transformation is only obtained thermally.^16^ Wavelength of maximal absorption, half‐life of the metastable *Z* isomer, and photoisomer distribution (PSD) are strongly influenced by different substitutions at the azobenzene core or the incorporation of heteroaromatic moieties affecting also other physicochemical properties, ultimately determining absorption and distribution.^17^

Building on our previous efforts in the design of photoswitchable sirtuin inhibitors, we now present the synthesis of various azopyridine‐based photoswitches in analogy to compound **4**.^8^ Furthermore, another azologization approach yielded azobenzene‐based photoswitches derived from recently published 5‐benzyloxynicotinamides (**2**).^6^ The photophysical and photochemical properties of the obtained azo dyes were studied and adjusted to long thermal half‐lives of the metastable *Z* isomers (>300 h) under physiological conditions. The biological activity of the compounds was determined *in vitro* applying a fluorescence‐based enzyme assay and could also be proven in a urinary cancer cell line.

## Results and Discussion

### Synthesis

**Scheme 1:**
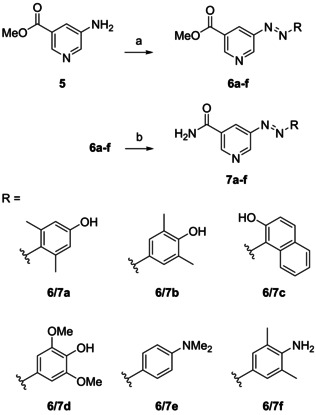
a) 1. NaNO_2_, HCl, H_2_O, 0 °C; 2. phenols or anilines, NaOH, H_2_O, 0 °C to RT, 31–65 %; b) NH_3_, MeOH, RT, 1–4 d, quant.

Starting from commercially available methyl 5‐aminonicotinate (**5**), a first set of heteroaryl azo dyes was easily accessible in two steps. Diazotization and azo coupling of **5** with phenols and anilines gave the respective methyl 5‐diazenylnicotinates (**6 a**–**f**) in moderate yields. Quantitative transformation to nicotinamides was accomplished by successive ammonolysis, providing phenols **7 a**–**d** and anilines **7 e** and **7 f** in satisfying overall yield (Scheme 1). Although, ammonolysis of **5** prior to derivatization by azo coupling might be more beneficial in the synthesis of a compound library, we generally performed ammonolysis at the final step because of more efficient chromatographic purification of methyl nicotinates in comparison to 5‐diazenylnicotinamides.

**Scheme 2:**
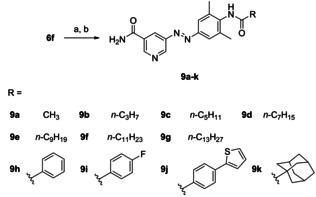
a) acyl chloride, pyridine or DIPEA, THF, 0 °C to RT, 1–3 d, 26–58 %; b) NH_3_, MeOH, RT, 1–4 d, quant.

Aniline **6 f** was converted to several amides (**8 a**–**k**, structures not shown) by reaction with acyl chlorides resulting in compounds **9 a**–**k** after final ammonolysis (Scheme 2). As nucleophilic attack of the amino group in **6 f** is hampered by the two neighbouring methyl groups, amide formation required long reaction times (1–3 days) and gave only modest yields.

**Scheme 3:**
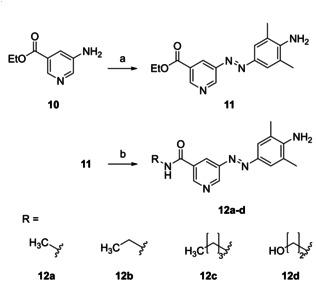
a) 1. NaNO_2_, HCl, H_2_O, 0 °C; 2. 2,6‐dimethylaniline, NaOH, H_2_O, 0 °C to RT, 42 %; b) R‐NH_2_, MeOH, 40–110 °C, 4 d, 88–98 %.

Derivatization to *N*‐alkyl nicotinamides (**12 a**–**d**) was achievable by aminolysis of ethyl nicotinate **11** using the respective primary alkylamines (Scheme 3). On the contrary, *N*‐alkyl nicotinamides could not be obtained through aminolysis of the corresponding methyl nicotinate **6 f** due to an insufficient reactivity of the methyl ester with primary alkylamines.

**Scheme 4:**
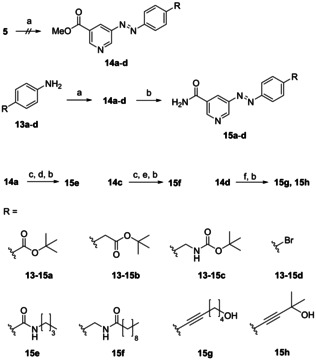
a) 1. oxone, CH_2_Cl_2_, H_2_O, RT, 4–48 h; 2. anilines **13 a**–**d** or **5**, HAc, 40 °C, 2–14 d, 7–43 %; b) NH_3_, MeOH, RT, 4 d, quant. c) TFA, CH_2_Cl_2_, RT, 12 h, quant. d) *n*Bu‐NH_2_, HATU, DIPEA, DMF, RT, 56 % e) decanoyl chloride, DIPEA, THF, RT, 80 % f) alkyne, Pd(PPh_3_)_4_, CuI, NEt_3_, THF or CH_2_Cl_2_, 50–85 °C, 24 h, 20 %.

A greater diversity of 5‐diazenylnicotinamides was realizable exerting Mills reaction for the synthesis of unsymmetrical azo dyes by reaction of aromatic nitroso derivatives with anilines in glacial acetic acid. Aromatic nitroso compounds were obtained by oxidation of anilines with potassium peroxymonosulfate (oxone) yielding the methyl 5‐diazenylnicotinates **14 a**–**d** after condensation with **5** (Scheme 4). In the first place, we tried to transform the electron‐deficient aminopyridine **5** to its nitroso derivative in order to use the electron‐rich anilines **13 a**–**d** as nucleophiles in the following condensation reaction. However, transformation of **5** to its nitroso derivative by potassium peroxymonosulfate failed, probably due to the oxidative susceptibility of amino pyridines towards formation of *N*‐oxides. Consequently, we interchanged the roles of the reagents, treated **13 a**–**d** with oxone, and used the less suitable aminopyridine **5** as nucleophile in the condensation reaction. Normally, Mills reaction provides good yields in the synthesis of azobenzenes, but in the case of methyl 5‐diazenyl‐nicotinates, yields were often poor and required long reaction times caused by the said reduced nucleophilicity of **5**. Compounds **15 a**–**d** were obtained by successive ammonolysis. In the case of **14 a** and **14 c**, further derivatization was performed by acidic cleavage of the *tert*‐butyl‐based protective group, followed by amide formation and ammonolysis to **15 e** and **15 f**. Sonogashira‐type coupling of arylbromide **14 d** with terminal alkynes resulted in formation of methyl nicotinates **14 g** and **14 h** (structure not shown) in modest yield, which were transformed to **15 g** and **15 h** as described above.

**Scheme 5:**
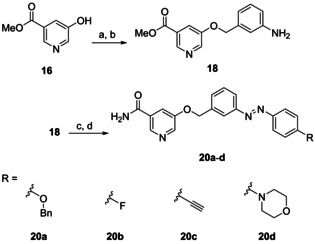
a) 3‐Nitrobenzyl bromide, Cs_2_CO_3_, DMF, RT, 61 %; b) Raney‐Ni, H_2_, THF, RT, 85 %; c) 1. Oxone, CH_2_Cl_2_, H_2_O, 2–14 h; 2. R‐NH_2_, HAc, 40 °C, 1–7 d, 23–42 %; d) NH_3_, MeOH, RT, 4 d, quant.

Based on closely related 5‐[(amidobenzyl)oxy]‐nicotinamides (**2**), we additionally synthesized a small set of analogous azobenzene photoswitches.^6^, ^18^ Compound **18** was prepared similarly to the original procedure and was converted to azo compounds **20 a**–**d** applying Mills reaction and subsequent ammonolysis (Scheme 5).

### Photochemistry

The design of photoswitchable drugs requires careful consideration of their photophysical and photochemical properties under physiological conditions. With water being the solvent of choice for all pharmaceutically relevant systems, it had to be considered that hydrogen bonding in polar solvents strongly affects thermal relaxation rates and may lead to rapid thermal *Z→E* isomerization in certain classes of azobenzenes.^16^ Though this can be desirable in some applications, in our case slow thermal *Z*→*E* isomerization was obligatory in order to retain the impact of short term UV irradiation.

According to their substitution pattern and spectral properties, phenylazopyridines **7 a**–**d** (2‐/4‐OH) as well as **7 e** and **f** (4‐NR_2_) can be classified as amino‐azobenzenes (aAB). In this sort of heteroarylic azo dyes, the rate of thermal isomerization is intensively increased by hydrogen bonding and tautomerization. Consequently, aAB undergo thermal *Z*→*E* isomerization within milliseconds to seconds in aqueous solution.^19^ Such rapid transformations could not be captured by our experimental set‐up. Noteworthy, in aprotic and less polar acetonitrile thermal *Z*→*E* isomerization of compound **7 a** was notably decelerated, showing a half‐life of 94 min in contrast to its constitutional isomer **7 b**, that exerted rapid thermal isomerization. We suggest that the o*rtho* methyl groups in **7 a** impeded isomerization leading to delayed thermal relaxation.

**Figure 2:**
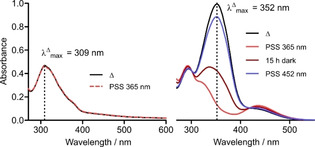
Left: **20 a** (50 μm) in assay buffer (5 % DMSO, *v/v*) at thermal equilibrium (Δ) and PSS after 365 nm irradiation (red). Due to aggregation of the chromophore, general light absorption is diminished and λΔmax appears blue‐shifted. Right: **20 a** (50 μm) in assay buffer (50 % DMSO, *v/v*) at thermal equilibrium (Δ), directly after 365 nm illumination (red) as well as additional 15 h in the dark (dark red). After 452 nm irradiation (blue) PSS displays almost complete transformation to thermal equilibrium (Δ). Under these conditions, aggregation of the compound is prevented, and photoisomerization proceeds unhindered.

In order to increase the thermal half‐life of the *Z* isomers, we performed additional structural modifications on the phenylazopyridine scaffold. However, investigation of thermal *Z*→*E* isomerization kinetics could not be performed in the exact solvent composition of the enzyme assay (i. e., 5 % DMSO (*v/v*) in assay buffer) for most of the synthesized compounds due to their high lipophilicity. In fact, many of them were prone for aggregation indicated by blue‐shifted maxima and notably decreased absorption in the UV‐Vis spectra.^20^ As a consequence, photoisomerization was hampered in the respective compounds. Higher amounts of DMSO prevented aggregation and enabled determination of *Z* isomer stability in aqueous environment as exemplified for compound **20 a** (Figure 2).

Acylation of **7 f** yielded compounds **9 a**–**k**, adopting PSS sufficiently stable for acquisition (Table 1). PSD of these compounds was approximately 90 % of the respective photoisomers, thus enabling almost perfect toggling between *Z* and *E* form. Regarding the *Z* isomer half‐life, amides **9 h**–**j** obtained from aromatic carboxylic acid chlorides were inferior to amides **9 a**–**g** and **9 k** prepared from aliphatic counterparts.

**Table 1:** Absorption maxima of azo dyes **9 a**–**k**, **15 a**–**h**, and **20 a**–**c** at thermal equilibrium (Δ), half‐lives of thermal *Z*→*E* isomerization and PSD after 365 nm and 452 nm irradiation.

	λΔmax	*t*_1/2_	*E*/*Z* [%]^[e]^	
	[nm]^[a]^	[h]^[a]^	@ 365 nm	@ 452 nm
**9 a** ^[b]^	329	6	12/88	90/10
**9 b** ^[b]^	332	8	10/90	86/14
**9 c** ^[c]^	339	16	9/91	87/13
**9 d** ^[d]^	345	21	9/91	88/12
**9 e** ^[d]^	344	22	10/90	90/10
**9 f** ^[d]^	346	21	10/90	91/9
**9 g** ^[d]^	345	26	14/86	87/13
**9 h** ^[c]^	337	0.7	14/86	91/9
**9 i** ^[c]^	337	0.6	12/88	91/9
**9 j** ^[c]^	330	0.6	29/71	93/7
**9 k** ^[c]^	339	7	14/86	89/11
**15 a** ^[c]^	325	178	31/69	94/6
**15 b** ^[c]^	332	23	6/94	90/10
**15 c** ^[b]^	329	319	11/89	86/14
**15 d** ^[b]^	332	220	9/91	91/9
**15 e** ^[c]^	326	254	40/60	89/11
**15 f** ^[d]^	333	102	5/95	79/21
**15 g** ^[c]^	355	50	25/75	89/11
**15 h** ^[c]^	350	77	23/77	92/8
**20 a** ^[c]^	352	33	4/96	80/20
**20 b** ^[c]^	324	166	2/98	83/17
**20 c** ^[c]^	340	63	14/86	85/15

[a] Solutions in DMSO/assay buffer (50 μm): [b] 5 % DMSO (*v/v*), [c] 50 % DMSO (*v/v*), [d] 90 % DMSO, (*v/v*); [e] PSD determined by HPLC analysis in MeOH/water mixtures applying isocratic conditions and evaporative light‐scattering detection (ELSD).

By inverting the amide bond, we received compound **15 a** and its derivative **15 e**. As reflected by strongly increased half‐lives of the thermal *Z*→*E* isomerization, inversion of the amide bond was beneficial for the thermal stability of the *Z* isomer, while PSD after 365 nm radiation was only about 60–70 % of the respective *Z* isomer. Insertion of a methylene bridge instead of the amide bond yielded **15 b**, **15 c**, and **15 f** exhibiting slow thermal *Z*→*E* isomerization kinetics as well as efficient switching between both states. Half‐life of the respective *Z* isomers and PSD for the ethinyl substituted **15 g** and **15 h** were still sufficient, but not as valid as in the methylene bridged compounds **15 c** and **15 f**.

Azobenzene‐based compounds **20 a**–**c** also elicited slow thermal *Z*→*E* isomerization in combination with an appropriate PSD of the respective photoisomers. Especially **20 a** and **20 b** demonstrated almost quantitative transformation to their *Z* isomers upon 365 nm irradiation. Contrarily, amino‐azobenzene (aAB) **20 d** showed rapid thermal *Z*→*E* isomerization due to 4‐NR_2_ substitution.

Conclusively, the half‐lives of the thermal *Z*→*E* isomerization as well as the PSD are not necessarily reflecting the situation in the enzyme assay mixture due to the discrepancies in the solvent compositions.^21^ Nevertheless, an approximate estimation of *Z* isomer half‐life in aqueous solutions was possible.

### Biochemistry

The effect of the compounds on deacetylase activity of three human sirtuin isotypes (Sirt1–3) was investigated by a homogeneous fluorescence‐based assay, using (*Z*)‐Lys(acetyl)‐AMC (ZMAL) as substrate. In order to determine the impact of photoisomerization, the respective compounds were irradiated (365 nm, 5 min) in DMSO prior to incubation in the enzyme assay mixture. The results of the irradiated probes were finally compared to non‐irradiated measurements. For compounds displaying rapid thermal isomerization in aqueous solution (**7 a**–**7 f**, **11**, **12 a**–**d**, **20 d**) only non‐irradiated probes were tested.

As previously published, lead **3** exhibited moderate potency for Sirt2 inhibition (IC_50_=25  μm), but at the same time represented the least‐selective hit, as Sirt3 activity was also notably affected (Table 1).^7^ Azologization of **3** yielded the direct azo analogue **7 a**. As the geometry of the molecule was unaltered by this structural modification, we expected **7 a** to display a biological activity comparable to the lead. In fact, exchange of the ethylene bridge by the azo group even improved inhibitory potency to the single digit μm range. However, isoenzyme selectivity remained unsatisfactory. In general, 5‐diazenylnicotinamides primarily inhibited Sirt2/Sirt3, whereas a distinct inhibition between Sirt2 and Sirt3 was harder to achieve. The existence and position of the two methyl groups on the phenyl substructure was found to be virtually irrelevant for biological activity as well as isoenzyme selectivity, as both the isomers **7 a**/**7 b** and compound **7 e** exerted similar activity and selectivity profiles. However, exchange of the methyl groups with methoxy ethers in **7 d** resulted in slightly improved isoenzyme selectivity with decreased Sirt3 inhibition, whereas Sirt2 inhibition was unaltered. Interestingly, aniline **7 f** exhibited the highest activity with an IC_50_ of 6 μm against Sirt2 and 9 μm against Sirt3, whereas methylation of the amino group provoked a slight decrease of Sirt2 inhibition in *N*,*N*‐dimethyl aniline derivative **7 e** indicating the relevance of the amino group for polar target interactions.

Compounds **6 f**, **11** and **12 a**–**d** confirmed that the primary amide group of the nicotinamide partial structure was essential for affinity towards Sirt1–3, as modifications on this residue provoked a dramatic loss of activity (Table 1). Hence, binding of compounds **7 a**–**f** was highly likely to occur in a fashion similar to nicotinamide itself. Nicotinamide is known to bind to the enzymatic C‐site, which is part of the catalytic cleft and is contained in all sirtuin isotypes. Thus, further interactions with specific domains were necessary in order to improve isoenzyme selectivity.

In the case of Sirt2, a hydrophobic selectivity pocket adjacent to the C‐site presents new possibilities for specific interactions.^22^ This unique binding pocket is typically formed after binding of the highly potent and selective sirtuin‐rearranging ligands (SirReals) by a conformational change to the so‐called locked open conformation. This conformational change is also observed upon binding of numerous other compounds as myristoylated substrates, for instance.^23^ A recently developed fluorescence polarization (FP)‐based binding assay was utilized to determine whether our compounds are able to prevent binding of a fluorescently labelled SirReal to the catalytic core of Sirt2.^7^ Serendipitously, competition with the fluorescent probe could be confirmed for **7 f** (Table S3). Hence, one could hypothesize that **7 f** exerts a binding mode similar to that of the SirReals and possibly is also able to introduce the same conformational change to the locked open conformation that was observed upon SirReals binding. Therefore, this scaffold seemed to be a promising structure for further derivatization.

Comparable to **7 f**, the acetylated derivative **9 a** may induce an opening of the selectivity pocket (Table S3). However, acetylation of the amino group caused a notable loss of inhibitory potency supporting the assumption that the amino group is involved in polar target interactions (Table 2). Nevertheless, as Sirt2 is known to bind and process myristoylated substrates, we anticipated a Sirt2 selective inhibition by long‐chain fatty acyl derivatives of **7 f**. In fact, extension of the acyl chain (**9 b**–**g**) enhanced inhibition of Sirt2 and yielded a potent myristic acid derivative **9 g**. However, with an increasing length of the fatty acyl chain, aqueous solubility obviously became a major concern. Indeed, Sirt3 stability was affected by **9 d**–**g** potentially due to substance precipitation during the activity assay (Table 3).

**Table 2:** Influence of stilbenoid lead **3** and **6 f**, **7 a**–**f**, **11**, **12 a**–**d**, and **20 d** on the deacetylase activity of human sirtuin isotypes Sirt1–3 determined in the fluorescence‐based ZMAL activity assay.

	Sirt1 inhibition^[a]^	Sirt2 inhibition^[a]^	Sirt3 inhibition^[a]^
**3**	27 % @ 50 μm	24.6±2.8 μm ^[b]^	41.7±2.0 μm ^[b]^
**6 f**	n.i.	n.i.	n.i.
**7 a**	14 % @ 50 μm	7.9±0.6 μm ^[b]^	9.5±0.9 μm ^[b]^
**7 b**	19 % @ 50 μm	10.8±0.6 μm ^[b]^	7.9±0.5 μm ^[b]^
**7 c**	n.i.	55 % @ 10 μm	64 % @ 10 μm
**7 d**	10 % @ 10 μm	62 % 10 μm	39 % @ 50 μm
**7 e**	17 % @ 10 μm	54 % @ 10 μm	66 % @ 10 μm
**7 f**	47 % @ 100 μm	5.8±0.7 μm ^[b]^	9.4±0.7 μm ^[b]^
**11**	n.i.	n.i.	n.i.
**12 a**	33 % @ 100 μm	45 % @ 100 μm	45 % @ 100 μm
**12 b**	n.i.	16 % @ 10 μm	11 % @ 10 μm
**12 c**	n.i.	17 % @ 10 μm	11 % @ 10 μm
**12 d**	n.i.	43 % @ 100 μm	43 % @ 100 μm
**20 d**	n.i.	49 % @ 10 μm	33 % @ 10 μm

[a] Percent inhibition relative to controls at the indicated concentration, [b] IC_50_ values (μm) with statistical limits; values are mean±SD of duplicate experiments, n.i.: no inhibition detected (<30 % @ 100 μm).

**Table 3:** Influence of azo dyes **9 a**–**k**, **15 a**–**h**, and **20 a**‐**c** on the deacetylase activity of human sirtuin isotypes Sirt1–3, determined by the fluorescence‐based ZMAL activity assay. Biological activity was investigated at the thermal equilibrium (Δ) and the PSS after 5 min of UV irradiation (365 nm).

	Sirt1 inhibition^[a]^		Sirt2 inhibition^[a]^		Sirt3 inhibition^[a]^	
	Δ	PSS [365 nm]	Δ	PSS [365 nm]	Δ	PSS [365 nm]
**9 a**	n.i.	n.i.	17 % @ 10 μm	11 % @ 10 μm	68 % @ 100 μm	52 % @ 100 μm
**9 b**	n.i.	n.i.	30 % @ 10 μm	4 % @ 10 μm	80 % @ 100 μm	52 % @ 100 μm
**9 c**	n.i.	n.i.	40 % @ 10 μm	24 % @ 10 μm	20 % @ 10 μm	3 % @ 10 μm
**9 d**	n.i.	n.i.	44.0±9.5 μm ^[b]^ (26 % @ 10 μm)	7.9±0.7 μm ^[b]^ (54 % @ 10 μm)	u.i.	u.i.
**9 e**	n.i.	n.i.	26 % @ 10 μm	78 % @ 10 μm	u.i.	u.i.
**9 f**	n.i.	n.i.	46 % @ 10 μm	65 % @ 10 μm	u.i.	u.i.
**9 g**	n.i.	n.i.	2.2±0.3 μm ^[b]^	0.9±0.2 μm ^[b]^	u.i.	u.i.
**9 h**	n.i.	n.i.	27 % @ 100 μm	52 % @ 100 μm	n.i.	32 % @ 100 μm
**9 i**	n.i.	n.i.	15 % @ 10 μm	15 % @ 10 μm	n.i.	n.i.
**9 j**	n.i.	n.i.	80 % @ 100 μm	59 % @ 100 μm	u.i.	u.i.
**9 k**	n.i.	n.i.	43 % @ 100 μm	71 % @ 100 μm	n.i.	n.i.
**15 a**	n.i.	7 % @ 10 μm	6.0±1.1 μm ^[b]^	6.8±0.4 μm ^[b]^	42 % @ 10 μm	45 % @ 10 μm
**15 b**	35 % @ 100 μm	44 % @ 100 μm	3.9±0.2 μm ^[b]^	6.9±0.6 μm ^[b]^	58 % @ 10 μm	30 % @ 10 μm
**15 c**	n.i.	6 % @ 10 μm	0.88±0.06 μm ^[b]^	2.8±0.1 μm ^[b]^	1.1±0.2 μm ^[b]^	4.0±0.7 μm ^[b]^
**15 d**	n.i.	47 % @ 100 μm	7.5±0.5 μm ^[b]^	8.4±0.9 μm ^[b]^	8.2±1.4 μm ^[b]^	13.0±2.2 μm ^[b]^
**15 e**	n.i.	10 % @ 10 μm	51 % @ 10 μm	61 % @ 10 μm	54 % @ 10 μm	49 % @ 10 μm
**15 f**	n.i.	n.i.	36.6±9.3 μm ^[b]^	1.6±0.2 μm ^[b]^	n.i.	n.i.
**15 g**	60 % @ 100 μm	54 % @ 100 μm	7.2±0.5 μm ^[b]^	11.8±1.0 μm ^[b]^	64 % @ 10 μm	22 % @ 10 μm
**15 h**	n.i.	n.i.	15.2±1.8 μm ^[b]^	37.5±5.2 μm ^[b]^	42 % @ 10 μm	18 % @ 10 μm
**20 a**	n.i.	n.i.	0.70±0.21 μm ^[b]^	1.6±0.2 μm ^[b]^	23 % @ 10 μm	24 % @ 10 μm
**20 b**	n.i.	n.i.	3.2±0.5 μm ^[b]^	6.4±0.5 μm ^[b]^	34 % @ 10 μm	47 % @ 10 μm
**20 c**	45 % @ 100 μm	62 % @ 100 μm	u.i.	6.2±0.54 μm ^[b]^	u.i.	17.2±1.57 μm ^[b]^

[a] Percent inhibition relative to controls at the indicated concentration. [b] IC_50_ values (μm) with statistical limits; values are mean ± SD of duplicate experiments. n.i.: no inhibition detected (<30 % @ 100 μm), u.i.: unspecific interactions.

Depending on the lipophilicity, UV irradiation (365 nm) showed varying effects on the biological activity of **9 a**–**g**. The three less lipophilic compounds **9 a**–**9 c** did not exhibit major differences in target engagement, yet a slight decrease of affinity towards Sirt2 and Sirt3 was observable. In contrast, **9 d** displayed an about fivefold increase of Sirt2 inhibition, which is most likely caused by the improved aqueous solubility and higher abundance of the *Z* isomer in the enzyme assay mixture. Irradiation of **9 g** even yielded sub‐micromolar inhibition of Sirt2 (IC_50_=0.9 μm), yet differential target engagement is less pronounced compared to **9 d** as affinity for Sirt2 is also increased in the *E* form of **9 g**.

In order to test the influence of nonpolar, bulky substituents, we synthesized compounds **9 h**–**k**. None of these compounds exerted potent inhibition, neither in their non‐irradiated *E* form nor after 365 nm irradiation. Consequently, a certain degree of flexibility in the hydrophobic substructure is regarded crucial for target affinity.

**Figure 3:**
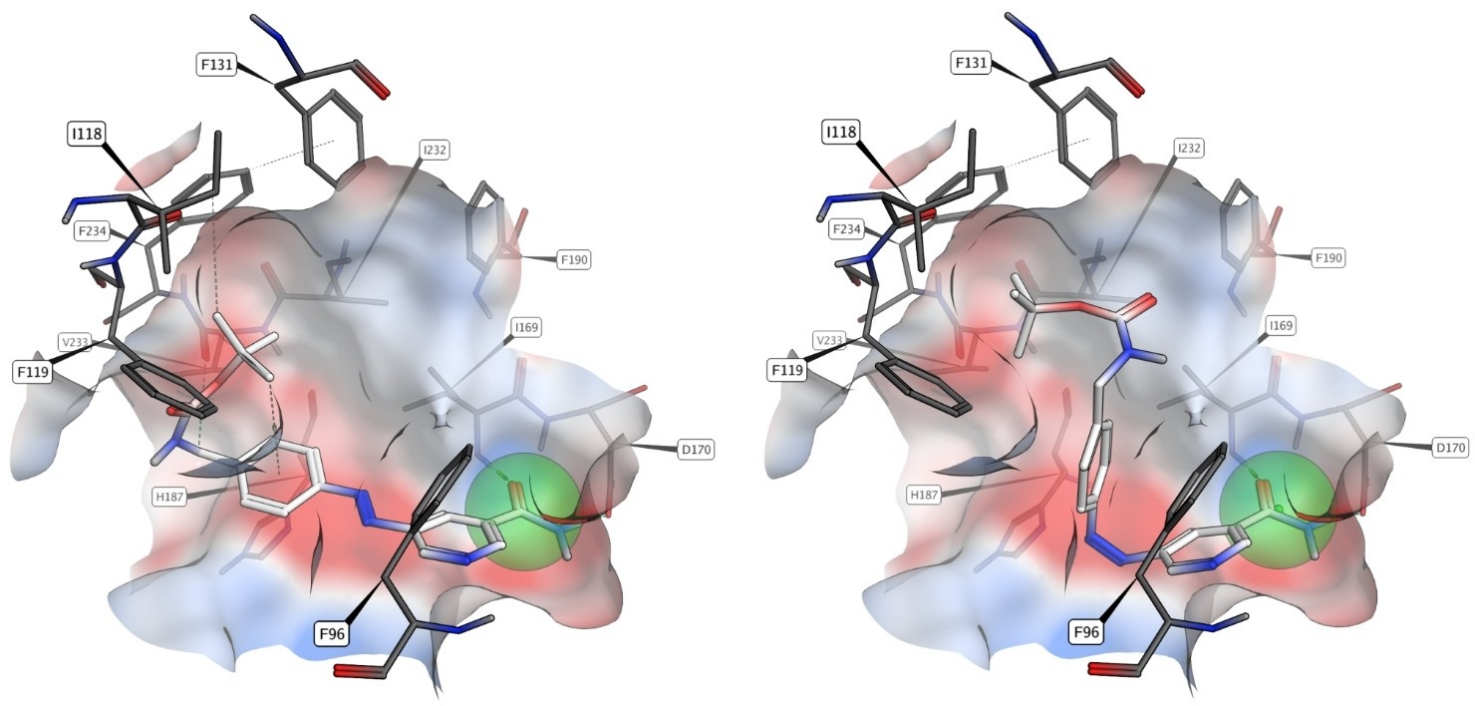
Binding pose for compound **15 c** in the *E* (left) and *Z* (right) configurations from pharmacophore‐guided docking. Because compound **15 c** is not selective for Sirt2, and modification of the primary amide group leads to complete loss of activity, binding of the nicotinamide moiety to residues I169 and D170 like NAD^+^is expected (C‐site). For (*E*)‐**15 c**, the *tert*‐butyl group points towards the hydrophobic acetyl‐lysine channel while exposing the polar carbamate towards the solvated entrance region. After photoisomerization, the phenyl group shifts to the more hydrophobic binding site, leading to unfavourable interactions for the carbamate; this might explain the decrease in activity. However, the binding of the nicotinamide moiety is not expected to be affected. The conformation of (*Z*)‐**15 c** was validated by analyses of potential energies in relation the CCNN‐dihedral angles (Figure S7). The pharmacophore region for an amide group is shown as green sphere.

Despite the detrimental effects of bulky hydrophobic substituents, we found a potent inhibition of Sirt2 and Sirt3 by *tert*‐butyl ester **15 a**. According to the previously mentioned low amount of *Z* isomers at the PSS after 365 nm irradiation, we found only negligible differences in target engagement of the irradiated probe. By insertion of a methylene bridge, we attempted to improve the photochemical behaviour of the chromophore and at the same time enhance flexibility of the hydrophobic acyl group. Indeed, **15 b** and **15 c** demonstrated both a stronger inhibition and a larger impact of irradiation compared to **15 a**. Boc‐protected 4‐aminomethyl derivative **15 c** inhibited Sirt2 within the sub‐micromolar range (IC_50_=0.9 μm). Upon irradiation, the IC_50_ value is diminished three‐ and fourfold for Sirt2 and Sirt3, respectively. Pharmacophore‐guided docking studies indicated binding of the voluminous *tert*‐butyl group to the hydrophobic acetyl‐lysine channel. However, photoisomerization is not expected to lead to substantially altered target interactions (Figure 3).

**Figure 4:**
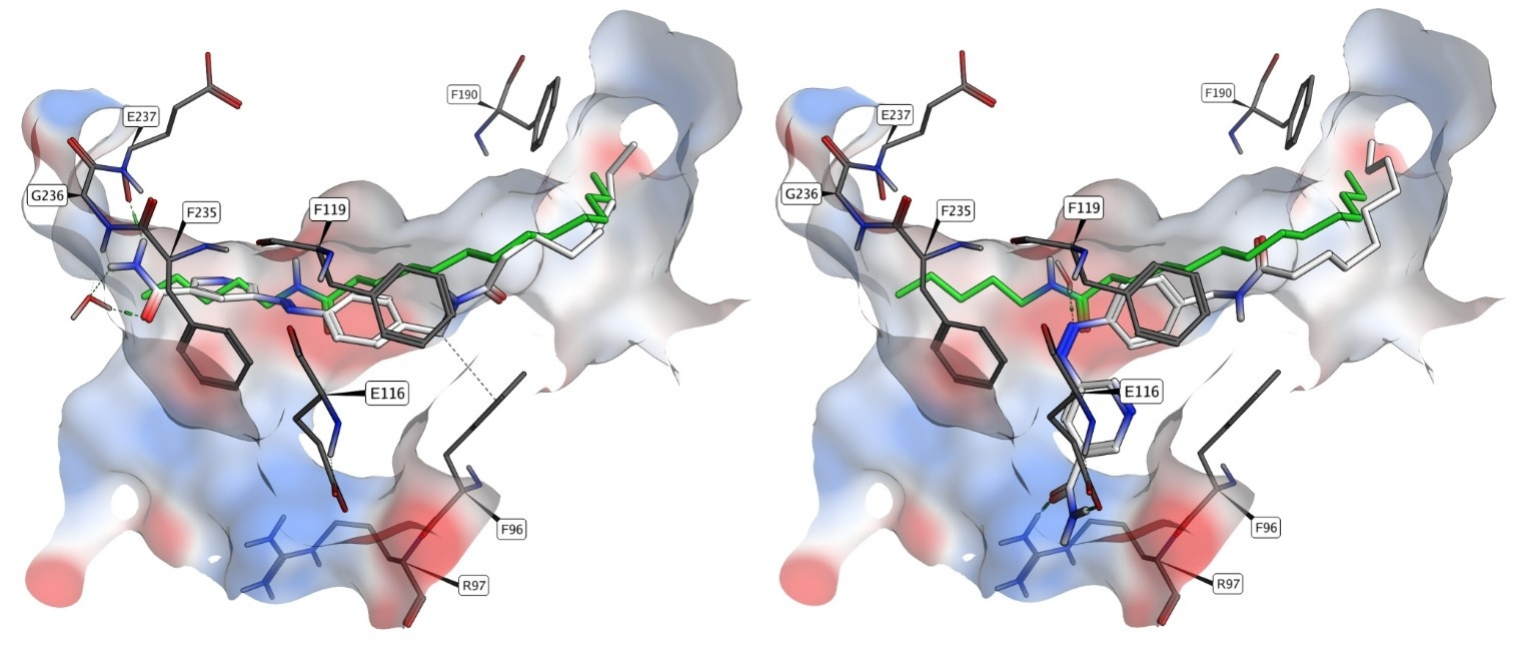
Binding pose for compound **15 f** in the *E* (left) and *Z* (right) configurations from molecular docking. The position of the decanoyl chain is in good agreement with the crystallized myristoylated lysine side chain (green) which opens the selectivity pocket (PDB ID: 4Y6L). Through photoisomerization, hydrophilicity is increased, and the polar (*Z*)‐azo group is accessible for hydrogen bond interactions with water molecules, while hiding the pyridine ring in a more hydrophobic pocket and conceiving two strong hydrogen bonds to the charged side chains of R97 and E116.

Inspired by the beneficial influence of a methylene bridge on biological activity and photochemical properties, we synthesized a methylene bridged derivative of **9 g**. In order to reduce lipophilicity, the fatty acyl chain had to be shortened by four links. Albeit, the resulting compound **15 f** exerted excellent isoenzyme selectivity, the strength of Sirt2 inhibition was dramatically diminished by this modification compared to **9 g**. However, illumination provoked a 23‐fold increase of inhibitory potency (IC_50_=1.6 μm). Besides better aqueous solubility, molecular docking studies implied favourable binding of (*Z*)‐**15 f** to be responsible for this remarkable improvement of biological activity (Figure 4).

Although insertion of a methylene bridge increased target affinity, gained flexibility at the same time reduced the overall net structural changes provoked by photoisomerization. Insertion of a more rigid ethinyl spacer instead was expected to enhance these structural changes and thus increase the impact of light‐switching.^24^ Furthermore, a polar hydroxyl group was integrated in compounds **15 g** and **15 h** in order to address the excessive lipophilicity of most compounds. However, this failed to enhance differential target engagement: Sirt2 inhibition is diminished by a factor of 1.6 for **15 g** and 2.5 for **15 h**. A similar influence is indicated regarding Sirt3.

Azologization of lead **2** yielded azobenzene‐based photoswitches **20 a**‐**d**. As in the case of lead **3**, the azo group could be inserted under full maintenance of the parent molecules geometry. Hence, we expected **20 a**–**d** to possess comparable biological activities. In fact, we obtained active sirtuin inhibitors also by this azologization approach, though not in every case the azo analogues maintained the biological activity of the respective lead structure: Compound **20 a** showed Sirt2 inhibition with an IC_50_ of 0.7 μm (Table 3), consistent with the originally published value of 0.1 μm for the respective derivative of **2** (R=OBn). Besides, inhibition of Sirt1 and Sirt3 was almost negligible, so that **20 a** approximates the excellent properties of its *N*‐arylbenzamide counterpart. On the contrary, inhibitory potency and isoenzyme selectivity of **20 b** is significantly decreased. In fact, the IC_50_ of **20 b** regarding Sirt2 inhibition is off by a factor of 100 compared to the parent structure (**2**, R=F). Also **20 c** and **20 d** did not resemble their parent compounds. Whereas the *N*‐arylbenzamides inhibit Sirt2 in the nanomolar range, **20 d** displayed Sirt2 inhibition with an IC_50_ value of about 10 μm (Table 1). In the case of **20 c**, unspecific interactions were indicated by a rather linear than sigmoidal dose response curve in the Sirt2 and Sirt3 activity assay. As this was not detected for irradiated probes of **20 c**, we assumed that solubility issues were responsible for the observed interference. In terms of their photochemical behaviour, **20 a** and **20 b** seemed promising as their PSS after UV irradiation displayed high amounts of the respective *Z* isomers. Still, both compounds did not exceed a twofold decrease of Sirt2 inhibition after illumination.

### Cell‐based activity assay

**Figure 5:**
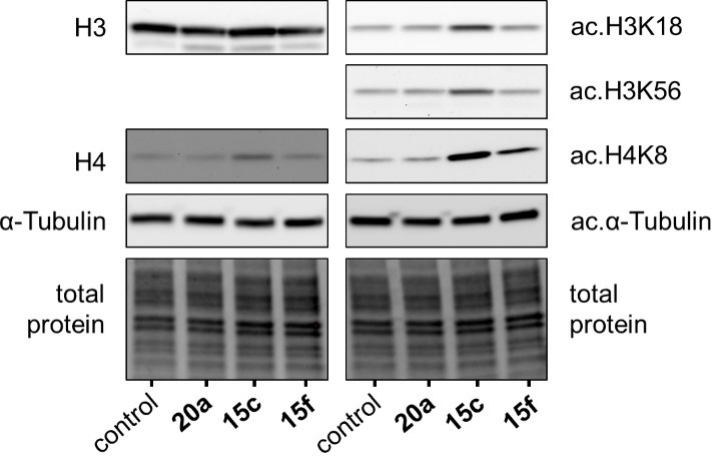
Representative western blots for total H3 resp. H4, α‐tubulin as well as specific histone acetylation sites. Compounds were irradiated by UV light (365 nm) prior to incubation with cells.

**Figure 6:**
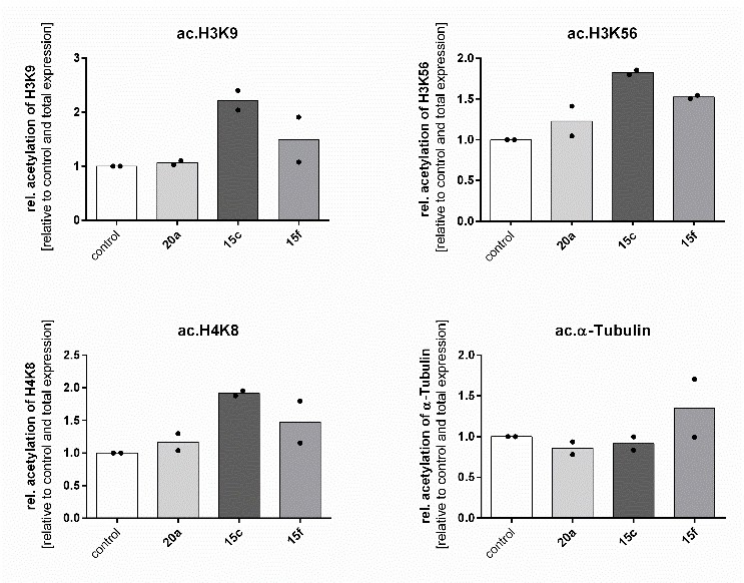
Relative acetylation levels of protein targets after incubation with compounds at 50 μm for 24 h in urinary bladder cancer cell line RT‐4 (mean of *n*=2).

Bioactivity towards a human urinary cancer cell line (RT‐4) was studied for the three most active inhibitors **15 c**, **15 f** (after UV illumination) and **20 a**. RT‐4 cells showed the highest expression of Sirt2 in our cellular inventory and therefore have been chosen for the cell‐based activity determination. In order to prove a general effect of the compounds inside the cells, we conducted preliminary studies solely for irradiated probes, since the enzyme‐based activity assay indicated either comparable activities of both photoisomers (**15 c**, **20 a**), or a higher activity of the *Z* isomer. Reductive stability of **15 c**, **15 f** and **20 a** in the presence of the cellular reductant glutathione (5 mm) was confirmed by HPLC analysis (Figure S5, Table S2). For the cell‐based activity assay, we illuminated the compounds solved in DMSO with UV light (365 nm) prior to incubation with the cells. The compiled western blots displayed that all three compounds (**20 a**, **15 c**, **15 f**) had no effect on the expression of total histone H3 and total α‐tubulin protein but in contrast, a slight increase of total H4 was detectable (Figure 5). As expected, **15 c** and **15 f** increased the levels of acetylated lysines H3K18 and H3K56, which are both affirmed substrates of Sirt2. In addition, we found H4K8 hyperacetylation even though this site is usually not affected by sirtuin catalysed deacetylation, indicating a further mode of action. In contrast, none of the compounds influenced the acetylation level of α‐tubulin, which is frequently consulted as it is specifically processed by Sirt2. However, also in a cell‐based activity assay of the highly potent Sirt2 inhibitor SirReal2 the degree of α‐tubulin hyperacetylation was less pronounced when examined by western blot technique. Instead, applying an immunofluorescence assay yielded clearer results.^22^ Surprisingly, compound **20 a** did not exert any activity regarding the examined substrates. In Figure 6, we show the relative results, whereby the acetylation product was related to total target protein and to the vehicle control. Again, we found an increase in acetylated histones induced by **15 c** and **15 f** which proves activity of these two compounds in living cells.

## Conclusions

In this work, azologization of two different lead structures was performed in order to obtain azobenzene and azopyridine‐based photoswitchable sirtuin inhibitors. Despite the high potential of diarylmaleimides and indolylfulgides as photoswitchable sirtuin inhibitors, we anticipated azo dyes to be more convenient, as this class of molecular photoswitches is known to possess reliable and highly tuneable photophysical properties also under physiological conditions. Indeed, azologization proved successful, since we obtained biologically active compounds, whose configuration could be reversibly toggled between *E* and *Z* form by UV (365 nm) or blue (452 nm) light irradiation. However, differential target engagement of the respective photoisomers was not as profound as anticipated. Whereas several photoswitchable drugs show a ten‐ to 50‐fold difference in activity, in our case differential inhibition did not exceed a factor of 3–4 for most compounds.^25^ Our docking studies implied that, despite the considerable structural change in the azo core, flexibility of the overall ligand structure could be responsible for the minor differences in bioactivity of the photoisomers. Furthermore, photoisomerization did not result in clearly unfavourable interactions between the ligand and residues of the sirtuins active site. Consequently, further rigidification and the introduction of sterically demanding groups could potentially be favourable in this regard. In contrast, pronounced effects were observed by photoswitching of highly lipophilic derivatives. Due to poor aqueous solubility, **15 f** exerted only moderate, yet selective Sirt2 inhibition (IC_50_=37 μm) in its thermally stable *E* form. Since polarity is significantly enhanced by photoisomerization, biological activity of **15 f** could be raised by a factor of 23 (IC_50_=1.6 μm) for Sirt2 inhibition. Additionally, activity of (*Z*)‐**15 f** was confirmed by increased acetylation levels of Sirt2 specific histones. Obviously, this effect is caused rather by a light‐mediated improvement of the aqueous solubility than by altered binding affinity, as anticipated in the classical sense of photoswitchable drugs. This aspect of azo‐based photoswitches has been studied recently and was exploited in the use of photochromic azo‐combrestatin A‐4 analogues.^26^ In the latter case, photoswitching to the bioactive *Z* isomers provoked an up to 550‐fold increase of bioactivity. Interestingly, in the same work the authors observed a longer *Z* isomer half‐life of less soluble compounds in watery environment, potentially contributing to the remarkable impact of photoisomerization.

Even though photoisomerization of **15 f** and other lipophilic compounds was hampered by chromophore aggregation in aqueous environment, solubility of these compounds should be enhanced in physiological media as blood, mediated by binding to albumin proteins. In this case, switching of an albumin‐bound compound to the *Z* isomer could provoke its liberation in this specific area, thereby enabling tissue specific pharmacotherapy.

## Experimental Section

**General procedure for azo coupling of phenols**: Methyl 5‐aminonicotinate (1.0 equiv.) was suspended in aqueous HCl (6  m, 3.0 equiv.) and cooled to 0 °C. An aqueous solution of NaNO_2_ (2.5 m, 1.0 equiv.) was added dropwise, so that the temperature did not exceed 5 °C. The resulting yellow solution was added slowly under stirring to the appropriate phenol (1.1 equiv.) dissolved in aqueous NaOH (2 m). If necessary, aqueous NaOH (2 m) was added to keep the resulting solution alkaline. After complete addition, the reaction mixture was acidified with aqueous HCl (6 m) and extracted with EtOAc. The combined organic extracts were washed with brine, dried over MgSO_4_, filtrated and freed from solvent. The crude product was purified by silica gel column chromatography.

**General procedure for azo coupling of anilines**: Diazotization of methyl 5‐aminonicotinate was carried out as described above. The yellow solution containing the diazonium salt was added slowly under stirring to the appropriate aniline (1.1 equiv.) dissolved in HCl (1 m). After complete addition, the reaction mixture was stirred for 10 min, then basified by using a saturated aqueous solution of Na_2_CO_3_ and extracted with EtOAc. The combined organic extracts were washed with brine, dried over MgSO_4_, filtered and freed from solvent. The crude product was purified by silica gel column chromatography.

**General procedure for synthesis of nicotinamides from methyl nicotinates**: The respective methyl nicotinate was treated with a saturated solution of ammonia in anhydrous MeOH (30 mL) and stirred in a sealed vessel at 40 °C until thin layer chromatography indicated complete conversion of the starting material (three to four days). The solvent was evaporated under reduced pressure and the residue washed sparingly with cold acetonitrile.

**Photochemistry**: All photoisomerization experiments were conducted under ruby light of 630 nm and total exclusion of daylight. Illumination was executed using a Bio‐Link 254 Crosslinker from Vilber‐Lourmat equipped with six Vilber‐Lourmat T8‐L lamps (8 W, 365 nm). Visible light radiation of 630 nm (ruby) and 452 nm (blue) was derived from a Paulmann FlexLED 3D strip. All compounds were irradiated in solution using spectrophotometric grade solvents. Photoisomerization and UV/Vis‐spectra measurement was conducted in quartz glass cuvettes (114‐QS, Hellma Analytics) at room temperature.

**Cloning, expression and purification of recombinant proteins**: Expression and purification of Sirt1_133−747_, Sirt2_56−356_, and Sirt3_118−395_ was carried out as described previously.^27^ Identity and purity were verified by SDS‐PAGE. Protein concentration was determined by the Bradford assay.^28^ Deacylase activity of sirtuin isotypes could be inhibited with nicotinamide and was shown to be NAD^+^‐dependent.

**Fluorescence‐based activity assay**: The inhibitory effect of compounds on Sirt1–3 was detected *via* a previously reported fluorescence‐based assay.^29^ The synthetic substrate (*Z*)‐Lys(acetyl)‐AMC (ZMAL) is deacetylated by sirtuins, followed by tryptic digestion and thereby release of 7‐aminomethylcoumarin, leading to a fluorescent readout. Inhibition was determined by comparing percentage substrate conversion to a DMSO control after subtraction of the blank fluorescence signal. All compounds were tested at 100 or 50 μm and 10 μm respectively. For compounds that showed more than 70 % inhibition at 10 μm an IC_50_ value was determined. IC_50_ values were calculated with OriginPro 9.0 G using a non‐linear regression to fit the dose response curve (Figure S6). An enzyme‐free blank control and a 100 % conversion control using AMC instead of ZMAL were measured as well. Inhibition measurements were performed in biological duplicates for all compounds.

**Molecular modelling**: All calculations were performed by using the Molecular Operating Environment (MOE) software suite (version 2019.01).^30^ If not explicitly stated otherwise, default settings and parameters were used.

*General preparation*: For molecular docking of ligands to human Sirt2, a crystal structure with open‐state selectivity pocket and bound ligand was used (PDB IDs: 5MAT, 4RMG). Both systems were prepared by applying AMBER14 force field parameters, adding hydrogen atoms and protonation with Protonate3D (pH 7.4). Missing flexible protein loops were rebuilt, followed by a restraint minimization of the protein‐ligand complex.

*Molecular docking*: Ligands used for docking experiments were prepared by generating three‐dimensional structures from SMILES, taking into account possible protonation states and both *E*/*Z* isomers for azo groups. AM1‐BCC charges were applied. The binding site was defined as all residues within 4.5 Å of the bound ligand in the crystal structure. Docking was performed using a two‐stage protocol with placement of 30 poses by Triangle Matcher and London dG scoring, followed by an Induced Fit refinement and more accurate MM/GBVI scoring. For each ligand, a total of 10 final poses were obtained and visually inspected. For pharmacophore‐guided molecular docking, a sphere with 1.7 Å was placed on the amide group of NAD^+^(PDB: 4RMG), which was encoded by the SMARTS expression “[#7X3H2][#6X3](=[#8X1])[#6]”.

## Conflict of interest

The authors declare no conflict of interest.

## Supporting information

As a service to our authors and readers, this journal provides supporting information supplied by the authors. Such materials are peer reviewed and may be re‐organized for online delivery, but are not copy‐edited or typeset. Technical support issues arising from supporting information (other than missing files) should be addressed to the authors.

Supplementary
