# Supplementary material for: Activation of Sirtuin 2 Inhibitors Employing Photoswitchable Geometry and Aqueous Solubility
Source: ChemMedChem. 2020 May 7;15(15):1480–9. doi: 10.1002/cmdc.202000148 (PMC7496931; doi:10.1002/cmdc.202000148)

ChemMedChem

Supporting Information

Activation of Sirtuin 2 Inhibitors Employing Photoswitchable Geometry and Aqueous Solubility

Christoph W. Grathwol, Nathalie Wössner, Steven Behnisch-Cornwell, Lukas Schulig, Lin Zhang, Oliver Einsle, Manfred Jung, and Andreas Link*© 2020 The Authors. Published by Wiley-VCH Verlag GmbH & Co. KGaA.
This is an open access article under the terms of the Creative Commons Attribution License, which permits use, distribution and reproduction in any medium, provided the original work is properly cited.

Author Contributions

C.G. Conceptualization:Equal; Investigation:Lead; Methodology:Lead; Validation:Equal; Writing - Original Draft:-Lead
N.W. Investigation:Supporting; Validation:Equal; Writing - Review & Editing:Supporting
S.B.-C. Investigation:Supporting; Writing - Review & Editing:Supporting
L.S. Investigation:Supporting; Software:Lead; Visualization:Equal
L.Z. Investigation:Supporting
O.E. Funding acquisition:Supporting; Investigation:Supporting; Resources:Supporting
M.J. Funding acquisition:Supporting; Supervision:Supporting; Writing - Review & Editing:Supporting
A.L. Conceptualization:Equal; Supervision:Lead; Writing - Review & Editing:Equal

Table of Content

General Remarks 1

Experimental Procedures and Data 2

Irradiation Setup 34

UV/Vis Spectra and Half-Life of Thermal Isomerization 35

Photostationary Distribution 40

Reductive Stability 41

Fluorescence-Based Activity Assay (ZMAL-Assay) 42

FP-Based Binding Assay 43

Cell-Based Activity Assay 45

Dihedral Analysis 46

NMR Spectra 47

General Remarks

All solvents and reagents were obtained from commercial suppliers and were used without purification. Anhydrous solvents were purchased from Acros Organics. Thin layer chromatography (TLC) was executed on silica gel 60 F_{254} aluminium plates purchased from Merck. Visualization of compounds, if not dyes themselves, was accomplished by UV-light (254 nm and 366 nm) or by staining with iodine or vanillin/sulfuric acid (3.0 g vanillin and 0.5 mL H_2SO_4 in 100 mL EtOH) reagent. Chromatographic purification of products was performed by flash chromatography on silica gel (20–45 µm, Carl Roth) applying pressured air up to 0.8 bar. NMR spectra were recorded on a Bruker Avance III instrument (^{1}H NMR: 400 MHz, ^{13}C NMR: 100.6 MHz). Chemical shifts were referenced to tetramethylsilane (TMS) as internal standard in deuterated solvents and reported in parts per million (ppm). Coupling constants (*J*) are reported in Hz using the abbreviations: br = broad, s = singlet, d = doublet, t = triplet, q = quartet, m = multiplet and combinations thereof. Infrared (IR-) spectra were recorded on a Bruker Alpha FT-IR spectrometer equipped with a diamond ATR unit and are indicated in terms of absorption frequency [cm^{-1}]. Melting points were measured in open capillary tubes using a Melting Point M-565 apparatus from Büchi and are uncorrected. High accuracy mass spectra were recorded on a Shimadzu LCMS-IT-TOF using ESI ionization. Purity of final compounds was determined by HPLC with DAD (applying the 100% method at 220 nm and 254 nm). Preparative and analytical HPLC were performed using Shimadzu devices CBM-20A, LC-20A P, SIL-20A, FRC-10A with SPD 20A UV/Vis detector and an ELSD-LTⅡ. In analytical mode a LiChroCART® (250×4 mm) and in preparative mode a Hibar® RT (250×25 mm) column were used, both containing LiChrospher® 100 RP-18e (5 µm). UV/Vis-spectra were obtained using a Thermo Scientific Genesys 10S UV-VIS spectrophotometer.

Experimental Procedures and Data

Methyl (*E*)-5-[(4-hydroxy-2,6-dimethylphenyl)diazenyl]nicotinate (**6a**)

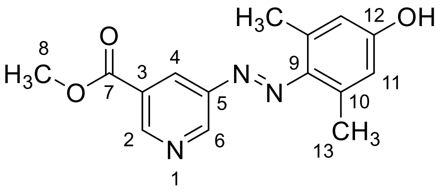

Synthesis was conducted according to the general procedure for azo coupling reactions using **5** (500 mg, 3.29 mmol, 1.0 equiv.) and 3,5-dimethylphenol (402 mg, 3.29 mmol, 1.0 equiv.). The product was obtained as red solid (409 mg, 1.43 mmol, 43 %): R_f = 0.52 (hexanes/EtOAc 1:1); mp: 199.5 °C; **^{1}H NMR, H,H-COSY (400 MHz, DMSO-*d$_6$*):** δ (ppm) = 10.21 (s, br, 1H, 12-*O*-H), 9.18 (d, *J* = 2.3 Hz, 1H, 6-H), 9.12 (d, *J* = 1.9 Hz, 1H, 2-H), 8.39 (pseudo-t, *J* = 2.1 Hz, 1H, 4-H), 6.63 (s, 2H, 11-H), 3.94 (s, 3H, 8-H), 2.48 (s, 6H, 13-H); **^{13}C NMR, DEPT135, HSQC, HMBC (101 MHz, DMSO-*d$_6$*):** δ (ppm) = 164.8 (7), 159.9 (12), 150.3 (2), 148.8 (6), 147.8 (5), 142.0 (9), 136.9 (10), 126.6 (4), 126.3 (3), 116.3 (11), 52.6 (8), 20.7 (13); **IR (ATR):** *v* (cm^{-1}) = 2959, 2916, 1722, 1606, 1573, 1474, 1402, 1279, 1221, 1141, 763.

Methyl (*E*)-5-[(4-hydroxy-3,5-dimethylphenyl)diazenyl]nicotinate (**6b**)

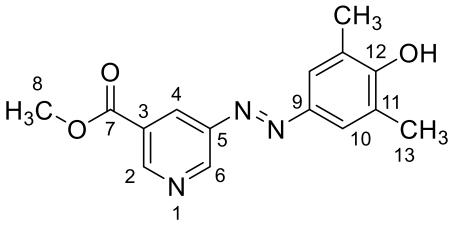

Synthesis was conducted according to the general procedure for azo coupling reactions using **5** (500 mg, 3.29 mmol, 1.0 equiv.) and 2,6-dimethylphenol (402 mg, 3.29 mmol, 1.0 equiv.). The product was obtained as orange solid (405 mg, 1.42 mmol, 43 %): R_f = 0.66 (hexanes/EtOAc 1:1); mp: 215.0 °C; **^{1}H NMR, H,H-COSY (400 MHz, DMSO-*d$_6$*):** δ (ppm) = 9.37 (s, br, 1H, 12-*O*-H), 9.24 (d, *J* = 2.3 Hz, 1H, 6-H), 9.13 (d, *J* = 1.9 Hz, 1H, 2-H), 8.43 (pseudo-t, *J* = 2.1 Hz, 1H, 4-H), 7.63 (s, 2H, 10-H), 3.94 (s, 3H, 8-H), 2.27 (s, 6H, 13-H); **^{13}C NMR, DEPT135, HSQC, HMBC (101 MHz, DMSO-*d$_6$*):** δ (ppm) = 164.8 (7), 158.1 (12), 150.6 (2), 149.2 (6), 147.1 (5), 144.9 (9), 126.3 (3), 125.9 (4), 125.1 (11), 124.0 (10), 52.6 (8), 16.6 (13); **IR (ATR):** *v* (cm^{-1}) = 3077, 2948, 1722, 1589, 1409, 1305, 1259, 1204, 1096, 765, 664.

Methyl (*E*)-5-[(2-hydroxynaphthalen-1-yl)diazenyl]nicotinate (**6c**)

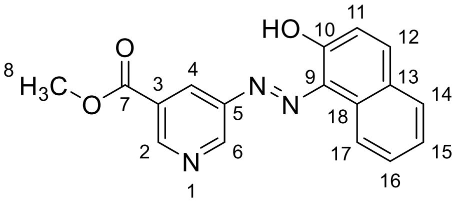

Synthesis was conducted according to the general procedure for azo coupling reactions using **5** (800 mg, 5.26 mmol, 1.0 equiv.) and 2-naphthole (834 mg, 5.79 mmol, 1.1 equiv.). The crude product was purified by silica gel chromatography (cyclohexane/EtOAc 2:1), which yielded **6c** as red crystals (628 mg, 2.04 mmol, 39 %): R_f = 0.41 (cyclohexane/EtOAc 2:1); mp: 192.9 °C; **^{1}H NMR, H,H-COSY (400 MHz, Benzene-*d$_6$*):** δ (ppm) = 15.03 (s, br, 1H, 10-*O*-H), 8.64 (d, *J* = 1.8 Hz, 1H, 6-H), 8.14 (d, *J* = 2.5 Hz, 1H, 2-H), 7.88 (d, *J* = 8.2 Hz, 1H, 17-H), 7.72 (dd, *J* = 2.4, 2.0 Hz, 1H, 4-H), 6.64 (ddd, *J* = 8.3, 7.0, 1.4 Hz, 1H, 16-H), 6.60–6.56

(m, 1H, 14-H), 6.55–6.47 (m, 2H, 15-H, 12-H), 6.15 (d, *J* = 9.4 Hz, 1H, 11-H), 2.81 (s, 3H, 8-H); **^{13}C NMR, DEPT135, HSQC, HMBC (101 MHz, Benzene-*d_6*):** δ (ppm) = 169.6 (10), 165.1 (7), 149.1 (6), 145.4 (2), 142.1 (5), 140.5 (16), 133.7 (18), 131.3 (9), 129.4 (14/15/16), 128.9 (14/15/16), 128.7 (13), 127.0 (3), 126.3 (14/15/16), 125.3 (4), 124.0 (11), 122.4 (17), 51.9 (8); **IR (ATR):** *v* (cm^{-1}) = 2952, 1724, 1614, 1283, 1240, 1207, 1093, 862, 755, 688, 505.

Methyl (*E*)-5-[(4-hydroxy-3,5-dimethoxyphenyl)diazenyl]nicotinate (**6d**)

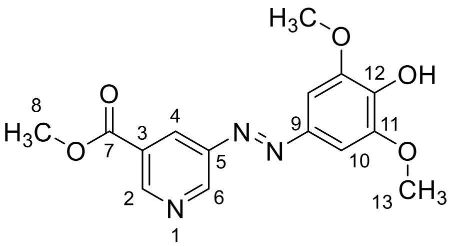

Synthesis was conducted according to the general procedure for azo coupling reactions using **5** (380 mg, 2.50 mmol, 1.0 equiv.) and 2,6-dimethoxyphenol (385 mg, 2.50 mmol, 1.0 equiv.). The crude product was purified by silca gel column chromatography (100 % DCM → DCM/MeOH 9:1), which yielded **6d** as orange solid (498 mg, 1.57 mmol, 31 %): R_f = 0.25 (cyclohexane/EtOAc 1:1); mp: 219.2 °C; **^{1}H NMR, H,H-COSY (400 MHz, DMSO-*d_6*):** δ (ppm) = 9.60 (s, br, 1H, 12-*O*-H), 9.29 (d, *J* = 1.8 Hz, 1H, 6-H), 9.15 (d, *J* = 1.2 Hz, 1H, 2-H), 8.49 (s, 1H, 4-H), 7.63 (s, 2H, 10-H), 7.35 (s, 2H, 10-H), 3.94 (s, 3H, 8-H), 3.90 (s, 6H, 13-H); **^{13}C NMR, DEPT135, HSQC, HMBC (101 MHz, DMSO-*d_6*):** δ (ppm) = 164.7 (7), 150.7 (2), 150.0 (6), 148.1 (11), 146.9 (5), 144.0 (12), 140.8 (9), 126.3 (3), 126.0 (4), 101.3 (10), 56.0 (13), 52.6 (8); **IR (ATR):** *v* (cm^{-1}) = 2999, 2954, 1724, 1602, 1575, 1507, 1471, 1269, 1204, 1110, 687.

Methyl (*E*)-5-{[4-(dimethylamino)phenyl]diazenyl}nicotinate (**6e**)

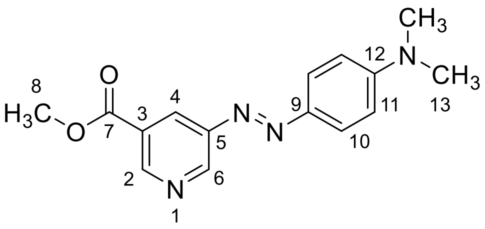

Synthesis was conducted according to the general procedure for azo coupling reactions using **5** (1.00 g, 6.57 mmol, 1.0 equiv.) and *N*,*N*-dimethylaniline (876 mg, 7.23 mmol, 1.1 equiv.). The crude product was purified by silca gel column chromatography (DCM/MeOH 98:2), which yielded **6e** as orange solid (1.21 g, 4.26 mmol, 65 %): R_f = 0.27 (DCM/MeOH 98:2); mp: 119.1 °C; **^{1}H NMR, H,H-COSY (400 MHz, DMSO-*d_6*):** δ (ppm) = 9.20 (d, *J* = 2.3 Hz, 1H, 6-H), 9.08 (d, *J* = 2.0 Hz, 1H, 2-H), 8.43 (m, 1H, 4-H), 7.86 (m, 2H, 10-H), 6.86 (m, 2H, 11-H), 3.93 (s, 3H, 8-H), 3.10 (s, 6H, 13-H); **^{13}C NMR, DEPT135, HSQC, HMBC (101 MHz, DMSO-*d_6*):** δ (ppm) = 164.9 (7), 153.2 (12), 150.0 (2), 147.5 (5), 142.6 (9), 126.2 (3), 125.6 (4), 125.4 (10), 111.5 (11), 52.6 (8); **IR (ATR):** *v* (cm^{-1}) = 3040, 2114, 1726, 1602, 1577, 1518, 1300, 1284, 1137, 1119, 823.

Methyl (*E*)-5-[(4-amino-3,5-dimethylphenyl)diazenyl]nicotinate (**6f**)

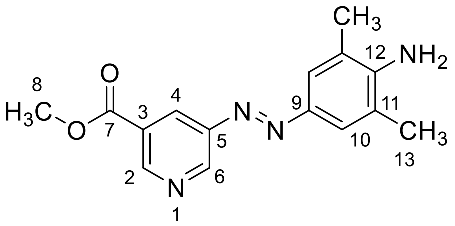

Synthesis was conducted according to the general procedure for azo coupling reactions using **5** (1.00 g, 6.57 mmol, 1.0 equiv.) and 2,6-dimethylaniline (786 mg, 7.23 mmol, 1.1 equiv.). The product was obtained **6f** as red solid (920 mg, 3.24 mmol, 52 %): R_f = 0.56 (DCM/MeOH 95:5); mp: 190.6 °C; **^{1}H NMR, H,H-COSY (400 MHz, DMSO-*d₆*):** δ (ppm) = 9.16 (d, *J* = 2.3 Hz, 1H, 6-H), 9.05 (d, *J* = 2.0 Hz, 1H, 2-H), 7.56 (pseudo-t, *J* = 2.2 Hz, 1H, 4-H), 7.56 (s, 2H, 10-H), 3.93 (s, 3H, 8-H), 2.20 (s, 6H, 13-H); **^{13}C NMR, DEPT135, HSQC, HMBC (101 MHz, DMSO-d_6):** δ (ppm) = 164.9 (7), 150.2 (9), 149.6 (6), 149.4 (2), 147.6 (5), 142.6 (12), 126.2 (3), 125.4 (4), 124.4 (10), 120.6 (11), 52.5 (8), 17.7 (13); **IR (ATR):** *v* (cm^{-1}) = 3463, 3339, 3217, 1712, 1654, 1599, 1385, 1262, 1120, 765, 694.

(*E*)-5-[(4-hydroxy-2,6-dimethylphenyl)diazenyl]nicotinamide (**7a**)

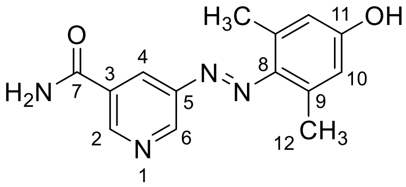

Synthesis was conducted according to the general procedure for the synthesis of nicotinamides from methyl nicotinates using **6a** (165 mg, 0.58 mmol, 1.00 equiv.). The product was obtained as red solid (150 mg, 0.55 mmol, 95 %): R_f = 0.63 (DCM/MeOH 90:10); mp: 259.7 °C; **^{1}H NMR, H,H-COSY (400 MHz, DMSO-d_6):** δ (ppm) = 10.15 (s, br, 11-*O*-H), 9.10 (pseudo-t, *J* = 1.9 Hz, 2H, 2-H, 6-H), 8.44 (pseudo-t, *J* = 2.2 Hz, 1H, 4-H), 8.35 (s, br, 1H, *N*-H), 7.74 (s, br, 1H, *N*-H), 6.64 (s, 2H, 10-H), 2.49 (s, 6H, 12-H); **^{13}C NMR, DEPT135, HSQC, HMBC (101 MHz, DMSO-d_6):** δ (ppm) = 165.9 (7), 159.6 (11), 149.4 (2/6), 147.8 (5), 147.0 (2/6), 142.2 (8), 136.5 (9), 130.5 (3), 125.6 (4), 116.2 (10), 20.6 (12); **IR (ATR):** *v* (cm^{-1}) = 3366, 3191, 1667, 1599, 1573, 1316, 1279, 1139, 843, 633, 581; **ESI-HRMS:** calcd. for $[C_{14}H_{14}N_4O_2+H]^+$ 270.1117, found 270.1112; **Comp. Purity (220 nm):** 100 %.

(*E*)-5-[(4-hydroxy-3,5-dimethylphenyl)diazenyl]nicotinamide (**7b**)

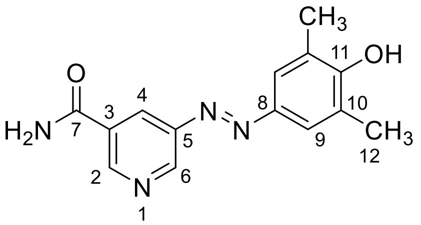

Synthesis was conducted according to the general procedure for the synthesis of nicotinamides from methyl nicotinates using **6b** (112 mg, 0.39 mmol, 1.0 equiv.). The product was obtained as yellow solid (105 mg, 0.39 mmol, 100 %): R_f = 0.47 (DCM/MeOH 90:10); mp: 166.4 °C; **^{1}H NMR, H,H-COSY (400 MHz, DMSO-d_6):** δ (ppm) = 9.33 (s, br, 1H, 12-*O*-H), 9.14 (d, *J* = 2.2 Hz, 1H, 6-H), 9.11 (d, *J* = 2.0 Hz, 1H, 2-H), 8.50 (pseudo-t, *J* = 2.2 Hz, 1H, 4-H), 8.34 (s, br, 1H, *N*-H), 7.74 (s, br, 1H, *N*-H), 7.64 (s, 2H, 10-H), 2.28 (s, 6H, 12-H); **^{13}C NMR, DEPT135, HSQC, HMBC (101 MHz, DMSO-d_6):** δ (ppm) = 165.9 (7), 158.0 (11), 149.6 (2), 147.8 (6), 147.1 (5), 145.0 (10), 130.5 (3), 125.3 (4), 125.1 (9), 123.8 (8), 16.6 (12); **IR (ATR):** *v* (cm^{-1}) = 3432,

2972, 1691, 1670, 1590, 1397, 1292, 1184, 1152, 696, 549, 487; **ESI-HRMS:** calcd. for $[C_{14}H_{14}N_4O_2+H]^+$ 270.1117, found 270.1116; **Comp. Purity (220 nm):** 100 %.

(*E*)-5-[(2-hydroxynaphthalen-1-yl)diazenyl]nicotinamide (**7c**)

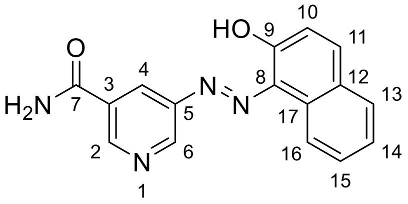

Synthesis was conducted according to the general procedure for the synthesis of nicotinamides from methyl nicotinates using **6c** (100 mg, 0.33 mmol, 1.00 equiv.). The product was obtained as orange solid (96 mg, 0.33 mmol, 100 %): R_f = 0.55 (EtOAc/MeOH 95:5); mp: 260.0 °C; **^{1}H NMR, H,H-COSY (400 MHz, DMSO-*d*$_6$):** δ (ppm) = 15.39 (s, br, 1H, 9-*O*-H), 9.19 (d, *J* = 2.4 Hz, 1H, 6-H), 8.97 (d, *J* = 1.9 Hz, 1H, 2-H), 8.66–8.58 (m, 2H, 4-H, 16-H), 8.39 (s, br, 1H, *N*-H), 8.00 (d, *J* = 9.4 Hz, 1H, 11-H), 7.84–7.74 (m, 2H, *N*-H, 13-H), 7.70–7.61 (m, 1H, 15-H), 7.55–7.45 (m, 1H, 14-H), 6.93 (d, *J* = 9.4 Hz, 1H, 10-H); **^{13}C NMR, DEPT135, HSQC, HMBC (101 MHz, DMSO-*d*$_6$):** δ (ppm) = 170.0 (9), 165.7 (7), 146.9 (2), 143.7 (6), 141.1 (5), 141.0 (10), 132.4 (8), 130.4 (3/12), 130.1 (3/12), 129.3 (15), 129.0 (13), 128.0 (17), 126.3 (14), 124.0 (11), 123.1 (4), 121.8 (16); **IR (ATR):** *v* (cm^{-1}) = 3347, 3174, 1666, 1618, 1499, 1482, 1448, 1255, 1223, 950, 913, 515; **ESI-HRMS:** calcd. for [C16H12N4O2+H]$^+$ 292.0960, found 292.0966; **Comp. Purity (220 nm):** 100 %.

(*E*)-5-[(4-hydroxy-3,5-dimethoxyphenyl)diazenyl]nicotinamide (**7d**)

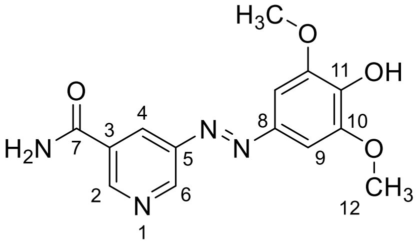

Synthesis was conducted according to the general procedure for the synthesis of nicotinamides from methyl nicotinates using **6d** (59 mg, 0.19 mmol, 1.00 equiv.). After evaporation of methanol, EtOAc was added to the residue, and heated to reflux for 5 minutes. After cooling to room temperature, the solid was collected by filtration and gave **7d** as ochre solid (50 mg, 0.17 mmol, 89 %): R_f = 0.38 (EtOAc/MeOH 95:5); mp: 250.2 °C; **^{1}H NMR, H,H-COSY (400 MHz, DMSO-*d*$_6$):** δ (ppm) = 9.60 (s, br, 1H, 11-*O*-H), 9.17 (s, 1H, 6-H), 9.11 (s, 1H, 2-H), 8.54 (s, 1H, 4-H), 8.34 (s, br, 1H, *N*-H), 7.75 (s, br, 1H, *N*-H), 7.34 (s, 2H, 9-H), 3.90 (s, 6H, 12-H); **^{13}C NMR, DEPT135, HSQC, HMBC (101 MHz, DMSO-*d*$_6$):** δ (ppm) = 165.8 (7), 149.5 (2), 148.2 (11), 147.6 (6), 146.8 (5), 143.9 (11/10), 140.6 (11/10), 130.4 (3), 125.3 (4), 101.1 (9), 56.0 (12); **IR (ATR):** *v* (cm^{-1}) = 3385, 3209, 1664, 1609, 1578, 1509, 1278, 1250, 1113, 847, 691, 629; **ESI-HRMS:** calcd. for $[C_{14}H_{14}N_4O_4+H]^+$ 302.1015, found 302.1016; **Comp. Purity (254 nm):** 96.5 %.

(*E*)-5-{[4-(dimethylamino)phenyl]diazenyl}nicotinamide (**7e**)

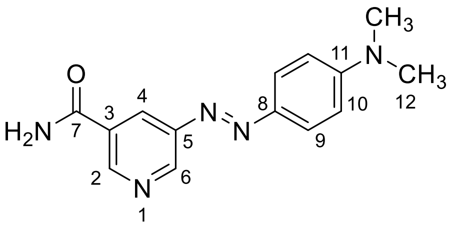

Synthesis was conducted according to the general procedure for the synthesis of nicotinamides from methyl nicotinates using **6e** (200 mg, 0.70 mmol, 1.00 equiv.). The product was obtained as orange solid (169 mg, 0.63 mmol, 90 %): R_f = 0.30 (DCM/MeOH 95:5); mp: 277.1 °C; **^{1}H NMR, H,H-COSY (400 MHz, DMSO-*d*$_6$):** δ (ppm) = 9.09 (d, *J* = 2.3 Hz, 1H, 6-H), 9.05 (d, *J* = 2.0 Hz, 1H, 2-H), 8.47 (pseudo-t, *J* = 2.2 Hz, 1H, 4-H), 8.30 (s, br, 1H, *N*-H), 7.71 (s, br, 1H, *N*-H), 7.86 (m, 2H, 9-H), 6.87 (m, 2H, 10-H), 3.09 (s, 6H, 12-H); **^{13}C NMR, DEPT135, HSQC, HMBC (101 MHz, DMSO-*d*$_6$):** δ (ppm) = 166.1 (7), 153.1 (11), 148.8 (2), 147.6 (6), 147.5 (5), 142.7 (11), 130.4 (3), 125.3 (9), 125.0 (4), 111.6 (10), 39.8 (12); **IR (ATR):** *v* (cm^{-1}) = 3162, 1671, 1598, 1515, 1360, 1140, 942, 910, 815, 694, 642, 627; **ESI-HRMS:** calcd. for $[C_{14}H_{15}N_5O+H]^+$ 169.1277, found 269.1267; **Comp. Purity (220 nm):** 100 %.

(*E*)-5-[(4-amino-3,5-dimethylphenyl)diazenyl]nicotinamide (**7f**)

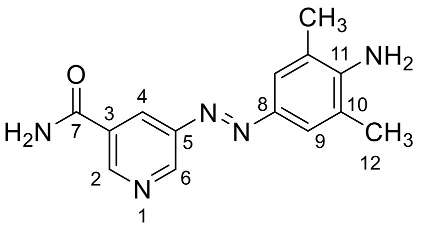

Synthesis was conducted according to the general procedure for the synthesis of nicotinamides from methyl nicotinates using **6f** (105 mg, 0.37 mmol, 1.00 equiv.). The product was obtained as orange solid (95 mg, 0.35 mmol, 95 %): R_f = 0.58 (EtOAC/MeOH 95:5); mp: 255.0 °C; **^{1}H NMR, H,H-COSY (400 MHz, DMSO-*d*$_6$):** δ (ppm) = 9.08 (d, *J* = 1.6 Hz, 1H, 6-H), 9.05 (s, 1H, 2-H), 8.46 (s, 1H, 4-H), 8.32 (s, br, 1H, *N*-H), 7.71 (s, br, 1H, *N*-H), 7.57 (s, 2H, 9-H), 5.80 (s, br, 2H, 9-*N*-H), 2.21 (s, 6H, 12-H); **^{13}C NMR, DEPT135, HSQC, HMBC (101 MHz, DMSO-*d*$_6$):** δ (ppm) = 166.0 (7), 149.9 (11), 148.5 (2), 147.5 (5), 147.3 (6), 142.7 (8), 130.4 (3), 124.9 (4), 124.2 (9), 120.6 (10), 17.5 (12); **IR (ATR):** *v* (cm^{-1}) = 3320, 1666, 1626, 1586, 1476, 1391, 1117, 1024, 892, 846, 695; **ESI-HRMS:** calcd. for $[C_{14}H_{15}N_5O+H]^+$ 269.1277, found 129.1281; **Comp. Purity (220 nm):** 100 %.

Methyl (*E*)-5-[(4-acetamido-3,5-dimethylphenyl)diazenyl]nicotinate (**8a**)

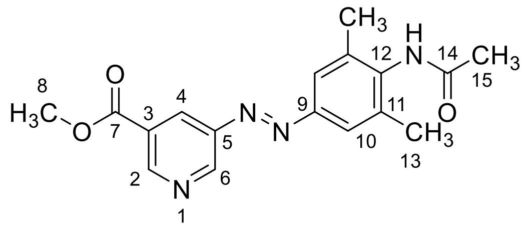

Synthesis was conducted according to the general procedure for amide formation with acyl chlorides using **6f** (200 mg, 0.70 mmol, 1.0 equiv.), acetyl chloride (75 µL, 1.05 mmol, 1.5 equiv.) and DIPEA (239 µL, 1.41 mmol, 2.0 equiv.) in THF_{abs} (20 ml). The crude product was purified by silica gel column chromatography (DCM/MeOH 95:5), which yielded **8a** as orange solid (75 mg, 0.23 mmol, 33 %): R_f = 0.60 (DCM/MeOH 95:5); mp: 260.1 °C; **^{1}H NMR, H,H-COSY (400 MHz, DMSO-*d*$_6$):** δ (ppm) = 9.49 (s, 1H, 12-*N*-H), 9.36 (d, *J* = 2.3 Hz, 1H, 6-H), 9.22 (d, *J* = 2.0 Hz, 1H, 2-H), 8.52 (m, 1H, 4-H), 7.72 (s, 2H, 10-H), 7.56

(s, 2H, 10-H), 3.95 (s, 3H, 8-H), 2.27 (s, 6H, 13-H), 2.10 (s, 3H, 15-H); **^{13}C NMR, DEPT135, HSQC, HMBC (101 MHz, DMSO-*d$_6$*):** δ (ppm) = 167.8 (14), 164.6 (7), 151.5 (2), 150.3 (6), 149.6 (9), 146.9 (5), 139.6 (12), 136.5 (11), 126.4 (3), 126.2 (4), 122.2 (10), 52.7 (8), 22.6 (15), 18.2 (13); **IR (ATR):** *v* (cm^{-1}) = 3236, 1731, 1652, 1523, 1304, 1273, 1151, 1116, 767, 696, 581.

Methyl (*E*)-5-[(4-butyramido-3,5-dimethylphenyl)diazenyl]nicotinate (**8b**)

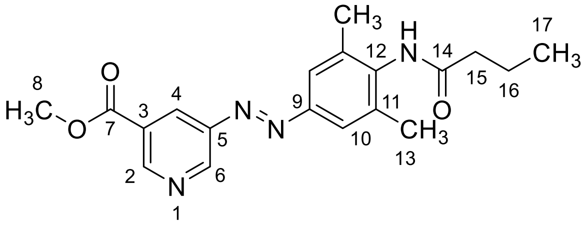

Synthesis was conducted according to the general procedure for amide formation with acyl chlorides using **6f** (300 mg, 1.06 mmol, 1.0 equiv.), butanoyl chloride (132 µL, 1.27 mmol, 1.2 equiv.) and DIPEA (361 µL, 2.12 mmol, 2.0 equiv.) in THF_{abs} (10 ml). The crude product was purified by silica gel column chromatography (DCM/MeOH 95:5) and successive recrystallization (*n*-hexane/acetone). The title compound was obtained as salmon-coloured solid (126 mg, 0.36 mmol, 34 %): R_f = 0.38 (DCM/MeOH 98:2); mp: 183.3 °C; **^{1}H NMR, H,H-COSY (400 MHz, DMSO-*d$_6$*):** δ (ppm) = 9.45 (s, br, 1H, 12-*N*-H), 9.36 (d, *J* = 2.3 Hz, 1H, 6-H), 9.22 (d, *J* = 2.0 Hz, 1H, 2-H), 8.52 (pseudo-t, *J* = 2.2 Hz, 1H, 4-H), 7.73 (s, 2H, 10-H), 3.95 (s, 3H, 8-H), 2.36 (t, *J* = 7.2 Hz, 2H, 15-H), 2.27 (s, 6H, 13-H), 1.67 (h, *J* = 7.3 Hz, 2H, 16-H), 0.98 (t, *J* = 7.4 Hz, 3H, 17-H); **^{13}C NMR, DEPT135, HSQC, HMBC (101 MHz, DMSO-*d$_6$*):** δ (ppm) = 170.8 (14), 164.7 (7), 151.6 (2), 150.4 (6), 149.7 (9), 147.0 (5), 139.7 (12), 136.6 (11), 126.5 (3), 126.3 (4), 122.3 (10), 52.8 (8), 37.4 (15), 18.8 (16), 18.3 (13), 13.7 (17); **IR (ATR):** *v* (cm^{-1}) = 3222, 2959, 1722, 1649, 1532, 1280, 1183, 1097, 790, 746, 580.

Methyl (*E*)-5-[(4-hexanamido-3,5-dimethylphenyl)diazenyl]nicotinate (**8c**)

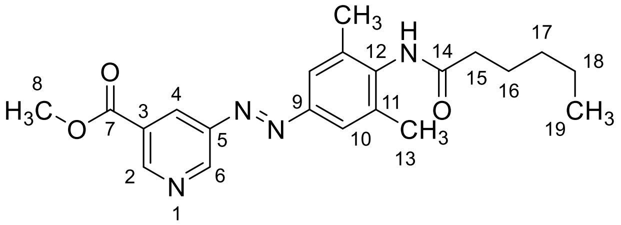

Synthesis was conducted according to the general procedure for amide formation with acyl chlorides using **6f** (300 mg, 1.06 mmol, 1.0 equiv.), hexanoyl chloride (176 µL, 1.27 mmol, 1.2 equiv.) and DIPEA (361 µL, 2.12 mmol, 2.0 equiv.) in THF_{abs} (10 ml). The crude product was purified by silica gel column chromatography (DCM/MeOH 98:2) and successive recrystallization (*n*-hexane/acetone). The title compound was obtained as orange solid (210 mg, 0.55 mmol, 52 %): R_f = 0.22 (DCM/MeOH 98:2); mp: 163.4 °C; **^{1}H NMR, H,H-COSY (400 MHz, DMSO-*d$_6$*):** δ (ppm) = 9.45 (s, 1H, 12-*N*-H), 9.35 (d, *J* = 2.3 Hz, 1H, 6-H), 9.21 (d, *J* = 1.9 Hz, 1H, 2-H), 8.52 (pseudo-t, *J* = 2.1 Hz, 1H, 4-H), 7.72 (s, 2H, 10-H), 3.95 (s, 3H, 8-H), 2.37 (t, *J* = 7.4 Hz, 2H, 15-H), 2.23 (s, 6H, 13-H), 1.65 (p, *J* = 7.2 Hz, 2H, 16-H), 1.38–1.31 (m, 4H, 17-H, 18-H), 0.90 (t, *J* = 7.0 Hz, 3H, 19-H); **^{13}C NMR, DEPT135, HSQC, HMBC (101 MHz, DMSO-*d$_6$*):** δ (ppm) = 170.9 (14),

164.7 (7), 151.6 (2), 150.4 (6), 149.7 (9), 147.0 (5), 139.7 (12), 136.6 (11), 126.5 (3), 126.3 (4), 122.3 (10), 52.8 (8), 35.4 (15), 30.9 (17), 25.0 (16), 21.9 (18), 18.3 (13), 13.9 (19); **IR (ATR):** *v* (cm^{-1}) = 3234, 2927, 1727, 1642, 1513, 1279, 1157, 1118, 739, 639, 588.

Methyl (*E*)-5-[(3,5-dimethyl-4-octanamidophenyl)diazenyl]nicotinate (**8d**)

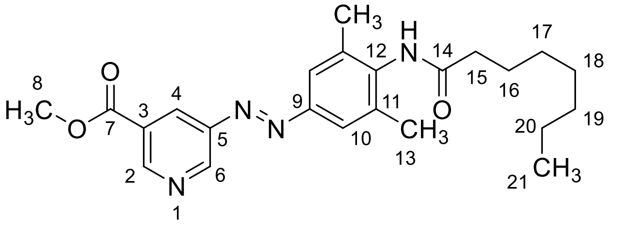

Synthesis was conducted according to the general procedure for amide formation with acyl chlorides using **6f** (300 mg, 1.06 mmol, 1.0 equiv.), octanoyl chloride (220 µL, 1.27 mmol, 1.2 equiv.) and DIPEA (361 µL, 2.12 mmol, 2.0 equiv.) in THF_{abs} (10 ml). The crude product was purified by silica gel column chromatography (DCM/MeOH 98:2) and successive recrystallization (*n*-hexane/acetone). The title compound was obtained as orange solid (128 mg, 0.31 mmol, 29 %): R_f = 0.22 (DCM/MeOH 98:2); mp: 162.1 °C; **^{1}H NMR, H,H-COSY (400 MHz, DMSO-*d$_6$*):** δ (ppm) = 9.45 (s, 1H, 12-*N*-H), 9.35 (d, *J* = 2.3 Hz, 1H, 6-H), 9.22 (d, *J* = 1.9 Hz, 1H, 2-H), 8.52 (pseudo-t, *J* = 2.2 Hz, 1H, 4-H), 7.72 (s, 2H, 10-H), 3.95 (s, 3H, 8-H), 2.37 (t, *J* = 7.3 Hz, 2H, 15-H), 2.26 (s, 6H, 13-H), 1.64 (p, *J* = 7.0 Hz, 2H, 16-H), 1.40–1.22 (m, 8H, 17–20-H), 0.88 (t, *J* = 6.9 Hz, 3H, 21-H); **^{13}C NMR, DEPT135, HSQC, HMBC (101 MHz, DMSO-*d$_6$*):** δ (ppm) = 170.9 (14), 164.7 (7), 151.6 (2), 150.4 (6), 149.7 (9), 147.0 (5), 139.7 (12), 136.6 (11), 126.5 (3), 126.3 (4), 122.3 (10), 52.8 (8), 35.4 (15), 31.2 (17), 28.7 (18), 28.4 (19), 25.4 (16), 22.1 (20), 18.3 (13), 13.9 (21); **IR (ATR):** *v* (cm^{-1}) = 3230, 2922, 1728, 1640, 1514, 1279, 1203, 1183, 766, 698, 589.

Methyl (*E*)-5-[(3,5-dimethyl-4-decanamidophenyl)diazenyl]nicotinate (**8e**)

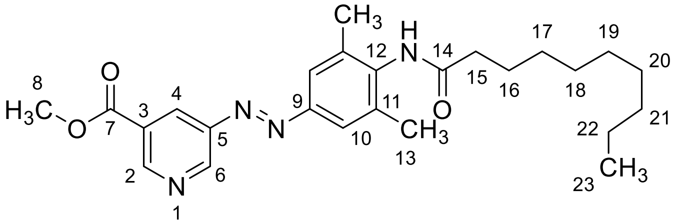

Synthesis was conducted according to the general procedure for amide formation with acyl chlorides using **6f** (100 mg, 0.35 mmol, 1.0 equiv.), decanoyl chloride (110 µL, 0.53 mmol, 1.5 equiv.) and DIPEA (119 µL, 0.70 mmol, 2.0 equiv.) in THF_{abs} (10 ml). The crude product was purified by silica gel column chromatography (*n*-hexane/THF 2:1) and successive recrystallization (n-hexane/acetone). The title compound was obtained as orange solid (40 mg, 0.09 mmol, 26 %): R_f = 0.38 (*n*-hexane/THF 2:1); mp: 156.7 °C (*n*-hexane/acetone); **^{1}H NMR, H,H-COSY (400 MHz, DMSO-*d$_6$*):** δ (ppm) = 9.45 (s, 1H, 12-*N*-H), 9.35 (d, *J* = 2.0 Hz, 1H, 6-H), 9.22 (d, *J* = 1.6 Hz, 1H, 2-H), 8.52 (pseudo-t, *J* = 2.0 Hz, 1H, 4-H), 7.72 (s, 2H, 10-H), 3.95 (s, 3H, 8-H), 2.37 (t, *J* = 7.3 Hz, 2H, 15-H), 2.26 (s, 6H, 13-H), 1.70–1.59 (m, 2H, 16-H), 1.42–1.19 (m, 12H, 17–22-H), 0.87 (t, *J* = 6.7 Hz, 3H, 23-H); **^{13}C NMR, DEPT135, HSQC, HMBC (101 MHz, DMSO-*d$_6$*):** δ (ppm) = 170.9 (14), 164.7 (7), 151.6 (2), 150.4 (6), 149.7 (9), 147.0

(5), 139.7 (12), 136.6 (11), 126.5 (3), 126.3 (4), 122.3 (10), 52.8 (8), 35.4 (15), 31.3 (21), 29.0–28.6 (17–20), 25.4 (16), 22.1 (22), 18.3 (13), 14.0 (23); **IR (ATR):** *v* (cm^{-1}) = 3235, 2921, 1729, 1640, 1514, 1280, 1203, 1184, 766, 698, 588.

Methyl (*E*)-5-[(4-dodecanamido-3,5-dimethylphenyl)diazenyl]nicotinate (**8f**)

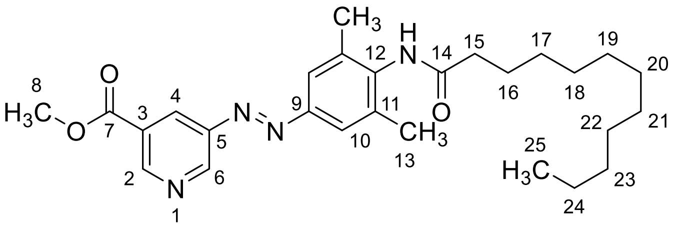

Synthesis was conducted according to the general procedure for amide formation with acyl chlorides using **6f** (300 mg, 1.06 mmol, 1.0 equiv.), dodecanoyl chloride (302 µL, 1.27 mmol, 1.2 equiv.) and DIPEA (361 µL, 2.12 mmol, 2.0 equiv.) in THF_{abs} (10 ml). The crude product was purified by silica gel column chromatography (DCM/MeOH 98:2) and successive recrystallization from (*n*-hexane/acetone). The title compound was obtained as orange solid (184 mg, 0.39 mmol, 37 %): R_f = 0.24 (DCM/MeOH 98:2); mp: 158.4 °C; **^{1}H NMR, H,H-COSY (400 MHz, DMSO-*d₆*):** δ (ppm) = 9.29 (s, 1H, 6-H), 9.28 (s, 1H, 2-H), 8.68–8.65 (m, 1H, 4-H), 7.69 (s, 2H, 10-H), 4.01 (s, 3H, 8-H), 2.45 (t, *J* = 7.6 Hz, 2H, 15-H), 2.33 (s, 6H, 13-H), 1.84–1.73 (m, 2H, 16-H), 1.48–1.17 (m, 16H, 17–24-H), 0.89 (t, *J* = 6.8 Hz, 3H, 25-H); **^{13}C NMR, DEPT135, HSQC, HMBC (101 MHz, DMSO-d_6):** δ (ppm) = 171.5 (14), 165.3 (7), 151.8 (2), 150.7 (9), 150.1 (6), 137.8 (12), 136.5 (11), 128.2 (4), 126.8 (3), 123.1 (10), 52.7 (8), 36.9 (15), 31.9 (23), 29.7–29.3 (17–22), 26.1 (16), 22.7 (24), 18.8 (13), 14.1 (25); **IR (ATR):** *v* (cm^{-1}) = 3240, 2954, 1729, 1640, 1513, 1280, 1204, 1185, 766, 698, 588.

Ethyl (*E*)-5-[(4-amino-3,5-dimethylphenyl)diazenyl]nicotinate (**11**)

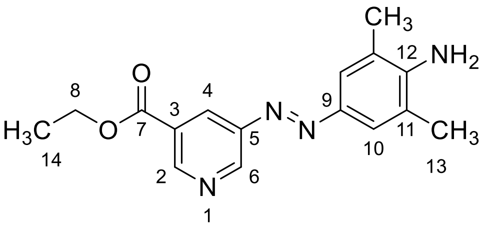

Synthesis was conducted according to the general procedure for azo coupling reactions using **10** (1.33 g, 8.00 mmol, 1.0 equiv.) and 2,6-dimethylaniline (1.07 g, 8.8 mmol, 1.1 equiv.). The product was purified by silica gel chromatography (DCM/MeOH 98:2) and successive recrystallization (cyclohexane/EtOAc 2:1). The product was obtained as red crystals (905 mg, 3.03 mmol, 38 %): R_f = 0.40 (DCM/MeOH 98:2); mp: 152.5 °C (cyclohexane/EtOAc); **^{1}H NMR, H,H-COSY (400 MHz, DMSO-d_6):** δ (ppm) = 9.16 (d, *J* = 2.3 Hz, 1H, 6-H), 9.06 (d, *J* = 2.0 Hz, 1H, 2-H), 8.40 (pseudo-t, *J* = 2.2 Hz, 1H, 4-H), 7.57 (s, 2H, 10-H), 5.85 (s, 1H, 12-*N*-H), 4.39 (q, *J* = 7.1 Hz, 2H, 8-H), 2.19 (s, 6H, 13-H), 1.37 (t, *J* = 7.1 Hz, 3H, 14-H); **^{13}C NMR, DEPT135, HSQC, HMBC (101 MHz, DMSO-d_6):** δ (ppm) = 164.4 (7), 150.3 (9), 149.4 (2, 6), 147.6 (5), 142.6 (12), 126.4 (3), 125.5 (4), 124.4 (10), 120.6 (11), 61.4 (8),17.7 (13) 14.0 (14); **IR (ATR):** *v* (cm^{-1}) = 3462, 3339, 3220, 1726, 1655, 1598, 1374, 1260, 1187, 1020, 763; **ESI-HRMS:** calcd. for $[C_{16}H_{18}N_4O_2+H]^+$ 298.1430, found 298.1443; **Comp. Purity (220 nm):** 100 %.

Ethyl (*E*)-5-[(3,5-dimethyl-4-tetradecanamidophenyl)diazenyl]nicotinate (**8g**)

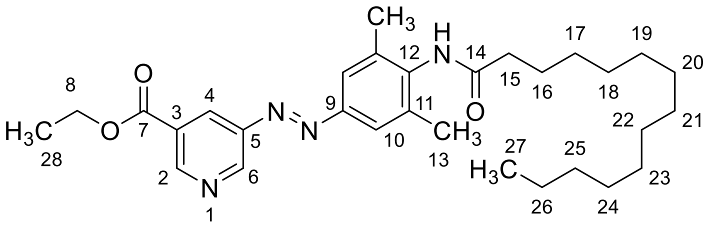

Synthesis was conducted according to the general procedure for amide formation with acyl chlorides using **11** (300 mg, 1.01 mmol, 1.0 equiv.), myristoyl chloride (408 µL, 1.50 mmol, 1.5 equiv.) and DIPEA (340 µL, 2.00 mmol, 2.0 equiv.) in THF_{abs} (10 ml). The crude product was purified by silica gel column chromatography (cyclohexane/THF 2:1) and successive recrystallization (cyclohexane/acetone). The pure product was yielded as salmon-coloured solid (390 mg, 0.77 mmol, 76 %): R_f = 0.63 (cyclohexane/THF 2:1); mp: 123.3 (cyclohexane/acetone); **^{1}H NMR (400 MHz, acetone-*d₆*):** δ (ppm) = 9.16 (s, 1H, 6-H), 9.10 (s, 1H, 2-H), 8.56 (s, 1H, 12-*N*-H), 8.47 (s, 1H, 4-H), 7.59 (s, 2H, 10-H), 4.33 (q, *J* = 7.0 Hz, 2H, 8-H), 2.35 (t, *J* = 7.3 Hz, 2H, 15-H), 2.22 (s, 6H, 13-H), 1.68–1.57 (m, 2H, 16-H), 1.30 (t, *J* = 7.0 Hz, 3H, 28-H), 1.26–1.06 (m, 24H, 17-H – 26-H); **IR (ATR):** *v* (cm^{-1}) = 3224, 2917, 2850, 1726, 1643, 1527, 1280, 1189, 1095, 1024, 766.

Methyl (*E*)-5-[(4-benzamido-3,5-dimethylphenyl)diazenyl]nicotinate (**8h**)

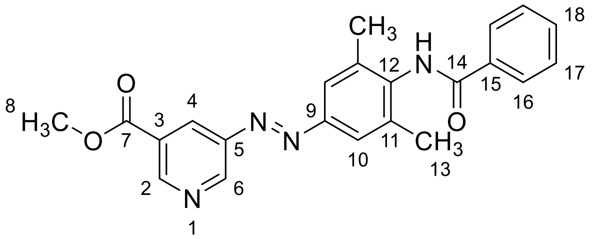

A ice-cooled solution of **6f** (300 mg, 1.06 mmol, 1.0 equiv.) in dry THF (20 mL) was treated successively with DIPEA (361 µL, 2.12 mmol, 2.0 equiv.) and benzoyl chloride (183 µL, 1.59 mmol, 1.5 equiv.) and stirred for 2 hours at room temperature. The reaction mixture was concentrated under reduced pressure and taken up in EtOAc (50 mL). It was washed successively with aq. HCl-solution (1M, 3 × 20 mL), water (3 × 20 mL) and sat. aq. NaCl-solution (2 × 20 mL) and dried over $MgSO_4$. After filtration, the solvent was removed under reduced pressure and the residue purified by silica gel column chromatography (DCM/MeOH 98:2). The title compound was obtained as orange solid (236 mg, 0.61 mmol, 58 %): R_f = 0.18 (DCM/MeOH 99:1); mp: 255.0 °C (decomp.); **^{1}H NMR (400 MHz, DMSO-*d₆*):** δ (ppm) = 10.0 (s, 1H, 12-*N*-H), 9.37 (d, *J* = 2.3 Hz, 1H, 6-H), 9.23 (d, *J* = 1.9 Hz, 1H, 2-H), 8.54 (pseudo-t, *J* = 2.1 Hz, 1H, 4-H), 8.05 (pseudo-d, J = 7.1 Hz, 2H, 16-H), 7.80 (s, 2H, 10-H), 7.63 (t, *J* = 7.3 Hz, 1H, 18-H), 7.56 (pseudo-t, J = 7.3 Hz, 2H, 17-H), 3.96 (s, 3H, 8-H), 2.33 (s, 6H, 13-H); **^{13}C NMR, DEPT135, HSQC, HMBC (101 MHz, DMSO-*d₆*):** δ (ppm) = 165.1 (14), 164.7 (7), 151.7 (2), 150.4 (6), 150.0 (12), 147.0 (5), 139.6 (9), 137.2 (11), 134.0 (15), 131.7 (18), 128.5 (17), 127.6 (16), 126.5 (3), 126.4 (4), 122.4 (10), 52.8 (8), 18.2 (13); **IR (ATR):** *v* (cm^{-1}) = 3241, 1721, 1635, 1600, 1579, 1511, 1311, 1281, 1206, 1183, 768.

Methyl (*E*)-5-{[4-(4-fluorobenzamido)-3,5-dimethylphenyl]diazenyl}nicotinate (**8i**)

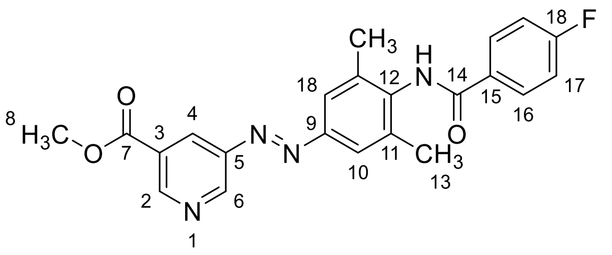

An ice-cooled solution of **6f** (300 mg, 1.06 mmol, 1.0 equiv.) in dry THF (20 mL) was treated successively with DIPEA (361 µL, 2.12 mmol, 2.0 equiv.) and 4-fluorobenzoyl chloride (188 µL, 1.59 mmol, 1.5 equiv.) and stirred under an argon atmosphere for 2 days at room temperature. The reaction mixture was concentrated under reduced pressure and taken up in EtOAc (100 mL). It was washed successively with aq. HCl-solution (1M, 3 × 20 mL), water (3 × 20 mL) and sat. aq. NaCl-solution (2 × 20 mL) and dried over $MgSO_4$. After filtration, the solvent was removed under reduced pressure and the residue purified by silica gel column chromatography (*n*-hexane/THF 2:1). The title compound was obtained as orange solid (171 mg, 0.42 mmol, 40 %): R_f = 0.34 (*n*-hexane/THF 2:1); mp: 210.7 °C (decomp.); **^{1}H NMR (400 MHz, DMSO-*d*$_6$):** δ (ppm) = 10.0 (s, 1H, 12-*N*-H), 9.38 (d, *J* = 2.2 Hz, 1H, 6-H), 9.23 (d, *J* = 1.9 Hz, 1H, 2-H), 8.54 (pseudo-t, *J* = 2.2 Hz, 1H, 4-H), 8.14–8.07 (m, 2H, 16-H), 7.80 (s, 2H, 10-H), 7.45–7.36 (m, 2H, 17-H), 3.96 (s, 3H, 8-H), 2.32 (s, 6H, 13-H); **^{13}C NMR, DEPT135, HSQC, HMBC (101 MHz, DMSO-*d*$_6$):** δ (ppm) = 164.6 (7), 164.1 (d, *J* = 249.3 Hz, 18), 163.9 (14), 151.6 (2), 150.4 (6), 149.9 (12), 146.9 (5), 139.4 (9), 137.1 (11), 130.4 (d, *J* = 2.8 Hz, 15), 130.2 (d, *J* = 9.1 Hz, 16), 126.5 (3), 126.3 (4), 122.3 (10), 115.4 (d, *J* = 21.8 Hz, 17), 52.7 (8), 13.1 (13); **IR (ATR):** *v* (cm^{-1}) = 3237, 1730, 1645, 1603, 1520, 1500, 1271, 1155, 846, 763, 697.

Methyl (*E*)-5-({3,5-dimethyl-4-[4-(thiophen-2-yl)benzamido]phenyl}diazenyl)nicotinate (**8j**)

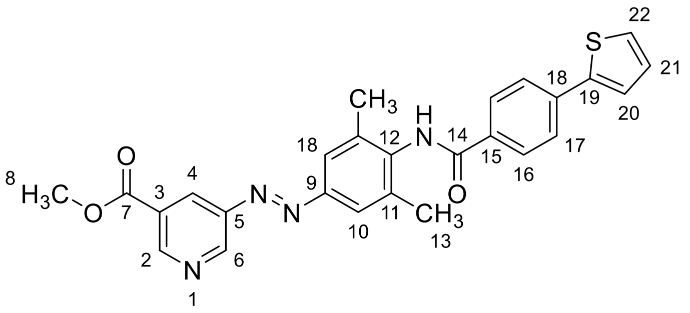

Under an argon atmosphere 4-(thiophen-2-yl)benzoic acid (431 mg, 2.11 mmol, 2.0 equiv.) was dissolved in dry DCM (10 mL). Oxalyl chloride (272 µL, 3.17 mmol, 3.0 equiv.) and dry DMF (5 µL) were added and the reaction mixture was stirred at room temperature until no further evolution of gas was observable (4 hours). DCM and excessive oxalyl chloride were evaporated under reduced pressure and the residue taken up in dry THF (10 mL). This solution was added slowly to an ice-cooled solution of **6f** (300 mg, 1.06 mmol, 1.0 equiv.) and pyridine (852 µL, 10.55 mmol, 10.0 equiv.) in dry THF (10 mL). After stirring for 3 days at room temperature the reaction mixture was concentrated under reduced pressure and taken up in EtOAc (50 mL). It was washed successively with sat. aq. NH_4Cl-solution

(30 mL), aq. NaOH-solution (1M, 3 × 30 mL), water (3 × 30 mL) and sat. aq. NaCl-solution (30 mL) and dried over $MgSO_4$. After filtration, the solvent was evaporated under reduced pressure and the crude product purified by silica gel column chromatography (DCM/MeOH 99:1). The title compound was obtained as orange solid (150 mg, 0.32 mmol, 30 %): R_f = 0.41 (DCM/MeOH 99:1); mp: 238.3 °C (decomp.); **^{1}H NMR (400 MHz, acetone-*d*$_6$):** δ (ppm) = 10.0 (s, 1H, 12-*N*-H), 9.38 (d, *J* = 2.2 Hz, 1H, 6-H), 9.23 (d, *J* = 1.8 Hz, 1H, 2-H), 8.54 (pseudo-t, *J* = 2.0 Hz, 1H, 4-H), 8.09 (d, *J* = 8.2 Hz, 2H, 16-H), 7.85 (d, *J* = 8.3 Hz, 2H, 17-H), 7.80 (s, 2H, 10-H), 7.69 (d, *J* = 3.0 Hz, 1H, 20-H), 7.66 (d, *J* = 5.0 Hz, 1H, 22-H), 7.21 (dd, *J* = 4.8, 3.8 Hz, 1H, 21-H), 3.96 (s, 3H, 8-H), 2.35 (s, 6H, 13-H); **^{13}C NMR, DEPT135, HSQC, HMBC (101 MHz, DMSO-*d*$_6$):** δ (ppm) = 164.6 (7), 164.4 (14), 151.6 (2), 150.4 (6), 149.9 (12), 146.9 (5), 142.1 (19), 139.5 (9), 137.1 (11), 136.7 (18), 132.5 (15), 128.7 (21), 128.5 (16), 126.9 (22), 126.4 (3), 126.3 (4), 125.2 (17), 125.0 (20), 122.3 (10), 52.7 (8), 18.2 (13); **IR (ATR):** *v* (cm^{-1}) = 3261, 1732, 1645, 1606, 1510, 1478, 1429, 1271, 1176, 851, 764.

Methyl (*E*)-5-{[4-(adamantane-1-carboxamido)-3,5-dimethylphenyl]diazenyl}nicotinate (**8k**)

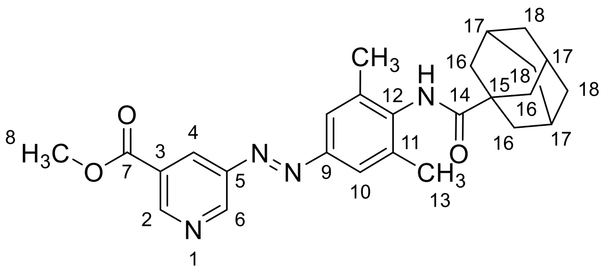

Adamantane-1-carboxylic acid (270 mg, 1.50 mmol, 1.0 equiv.) was treated with thionyl chloride (544 µL, .7.50 mmol, 5.0 equiv.) and refluxed for 2 hours under anhydrous conditions. Excessive thionyl chloride was removed under reduced pressure and the residue dissolved in dry THF (3 mL). This solution was added to an ice-cooled solution of **6f** (426 mg, 1.50 mmol, 1.0 equiv.) and pyridine (1.21 mL, 15 mmol, 10 equiv.) in dry THF (10 mL). After stirring for 3 days at room temperature, the reaction mixture was concentrated under reduced pressure and taken up in EtOAc (50 mL). It was washed successively with sat. aq. $NaHCO_3$-solution (3 × 30 mL), water (3 × 30 mL) and aq. sat. NaCl-solution (30 mL) and dried over $MgSO_4$. After filtration, the solvent was removed under reduced pressure and the residue purified by silica gel column chromatography (DCM/MeOH 98:2) and successive recrystallization (acetone). The title compound was yielded as orange solid (260 mg, 0.58 mmol, 39 %): R_f = 0.40 (DCM/MeOH 99:1); mp: 230.4 °C (decomp.); **^{1}H NMR, H,H-COSY (400 MHz, $CDCl_3$):** δ (ppm) = 9.28 (s, br, 2H, 2-H, 6-H), 8.72 (s, br, 1H, 4-H), 7.68 (s, 2H, 10-H), 7.12 (s, br, 12-*N*-H), 4.01 (s, 3H, 8-H), 2.29 (s, 6H, 13-H), 2.15–2.10 (m, 3H, 17-H), 2.09–2.00 (m, 6H, 16-H), 1.85–1.72 (m, 6H, 18-H); **^{13}C NMR, DEPT135, HSQC, HMBC (101 MHz, $CDCl_3$):** δ (ppm) = 176.1 (14), 164.3 (7), 150.4 (12), 149.2 (2), 148.1 (5), 147.2 (6), 138.9 (9), 136.8 (11), 130.4 (4), 127.8 (3), 123.2 (10), 53.1 (8), 41.5

(15), 39.6 (16), 36.5 (18), 28.2 (17), 18.6 (13); **IR (ATR):** *v* (cm^{-1}) = 3368, 2912, 2889, 2850, 1721, 1639, 1504, 1473, 1309, 1281, 1179.

(*E*)-5-[(4-acetamido-3,5-dimethylphenyl)diazenyl]nicotinamide (**9a**)

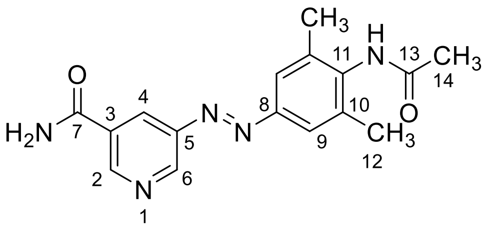

Synthesis was conducted according to the general procedure for the synthesis of nicotinamides from methyl nicotinates using **8a** (58 mg, 0.19 mmol, 1.00 equiv.). The product was obtained as orange solid (58 mg, 0.19 mmol, 100 %): R_f = 0.28 (EtOAC/MeOH 95:5); mp: 285.6 °C; **^{1}H NMR, H,H-COSY (400 MHz, DMSO-*d*$_6$):** δ (ppm) = 9.49 (s, 1H, 11-*N*-H), 9.23 (d, *J* = 2.3 Hz, 1H, 6-H), 9.17 (d, *J* = 2.0 Hz, 1H, 2-H), 8.56 (m, 1H, 4-H), 8.37 (s, br, 1H, 7-*N*-H), 7.77 (s, br, 1H, *N*-H), 7.71 (s, 2H, 9-H), 2.27 (s, 6H, 12-H), 2.10 (s, 3H, 14-H); **^{13}C NMR, DEPT135, HSQC, HMBC (101 MHz, DMSO-*d*$_6$):** δ (ppm) = 167.9 (13), 165.7 (7), 150.6 (2), 149.7 (8), 148.2 (6), 147.0 (5), 139.5 (11), 136.5 (10), 130.6 (3), 125.6 (4), 122.1 (9), 22.6 (14), 18.3 (12); **IR (ATR):** *v* (cm^{-1}) = 3226, 3132, 1699, 1647, 1525, 1394, 1368, 1136, 1024, 885, 696; **ESI-HRMS:** calcd. for $[C_{16}H_{17}N_5O_2+H]^+$ 311.1382, found 311.1376; **Comp. Purity (220 nm):** 100 %.

(*E*)-5-[(4-butyramido-3,5-dimethylphenyl)diazenyl]nicotinamide (**9b**)

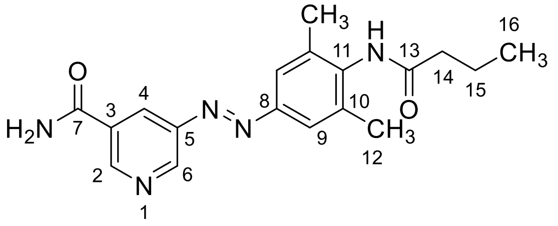

Synthesis was conducted according to the general procedure for the synthesis of nicotinamides from methyl nicotinates using **8b** (100 mg, 0.28 mmol, 1.00 equiv.). The product was obtained as orange solid (95 mg, 0.28 mmol, 100 %): R_f = 0.45 (n-hexane/acetone 1:2); mp: 260.0 °C; **^{1}H NMR, H,H-COSY (400 MHz, DMSO-*d*$_6$):** δ (ppm) = 9.45 (s, 1H, 11-*N*-H), 9.23 (d, *J* = 2.3 Hz, 1H, 6-H), 9.17 (d, *J* = 2.0 Hz, 1H, 2-H), 8.56 (pseudo-t, *J* = 2.2 Hz, 1H, 4-H), 8.37 (s, br, 1H, *N*-H), 7.77 (s, br, 1H, 7-*N*-H), 7.71 (s, 2H, 9-H), 2.36 (t, *J* = 7.2 Hz, 2H, 14-H), 2.27 (s, 6H, 12-H), 1.67 (h, *J* = 7.3 Hz, 2H, 15-H), 0.98 (t, *J* = 7.4 Hz, 3H, 16-H); **^{13}C NMR, DEPT135, HSQC, HMBC (101 MHz, DMSO-*d*$_6$):** δ (ppm) = 170.7 (13), 165.6 (7), 150.5 (2), 149.7 (8), 148.2 (6), 146.9 (5), 139.4 (11), 136.5 (10), 130.5 (3), 125.5 (4), 122.1 (9), 3733 (14), 18.7 (15), 18.3 (12), 13.6 (16); **IR (ATR):** *v* (cm^{-1}) = 3235, 1704, 1648, 1520, 1395, 1294, 1126, 1025, 914, 884, 695; **ESI-HRMS:** calcd. for $[C_{18}H_{21}N_5O_2+H]^+$ 339.1695, found 339.1712; **Comp. Purity (254 nm):** 100 % (*E*+*Z*)-**7b**.

(*E*)-5-[(4-hexanamido-3,5-dimethylphenyl)diazenyl]nicotinamide (**9c**)

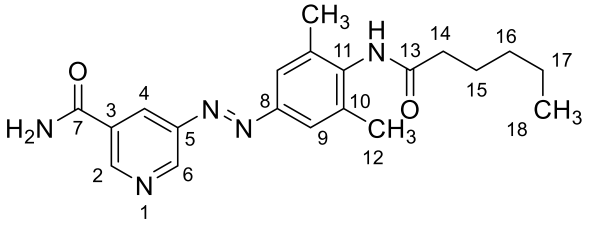

Synthesis was conducted according to the general procedure for the synthesis of nicotinamides from methyl nicotinates using **8c** (150 mg, 0.39 mmol, 1.00 equiv.). The product was obtained as yellow-orange solid (137 mg, 0.37 mmol, 95 %): R_f = 0.50 (*n*-hexane/acetone 1:2); mp: 263.9 °C; **^{1}H NMR, H,H-COSY (400 MHz, DMSO-*d$_6$*):** δ (ppm) = 9.45 (s, 1H, 11-*N*-H), 9.23 (d, *J* = 2.3 Hz, 1H, 6-H), 9.17 (d, *J* = 2.0 Hz, 1H, 2-H), 8.56 (pseudo-t, *J* = 2.2 Hz, 1H, 4-H), 8.37 (s, br, 1H, 7-*N*-H), 7.77 (s, br, 1H, 7-*N*-H), 7.71 (s, 2H, 9-H), 2.37 (t, *J* = 7.4 Hz, 2H, 14-H), 2.27 (s, 6H, 12-H), 1.65 (p, *J* = 7.3 Hz, 2H, 15-H), 1.40–1.31 (m, 4H, 16-H, 17-H), 0.90 (t, *J* = 7.0 Hz, 3H, 18-H); **^{13}C NMR, DEPT135, HSQC, HMBC (101 MHz, DMSO-*d$_6$*):** δ (ppm) = 170.8 (13), 165.6 (7), 150.5 (2), 149.7 (8), 148.1 (6), 146.9 (5), 139.4 (11), 136.5 (10), 130.5 (3), 125.5 (4), 122.1 (9), 35.3 (14), 30.8 (16), 25.0 (15), 21.8 (17), 18.2 (12), 13.8 (18); **IR (ATR):** *v* (cm^{-1}) = 3237, 3151, 1705, 1650, 1521, 1397, 1296, 1132, 914, 884, 695; **ESI-HRMS:** calcd. for $[C_{20}H_{25}N_5O_2+H]^+$ 367.2008, found 367.2013; **Comp. Purity (254 nm):** 100 % (*E*+*Z*)-**7c**.

(*E*)-5-[(3,5-dimethyl-4-octanamidophenyl)diazenyl]nicotinamide (**9d**)

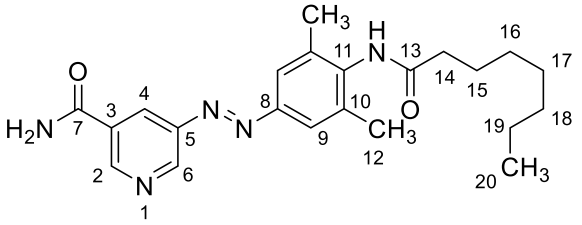

Synthesis was conducted according to the general procedure for the synthesis of nicotinamides from methyl nicotinates using **8d** (92 mg, 0.22 mmol, 1.00 equiv.). The product was obtained as orange solid (71 mg, 0.18 mmol, 82 %): R_f = 0.55 (*n*-hexane/acetone 1:2); mp: 259.2 °C; **^{1}H NMR, H,H-COSY (400 MHz, DMSO-*d$_6$*):** δ (ppm) = 9.45 (s, 1H, 11-*N*-H), 9.23 (d, *J* = 2.3 Hz, 1H, 6-H), 9.17 (d, *J* = 2.0 Hz, 1H, 2-H), 8.56 (pseudo-t, *J* = 2.2 Hz, 1H, 4-H), 8.37 (s, br, 1H, 7-*N*-H), 7.77 (s, br, 1H, 7-*N*-H), 7.71 (s, 2H, 9-H), 2.37 (t, *J* = 7.4 Hz, 2H, 14-H), 2.26 (s, 6H, 12-H), 1.70–1.59 (m, 2H, 15-H), 1.40–1.22 (m, 8H, 16 – 19-H), 0.88 (t, *J* = 6.9 Hz, 3H, 20-H); **^{13}C NMR, DEPT135, HSQC, HMBC (101 MHz, DMSO-*d$_6$*):** δ (ppm) = 170.8 (13), 165.6 (7), 150.5 (2), 149.7 (8), 148.1 (6), 146.9 (5), 139.4 (11), 136.5 (10), 130.5 (3), 125.5 (4), 122.1 (9), 35.3 (14), 31.1 (18), 28.6 (16/17), 28.3 (16/17), 25.3 (15), 22.0 (19), 18.2 (12), 13.9 (20); **IR (ATR):** *v* (cm^{-1}) = 3235, 3152, 1703, 1647, 1517, 1394, 1295, 1125, 914, 884, 694; **ESI-HRMS:** calcd. for $[C_{22}H_{29}N_5O_2+H]^+$ 395.2321, found 395.2319; **Comp. Purity (254 nm):** 100 % (*E*+*Z*)-**7d**.

(*E*)-5-[(4-decanamido-3,5-dimethylphenyl)diazenyl]nicotinamide (**9e**)

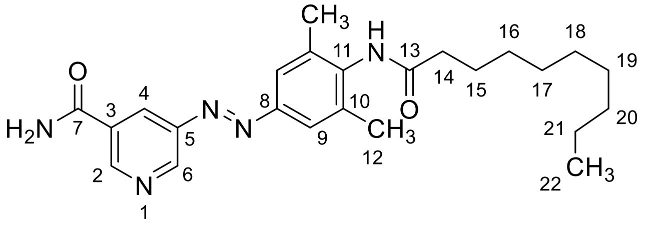

Synthesis was conducted according to the general procedure for the synthesis of nicotinamides from methyl nicotinates using **8e** (30 mg, 0.07 mmol, 1.00 equiv.). The product was obtained as orange solid (28 mg, 0.07 mmol, 100 %): R_f = 0.59 (*n*-hexane/acetone 1:2); mp: 260.0 °C; **^{1}H NMR, H,H-COSY (400 MHz, DMSO-*d₆*):** δ (ppm) = 9.44 (s, 1H, 11-*N*-H), 9.23 (d, *J* = 2.3 Hz, 1H, 6-H), 9.17 (d, *J* = 2.0 Hz, 1H, 2-H), 8.56 (pseudo-t, *J* = 2.2 Hz, 1H, 4-H), 8.37 (s, br, 1H, 7-*N*-H), 7.77 (s, br, 1H, 7-*N*-H), 7.71 (s, 2H, 9-H), 2.36 (t, J = 7.3 Hz, 2H, 14-H), 2.26 (s, 6H, 12-H), 1.68–1.60 (m, 2H, 15-H), 1.40–1.18 (m, 12H, 16 – 21-H), 0.87 (t, *J* = 6.8 Hz, 3H, 22-H); **^{13}C NMR, DEPT135, HSQC, HMBC (101 MHz, DMSO-d_6):** δ (ppm) = 170.8 (13), 165.6 (7), 150.5 (2), 149.7 (8), 148.1 (6), 146.9 (5), 139.4 (11), 136.5 (10), 130.5 (3), 125.5 (4), 122.1 (9), 35.3 (14), 31.2 (20), 29.0–28.3 (16–19), 25.3 (15), 22.0 (21), 18.2 (12), 13.9 (22); **IR (ATR):** *v* (cm^{-1}) = 3222, 2916, 2850, 1705, 1642, 1527, 1395, 1128, 1025, 884, 695; **ESI-HRMS:** calcd. for $[C_{24}H_{33}N_5O_2+H]^+$ 423.2634, found 423.2637; **Comp. Purity (254 nm):** 98.5 % (*E*+*Z*)-**7e**.

(*E*)-5-[(4-dodecanamido-3,5-dimethylphenyl)diazenyl]nicotinamide (**9f**)

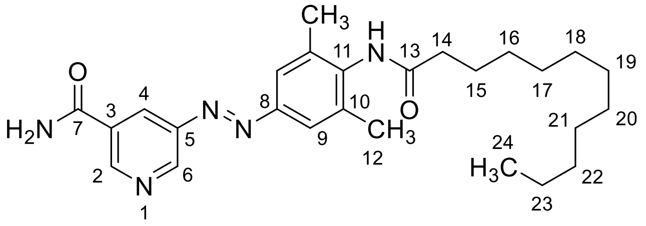

Synthesis was conducted according to the general procedure for the synthesis of nicotinamides from methyl nicotinates using **8f** (133 mg, 0.29 mmol, 1.00 equiv.). The product was obtained as orange solid (130 mg, 0.29 mmol, 100 %): R_f = 0.62 (n-hexane/acetone 1:2); mp: 256.8 °C; **^{1}H NMR, H,H-COSY (400 MHz, DMSO-d_6):** δ (ppm) = 9.44 (s, 1H, 11-*N*-H), 9.22 (d, *J* = 2.3 Hz, 1H, 6-H), 9.17 (d, *J* = 2.0 Hz, 1H, 2-H), 8.56 (pseudo-t, *J* = 2.1 Hz, 1H, 4-H), 8.37 (s, br, 1H, 7-*N*-H), 7.77 (s, br, 1H, 7-*N*-H), 7.70 (s, 2H, 9-H), 2.36 (t, J = 7.3 Hz, 2H, 14-H), 2.26 (s, 6H, 12-H), 1.69–1.58 (m, 2H, 15-H), 1.41–1.17 (m, 16H, 16 – 23-H), 0.86 (t, *J* = 6.8 Hz, 3H, 24-H); **^{13}C NMR, DEPT135, HSQC, HMBC (101 MHz, DMSO-d_6):** δ (ppm) = 170.8 (13), 165.6 (7), 150.5 (2), 149.7 (8), 148.1 (6), 146.9 (5), 139.4 (11), 136.5 (10), 130.5 (3), 125.5 (4), 122.1 (9), 35.3 (14), 31.2 (22), 29.2–28.1 (16–21), 25.3 (15), 22.0 (23), 18.2 (12), 13.9 (24); **IR (ATR):** *v* (cm^{-1}) = 2954, 2850, 1707, 1642, 1529, 1467, 1296, 1153, 1127, 1095, 694; **ESI-HRMS:** calcd. for $[C_{26}H_{37}N_5O_2+H]^+$ 451.2947, found 451.2961; **Comp. Purity (254 nm):** 100 % (*E*+*Z*)-**7f**.

(*E*)-5-[(3,5-dimethyl-4-tetradecanamidophenyl)diazenyl]nicotinamide (**9g**)

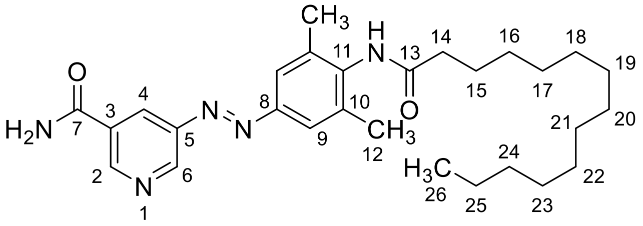

Synthesis was conducted according to the general procedure for the synthesis of nicotinamides from methyl nicotinates using **8g** (100 mg, 0.20 mmol, 1.00 equiv.). The product was obtained as orange solid (95 mg, 0.20 mmol, 100 %): R_f = 0.59 (EtOAC/MeOH 95:5); mp: 254.1 °C; **^{1}H NMR, H,H-COSY (400 MHz, DMSO-*d$_6$*):** δ (ppm) = 9.43 (s, 1H, 11-*N*-H), 9.23 (d, *J* = 2.3 Hz, 1H, 6-H), 9.17 (d, *J* = 2.0 Hz, 1H, 2-H), 8.56 (pseudo-t, *J* = 2.2 Hz, 1H, 4-H), 8.36 (s, br, 1H, *N*-H), 7.77 (s, br, 1H, *N*-H), 7.71 (s, 2H, 9-H), 2.36 (t, *J* = 7.3 Hz, 2H, 15-H), 2.26 (s, 6H, 12-H), 1.70–1.58 (m, 2H, 15-H), 1.11–1.43 (m, 21H, 16-H – 25-H), 0.86 (t, *J* = 6.8 Hz, 3H, 26-H); **^{13}C NMR, DEPT135, HSQC, HMBC (101 MHz, DMSO-*d$_6$*):** δ (ppm) = 170.8 (13), 165.6 (7), 150.5 (2), 149.7 (8), 148.1 (6), 146.9 (5), 139.4 (11), 136.5 (10), 130.6 (3), 125.6 (4), 122.1 (9), 35.3 (14), 28.1–31.2 (16–25), 25.3 (15), 18.2 (12), 13.9 (26); **IR (ATR):** *v* (cm^{-1}) = 2915, 2849, 1701, 1638, 1527, 1468, 1392, 1127, 1094, 885, 696; **ESI-HRMS:** calcd. for $[C_{28}H_{41}N_5O_2+H]^+$ 479.3260, found 479.3246; **Comp. Purity (254 nm):** 100 %.

(*E*)-5-[(4-benzamido-3,5-dimethylphenyl)diazenyl]nicotinamide (**9h**)

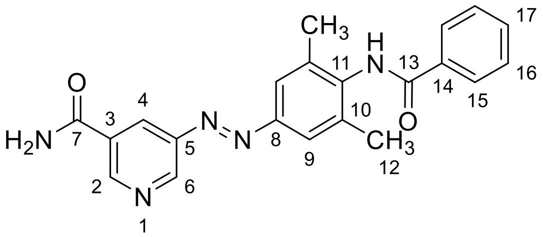

Synthesis was conducted according to the general procedure for the synthesis of nicotinamides from methyl nicotinates using **8h** (110 mg, 0.28 mmol, 1.0 equiv.). The product was obtained as orange solid (96 mg, 0.26 mmol, 93 %): R_f = 0.49 (EtOAc/MeOH 95:5); mp: 267.0 °C (decomp.); **^{1}H NMR (400 MHz, DMSO-*d$_6$*):** δ (ppm) = 9.98 (s, br, 1H, 11-*N*-H), 9.26 (d, *J* = 2.1 Hz, 1H, 6-H), 9.20 (d, *J* = 1.8 Hz, 1H, 2-H), 8.59 (pseudo-t, *J* = 2.0 Hz, 1H, 4-H), 8.05 (d, J = 7.3 Hz, 2H, 15-H), 7.78 (s, 3H, 9-H, 7-*N*-H), 7.63 (t, *J* = 7.2 Hz, 1H, 17-H), 7.56 (pseudo-t, J = 7.4 Hz, 2H, 16-H), 2.34 (s, 6H, 12-H); **^{13}C NMR, DEPT135, HSQC, HMBC (101 MHz, DMSO-*d$_6$*):** δ (ppm) = 165.7 (7), 165.1 (14), 150.7 (2), 150.1 (11), 148.3 (6), 147.0 (5), 139.4 (9), 137.2 (10), 134.0 (14), 131.7 (17), 128.5 (16), 127.6 (15), 125.7 (4), 122.2 (9), 18.3 (12); **IR (ATR):** *v* (cm^{-1}) = 3298, 1673, 1641, 1623, 1508, 1479, 1412, 1292, 1275, 1122, 694; **ESI-HRMS:** calcd. for $[C_{21}H_{19}N_5O_2+H]^+$ 373.1539, found 373.1542; **Comp. Purity (220 nm):** 100 %.

(*E*)-5-{[4-(4-fluorobenzamido)-3,5-dimethylphenyl]diazenyl}nicotinamide (**9i**)

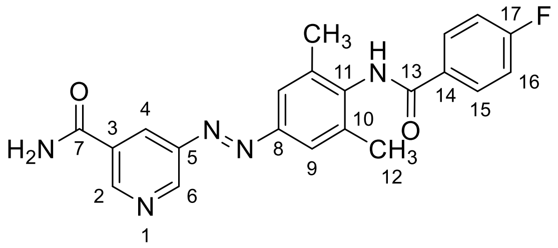

Synthesis was conducted according to the general procedure for the synthesis of nicotinamides from methyl nicotinates using **8i** (145 mg, 0.36 mmol, 1.00 equiv.). After evaporation of the solvent, the residue was purified by recrystallization (acetonitrile/THF). The title compound was obtained as orange solid (90 mg, 0.23 mmol, 64 %): R_f = 0.68 (EtOAc/MeOH 90:10); mp: 259.0 °C; **^{1}H NMR (400 MHz, DMSO-*d*$_6$):** δ (ppm) = 10.0 (s, 1H, 11-*N*-H), 9.26 (d, *J* = 2.2 Hz, 1H, 6-H), 9.20 (d, *J* = 2.0 Hz, 1H, 2-H), 8.59 (pseudo-t, *J* = 2.1 Hz, 1H, 4-H), 8.39 (s, br, 7-*N*-H), 8.12 (dd, *J* = 8.7, 5.5 Hz, 2H, 15-H), 7.78 (s, 2H, 9-H), 7.40 (t, *J* = 8.8 Hz, 2H, 16-H), 2.33 (s, 6H, 12-H); **^{13}C NMR, DEPT135, HSQC, HMBC (101 MHz, DMSO-*d*$_6$):** δ (ppm) = 165.6 (7), 164.1 (d, *J* = 249.2 Hz, 17), 163.9 (13), 150.6 (2), 150.0 (12), 148.2 (6), 146.9 (5), 139.2 (9), 137.1 (10), 130.5 (3), 130.4 (d, *J* = 2.9 Hz, 14), 130.2 (d, *J* = 9.1 Hz, 15), 125.6 (4), 122.2 (9), 115.4 (d, *J* = 21.8 Hz, 16), 18.2 (12); **IR (ATR):** *v* (cm^{-1}) = 3287, 1683, 1638, 1601, 1521, 1482, 1304, 1275, 1236, 1153, 845, 767, 632; **ESI-HRMS:** calcd. for $[C_{21}H_{18}N_5O_2F+H]^+$ 391.1445, found 391.1450; **Comp. Purity (220 nm):** 100 %.

(*E*)-5-({3,5-dimethyl-4-[4-(thiophen-2-yl)benzamido]phenyl}diazenyl)nicotinamide (**9j**)

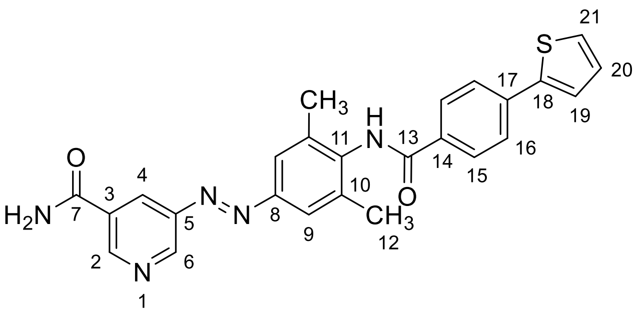

Synthesis was conducted according to the general procedure for the synthesis of nicotinamides from methyl nicotinates using **8j** (40 mg, 0.085 mmol, 1.00 equiv.). The product was obtained as orange solid (36 mg, 0.079 mmol, 93 %): R_f = 0.57 (EtOAc/MeOH 95:5); mp: 306.5 °C (decomp.); **^{1}H NMR (400 MHz, DMSO-*d*$_6$):** δ (ppm) = 10.0 (s, 1H, 11-*N*-H), 9.26 (d, *J* = 2.1 Hz, 1H, 6-H), 9.20 (d, *J* = 1.8 Hz, 1H, 2-H), 8.60 (s, 1H, 4-H), 8.39 (s, br, 1H, 7-*N*-H), 8.09 (d, *J* = 8.2 Hz, 2H, 15-H), 7.85 (d, *J* = 8.3 Hz, 2H, 16-H), 7.79 (s, 3H, 9-H, 7-*N*-H), 7.69 (d, *J* = 3.0 Hz, 1H, 19-H), 7.66 (d, *J* = 5.0 Hz, 1H, 21-H), 7.27–7.18 (m, 1H, 20-H), 2.34 (s, 6H, 12-H); **^{13}C NMR, DEPT135, HSQC, HMBC (101 MHz, DMSO-*d*$_6$):** δ (ppm) = 165.7 (7), 164.5 (13), 150.7 (2), 150.1 (11), 148.3 (6), 147.0 (5), 142.2 (18), 139.4 (8), 137.2 (10), 136.8 (17), 132.6 (14), 130.6 (3), 128.8 (20), 128.6 (15), 127.0 (21), 125.7 (4), 125.3 (16), 125.1 (19), 122.2 (9), 18.2 (12); **IR (ATR):** *v* (cm^{-1}) = 3099, 1686, 1641, 1606, 1517, 1415, 1304, 1127, 1111, 972, 706; **ESI-HRMS:** calcd. for $[C_{25}H_{21}N_5O_2+H]^+$ 155.1416, found 455.1425; **Comp. Purity (220 nm):** 100 %.

(*E*)-5-{[4-(adamantane-1-carboxamido)-3,5-dimethylphenyl]diazenyl}nicotinamide (**9k**)

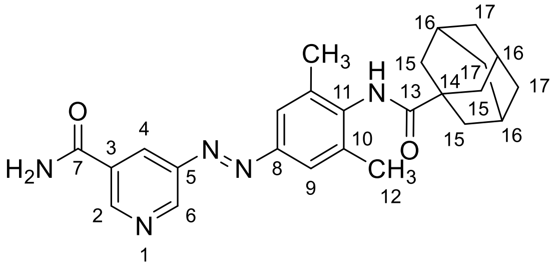

Synthesis was conducted according to the general procedure for the synthesis of nicotinamides from methyl nicotinates using **8k** (102 mg, 0.23 mmol, 1.0 equiv.). After evaporation of the solvent under reduced pressure, the residue was recrystallized (acetonitrile). The title compound was obtained as orange solid (76 mg, 0.18 mmol, 77 %): R_f = 0.52 (EtOAc/MeOH 95:5); mp: 313.1 °C (decomp.); **^{1}H NMR, H,H-COSY (400 MHz, $CDCl_3$):** δ (ppm) = 9.23 (d, *J* = 2.1 Hz, 1H, 6-H), 9.18 (d, *J* = 1.8 Hz, 1H, 2-H), 8.97 (s, br, 11-*N*-H), 8.57 (pseudo-t, *J* = 1.9 Hz, 1H, 4-H), 8.37 (s, br, 1H, 7-*N*-H), 7.78 (s, br, 1H, 7-*N*-H), 7.70 (s, 2H, 9-H), 2.23 (s, 6H, 12-H), 2.13–2.01 (m, 3H, 16-H), 1.98 (s, 6H, 15-H), 1.73 (s, 6H, 17-H); **^{13}C NMR, DEPT135, HSQC, HMBC (101 MHz, $CDCl_3$):** δ (ppm) = 175.6 (13), 165.6 (7), 150.5 (2), 149.8 (11), 148.1 (6), 146.9 (5), 139.8 (8), 137.0 (10), 130.5 (3), 125.6 (4), 122.0 (9), 40.5 (14), 38.7 (15), 36.1 (17), 27.6 (16), 18.0 (12); **IR (ATR):** *v* (cm^{-1}) = 2898, 2847, 1671, 1641, 1485, 1416, 1375, 1254, 1119, 693, 627; **ESI-HRMS:** calcd. for $[C_{25}H_{29}N_5O_2+Na]^+$ 431.2321, found 431.2317; **Comp. Purity (220 nm):** 100 %.

(*E*)-5-[(4-amino-3,5-dimethylphenyl)diazenyl]-*N*-methylnicotinamide (**12a**)

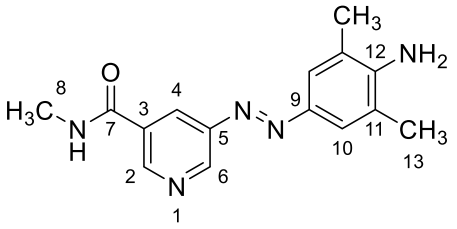

In a closed vessel, **11** (101 mg, 0.34 mmol, 1.0 equiv.) was stirred with methylamine (40 % in MeOH, 40 mL) for 2 days at 40 °C. After complete transformation it was evaporated to dryness under reduced pressure. The title compound was obtained as orange solid (96 mg, 0.34 mmol, 100 %): R_f = 0.30 (DCM/MeOH 98:2); mp: 209.0 °C; **^{1}H NMR, H,H-COSY (400 MHz, DMSO-*d$_6$*):** δ (ppm) = 9.06 (d, *J* = 2.3 Hz, 1H, 6-H), 8.99 (d, *J* = 2.0 Hz, 1H, 2-H), 8.78 (d, *J* = 4.5 Hz, 1H, 7-*N*-H), 8.40 (pseudo-t, *J* = 2.2 Hz, 1H, 4-H), 7.56 (s, 2H, 10-H), 5.80 (s, 2H, 12-*N*-H), 2.84 (d, *J* = 4.5 Hz, 3H, 8-H), 2.20 (s, 6H, 13-H); **^{13}C NMR, DEPT135, HSQC, HMBC (101 MHz, DMSO-*d$_6$*):** δ (ppm) = 164.7 (7), 150.0 (12), 148.1 (2), 147.5 (5), 147.4 (6), 142.6 (9), 130.5 (3), 124.3 (4), 124.2 (10), 120.6 (11), 26.2 (8), 17.8 (13); **IR (ATR):** *v* (cm^{-1}) = 3291, 1643, 1550, 1481, 1396, 1311, 1124, 1113, 896, 698, 484; **ESI-HRMS:** calcd. for $[C_{15}H_{17}N_5O+H]^+$ 283.1433, found 283.1431; **Comp. Purity (220 nm):** 100 %.

(*E*)-5-[(4-amino-3,5-dimethylphenyl)diazenyl]-*N*-ethylnicotinamide (**12b**)

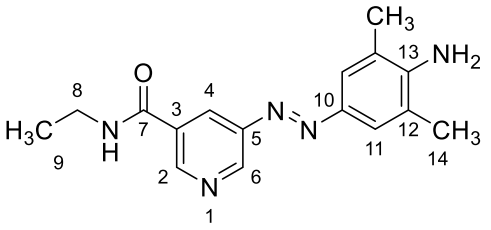

In a closed vessel, **11** (80 mg, 0.27 mmol, 1.0 equiv.) was stirred with ethylamine (20 % in MeOH, 30 mL) for 2 days at 50 °C. After complete transformation it was evaporated to dryness under reduced pressure. The title compound was obtained as orange solid (81 mg, 0.27 mmol, 100 %): R_f = 0.70 (cyclohexane/THF 1:2); mp: 190.9 °C; **^{1}H NMR, H,H-COSY (400 MHz, DMSO-*d*$_6$):** δ (ppm) = 9.06 (d, *J* = 2.2 Hz, 1H, 6-H), 8.99 (d, *J* = 2.0 Hz, 1H, 2-H), 8.81 (t, *J* = 5.3 Hz, 1H, 7-*N*-H), 8.40 (pseudo-t, *J* = 2.1 Hz, 1H, 4-H), 7.56 (s, 2H, 11-H), 5.80 (s, br, 2H, 13-*N*-H), 3.38–3.29 (m, 2H, 8-H), 2.20 (s, 6H, 14-H), 1.16 (t, *J* = 7.2 Hz, 3H, 9-H); **^{13}C NMR, DEPT135, HSQC, HMBC (101 MHz, DMSO-*d*$_6$):** δ (ppm) = 164.0 (7), 150.0 (13), 148.2 (2), 147.5 (5), 147.3 (6), 142.6 (10), 130.7 (3), 124.3 (4), 124.1 (11), 120.6 (12), 34.1 (8), 17.8 (14), 14.5 (9); **IR (ATR):** *v* (cm^{-1}) = 3378, 3208, 2874, 1671, 1625, 1539, 1480, 1459, 1399, 1316, 1194, 1124; **ESI-HRMS:** calcd. for $[C_{16}H_{19}N_5O+H]^+$ 297.1590, found 297.1599; **Comp. Purity (220 nm):** 100 %.

(*E*)-5-[(4-amino-3,5-dimethylphenyl)diazenyl]-*N*-butylnicotinamide (**12c**)

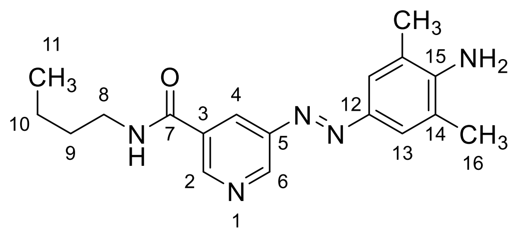

In a closed vessel, **11** (110 mg, 0.37 mmol, 1.0 equiv.) was stirred with *n*-butylamine (6 mL) for 4 days at 50 °C. After complete transformation, excessive *n*-butylamine was evaporated under reduced pressure. The resulting residue was purified by reversed phase column chromatography (water/MeOH binary gradient 40→90 %). The title compound was obtained as yellow-orange solid (98 mg, 0.30 mmol, 80 %): R_f = 0.69 (cyclohexane/THF 1:2); mp: 173.6 °C; **^{1}H NMR, H,H-COSY (400 MHz, DMSO-*d*$_6$):** δ (ppm) = 9.06 (d, *J* = 2.2 Hz, 1H, 6-H), 8.99 (d, *J* = 2.0 Hz, 1H, 2-H), 8.78 (t, *J* = 5.5 Hz, 1H, 7-*N*-H), 8.40 (pseudo-t, *J* = 2.1 Hz, 1H, 4-H), 7.56 (s, 2H, 13-H), 5.80 (s, 2H, 15-*N*-H), 3.36–3.23 (m, 2H, 8-H), 2.20 (s, 6H, 16-H), 1.61–1.49 (m, 2H, 9-H), 1.42–1.29 (m, 2H, 10-H), 0.92 (t, *J* = 7.3 Hz, 3H, 11-H); **^{13}C NMR, DEPT135, HSQC, HMBC (101 MHz, DMSO-*d*$_6$):** δ (ppm) = 164.2 (7), 150.0 (15), 148.2 (2), 147.5 (5), 147.3 (6), 142.6 (12), 130.7 (3), 124.4 (4), 124.2 (13), 120.6 (14), 38.9 (8), 31.0 (9), 19.6 (10), 17.8 (16), 13.6 (11); **IR (ATR):** *v* (cm^{-1}) = 3286, 1634, 1547, 1480, 1403, 1307, 1293, 1118, 907, 892, 699; **ESI-HRMS:** calcd. for $[C_{18}H_{23}N_5O+H]^+$ 325.1903, found 325.1898; **Comp. Purity (220 nm):** 100 %.

(*E*)-5-[(4-amino-3,5-dimethylphenyl)diazenyl]-*N*-(2-hydroxyethyl)nicotinamide (**12d**)

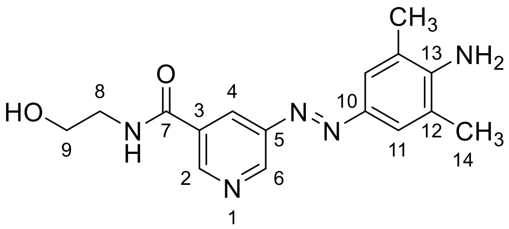

In a closed vessel, **11** (75 mg, 0.25 mmol, 1.0 equiv.) was stirred with ethanolamine (3 mL) for 3 days at 110 °C. After complete transformation, excessive ethanolamine was evaporated under reduced pressure. The resulting residue was purified by reversed phase column chromatography (water/MeOH binary gradient 40→90 %). The title compound was obtained as yellow solid (69 mg, 0.22 mmol, 88 %): R_f = 0.30 (EtOAc/MeOH 95:5); mp: 192.5 °C; **^{1}H NMR, H,H-COSY (400 MHz, DMSO-*d₆*):** δ (ppm) = 9.06 (d, *J* = 2.2 Hz, 1H, 6-H), 9.01 (d, *J* = 2.0 Hz, 1H, 2-H), 8.80 (t, *J* = 5.5 Hz, 1H, 7-*N*-H), 8.42 (pseudo-t, *J* = 2.1 Hz, 1H, 4-H), 7.56 (s, 2H, 11-H), 5.80 (s, 2H, 13-*N*-H), 4.78 (s, br, 1H, 9-*O*-H), 3.59–3.52 (m, 2H, 8-H), 3.42–3.36 (m, 2H, 9-H), 2.20 (s, 6H, 14-H); **^{13}C NMR, DEPT135, HSQC, HMBC (101 MHz, DMSO-d_6):** δ (ppm) = 164.5 (7), 149.9 (13), 148.3 (2), 147.5 (5), 147.4 (6), 142.6 (10), 130.6 (3), 124.4 (4), 124.2 (11), 120.6 (12), 59.5 (9), 42.2 (8), 17.8 (14); **IR (ATR):** *v* (cm^{-1}) = 3359, 3125, 1669, 1628, 1429, 1378, 1136, 838, 808, 660, 476; **ESI-HRMS:** calcd. for $[C_{16}H_{19}N_5O_2+H]^+$ 313.1539, found 313.1551; **Comp. Purity (220 nm):** 100 %.

Methyl (*E*)-5-{[4-(tert-butoxycarbonyl)phenyl]diazenyl}nicotinate (**14a**)

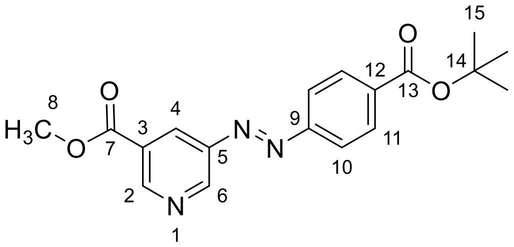

A solution of oxone® (12.31 g, 20.00 mmol, 2.0 equiv.) in water (75 mL) was given to a solution of *tert*-butyl 4-aminobenzoate (1.93 g, 10.00 mmol, 1.0 equiv.) in DCM (50 mL). After 2 days of vigorous stirring at room temperature the aqueous phase was separated and extracted with DCM (2 × 50 mL). The combined organic phases were washed successively with an aq. HCl-solution (1 M, 2 × 50 mL), water (2 × 50 mL) and sat. aq. NaCl-solution (50 mL), dried over $MgSO_4$ and filtrated. After evaporation of the solvent under reduced pressure the crude nitroso compound was set under an argon atmosphere and dissolved in glacial acetic acid (25 mL). Compound **5** (1.52 g, 10 mmol, 1.0 equiv.) was added in one portion and the resulting mixture stirred for 4 days at room temperature. The reaction mixture was poured onto ice-cooled sat. aq. $NaHCO_3$-solution and extracted with EtOAc (3 × 100 mL). The combined organic extracts were washed successively with water (3 × 100 mL) and brine (2 × 100 mL), dried over $MgSO_4$, filtrated and freed from solvent under reduced pressure. The crude residue was purified by silica gel column chromatography (cyclohexane/THF 2.5:1) and yielded **11a** as orange solid (1.36 g, 3.98 mmol, 40 %): R_f = 0.68 (cyclohexane/THF 2.5:1); mp: 103.9 °C; **^{1}H NMR, H,H-COSY (400 MHz, DMSO-d_6):** δ (ppm) = 9.41 (d, *J* = 2.3 Hz, 1H, 6-H), 9.25 (d, *J* = 2.0 Hz, 1H, 2-H), 8.55 (pseudo-t, *J* = 2.2 Hz, 1H, 4-H), 8.13 (d, *J* = 8.7 Hz, 1H, 11-H), 8.05 (d, *J* = 8.7 Hz, 2H, 10-H), 3.96 (s, 3H, 8-H), 1.59 (s,

9H, 15-H); **^{13}C NMR, DEPT135, HSQC, HMBC (101 MHz, DMSO-*d₆*):** δ (ppm) = 164.5 (7), 164.1 (13), 153.9 (12), 152.3 (2), 150.7 (6), 146.8 (5), 134.1 (9), 130.4 (11), 126.7 (4), 126.5 (3), 123.0 (10), 81.5 (14), 52.8 (8), 27.7 (15); **IR (ATR):** *ν* (cm^{-1}) = 2971, 1728, 1709, 1303, 1213, 1176, 1116, 1094, 847, 774, 697.

Methyl (*E*)-5-({4-[2-(tert-butoxy)-2-oxoethyl]phenyl}diazenyl)nicotinate (**14b**)

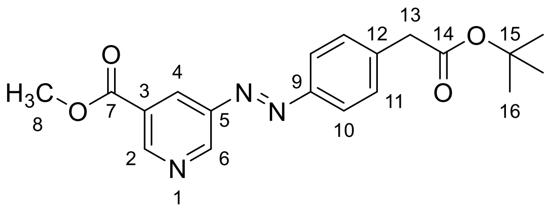

Synthesis was conducted according the procedure of **14a** using *tert*-butyl 2-(4-aminobenzyl)acetate (1,00 g, 4.81 mmol, 1.0 equiv.) in DCM (100 mL) and oxone® (5.94 g, 9.65 mmol, 2.0 equiv.) in water (50 mL). The crude nitroso compound was reacted with **5** (732 mg, 4.81 mmol, 1.0 equiv.) in glacial acetic acid (10 mL) by stirring under an argon atmosphere for two weeks at room temperature. Purification of the crude product by silica gel column chromatography (cyclohexane/THF 2:1) yielded the title compound as orange solid (122 mg, 0.34 mmol, 8 %): **^{1}H NMR, H,H-COSY (400 MHz, DMSO-*d₆*):** δ (ppm) = 9.36 (d, *J* = 2.2 Hz, 1H, 6-H), 9.23 (d, *J* = 1.8 Hz, 1H, 2-H), 8.53 (pseudo-t, *J* = 2.2 Hz, 1H, 4-H), 7.94 (d, *J* = 8.4 Hz, 1H, 10-H),7.52 (d, *J* = 8.5 Hz, 2H, 11-H), 3.95 (s, 3H, 8-H), 3.72 (s, 2H, 13-H), 1.42 (s, 9H, 16-H); **^{13}C NMR, DEPT135, HSQC, HMBC (101 MHz, DMSO-*d₆*):** δ (ppm) = 169.8 (14), 164.6 (7), 151.7 (2), 150.6 (9), 150.3 (6), 146.8 (5), 139.8 (12), 134.8 (3), 130.5 (10), 126.4 (4), 122.9 (11), 80.4 (15), 52.7 (8), 41.3 (13), 27.6 (16); **IR (ATR):** *ν* (cm^{-1}) = 2978, 1774, 1726, 1599, 1286, 1141, 1097, 990, 767, 688, 576.

Methyl (*E*)-5-[(4-{[(tert-butoxycarbonyl)amino]methyl}phenyl)diazenyl]nicotinate (**14c**)

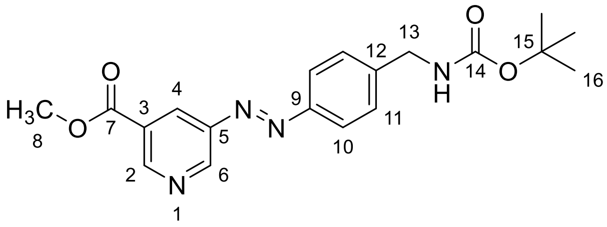

Synthesis was conducted according the procedure of **14a** using *tert*-butyl (4-aminobenzyl)carbamate (600 mg, 2.70 mmol, 1.3 equiv.) in DCM (20 mL) and oxone® (3.32 g, 5.40 mmol, 2.6 equiv.) in water (20 mL). The crude nitroso compound was reacted with **5** (317 mg, 2.08 mmol, 1.0 equiv.) in glacial acetic acid (10 mL) by stirring under an argon atmosphere for two weeks at 50 °C. Purification of the crude product by silica gel column chromatography (cyclohexane/EtOAc 2:1) yielded the title compound as orange solid (290 mg, 0.78 mmol, 38 %): mp: 128.8 °C; **^{1}H NMR, H,H-COSY (400 MHz, DMSO-*d₆*):** δ (ppm) = 9.36 (d, *J* = 2.3 Hz, 1H, 6-H), 9.22 (d, *J* = 2.0 Hz, 1H, 2-H), 8.53 (pseudo-t, *J* = 2.2 Hz, 1H, 4-H), 7.95 (d, *J* = 8.3 Hz, 1H, 10-H),7.58–7.53 (m, 1H, *N*-H), 7.49 (d, *J* = 8.4 Hz, 2H, 11-H), 4.25 (d, *J* = 6.1 Hz, 2H, 13-H), 3.95 (s, 3H, 8-H), 1.42 (s, 9H, 16-H); **^{13}C NMR, DEPT135, HSQC, HMBC (101 MHz, DMSO-*d₆*):** δ (ppm) = 164.6 (7), 155.8 (14), 151.6 (2), 150.7 (9), 150.3 (6), 146.8 (5), 145.2

(12), 127.8 (11), 126.4 (3), 126.4 (4), 123.0 (10), 77.9 (13), 52.7 (15), 43.1 (13), 28.1 (16); **IR (ATR):** *v* (cm^{-1}) = 3349, 1723, 1683, 1505, 1290, 1244, 1161, 1049, 767, 688, 576.

Methyl (*E*)-5-[(4-bromophenyl)diazenyl]nicotinate (**14d**)

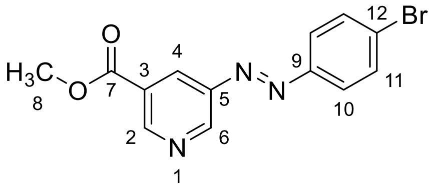

Synthesis was conducted according the procedure of **14a** using 4-bromoaniline (516 mg, 3.00 mmol, 1.0 equiv.) in DCM (10 mL) and oxone® (3.69 g, 6.00 mmol, 2.0 equiv.) in water (20 mL). The crude nitroso compound was reacted with **5** (558 mg, 3.00 mmol, 1.0 equiv.) in glacial acetic acid (25 mL) by stirring under an argon atmosphere for 5 days at room temperature. Purification of the crude product by silica gel column chromatography (cyclohexane/THF 5:1) yielded the title compound as orange-red crystals (413 mg, 1.29 mmol, 43 %): mp: 142.7 °C; **^{1}H NMR, H,H-COSY (400 MHz, DMSO-*d*$_6$):** δ (ppm) = 9.37 (d, *J* = 2.3 Hz, 1H, 6-H), 9.24 (d, *J* = 2.0 Hz, 1H, 2-H), 8.54 (pseudo-t, *J* = 2.1 Hz, 1H, 4-H), 7.96–7.89 (m, 2H, 11-H), 7.89–7.83 (m, 2H, 10-H), 3.95 (s, 3H, 8-H); **^{13}C NMR, DEPT135, HSQC, HMBC (101 MHz, DMSO-*d*$_6$):** δ (ppm) = 164.5 (7), 152.0 (2), 150.6 (12), 150.4 (6), 146.7 (5), 132.7 (11), 126.6 (4), 126.5 (3), 126.2 (9), 124.8 (10), 52.7 (8); **IR (ATR):** *v* (cm^{-1}) = 3048, 1718, 1566, 1478, 1430, 1417, 1396, 1306, 1289, 1098, 829.

Methyl (*E*)-5-{[4-(butylcarbamoyl)phenyl]diazenyl}nicotinate (**14e**)

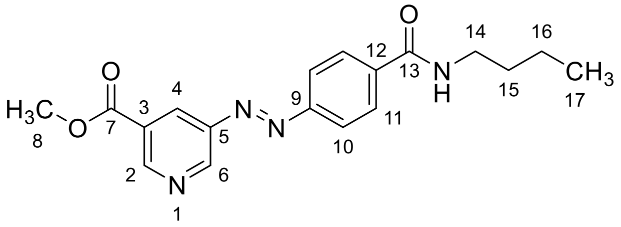

TFA (10 mL) was slowly added to a solution of **14a** (300 mg, 0.88 mmol, 1.0 equiv.) in dry DCM (50 mL) and stirred over night at room temperature. The reaction mixture was evaporated to dryness under reduced pressure. Residual TFA was removed by azeotropic distillation with DCM (3 × 50 mL). The resulting crude carboxylic acid was dissolved in dry DMF (10 mL) and treated with DIPEA (537 µL, 3.08 mmol, 3.5 equiv.) and HATU (1.00 g, 2.64 mmol, 3.0 equiv.). After 15 min *n*-butylamine (105 µL, 1.06 mmol,1.2 equiv.) was added and the resulting mixture was stirred over night at room temperature. Water (50 mL) was added to the reaction mixture prior to extraction with EtOAc (3 × 50 mL). The combined organic extracts were washed successively with aq. sat. NH_4Cl-solution (3 × 20 mL), water (3 × 20 mL) and aq. sat. NaCl-solution (2 × 20 mL). It was dried over $MgSO_4$, filtrated and freed from solvent under reduced pressure. The residue was purified by silica gel column chromatography (cyclohexane/EtOAc 1:1) and yielded the title compound as orange solid (200 mg, 0.54 mmol, 61 %): R_f = 0.41 (cyclohexane/EtOAc 1:1); mp: 164.0 °C; **^{1}H NMR, H,H-COSY (400 MHz, DMSO-*d*$_6$):** δ (ppm) = 9.41 (d, *J* = 2.3 Hz, 1H, 6-H), 9.26 (d, *J* = 2.0 Hz, 1H, 2-H), 8.66 (t, *J* = 5.6 Hz, 1H, 13-*N*-H), 8.58–8.56 (m, 1H, 4-H), 8.11–8.06 (m, 2H, 11-H), 8.06–8.02 (m, 2H, 10-H), 3.96 (s, 3H, 8-H), 3.33–3.26 (m, 2H, 14-H), 1.59–1.50 (m, 2H, 15-H), 1.41–1.30 (m, 2H, 16-H), 0.92 (t, *J* = 7.3 Hz,

3H, 17-H); **^{13}C NMR, DEPT135, HSQC, HMBC (101 MHz, DMSO-*d$_6$*):** δ (ppm) = 165.1 (13), 164.5 (7), 152.9 (12), 152.1 (2), 150.5 (6), 146.8 (5), 137.7 (9), 128.5 (11), 126.5 (4), 126.5 (3), 122.7 (10), 52.7 (8), 38.9 (14), 31.1 (15), 19.6 (16), 13.6 (17); **IR (ATR):** *v* (cm^{-1}) = 3300, 2957, 2927, 1728, 1630, 1550, 1305, 1287, 1218, 1178, 858.

Methyl (*E*)-5-{[4-(decanamidomethyl)phenyl]diazenyl}nicotinate (**14f**)

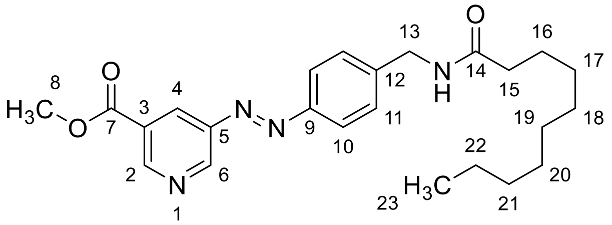

TFA (2 mL) was slowly added to a solution of **14c** (152 mg, 0.41 mmol, 1.0 equiv.) in dry DCM (15 mL) and stirred over night at room temperature. The reaction mixture was evaporated to dryness under reduced pressure. Residual TFA was removed by azeotropic distillation with DCM (3 × 40 mL). The obtained crude ammonium salt was dissolved in dry THF (15 mL), treated with DIPEA (214 µL, 1.23 mmol, 3.0 equiv.) and decanoyl chloride (128 µL, 0,62 mmol, 1.5 equiv.) and stirred over night at room temperature. The reaction mixture was concentrated under reduced pressure, taken up in EtOAc (50 mL) and washed successively with water (3 × 30 mL) and aq. sat. NaCl-solution (30 mL). It was dried over $MgSO_4$, filtrated and freed from solvent under reduced pressure. The residue was purified by silica gel column chromatography (cyclohexane/THF 1.5:1) and recrystallization (cyclohexane/THF 2:1) and yielded the title compound as yellow solid (139 mg, 0.33 mmol, 80 %): mp: 143.7 °C; **^{1}H NMR (400 MHz, DMSO-*d$_6$*):** δ (ppm) = 9.36 (d, *J* = 2.3 Hz, 1H, 6-H), 9.23 (d, *J* = 1.9 Hz, 1H, 2-H), 8.54 (pseudo-t, *J* = 2.1 Hz, 1H, 4-H), 8.43 (t, J = 6.0 Hz, 1H, 13-*N*-H), 7.95 (d, *J* = 8.4 Hz, 1H, 10-H),7.49 (d, *J* = 8.4 Hz, 2H, 11-H), 4.37 (d, *J* = 6.0 Hz, 2H, 13-H), 3.95 (s, 3H, 8-H), 2.17 (t, J = 7.3 Hz, 2H, 15-H), 1.52 (m, 2H, 16-H), 1.33–1.09 (m, 12H, 17 – 22-H), 0.84 (t, J = 6.1 Hz, 3H, 23-H); **IR (ATR):** *v* (cm^{-1}) = 3280, 2920, 2849, 1725, 1639, 1535, 1281, 1212, 1176, 836, 700.

Methyl (*E*)-5-{[4-(6-hydroxyhex-1-yn-1-yl)phenyl]diazenyl}nicotinate (**14g**)

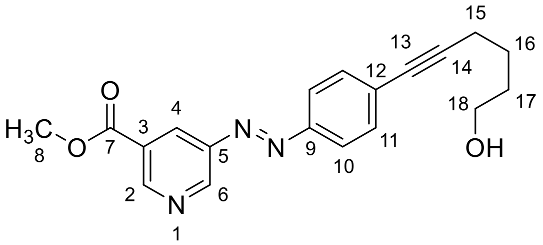

In a Schlenk flask, **14d** (320 mg, 1.00 mmol, 1.0 equiv.) was set under an argon atmosphere and dissolved in a mixture of NEt_3 and dry DCM (3:1, 15 mL). Hex-5-yn-1-ol (108 µL, 1.21 mmol, 1.2 equiv.), CuI (6 mg, 0.03 mmol, 3 mol%) and tetrakis(triphenylphosphine)palladium(0) (35 mg, 0.03 mmol, 3 mol%) were added successively. The resulting reaction mixture was stirred at 50 °C for 24 hours. After cooling to room temperature EtOAc (50 mL) was added and the organic layer separated. The aqueous phase was extracted with EtOAc (2 × 20 mL) and the combined organic phases washed successively with water (3 × 20 mL) and sat. aq. NaCl-solution (20 mL). It was dried over $MgSO_4$, filtrated and freed from solvent under reduced pressure. Purification of the residue by

silica gel column chromatography (*n*-hexane/EtOAc 2:1) yielded the title compound as orange solid (64 mg, 0.19 mmol, 19 %): R_f = 0.48 (*n*-hexane/EtOAc 2:1); mp: 91.0 °C; **^{1}H NMR, H,H-COSY (400 MHz, DMSO-*d*$_6$):** δ (ppm) = 9.36 (d, *J* = 2.3 Hz, 1H, 6-H), 9.23 (d, *J* = 2.0 Hz, 1H, 2-H), 8.53 (pseudo-t, *J* = 2.2 Hz, 1H, 4-H), 7.98–7.91 (m, 2H, 10-H), 7.66–7.59 (m, 2H, 11-H), 4.45 (t, *J* = 5.2 Hz, 1H, 18-*O*-H), 3.95 (s, 3H, 8-H), 3.46 (q, *J* = 6.0 Hz, 2H, 18-H), 2.55–2.46 (m, 2H, 15-H), 1.67–1.55 (m, 4H, 16-H, 17-H); **^{13}C NMR, DEPT135, HSQC, HMBC (101 MHz, DMSO-*d*$_6$):** δ (ppm) = 164.5 (7), 151.8 (2), 150.5 (9), 150.4 (6), 146.8 (5), 132.4 (11), 127.5 (12), 126.4 (3+4), 123.2 (10), 94.8 (14), 80.2 (13), 60.1 (18), 52.7 (8), 31.6 (17), 24.7 (16), 18.6 (15); **IR (ATR):** *v* (cm^{-1}) = 2942, 1726, 1584, 1496, 1419, 1287, 1250, 1176, 1156, 843, 693.

Methyl (E)-5-{[4-(3-hydroxy-3-methylbut-1-yn-1-yl)phenyl]diazenyl}nicotinate (**14h**)

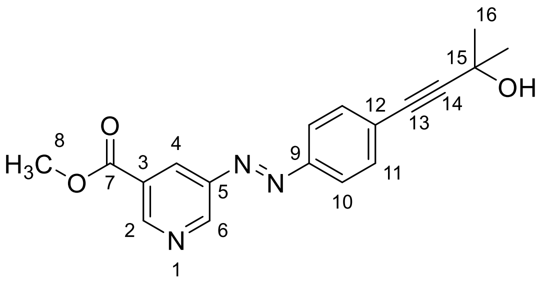

In a Schlenk flask, **14d** (320 mg, 1.00 mmol, 1.0 equiv.) was set under an argon atmosphere and dissolved in a mixture of NEt_3 and dry DMF (4:1, 20 mL). To this solution were added successively 2-methylbut-3-yn-2-ol (118 µL, 1.21 mmol, 1.2 equiv.), CuI (6 mg, 0.03 mmol, 3 mol%) and tetrakis(triphenylphosphine)palladium(0) (35 mg, 0.03 mmol, 3 mol%). The resulting reaction mixture was stirred at 85 °C for 24 hours. After cooling to room temperature the reaction mixture was taken up in EtOAc (50 mL) and washed successively with water (3 × 20 mL) and sat. aq. NaCl-solution (20 mL). It was dried over $MgSO_4$, filtrated and freed from solvent under reduced pressure. Purification of the residue by silica gel column chromatography (*n*-hexane/EtOAc 2:1) yielded the title compound as orange solid (64 mg, 0.20 mmol, 20 %): R_f = 0.48 (*n*-hexane/EtOAc 2:1); mp: 91.0 °C; **^{1}H NMR, H,H-COSY (400 MHz, DMSO-*d*$_6$):** δ (ppm) = 9.36 (d, *J* = 2.3 Hz, 1H, 6-H), 9.23 (d, *J* = 2.0 Hz, 1H, 2-H), 8.52 (pseudo-t, *J* = 2.2 Hz, 1H, 4-H), 8.01–7.91 (m, 2H, 10-H), 7.67–7.58 (m, 2H, 11-H), 5.85 (s, 1H, 15-O-H), 3.95 (s, 3H, 8-H), 1.51 (s, 6H, 16-H); **^{13}C NMR, DEPT135, HSQC, HMBC (101 MHz, DMSO-*d*$_6$):** δ (ppm) = 164.5 (7), 151.9 (2), 150.7 (9), 150.4 (6), 146.8 (5), 132.4 (11), 126.7 (12), 126.5 (4), 126.4 (3), 123.2 (10), 99.6 (14), 79.9 (13), 63.6 (15), 52.7 (8), 31.4 (16); **IR (ATR):** *v* (cm^{-1}) = 3283, 1723, 1581, 1434, 1418, 1304, 1174, 1139, 969, 766, 691.

(*E*)-5-{[4-(tert-butoxycarbonyl)phenyl]diazenyl}nicotinamide (**15a**)

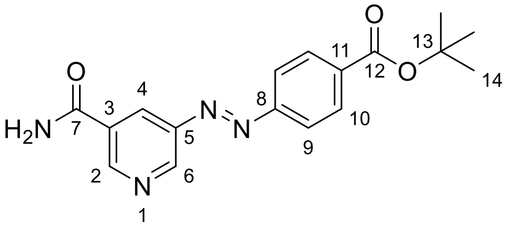

Synthesis was conducted according to the general procedure for the synthesis of nicotinamides from methyl nicotinates using **14a** (200 mg, 0.59 mmol, 1.00 equiv.). The product was obtained as salmon-coloured solid (192 mg, 0.59, mmol, 100 %): R_f = 0.70 (EtOAc/MeOH 95:5); mp: 319.5 °C; **^{1}H NMR, H,H-COSY (400 MHz, DMSO-*d*$_6$):** δ (ppm)

= 9.30 (d, *J* = 2.3 Hz, 1H, 6-H), 9.23 (d, *J* = 2.0 Hz, 1H, 2-H), 8.62 (pseudo-t, *J* = 2.2 Hz, 1H, 4-H), 8.40 (s, br, 1H, *N*-H), 8.15 (d, *J* = 8.8 Hz, 2H, 11-H), 8.04 (d, *J* = 8.8 Hz, 2H, 10-H), 7.81 (s, br, 1H, *N*-H), 1.59 (s, 9H, 14-H); **^{13}C NMR, DEPT135, HSQC, HMBC (101 MHz, DMSO-*d*$_6$):** δ (ppm) = 165.5 (7), 164.2 (12), 154.0 (11), 151.4 (2), 148.7 (6), 146.8 (5), 134.0 (9), 130.4 (11), 130.6 (3), 125.6 (4), 122.9 (10), 81.4 (13), 27.7 (14); **IR (ATR):** *v* (cm^{-1}) = 3371, 3141, 2977, 1706, 1676, 1294, 1121, 918, 774, 700, 557; **ESI-HRMS:** calcd. for $[C_{17}H_{18}N_4O_3+H]^+$ 326.1379, found 326.1364; **Comp. Purity (220 nm):** 100 % (*E*+*Z*)-**15a**.

(*E*)-5-({4-[2-(tert-butoxy)-2-oxoethyl]phenyl}diazenyl)nicotinamide (**15b**)

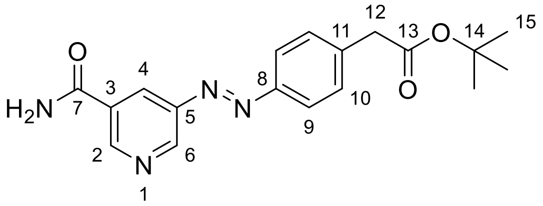

Synthesis was conducted according to the general procedure for the synthesis of nicotinamides from methyl nicotinates using **14b** (122 mg, 0.36 mmol, 1.0 equiv.). After evaporation of the solvent under reduced pressure, the residue purified by silica gel column chromatography (DCM/MeOH 96:4) and successive recrystallization (acetonitrile). The title compound was obtained as orange solid (77 mg, 0.23 mmol, 64 %): R_f = 0.22 (DCM/MeOH 96:4); mp: 250.0 °C (decomp.); **^{1}H NMR, H,H-COSY (400 MHz, DMSO-*d*$_6$):** δ (ppm) = 9.24 (d, *J* = 2.3 Hz, 1H, 6-H), 9.18 (d, *J* = 2.0 Hz, 1H, 2-H), 8.57 (pseudo-t, *J* = 2.2 Hz, 1H, 4-H), 8.38 (s, br, 1H, 7-*N*-H), 7.93 (d, *J* = 8.4 Hz, 1H, 9-H), 7.78 (s, br, 1H, 7-*N*-H), 7.52 (d, *J* = 8.5 Hz, 2H, 10-H), 3.72 (s, 2H, 12-H), 1.42 (s, 9H, 15-H); **^{13}C NMR, DEPT135, HSQC, HMBC (101 MHz, DMSO-*d*$_6$):** δ (ppm) = 169.8 (13), 165.6 (7), 150.6 (2+8), 148.2 (6), 146.8 (5), 139.5 (11), 130.5 (3+10), 125.6 (4), 122.8 (9), 80.4 (14), 41.3 (12), 27.6 (15); **IR (ATR):** *v* (cm^{-1}) = 3366, 3289, 2920, 2851, 1671, 1640, 1544, 1402, 1220, 1145, 694; **ESI-HRMS:** calcd. for $[C_{18}H_{20}N_4O_3+H]^+$ 340.1535, found 340.1535; **Comp. Purity (220 nm):** 100 % (*E*+*Z*)-**15b**.

(*E*)-5-[(4-{[(tert-butoxycarbonyl)amino]methyl}phenyl)diazenyl]nicotinamide (**15c**)

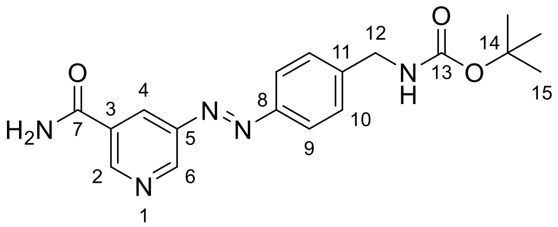

Synthesis was conducted according to the general procedure for the synthesis of nicotinamides from methyl nicotinates using **14c** (67 mg, 0.18 mmol, 1.00 equiv.). The product was obtained as yellow solid (64 mg, 0.18 mmol, 100 %): R_f = 0.63 (EtOAc/MeOH 95:5); mp: 212.1 °C (Decomp.); **^{1}H NMR, H,H-COSY (400 MHz, DMSO-*d*$_6$):** δ (ppm) = 9.23 (d, *J* = 2.3 Hz, 1H, 6-H), 9.18 (d, *J* = 2.0 Hz, 1H, 2-H), 8.57 (pseudo-t, *J* = 2.2 Hz, 1H, 4-H), 8.38 (s, br, 1H, 7-*N*-H), 7.94 (d, *J* = 8.3 Hz, 1H, 9-H), 7.78 (s, br, 1H, 7-*N*-H), 7.57–7.51 (m, 1H, 12-*N*-H), 7.49 (d, *J* = 8.4 Hz, 2H, 10-H), 4.25 (d, *J* = 6.1 Hz, 2H, 12-H), 1.42 (s, 9H, 15-H); **^{13}C NMR, DEPT135, HSQC, HMBC (101 MHz, DMSO-*d*$_6$):** δ (ppm) = 165.6 (7), 155.8 (13), 150.7 (8), 150.6 (2), 148.2 (6), 148.8 (5), 145.0 (11), 130.5 (3),

127.8 (9), 125.6 (4), 122.8 (10), 77.9 (14), 43.1 (12), 28.1 (15); **IR (ATR):** *v* (cm^{-1}) = 3345, 3134, 1684, 1526, 1250, 1170, 1142, 916, 836, 694, 665; **ESI-HRMS:** calcd. for $[C_{18}H_{21}N_5O_3+H]^+$ 355.1644, found 355.1629; **Comp. Purity (220 nm):** 100 % (*E*+*Z*)-**15c**.

(*E*)-5-[(4-bromophenyl)diazenyl]nicotinamide (**15d**)

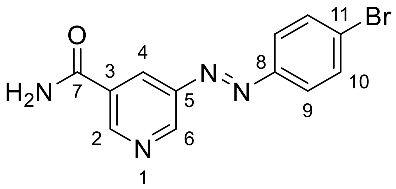

Synthesis was conducted according to the general procedure for the synthesis of nicotinamides from methyl nicotinates using **14d** (76 mg, 0.24 mmol, 1.0 equiv.). The product was obtained as orange solid (72 mg, 0.24 mmol, 100 %): R_f = 0.72 (*n*-hexane/acetone 1:2); mp: 231.5 °C; **^{1}H NMR, H,H-COSY (400 MHz, DMSO-*d₆*):** δ (ppm) = 9.26 (d, *J* = 2.3 Hz, 1H, 6-H), 9.21 (d, *J* = 2.0 Hz, 1H, 2-H), 8.59 (pseudo-t, *J* = 2.2 Hz, 1H, 4-H), 8.38 (s, br, 1H, 7-*N*-H), 7.96–7.89 (m, 2H, 9-H), 7.89–7.83 (m, 2H, 10-H), 7.80 (s, br, 7-*N*-H); **^{13}C NMR, DEPT135, HSQC, HMBC (101 MHz, DMSO-d_6):** δ (ppm) = 164.6 (7), 151.1 (2), 150.7 (11), 148.5 (6), 146.8 (5), 132.8 (10), 130.6 (3), 126.0 (8), 125.8 (4), 124.7 (9); **IR (ATR):** *v* (cm^{-1}) = 3362, 3161, 1655, 1625, 1568, 1479, 1388, 1136, 1097, 1062, 1003, 689; **ESI-HRMS:** calcd. For $[C_{12}H_9N_4OBr+H]^+$ 303.9960, found 303.9950; **Comp. Purity (220 nm):** 100 %.

(*E*)-5-{[4-(butylcarbamoyl)phenyl]diazenyl}nicotinamide (**15e**)

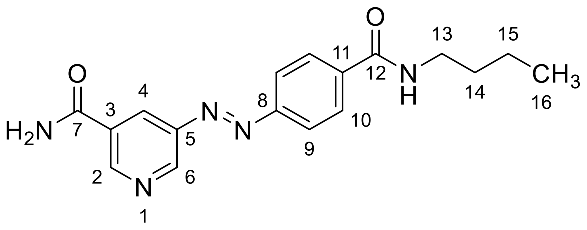

Synthesis was conducted according to the general procedure for the synthesis of nicotinamides from methyl nicotinates using **14a** (100 mg, 0.29 mmol, 1.00 equiv.). The product was obtained as brownish solid (94 mg, 0.29 mmol, 100 %): R_f = 0.51 (EtOAc/MeOH 95:5); mp: 292.3 °C; **^{1}H NMR, H,H-COSY (400 MHz, DMSO-d_6):** δ (ppm) = 9.29 (d, *J* = 2.3 Hz, 1H, 6-H), 9.21 (d, *J* = 2.0 Hz, 1H, 2-H), 8.66 (t, *J* = 5.6 Hz, 1H, 12-*N*-H), 8.61 (pseudo-t, *J* = 2.2 Hz, 1H, 4-H), 8.39 (s, br, 1H, 7-*N*-H), 8.12–8.06 (m, 2H, 10-H), 8.06–7.99 (m, 2H, 9-H), 3.32–3.23 (m, 2H, 13-H), 1.62–1.48 (m, 2H, 14-H), 1.43–1.29 (m, 2H, 15-H), 0.92 (t, *J* = 7.3 Hz, 3H, 16-H); **^{13}C NMR, DEPT135, HSQC, HMBC (101 MHz, DMSO-d_6):** δ (ppm) = 165.5 (7), 165.1 (12), 153.0 (11), 151.1 (2), 148.5 (6), 146.8 (5), 137.6 (8), 130.5 (3), 128.5 (10), 125.7 (4), 122.6 (9), 38.9 (13), 31.1 (14), 19.6 (15), 13.6 (16); **IR (ATR):** *v* (cm^{-1}) = 3375, 3320, 3116, 2957, 2870, 1680, 1630, 1530, 1401, 1300, 1132; **ESI-HRMS:** calcd. for $[C_{17}H_{19}N_5O_2+H]^+$ 325.1539, found 325.1548; **Comp. Purity (220 nm):** 100 %.

(*E*)-5-{[4-(decanamidomethyl)phenyl]diazenyl}nicotinamide (**15f**)

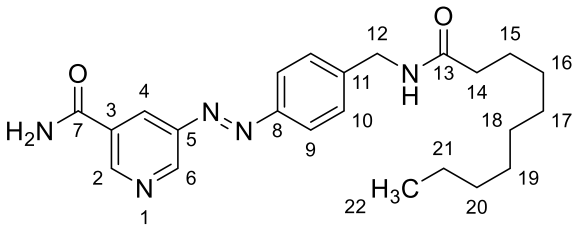

Synthesis was conducted according to the general procedure for the synthesis of nicotinamides from methyl nicotinates using **14d** (100 mg, 0.24 mmol, 1.0 equiv.). The product was obtained as yellow solid (100 mg, 0.24 mmol, 100 %): R_f = 0.52 (EtOAc/MeOH 95:5); mp: 259.5 °C; **^{1}H NMR, H,H-COSY (400 MHz, DMSO-*d$_6$*):** δ (ppm) = 9.23 (d, *J* = 2.2 Hz, 1H, 6-H), 9.18 (d, *J* = 1.9 Hz, 1H, 2-H), 8.57 (pseudo-t, *J* = 2.1 Hz, 1H, 4-H), 8.43 (t, *J* = 5.9 Hz, 1H, 12-*N*-H), 8.37 (s, br, 1H, 7-*N*-H), 7.93 (d, *J* = 8.3 Hz, 1H, 9-H), 7.77 (s, br, 1H, 7-*N*-H), 7.49 (d, *J* = 8.4 Hz, 2H, 10-H), 4.37 (d, *J* = 5.9 Hz, 2H, 12-H), 2.17 (t, *J* = 7.3 Hz, 2H, 14-H), 1.58–1.49 (m, 2H, 15-H), 1.32–1.14 (m, 16 – 21-H), 0.84 (t, *J* = 6.5 Hz, 3H, 22-H); **^{13}C NMR, DEPT135, HSQC, HMBC (101 MHz, DMSO-*d$_6$*):** δ (ppm) = 172.3 (13), 165.7 (7), 150.8 (8), 150.7 (2), 148.3 (6), 146.9 (5), 144.8 (11), 130.6 (3), 128.2 (9), 125.6 (4), 122.9 (10), 41.7 (12), 35.3 (14), 31.3 (20), 29.2–28.3 (16-19), 25.3 (15), 22.1 (21), 13.9 (22); **IR (ATR):** *v* (cm^{-1}) = 3366, 3289, 2920, 2851, 1671, 1640, 1544, 1429, 1402, 1145, 954, 833, 694; **ESI-HRMS:** calcd. for $[C_{23}H_{31}N_5O_2+H]^+$ 409.2478, found 409.2472; **Comp. Purity (220 nm):** 100 % (*E*+*Z*)-**15f**.

(*E*)-5-{[4-(6-hydroxyhex-1-yn-1-yl)phenyl]diazenyl}nicotinamide (**15g**)

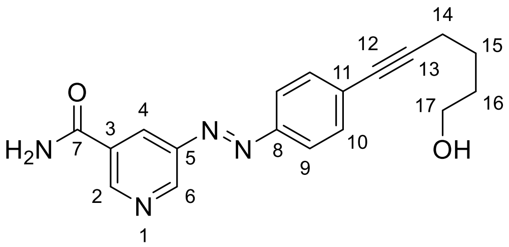

Synthesis was conducted according to the general procedure for the synthesis of nicotinamides from methyl nicotinates using **14h** (50 mg, 0.15 mmol, 1.00 equiv.). The product was obtained as ochre solid (48 mg, 0.15 mmol, 100 %): R_f = 0.15 (n-hexane/EtOAc 2:1); mp: 196.8 °C; **^{1}H NMR, H,H-COSY (400 MHz, DMSO-*d$_6$*):** δ (ppm) = 9.25 (d, *J* = 2.3 Hz, 1H, 6-H), 9.19 (d, *J* = 2.0 Hz, 1H, 2-H), 8.58 (pseudo-t, *J* = 2.2 Hz, 1H, 4-H), 8.38 (s, br, 1H, 7-*N*-H), 7.98–7.90 (m, 2H, 9-H), 7.79 (s, br, 1H, 7-*N*-H), 7.66–7.60 (m, 2H, 10-H), 4.45 (t, *J* = 5.2 Hz, 1H, 17-*O*-H), 3.46 (q, *J* = 6.0 Hz, 2H, 17-H), 2.55–2.48 (m, 2H, 14-H), 1.68–1.51 (m, 4H, 15-H, 16-H); **^{13}C NMR, DEPT135, HSQC, HMBC (101 MHz, DMSO-*d$_6$*):** δ (ppm) = 165.6 (7), 150.9 (8), 150.7 (2), 148.4 (6), 146.9 (5), 132.5 (10), 130.6 (3), 127.3 (11), 125.7 (4), 123.1 (9), 94.7 (13), 80.3 (12), 60.1 (17), 31.7 (16), 24.7 (15), 18.6 (14); **IR (ATR):** *v* (cm^{-1}) = 3409, 1657, 1611, 1383, 1329, 1139, 1028, 1004, 916, 771, 692; **ESI-HRMS:** calcd. For $[C_{18}H_{18}N_4O_2+H]^+$ 322.1430, found 322.1415; **Comp. Purity (220 nm):** 100 %.

(*E*)-5-{[4-(3-hydroxy-3-methylbut-1-yn-1-yl)phenyl]diazenyl}nicotinamide (**15h**)

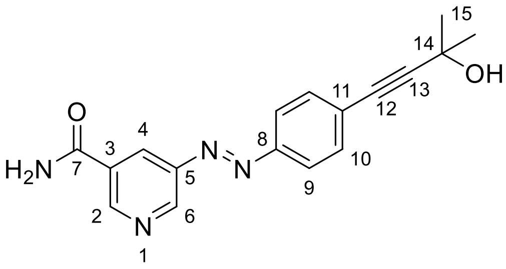

Synthesis was conducted according to the general procedure for the synthesis of nicotinamides from methyl nicotinates using **14g** (82 mg, 0.25 mmol, 1.0 equiv.). The product was obtained as ochre solid (75 mg, 0.24/0,25 mmol, 97 %): R_f = 0.65 (acetone); mp: 223.3 °C; **^{1}H NMR, H,H-COSY (400 MHz, DMSO-*d₆*):** δ (ppm) = 9.26 (d, *J* = 2.3 Hz, 1H, 6-H), 9.20 (d, *J* = 2.0 Hz, 1H, 2-H), 8.58 (pseudo-t, *J* = 2.2 Hz, 1H, 4-H), 8.38 (s, br, 1H, 7-*N*-H), 8.00–7.91 (m, 2H, 9-H), 7.80 (s, br, 1H, 7-*N*-H), 7.72–7.59 (m, 2H, 10-H), 5.85 (s, 1H, 14-O-H), 1.50 (s, 6H, 15-H); **^{13}C NMR, DEPT135, HSQC, HMBC (101 MHz, DMSO-*d₆*):** δ (ppm) = 165.5 (7), 150.9 (2), 150.8 (8), 148.4 (6), 146.8 (5), 132.4 (10), 130.5 (3), 126.5 (11), 125.6 (4), 123.1 (9), 99.5 (13), 79.9 (12), 63.6 (14), 31.4 (15); **IR (ATR):** *v* (cm^{-1}) = 3378, 3190, 1676, 1630, 1593, 1386, 1271, 1168, 1157, 899, 844; **ESI-HRMS:** calcd. For $[C_{17}H_{16}N_4O_2+H]^+$ 308.1273, found 308.1261; **Comp. Purity (220 nm):** 100 %.

Methyl 5-[(3-nitrobenzyl)oxy]nicotinate (**17**)

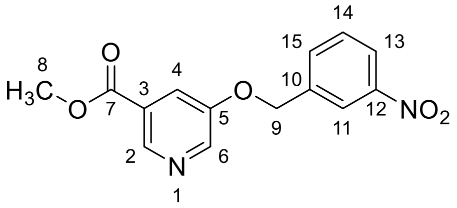

To a solution of 3-nitrobenzylbromide (4.66 g, 21.55 mmol, 1.1 equiv.) and **16** (3.00 g, 19.59 mmol, 1.0 equiv.) in DMF_{abs} (40 mL) Cs_2CO_3 (12.77 g, 39.18 mmol, 2.0 equiv.) was added in one portion. The reaction mixture was stirred for 4 h at room temperature. Another 0.5 equivalents of 3-nitrobenzylbromide (2.12 g, 9.80 mmol) were added to the reaction mixture prior to stirring over night at room temperature. The reaction mixture was partitioned between water (100 mL) and EtOAc (100 mL) and the organic phase washed successively with sat. aq. Na_2CO_3-solution (3 × 40 mL), water (3 × 40 mL) and sat. aq. NaCl-solution (40 mL). It was dried over $MgSO_4$, filtrated and freed from solvent under reduced pressure. The residue was purified by silica gel column chromatography (DCM/EtOAc 3:1) and successive recrystallization (cyclohexane/EtOAc 2:1). The product was obtained as brownish solid (2.33 g, 8.08 mmol, 41 %): R_f = 0.67 (DCM/EtOAc 1:1); mp: 134.2 °C; **^{1}H NMR, H,H-COSY (400 MHz, DMSO-*d₆*):** δ (ppm) = 8.73 (d, *J* = 1.5 Hz, 1H, 2-H), 8.66 (d, *J* = 2.9 Hz, 1H, 6-H), 8.38–8.35 (m, 1H, 11-H), 8.23 (dd, 1H, *J* = 8.2, 2.4, 1.0 Hz, 1H, 13-H), 7.98–7.94 (m, 1H, 15-H), 7.91 (dd, *J* = 2.9, 1.5 Hz, 1H, 4-H), 7.73 (pseudo-t, *J* = 7.9 Hz, 1H, 14-H), 5.44 (s, 2H, 9-H), 3.90 (s, 3H, 8-H); **^{13}C NMR, DEPT135, HSQC, HMBC (101 MHz, DMSO-*d₆*):** δ (ppm) = 165.0 (7), 154.1 (5), 157.8 (12), 142.7 (6), 142.4 (2), 138.4 (10), 134.2 (15), 130.1 (14), 126.1 (3), 123.0 (13), 122.2 (11), 121,0 (4), 68.5 (9), 52.5 (8); **IR (ATR):** *v* (cm^{-1}) = 3060, 2956, 1720, 1587, 1526, 1462, 1429, 1351, 1303, 1038, 765.

Methyl 5-[(3-aminobenzyl)oxy]nicotinate (**18**)

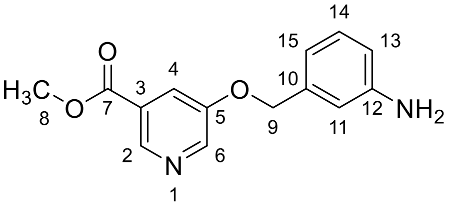

An aqueous suspension of Raney-Ni (500 µL) was added to a solution of **17** (500 mg, 1.73 mmol, 1.0 equiv.) in THF (20 mL). The reaction mixture was set under a hydrogen atmosphere and stirred vigorously at room temperature for 4 h. The catalyst was removed by filtration through a pad of celite® and the filtrate freed from solvent under reduced pressure. The residue was purified by silica gel column chromatography (DCM/EtOAc 1:1), which yielded **18** as colourless solid (381 mg, 1.48 mmol, 85 %): R_f = 0.42 (DCM/EtOAc 1:1); mp: 170.0 °C; **^{1}H NMR, H,H-COSY (400 MHz, DMSO-*d*$_6$):** δ (ppm) = 8.69 (d, *J* = 1.5 Hz, 1H, 2-H), 8.57 (d, *J* = 2.8 Hz, 1H, 6-H), 7.81 (dd, *J* = 2.8, 1.5 Hz, 1H, 4-H), 7.03 (pseudo-t, *J* = 7.7 Hz, 1H, 14-H), 8.38–8.35 (m, 1H, 11-H), 6.64 (pseudo-t, *J* = 1.7 Hz, 1H, 11-H), 6.60–6.55 (m, 1H, 15-H), 6.52 (ddd, *J* = 8.0, 2.2, 0.9 Hz, 1H, 13-H), 5.13 (s, br, 2H, 12-*N*-H), 5.12 (s, 2H, 9-H), 3.89 (s, 3H, 8-H); **^{13}C NMR, DEPT135, HSQC, HMBC (101 MHz, DMSO-*d*$_6$):** δ (ppm) = 165.0 (7), 154.1 (5), 148.8 (12), 142.7 (6), 142.0 (2), 136.5 (10), 128.9 (14), 126.1 (3), 120.8 (4), 114.9 (13), 113.6 (15), 112.7 (11), 70.15 (9), 52.5 (8); **IR (ATR):** *v* (cm^{-1}) = 3421, 3327, 3223, 1710, 1583, 1304, 1237, 1109, 764, 685, 628.

Methyl (*E*)-5-[(3-{[4-(benzyloxy)phenyl]diazenyl}benzyl)oxy]nicotinate (**19a**)

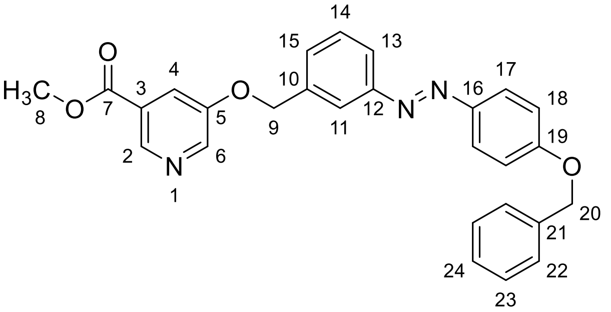

A solution of **18** (358 mg, 1.39 mmol, 1.0 equiv.) in DCM (30 mL) was cooled in an ice bath and treated with a solution of oxone® (1.71 g, 2.77 mmol, 2.0 equiv.) in water (15 mL). The resulting biphasic mixture was vigorously stirred until TLC indicated complete transformation of **18** (2 h). The aqueous phase was separated and extracted with DCM (2 × 15 mL). All organic phases were combined and washed with successively with an aq. HCl-solution (1 M, 15 mL), sat. aq. $NaHCO_3$-solution (15 mL), water (15 mL) and sat. aq. NaCl-solution (15 mL). It was dried over $MgSO_4$, filtrated and freed from solvent under reduced pressure. The residue was set under an argon atmosphere, taken up in glacial acetic acid (10 mL) and treated with one portion of 4-(benzyloxy)aniline (249 mg, 1.25 mmol, 0.9 equiv.). After four days of stirring at room temperature, the reaction mixture was poured onto ice-cooled sat. aq. $NaHCO_3$-solution (200 mL) and extracted with EtOAc (3 × 100 mL). The combined organic extracts were washed successively with water (3 × 50 mL) and sat. aq. NaCl-solution (2 × 50 mL) and dried over $MgSO_4$. After filtration, the solvent was evaporated under reduced pressure and the residue purified by silica gel column chromatography (cyclohexane/THF 2.5:1). The title compound

was obtained as yellow solid (223 mg, 0.49 mmol, 35 %): R_f = 0.38 (cyclohexane/THF 2.5:1); mp: 145.9 °C; **^{1}H NMR, H,H-COSY (400 MHz, DMSO-*d$_6$*):** δ (ppm) = 8.72 (d, *J* = 1.6 Hz, 1H, 2-H), 8.66 (d, *J* = 2.9 Hz, 1H, 6-H), 7.96 (s, br, 1H, 11-H), 7.94–7.89 (m, 3H, 4-H, 17-H), 7.84 (dt, *J* = 6.8, 2.1 Hz, 1H, 13-H), 7.67–7.59 (m, 2H, 14-H, 15-H), 7.52–7.47 (m, 2H, 22-H), 7.45–7.39 (m, 2H, 23-H), 7.39–7.33 (m, 1H, 24-H), 7.26–7.20 (m, 1H, 18-H), 5.41 (s, 2H, 9-H), 5.24 (s, 2H, 20-H), 3.89 (s, 3H, 8-H); **^{13}C NMR, DEPT135, HSQC, HMBC (101 MHz, DMSO-*d$_6$*):** δ (ppm) = 165.0 (7), 161.2 (19), 154.3 (5), 152.1 (12), 146.2 (16), 142.7 (6), 142.2 (2), 137.5 (10), 136.4 (21), 129.9 (14/15), 129.6 (14/15), 128.4 (23), 127.9 (24), 127.7 (22), 126.1 (3), 124.6 (17), 122.2 (13), 121.0 (11), 120.9 (4), 115.4 (18), 69.6 (20), 69.3 (9), 52.5 (8); **IR (ATR):** *v* (cm^{-1}) = 3079, 2950, 1720, 1599, 1578, 1499, 1299, 1241, 1222, 1102, 1018.

Methyl (*E*)-5-({3-[(4-fluorophenyl)diazenyl]benzyl}oxy)nicotinate (**19b**)

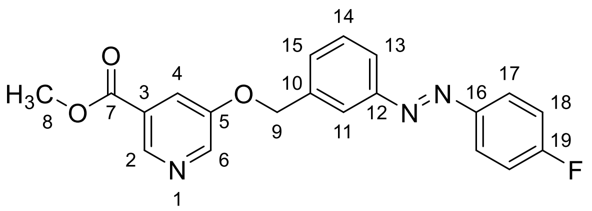

A solution of 4-fluoroaniline (142 mg, 1.28 mmol, 1.2 equiv.) in 15 mL DCM was converted to the nitroso compound using a solution of oxone® (1.58 g, 2.56 mmol, 2.2 equiv.) in water (15 mL). After 14 h of vigorous stirring at room temperature, the aqueous phase was separated and extracted with DCM (2 × 15 mL). The combined organic phases were washed successively with an aq. HCl-solution (1 M, 30 mL), sat. aq. $NaHCO_3$-solution (30 mL), water (30 mL) and sat. aq. NaCl-solution (30 mL), dried over $MgSO_4$, filtrated and freed from solvent under reduced pressure. The residue was set under an argon atmosphere and taken up in glacial acetic acid (15 mL). After addition of **18** (300 mg, 1.16 mmol, 1.0 equiv.) the resulting mixture was reacted at room temperature for 7 days, then poured onto ice-cooled sat. aq. $NaHCO_3$-solution (100 mL) and extracted with EtOAc (3 × 50 mL). The combined organic extracts were washed successively with water (50 mL) and sat. aq. NaCl-solution (50 mL) and dried over $MgSO_4$. After filtration, the solvent was evaporated under reduced pressure and the crude product purified by silica gel column chromatography (cyclohexane/THF 2.5:1), which yielded the title compound as yellow solid (123 mg, 0.34 mmol, 34 %): R_f = 0.42 (cyclohexane/THF 2.5:1); mp: 117.2 °C; **^{1}H NMR, H,H-COSY (400 MHz, DMSO-*d$_6$*):** δ (ppm) = 8.73 (d, *J* = 1.6 Hz, 1H, 2-H), 8.66 (d, *J* = 2.9 Hz, 1H, 6-H), 8.02–7.96 (m, 3H, 11-H, 17-H), 7.91 (dd, *J* = 2.9, 1.7 Hz, 1H, 4-H), 7.89 (pseudo-dt, *J* = 7.5, 1.7 Hz, 1H, 13-H), 7.70 (pseudo-dt, *J* = 7.5, 1.5 Hz, 1H, 15-H), 7.65 (pseudo-t, *J* = 7.6 Hz, 1H, 14-H), 7.49–7.42 (m, 2H, 18-H), 5.42 (s, 2H, 9-H), 3.90 (s, 3H, 8-H); **^{13}C NMR, DEPT135, HSQC, HMBC (101 MHz, DMSO-*d$_6$*):** δ (ppm) = 165.0 (7), 163.8 (d, *J* = 250.2 Hz, 19), 154.3 (5), 151.8 (12), 148.6 (16), 142.7 (6), 142.2 (2), 137.7 (10), 130.6 (15), 129.7 (14), 126.1 (3), 124.9 (d, *J* = 9.3 Hz, 17), 122.5 (13), 121.3 (11), 120.9 (4), 116.4 (d, *J* = 23.1 Hz, 18), 69.2 (9), 52.5 (8); **IR (ATR):** *v* (cm^{-1}) = 1725, 1593, 1580, 1501, 1429, 1320, 1306, 1249, 1221, 1105, 837.

Methyl (*E*)-5-({3-[(4-ethynylphenyl)diazenyl]benzyl}oxy)nicotinate (**19c**)

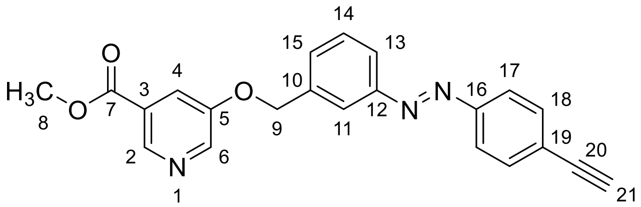

Synthesis was conducted according the procedure of **19b** using 4-ethynylaniline (152 mg, 1.30 mmol, 1.0 equiv.) in DCM (10 mL) and oxone® (1.60 g, 2.60 mmol, 2.0 equiv.) in water (15 mL). The crude nitroso compound was reacted with **18** (114 mg, 0.97 mmol, 0.7 equiv.) in glacial acetic acid (5 mL) by stirring under an argon atmosphere for 4 days at room temperature. Purification of the crude product by silica gel column chromatography (cyclohexane/THF 2.5:1) yielded the title compound as yellow solid (139 mg, 0.37 mmol, 42 %): R_f = 0.22 (cyclohexane/THF 2.5:1); mp: 153.6 °C; **^{1}H NMR, H,H-COSY (400 MHz, DMSO-*d*$_6$):** δ (ppm) = 8.73 (d, *J* = 1.6 Hz, 1H, 2-H), 8.67 (d, *J* = 2.9 Hz, 1H, 6-H), 8.02 (s, br, 1H, 11-H), 7.95–7.87 (m, 4H, 4-H, 13-H, 17-H), 7.75–7.69 (m, 3H, 15-H, 18-H), 7.66 (pseudo-t, *J* = 7.6 Hz, 1H, 14-H), 5.42 (s, 2H, 9-H), 4.47 (s, 1H, 21-H), 3.90 (s, 3H, 8-H); **^{13}C NMR, DEPT135, HSQC, HMBC (101 MHz, DMSO-*d*$_6$):** δ (ppm) = 165.0 (7), 154.2 (5), 151.9 (12), 151.3 (16), 142.7 (6), 142.3 (2), 137.8 (10), 132.9 (18), 130.9 (15), 129.7 (14), 126.1 (3), 124.7 (19), 122.8 (17), 122.7 (13), 121.4 (11), 120.9 (4), 83.5 (21), 82.9 (20), 69.2 (9), 52.5 (8); **IR (ATR):** *ν* (cm^{-1}) = 3261, 1724, 1579, 1427, 1247, 1230, 1104, 1011, 842, 791, 634.

Methyl (*E*)-5-({3-[(4-morpholinophenyl)diazenyl]benzyl}oxy)nicotinate (**19d**)

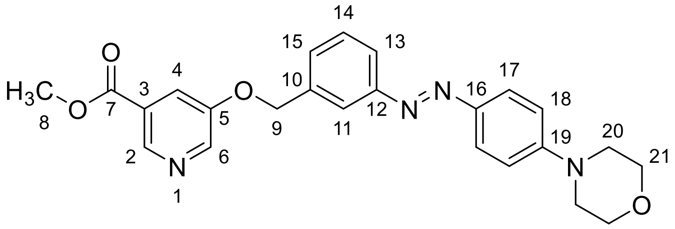

Synthesis was conducted according the procedure of **19a** using **18** (358 mg, 1.39 mmol, 1.0 equiv.) in DCM (10 mL) and oxone® (1.71 g, 2.77 mmol, 2.0 equiv.) in water (15 mL). The crude nitroso compound was reacted with 4-morpholinoaniline (173 mg, 0.97 mmol, 0.7 equiv.) in glacial acetic acid (5 mL) by stirring under an argon atmosphere over night at room temperature. Purification of the crude product by silica gel column chromatography (cyclohexane/THF 1:1) yielded the title compound as orange solid (97 mg, 0.22 mmol, 23 %): R_f = 0.43 (cyclohexane/THF 1:1); mp: 140.1 °C; **^{1}H NMR, H,H-COSY (400 MHz, DMSO-*d*$_6$):** δ (ppm) = 8.72 (d, *J* = 1.6 Hz, 1H, 2-H), 8.66 (d, *J* = 2.9 Hz, 1H, 6-H), 7.94–7.89 (m, 2H, 4-H, 11-H), 7.85–7.81 (m, 2H, 17-H), 7.81–7.77 (m, 2H, 14-H, 15-H), 7.12–7.08 (m, 2H, 18-H), 5.39 (s, 2H, 9-H), 3.89 (s, 3H, 8-H), 3.79–3.73 (m, 4H, 20-H), 3.35–3.29 (m, 4H, 21-H); **^{13}C NMR, DEPT135, HSQC, HMBC (101 MHz, DMSO-*d*$_6$):** δ (ppm) = 165.0 (7), 154.3 (5), 153.2 (19), 152.3 (12), 144.1 (16), 142.7 (6), 142.2 (2), 137.4 (10), 129.5 (14/15), 129.2 (14/15), 126.1 (3), 124.4 (17), 122.0 (13), 120.9 (4/11), 120.8 (4/11), 113.8 (18), 69.4 (9), 65.8 (20), 52.5 (8), 47.0 (21); **IR (ATR):** *ν* (cm^{-1}) = 2832, 1719, 1601, 1586, 1297, 1161, 1120, 822, 770, 694, 589.

(*E*)-5-[(3-{[4-(benzyloxy)phenyl]diazenyl}benzyl)oxy]nicotinamide (**20a**)

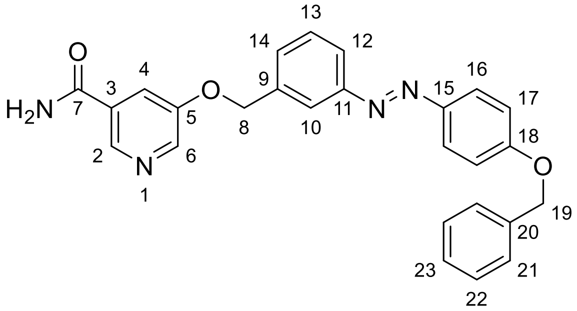

Synthesis was conducted according to the general procedure for the synthesis of nicotinamides from methyl nicotinates using **19a** (100 mg, 0.23 mmol, 1.00 equiv.). The product was obtained as yellow solid (103 mg, 0.23 mmol, 100 %): R_f = 0.57 (EtOAc/MeOH 95:5); mp: 183.9 °C; **^{1}H NMR, H,H-COSY (400 MHz, DMSO-*d₆*):** δ (ppm) = 8.68 (d, *J* = 1.7 Hz, 1H, 2-H), 8.54 (d, *J* = 2.8 Hz, 1H, 6-H), 8.15 (s, br, 1H, *N*-H), 7.95 (s, br, 1H, 10-H), 7.94–7.89 (m, 3H, 4-H, 16-H), 7.84 (pseudo-dt, *J* = 6.4, 2.3 Hz, 1H, 12-H), 7.67–7.58 (m, 3H, 13-H, 14-H, *N*-H), 7.52–7.48 (m, 2H, 21-H), 7.45–7.40 (m, 2H, 22-H), 7.38–7.33 (m, 1H, 23-H), 7.26–7.20 (m, 2H, 17-H), 5.37 (s, 2H, 8-H), 5.24 (s, 2H, 19-H); **^{13}C NMR, DEPT135, HSQC, HMBC (101 MHz, DMSO-d_6):** δ (ppm) = 166.0 (7), 161.2 (18), 154.2 (5), 152.1 (11), 146.2 (15), 141.0 (2), 140.5 (6), 137.7 (9), 136.4 (20), 130.3 (3), 129.9 (13/14), 129.6 (13/14), 128.4 (21), 127.9 (23), 127.7 (22), 124.6 (16), 122.2 (12), 121.0 (10), 120.0 (4), 115.4 (17), 69.6 (8), 69.2 (19); **IR (ATR):** *v* (cm^{-1}) = 3366, 3138, 1680, 1596, 1579, 1381, 1234, 1151, 1008, 843, 698; **ESI-HRMS:** calcd. For $[C_{26}H_{22}N_4O_3+H]^+$ 438.1692, found 438.1682; **Comp. Purity (220 nm):** 100 % (*E*+*Z*)-**20a**.

(*E*)-5-({3-[(4-fluorophenyl)diazenyl]benzyl}oxy)nicotinamide (**20b**)

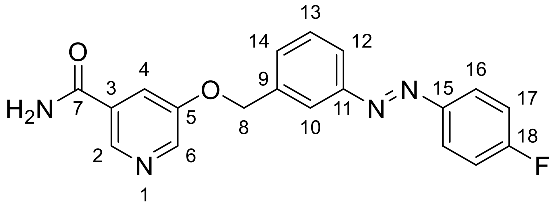

Synthesis was conducted according to the general procedure for the synthesis of nicotinamides from methyl nicotinates using **19b** (100 mg, 0.27 mmol, 1.00 equiv.). The product was obtained as yellow solid (93 mg, 0.27 mmol, 100 %): R_f = 0.58 (EtOAc/MeOH 95:5); mp: 202.1 °C; **^{1}H NMR, H,H-COSY (400 MHz, DMSO-d_6):** δ (ppm) = 8.68 (d, *J* = 1.6 Hz, 1H, 2-H), 8.54 (d, *J* = 2.8 Hz, 1H, 6-H), 8.16 (s, br, 1H, *N*-H), 8.04–7.95 (m, 3H, 10-H, 16-H), 7.94–7.86 (m, 2H, 4-H, 12-H), 7.73–7.59 (m, 3H, 13-H, 14-H, *N*-H), 7.49–7.41 (m, 2H, 17-H), 5.39 (s, 2H, 8-H); **^{13}C NMR, DEPT135, HSQC, HMBC (101 MHz, DMSO-d_6):** δ (ppm) = 166.1 (7), 163.9 (d, *J* = 250.3 Hz, 18), 154.2 (5), 151.9 (11), 148.6 (15), 141.1 (), 141.0 (), 137.9 (9), 130.7 (14), 130.4 (3), 129.7 (13), 125.0 (d, *J* = 9.3 Hz, 16), 122.6 (12), 121.3 (10), 120.1 (4), 116.5 (d, *J* = 23.1 Hz, 17), 69.2 (8); **IR (ATR):** *v* (cm^{-1}) = 3412, 3166, 1698, 1592, 1503, 1379, 1274, 1226, 1142, 841, 686; **ESI-HRMS:** calcd. For $[C_{19}H_{15}N_4O_2F+H]^+$ 350.1179, found 350.1163; **Comp. Purity (220 nm):** 100 % (*E*+*Z*)-**17b**.

(*E*)-5-({3-[(4-ethynylphenyl)diazenyl]benzyl}oxy)nicotinamide (**20c**)

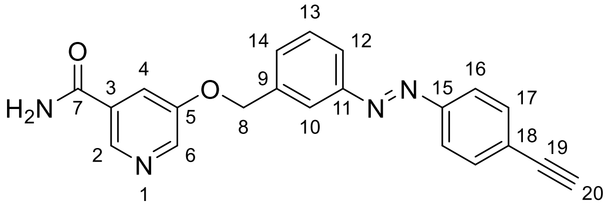

Synthesis was conducted according to the general procedure for the synthesis of nicotinamides from methyl nicotinates using **19c** (119 mg, 0.32 mmol, 1.00 equiv.). The product was obtained as yellow solid (104 mg, 0.29 mmol, 91 %): R_f = 0.58 (EtOAc/MeOH 95:5); mp: 203.1 °C; **^{1}H NMR, H,H-COSY (400 MHz, DMSO-*d₆*):** δ (ppm) = 8.68 (s, 1H, 2-H), 8.54 (s, 1H, 6-H), 8.16 (s, br, 1H, *N*-H), 8.02 (s, br, 1H, 10-H), 7.96–7.86 (m, 4H, 4-H, 12-H, 16-H), 7.67–7.59 (m, 5H, 13-H, 14-H, 17-H, *N*-H), 5.39 (s, 2H, 8-H), 4.47 (s, 1H, 20-H); **^{13}C NMR, DEPT135, HSQC, HMBC (101 MHz, DMSO-*d₆*):** δ (ppm) = 166.0 (7), 154.1 (5), 151.9 (11), 151.3 (15), 142.1 (2), 140.6 (6), 137.9 (9), 132.9 (17), 130.9 (14), 130.3 (3), 129.7 (13), 124.7 (18), 122.8 (16), 122.7 (12), 121.4 (10), 120.0 (4), 83.5 (20), 82.9 (19), 69.1 (8); **IR (ATR):** *v* (cm^{-1}) = 3410, 3269, 1695, 1631, 1588, 1573, 1432, 1390, 1273, 1177, 685; **ESI-HRMS:** calcd. For $[C_{21}H_{16}N_4O_2+H]^+$ 356.1273, found 356.1265; **Comp. Purity (220 nm):** 97 % (*E*+*Z*)-**17c**.

(*E*)-5-({3-[(4-morpholinophenyl)diazenyl]benzyl}oxy)nicotinamide (**20d**)

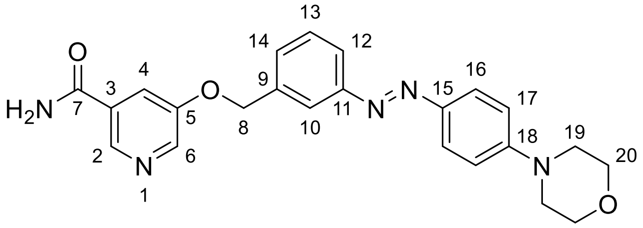

Synthesis was conducted according to the general procedure for the synthesis of nicotinamides from methyl nicotinates using **19d** (68 mg, 0.16 mmol, 1.00 equiv.). The product was obtained as orange solid (67 mg, 0.16 mmol, 100 %): R_f = 0.48 (EtOAc/MeOH 95:5); mp: 202.1 °C; **^{1}H NMR, H,H-COSY (400 MHz, DMSO-*d₆*):** δ (ppm) = 8.67 (d, *J* = 1.7 Hz, 1H, 2-H), 8.53 (d, *J* = 2.8 Hz, 1H, 6-H), 8.14 (s, br, 1H, *N*-H), 7.95–7.88 (m, 2H, 4-H, 10-H), 7.83 (d, *J* = 9.1 Hz, 2H, 16-H), 7.81–7.76 (m, 1H, 12-H), 7.63–7.57 (m, 3H, 13-H, 14-H, *N*-H), 7.10 (d, *J* = 9.2 Hz, 2H, 17-H), 5.36 (s, 2H, 8-H), 3.78–3.73 (m, 4H, 19-H), 3.36–3.30 (m, 4H, 20-H); **^{13}C NMR, DEPT135, HSQC, HMBC (101 MHz, DMSO-*d₆*):** δ (ppm) = 166.0 (7), 154.2 (5), 153.2 (18), 152.3 (11), 144.1 (15), 141.0 (2), 140.5 (6), 137.6 (9), 130.3 (3), 129.4 (13/14), 129.2 (13/14), 124.4 (16), 122.0 (12), 120.8 (10), 120.0 (4), 113.8 (17), 69.3 (8), 65.8 (19), 47.0 (20); **IR (ATR):** *v* (cm^{-1}) = 3195, 1679, 1596, 1566, 1505, 1445, 1374, 1235, 11544, 1112, 926; **ESI-HRMS:** calcd. For $[C_{23}H_{23}N_5O_3+H]^+$ 417.1801, found 417.1792; **Comp. Purity (220 nm):** 100 % (*E*+*Z*)-**17d**.

Irradiation Setup

Investigation of the photochromic properties was conducted at room temperature under total exclusion of daylight, using ruby light (630 nm) in a dark room. For all photochemical experiments, spectrophotometric grade solvents and stoppered quartz glass cuvettes (114-QS, Hellma Analytics) were used. UV irradiation was conducted in a Vilber-Lourmat Bio-Link 254 Crosslinker equipped with six Vilber-Lourmat T8-L lamps (8 W, 365 nm, Figure S1).

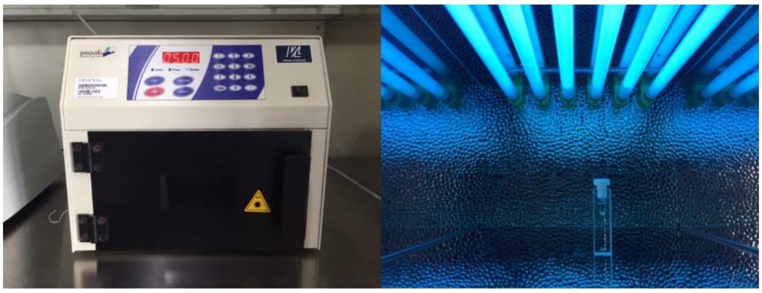

Figure S1: Setup for UV radiation.

As visible light source 1.5 m of a 11 W·m^{-1} RGB-LED strip (Paulmann FlexLED 3D Set, 63 LEDs) wound in a hollow glass cylinder (h = 12 cm, ø 10 cm) was applied. The quartz glass cuvette was put on a slice of aluminium foil and the cylinder placed over it for irradiation (Figure S2).

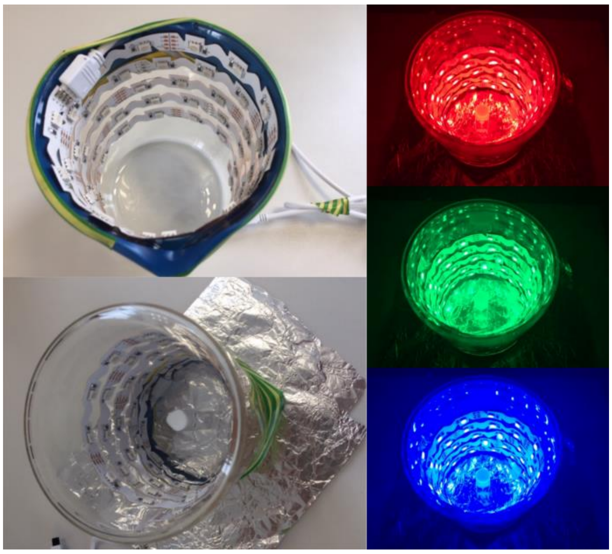

Figure S2: Setup for irradiation with visible light of 630 nm (red), 500 nm (green) or 452 nm (blue).

UV/Vis Spectra and Half-Life of Thermal Isomerization

The half-life of the thermal *Z*→*E* isomerization after 5 minutes of 365 nm irradiation was determined by UV/Vis-spectroscopy. Therefore, solutions of the respective compounds (50 µM) in 5%, 50% or 90% DMSO in enzyme assay buffer (v/v) were prepared. An initial spectrum of the thermal equilibrium was recorded. After 5 minutes of irradiation with 365 nm the increase of absorbance at the thermal equilibrium maximum (λ^{Δ}_{max}) was recorded as exemplified by compound **15c** (Figure S3). Absorbance at $\lambda = \lambda^{\Delta}_{max}$ was plotted as a function of time. Non-linear regression yielded the half-life of the thermal *Z*→*E* isomerization (Table S1). For extrapolation of the reaction kinetics, the plateau value was set to the initial absorbance at $\lambda=\lambda^{\Delta}_{max}$. The following equation was used for non-linear regression:

$$y = (y_0 - Plateau)e^{-kx} + Plateau$$

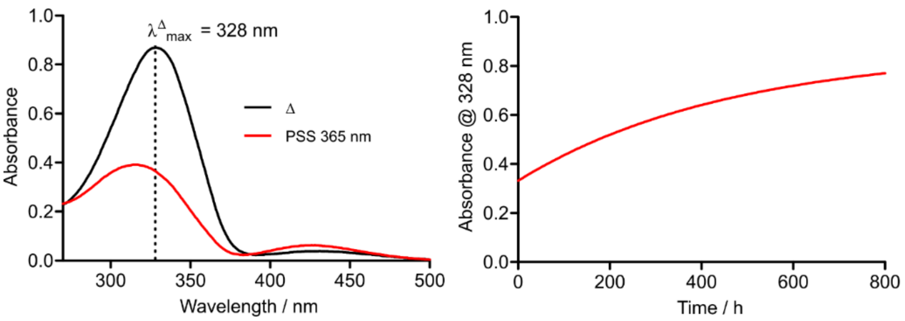

Figure S3: Left: UV-Vis spectrum of **15c** (50 µM) in assay buffer (5% DMSO, v/v) at the thermal equilibrium (Δ) and the photostationary state (PSS) after UV irradiation (365 nm). Right: Absorbance of **15c** (50 µm) in assay buffer (5% DMSO, v/v) at λ^{Δ}_{max} = 328 nm after 5 minutes of UV (365 nm) irradiation.

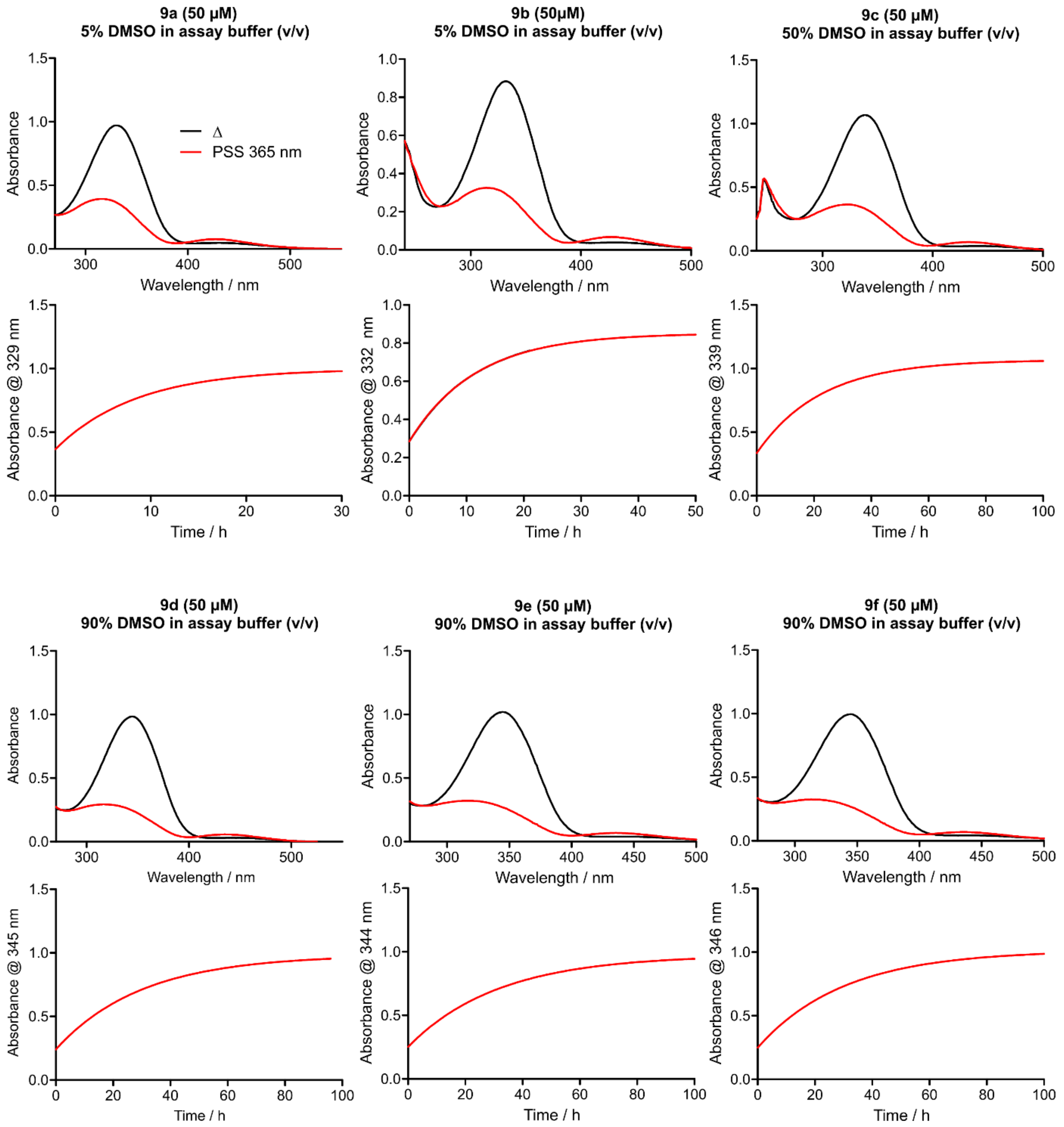
9a (50 µM)
5% DMSO in assay buffer (v/v)
Δ
PSS 365 nm
Absorbance
Wavelength / nm
Absorbance @ 329 nm
Time / h
9b (50µM)
5% DMSO in assay buffer (v/v)
Absorbance @ 332 nm
9c (50 µM)
50% DMSO in assay buffer (v/v)
Absorbance @ 339 nm
9d (50 µM)
90% DMSO in assay buffer (v/v)
Absorbance @ 345 nm
9e (50 µM)
90% DMSO in assay buffer (v/v)
Absorbance @ 344 nm
9f (50 µM)
90% DMSO in assay buffer (v/v)
Absorbance @ 346 nm

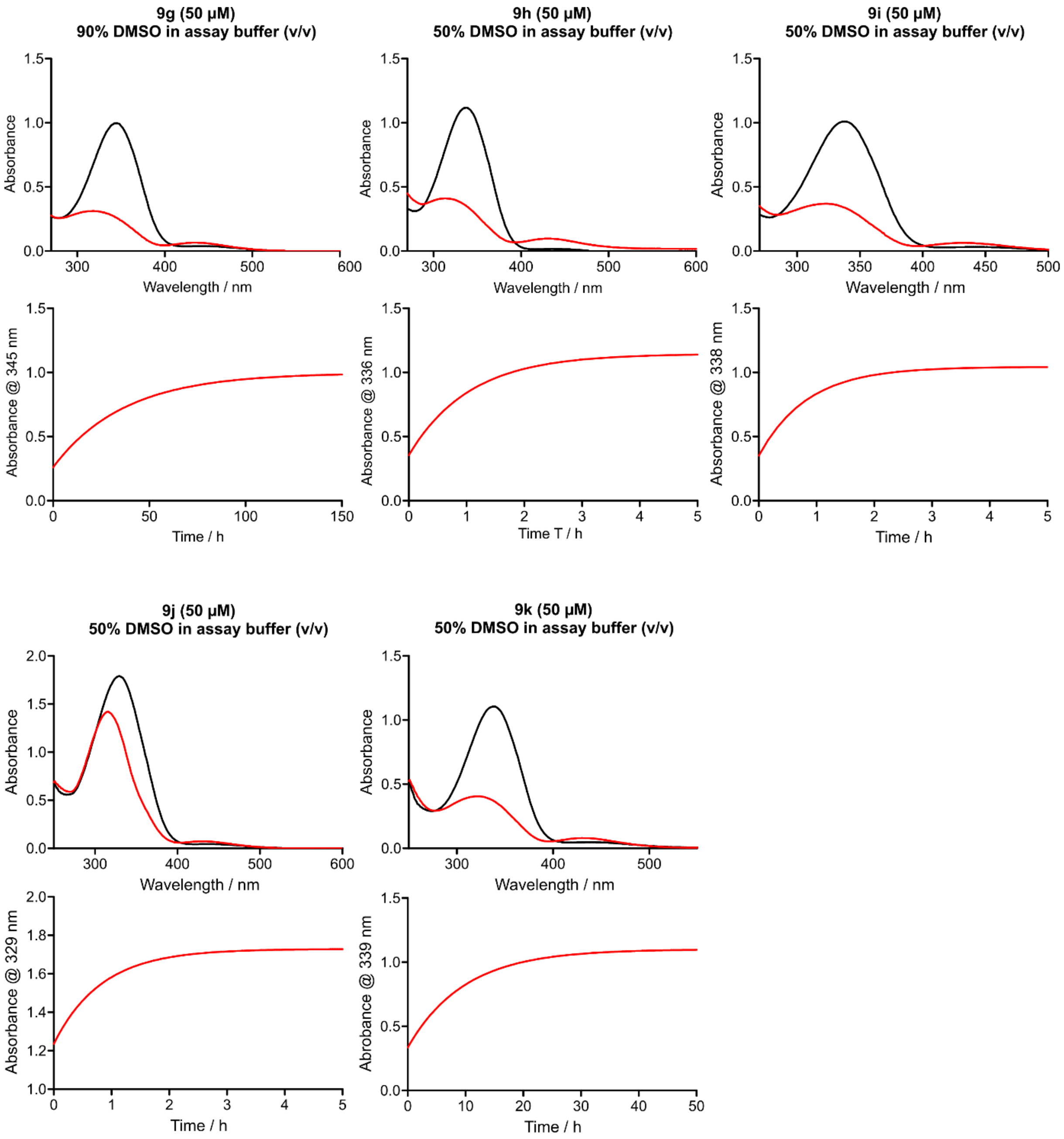

9g (50 µM)
90% DMSO in assay buffer (v/v)
Absorbance
Wavelength / nm
Absorbance @ 345 nm
Time / h
9h (50 µM)
50% DMSO in assay buffer (v/v)
Absorbance
Wavelength / nm
Absorbance @ 336 nm
Time T / h
9i (50 µM)
50% DMSO in assay buffer (v/v)
Absorbance
Wavelength / nm
Absorbance @ 338 nm
Time / h
9j (50 µM)
50% DMSO in assay buffer (v/v)
Absorbance
Wavelength / nm
Absorbance @ 329 nm
Time / h
9k (50 µM)
50% DMSO in assay buffer (v/v)
Absorbance
Wavelength / nm
Abrobance @ 339 nm
Time / h

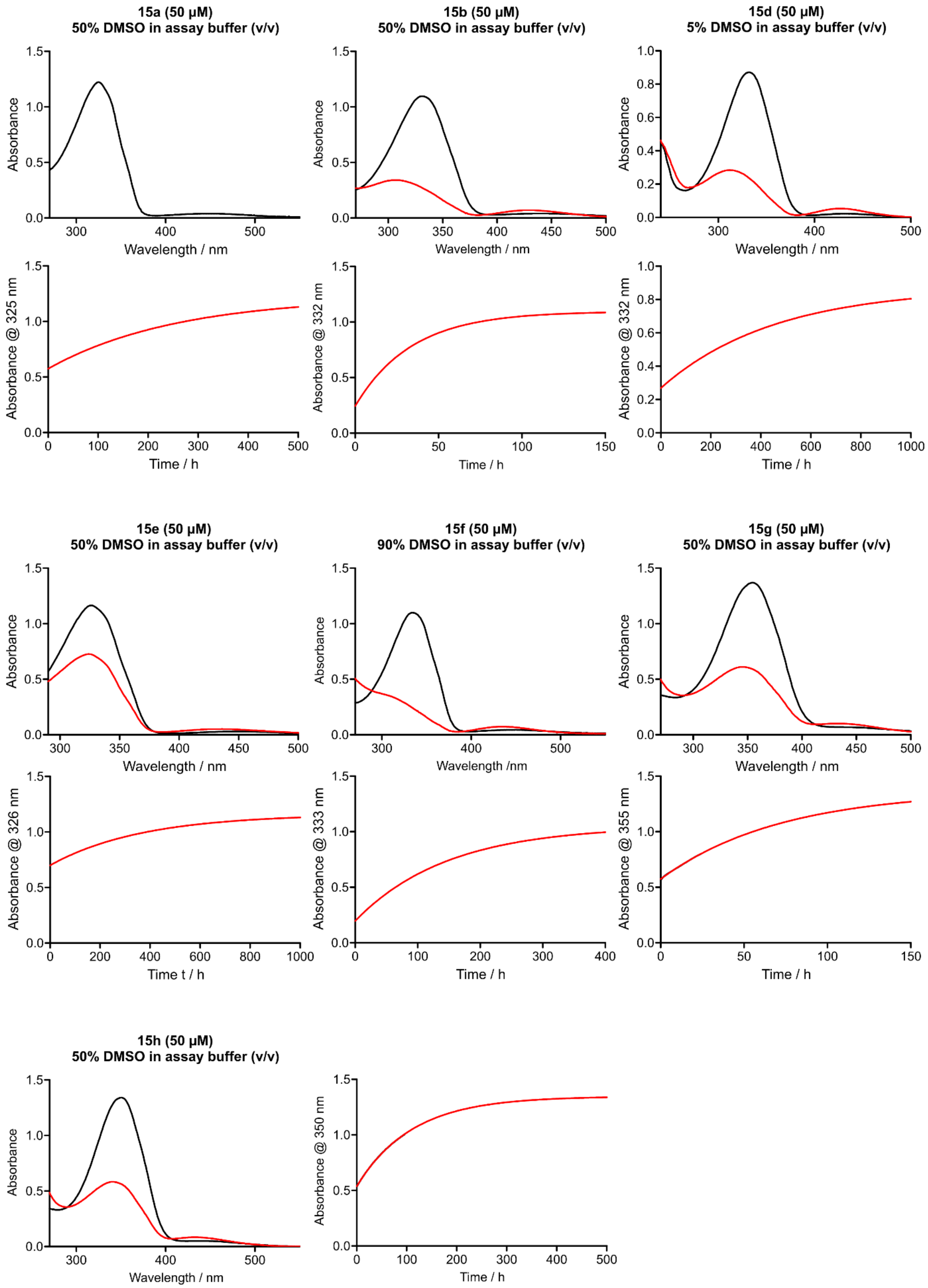

15a (50 µM)
50% DMSO in assay buffer (v/v)
Absorbance
Wavelength / nm
Absorbance @ 325 nm
Time / h
15b (50 µM)
50% DMSO in assay buffer (v/v)
Absorbance
Wavelength / nm
Absorbance @ 332 nm
Time / h
15d (50 µM)
5% DMSO in assay buffer (v/v)
Absorbance
Wavelength / nm
Absorbance @ 332 nm
Time / h
15e (50 µM)
50% DMSO in assay buffer (v/v)
Absorbance
Wavelength / nm
Absorbance @ 326 nm
Time t / h
15f (50 µM)
90% DMSO in assay buffer (v/v)
Absorbance
Wavelength /nm
Absorbance @ 333 nm
Time / h
15g (50 µM)
50% DMSO in assay buffer (v/v)
Absorbance
Wavelength / nm
Absorbance @ 355 nm
Time / h
15h (50 µM)
50% DMSO in assay buffer (v/v)
Absorbance
Wavelength / nm
Absorbance @ 350 nm
Time / h

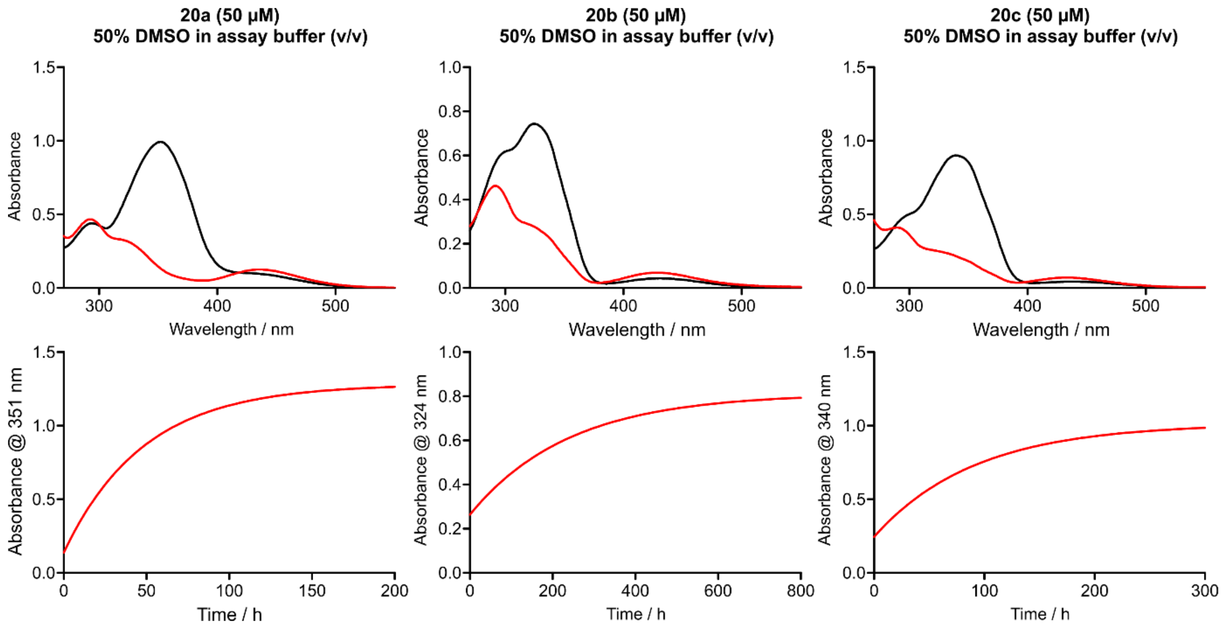

Table S1: Non-linear regression of the thermal Z→E isomerization kinetics of compounds **9a-k**, **15a-h** and **20a-c**.

	y_0	Std. error	Plateau	k	Std. error	$t_{½}$ / h
**9a**	0.3652	2.420e-004	0.9987	0.1176	3.110e-004	5.894
**9b**	0.2831	2.871e-004	0.8520	0.08608	7.387e-005	8.020
**9c**	0.3601	2.009e-003	1.068	0.04300	1.747e-004	16.12
**9d**	0.2436	5.651e-004	0.9900	0.03293	3.670e-005	21.05
**9e**	0.2551	6.036e-004	0.9750	0.03173	4.145e-005	21.85
**9f**	0.2568	8.677e-004	1.014	0.03286	5.594e-005	21.09
**9g**	0.2612	2.875e-004	0.9970	0.02709	1.595e-005	25.59
**9h**	0.3557	1.710e-003	1.146	0.9524	3.599e-003	0.7278
**9i**	0.3513	2.408e-003	1.044	1.193	8.000e-003	0.5811
**9j**	1.236	3.942e-003	1.729	1.212	1.583e-002	0.5720
**9k**	0.3359	9.300e-004	1.101	0.1022	2.426e-004	6.784
**15a**	0.5746	2.993e-004	1.223	0.003905	9.195e-006	177.5
**15b**	0.2439	1.204e-003	1.096	0.02959	1.246e-004	23.43
**15c**	0.3317	7.722e-005	0.8640	0.002173	2.774e-006	319.0
**15d**	0.2657	2.236e-004	0.7238	0.003156	4.807e-005	219.6
**15e**	0.6997	8.215e-005	1.161	0.002728	3.455e-006	254.1
**15f**	0.1967	8.105e-004	1.052	0.006796	2.050e-005	102.0
**15g**	0.5740	6.886e-004	1.369	0.01390	6.812e-005	49.88
**15h**	0.5380	1.681e-002	1.347	0.009060	1.058e-005	76.51
**20a**	0.1380	4.291e-004	1.282	0.02070	6.313e-005	33.49

	y_0	Std. error	Plateau	k	Std. error	$t_{½}$ / h
**20b**	0.2636	1.886e-004	0.8130	0.004182	6.924e-006	165.8
**20c**	0.2447	8.611e-004	1.014	0.01098	2.757e-005	63.10

Photostationary Distribution

Photostationary distribution (PSD) of the respective photoisomers at the PSS (365 nm and 452 nm) was determined *via* analytical HPLC, applying evaporative light scattering detection (ELSD). Methanolic solutions (1 mM) of the respective compounds were injected (15 µL) before and after 5 minutes of 365 nm and 452 nm irradiation, respectively. Isocratic elution using water/methanol mixtures gave complete separation of the isomers (Figure S4). Integration of the peak areas yielded the relative percentages of (*E*)- and (*Z*)-photoisomers.

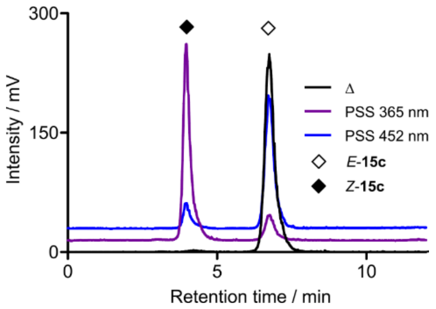

Figure S4: HPLC analysis of **15c** solved in methanol (1 mM) at the thermal equilibrium (Δ) and the photostationary state (PSS) after UV (365 nm) and blue (452 nm) light irradiation. Isocratic elution using a mixture of methanol/water (60% MeOH) gave complete separation of the photoisomers.

Reductive Stability

Glutathione (GSH) is able to reduce certain azobenzenes yielding the respective hydrazines. Therefore, we determined the reductive stability of **15c**, **15f** and **20a** prior to examination of the biological activity in a cell-based assay. The respective azo dyes solved in a mixture of methanol/H_2O (90% methanol (v/v)) were incubated with GSH (5 mM) at ambient temperature for 24 h. HPLC analysis proved stability of the compounds in the given time frame, as the ratio between GSH and both photoisomers remained virtually unaffected (Figure S5, Table S2). Subsequent switching to PSS by 365 nm (purple) and 452 nm (blue) irradiation verified functionality of the photoswitchable azo moiety.

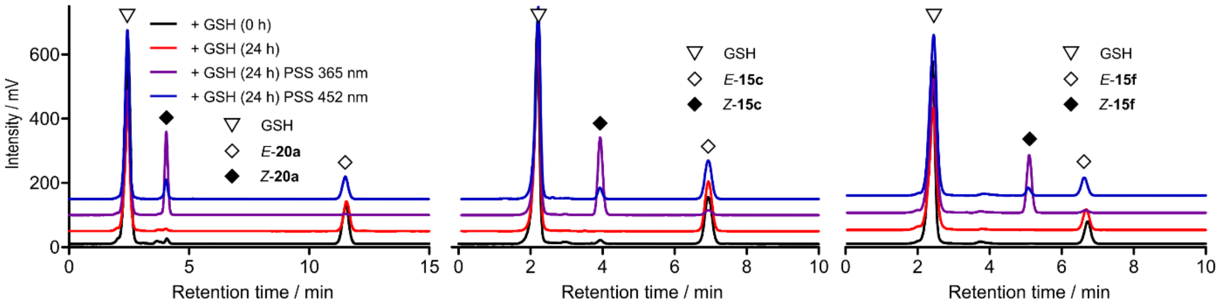

Figure S5: HPLC analysis of **20a** (left; 1 mM), **15c** (middle; 1 mM) and **15f** (right; 1 mM) in the presence of GSH (5 mM) applying evaporative light scattering detection (ELSD). Isocratic elution (Flowrate: 1 mL · min^{-1}) using methanol (solvent B) and water gave complete separation of the compounds: **20a** (80% B), **15c** (65% B), **15f** (80% B).

Table S2: Relative percentages (%) of GSH and the respective photoisomers extracted from the HPLC analysis. Samples were analyzed right after addition of GSH (0 h) and after 24 h of incubation at ambient temperature.

	Incubation time	GSH (%)	*Z*-Isomer (%)	*E*-Isomer (%)	*Z*+*E*-Isomer (%)
**15c**	0 h	76	1	23	24
	24 h	75	0	25	25
	24 h PSS 365 nm	71	27	2	29
	24 h PSS 452 nm	76	4	20	24
**15f**	+ GSH 0 h	87	0	13	13
	+ GSH 24 h	86	0	14	14
	+ GSH 24 h PSS 365 nm	77	22	1	23
	+ GSH 24 h PSS 452 nm	89	2	9	11
**20a**	+ GSH 0 h	77	2	21	23
	+ GSH 24 h	75	2	23	25
	+ GSH 24 h PSS 365 nm	70	29	1	30
	+ GSH 24 h PSS 452 nm	79	7	14	21

Fluorescence-Based Activity Assay (ZMAL-Assay)

The influence of the synthesized compounds on the deacetylase activity of the class I sirtuins Sirt1–3 was determined in a homogenous fluorescence-based enzyme assay, using the artificial substrate Z-(Ac)Lys-AMC (ZMAL). ZMAL is processed by sirtuins yielding Z-Lys-AMC. The latter is subsequently cleaved by tryptic digestion, releasing 7-amino-4-methylcoumarin (AMC), excitation of which leads to a fluorescent read out (λ_{ex}=390 nm; λ_{em}=460 nm). Instead, non-processed ZMAL, does not serve as a substrate for the tryptic digestion and does not contribute to the fluorescence signal. Therefore, the fluorescence intensity is proportional to the activity of the respective sirtuin.

After subtraction of the blank fluorescence signal (without enzyme) from the signal of the respective probe, the residual signal intensity is related to a 100 % conversion control (AMC),

yielding the percentage substrate conversion. Comparison of the determined substrate conversion with a no-inhibition DMSO control yields the percentage inhibition at the respective inhibitor concentration.

For determination of IC_{50}, inhibitory activity of the respective compound was determined at 8 to 10 distinct concentrations. The measured values were analysed by a non-linear fit to the dose-response curve using a four parameter logistic model (Figure S6):

$$y = A2 + \frac{A1 - A2}{1 + \left(\frac{x}{x_0}\right)^p} \qquad x_0 = IC_{50}$$

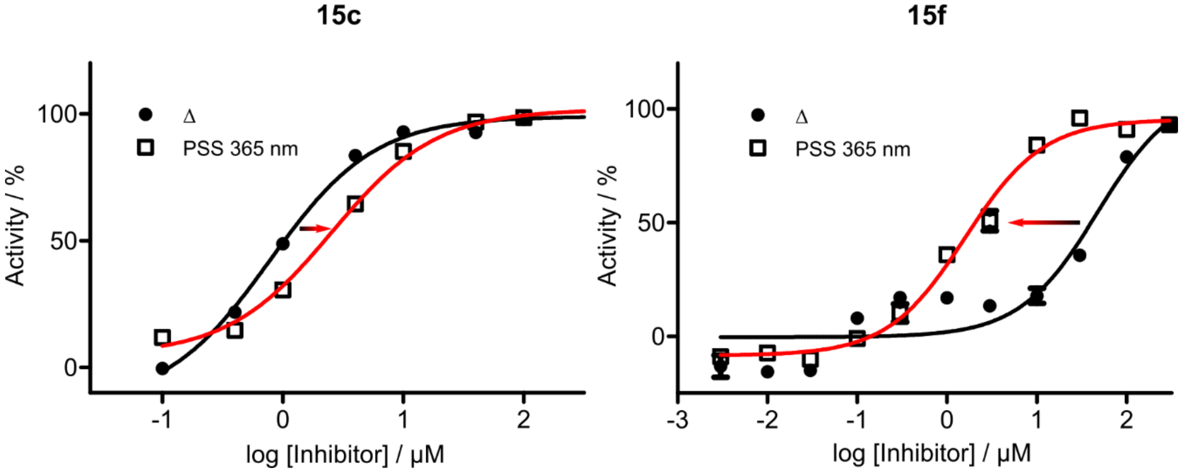

Figure S6: Non-linear regression of the dose-response curves of compounds **15c** and **15f** at the thermal equilibrium (Δ, black) and after 5 minutes of 365 nm irradiation (red).

FP-Based Binding Assay

To get more insight on the binding mode of the compounds **7c**, **7f** and **9a** a competition assay with a SirReal based fluorescent probe that binds to the selectivity pocket of Sirt2 was performed. A 400 nM solution of $Sirt2_{56\text{-}356}$ and a 160 nM solution of the TAMRA-labelled probe **21** were prepared in assay buffer (50 mM Tris, 137 mM NaCl, 2.7 mM KCl, 1 mg/mL BSA, 0.05% CHAPS, pH 8.0). As a negative control a 400 µM solution of unlabelled SirReal-inhibitor **22** in DMSO (20 µM final assay concentration) was prepared. The assay was performed in black 384-well non-binding microplates (Greiner Bio-One). 10 µL of enzyme solution (200 nM final assay concentration) were mixed with 1 µL of DMSO or inhibitor in various concentrations dissolved in DMSO (5% DMSO in assay) and filled up to a total volume of 15 µL with assay buffer. A maximal polarization control using just DMSO and a minimal polarization control using 1 µL of the 400 µM solution of **22** instead of inhibitor were performed as well as a blank control containing 1 µL of DMSO in 19 µL assay buffer. The mixture was incubated 10 min at 37 °C

before 5 µL of the solution of **21** (40 nM final assay concentration) were added. After further 30 min of incubation at 37 °C the fluorescence intensities were recorded with an EnVision™ plate reader (PerkinElmer) with the following settings: optical module – BODIPY TMR FP, excitation filter – FP 531 nm, emission filter 1 – FP p-pol 595 nm, emission filter 2 – FP s-pol 595 nm. Fluorescence polarization (P) values were determined as $\frac{(I_s - G \times I_p)}{(I_s + G \times I_p)} \times 1000\ (mP)$, where I_S/I_P are the fluorescence intensities measured in the s- and the p-plane respectively, both blank corrected. G (grating factor) is an instrument specific constant. Inhibition was calculated from fluorescence polarization in relation to the maximal polarization and minimal polarization controls and are displayed in % inhibition of binding of the fluorescent probe **21** (Table S1).

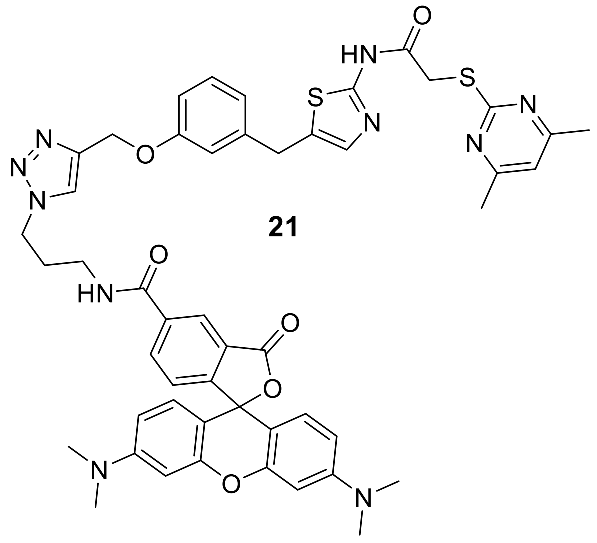

21

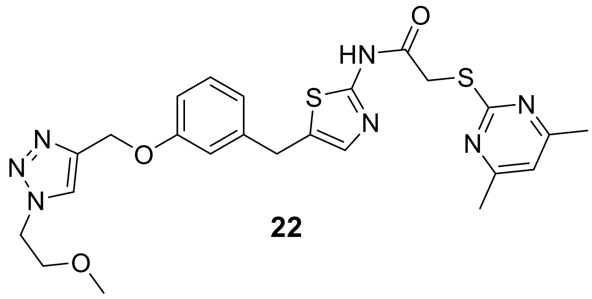

22

Table S3: Compounds **7c**, **7f** and **9a** examined in the FP-based binding assay and in the ZMAL activity assay. Inhibition of Sirt2 in the FP-based binding assay indicates displacement of the fluorescence-labelled SirReal probe and implies a similar binding mode.

Entry	Sirt2 inhibition FP-Assay[a]	Sirt2 inhibition ZMAL-Assay[a]
**7c**	n.i.	55% @ 10 µM
**7f**	46% @ 10 µM	5.8 ± 0.7 µM[b]
**9a**	69% @ 100 µM	65% @ 100 µM

[a] Percent inhibition relative to controls at the indicated concentration, [b] IC_{50} values (µM) with statistical limits; values are the mean ± SD of duplicate experiments, n.i. = no inhibition detected.

Cell-Based Activity Assay

To qualify inhibitory activity of compounds on sirtuins we used the western blot technique. Therefore, we used precast “Mini-Protean TGX Stain-Free Gels” and corresponding “Trans-Blot Turbo Transfer Pack Mini” PVDF membranes (Biorad, Germany). For sample preparation, we used the urinary bladder cancer cell line RT-4, which showed the highest expression of Sirt2 in our cell line stock (Data not shown), obtained from “Deutsche Sammlung von Mikroorganismen und Zellkulturen” (DSMZ), Germany. Cells were grown in RPMI 1640 cell medium supplemented with 10% fetal bovine serum and 1% penicillin/streptomycin purchased from PAN Biotec, Germany and stored in a humidified incubator at 37 °C with 5% CO_2 atmosphere. For sample preparation, cells were harvested with Trypsin/EDTA and counted in a Beckman’s Coulter Counter. In six well plates, $5 \cdot 10^5$ cells were seeded out in 3 mL medium and let allow to attach for 24 h. Stock solutions of compounds (1000-fold) were prepared with DMSO and illuminated with UV light (365 nm) for 5 min. Then, cells were exposed to compounds at a final concentration of 50 µM and incubated for 24 h. Cells were detached with Trypsin/EDTA and washed with phosphate buffered saline (PBS) by centrifugation at 500 g for 5 min and resuspended in PBS. Cell pellets were lysed with a lysis buffer containing Tris 50 mM (pH 7.4), 100 mM NaCl, 100 mM NaF, 5 mM EDTA, 0.2 mM Na_3VO_4, 0.1% Triton X and freshly added 1% protease inhibitor cocktail (Sigma Aldrich, Germany) on ice, followed by sonification for 30 minutes. The protein concentration was determined by the Bradford method against bovine serum albumin (BSA) as standard. Each slot of the precast gel was loaded with 30 µg protein followed by an electrophoretic separation and a transfer onto PVDF membranes.

Blots were blocked with 10% non-fat milk powder in Tris-buffered saline containing 20 mM Tris, 145 mM NaCl and Tween 0.5% (TBST) for 2 h and incubated with primary antibody in 1:1000 dilutions (in TBST and 1% BSA) over night at 4 °C. After washing procedure, blots were incubated with secondary antibody conjugated with horse radish peroxidase (1:5000 in TBST and 1% BSA) for 2 h. All used antibodies were obtained from cell signalling (UK): anti ac.H3K9 (#9649), anti ac. H3K56 (#4243), anti H3 (#4499), anti ac. H4K8 (#2594), anti H4 (#13919), anti ac.α-tubulin (#5335), anti α-tubulin (#2125) and anti rabbit-HRP (#7074). Target protein bands were detected with Clarity Western ECL Substrate (Biorad, Germany) and recorded at an Advanced Fluorescence Imager (INTAS, Germany). Band intensity was related to internal standard of the TGX Stain-Free gels as loading control.

Dihedral Analysis

In order to verify the conformations of *Z*-**15c** and *Z*-**15f** obtained from our molecular docking experiments, a two-dimensional dihedral analysis was performed using MOE with 3-(2-phenyldiazenyl)-pyridine as reference compound. Both dihedrals were rotated by 360° in 5° steps. At each step, the potential energy was calculated with AMBER force field parameters and AM1-BCC partial charges applied. The diazenyl double bond of both compounds is nearly perfectly planar with values of 2.5° ((*Z*)-**15c**) and 2.1° ((*Z*)-**15f**), respectively. All values were truncated to 10 kcal/mol after subtracting the value of the lowest energy conformation.

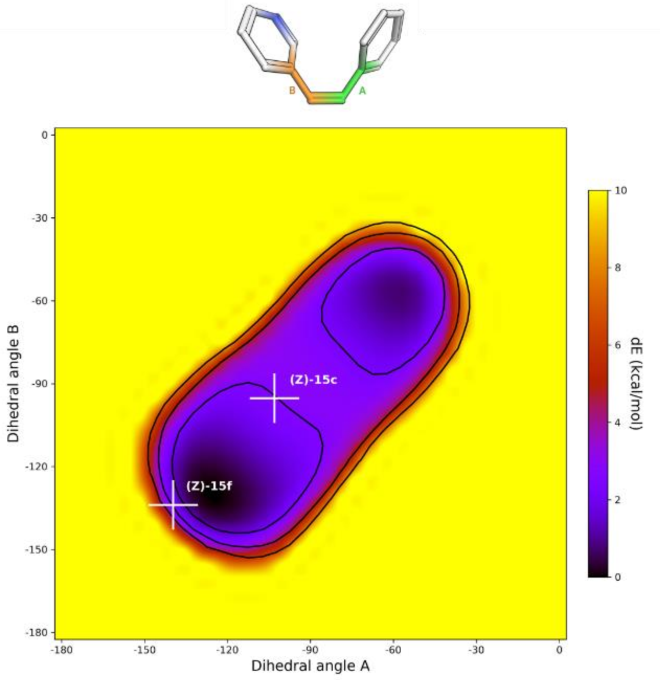

Figure S7: Potential energy diagram of 3-(2-phenyldiazenyl)-pyridine in relation to the CCNN-dihedral angles A and B. Conformations of the *Z* isomers depicted in Figure 3 and Figure 4 are assigned as white crosses.

NMR Spectra

Methyl (*E*)-5-[(4-hydroxy-2,6-dimethylphenyl)diazenyl]nicotinate (**6a**)

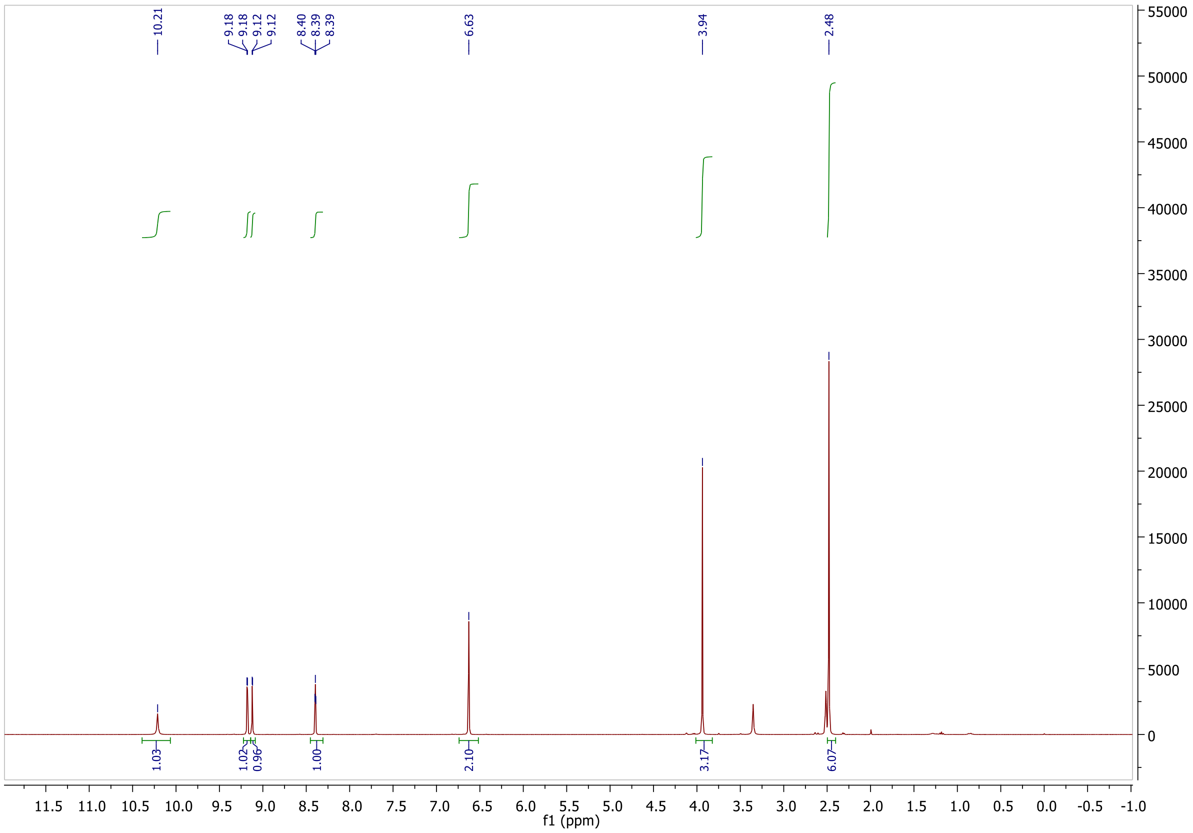

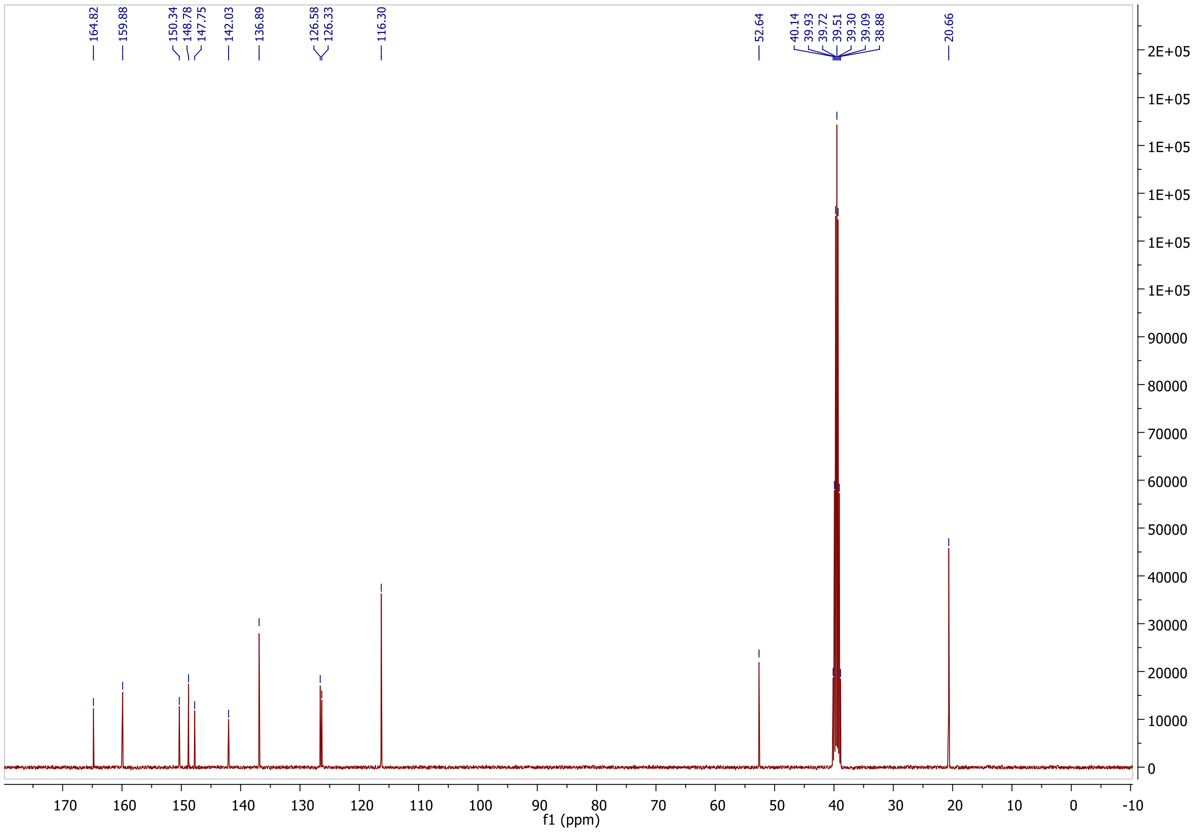

Methyl (*E*)-5-[(4-hydroxy-3,5-dimethylphenyl)diazenyl]nicotinate (**6b**)

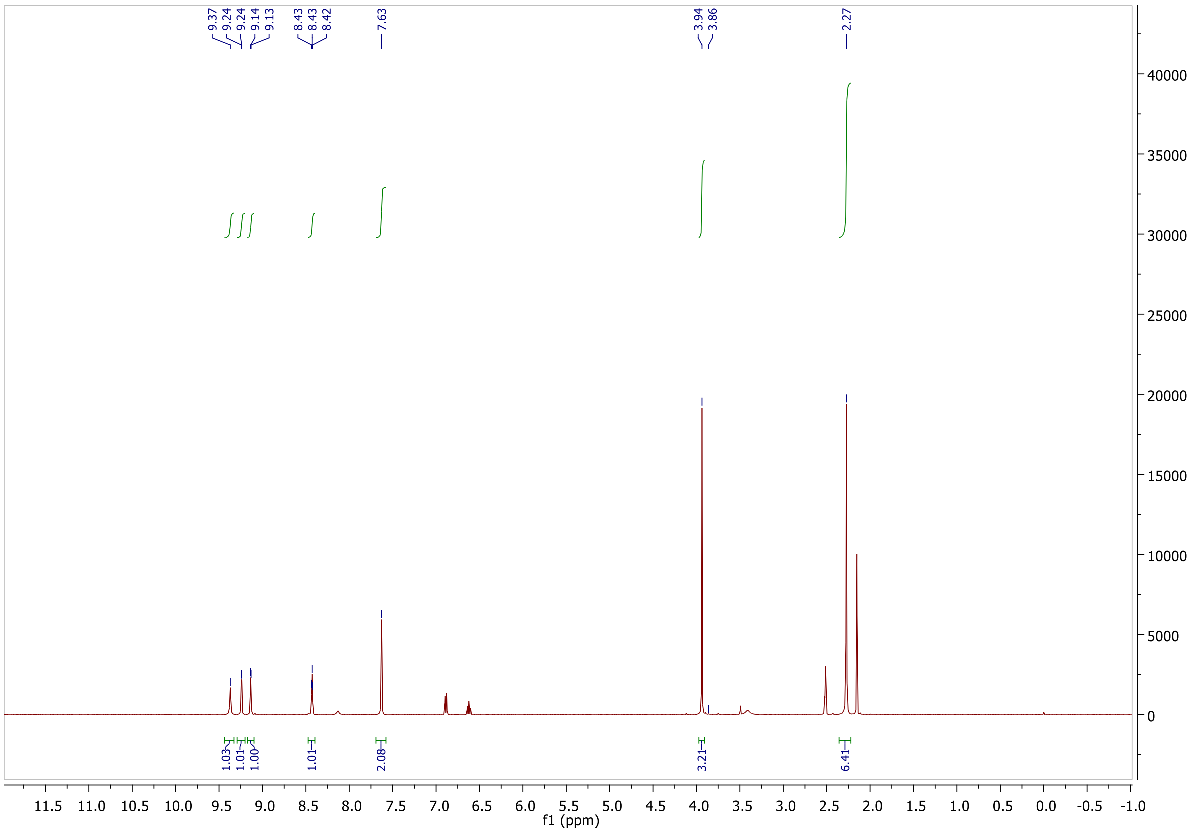

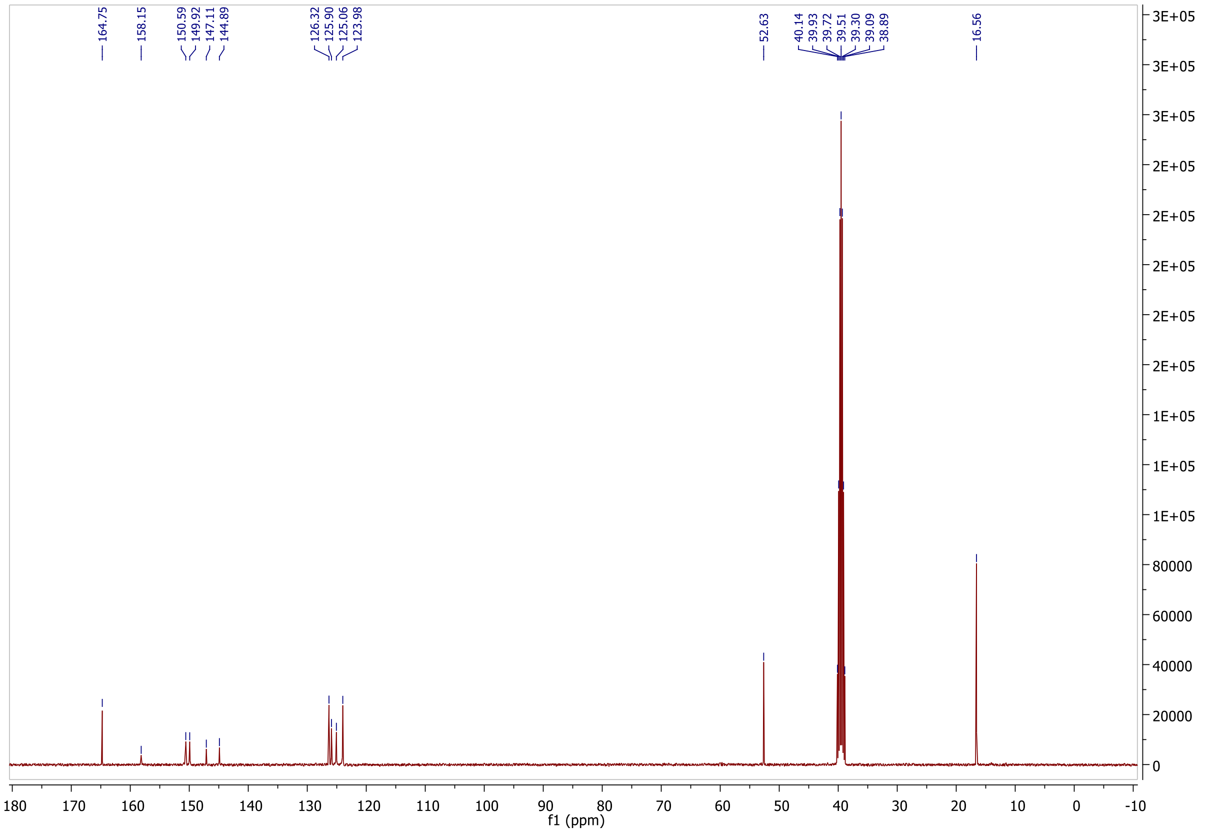

Methyl (*E*)-5-[(2-hydroxynaphthalen-1-yl)diazenyl]nicotinate (**6c**)

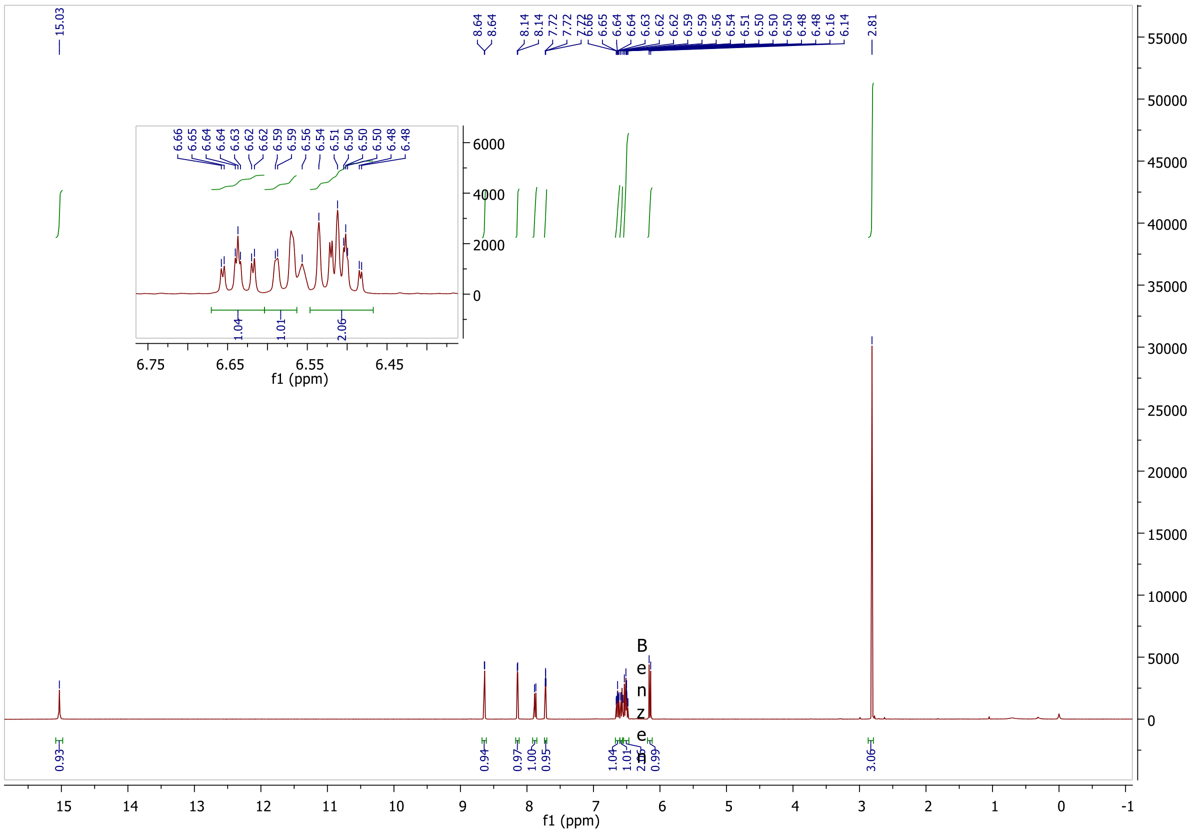

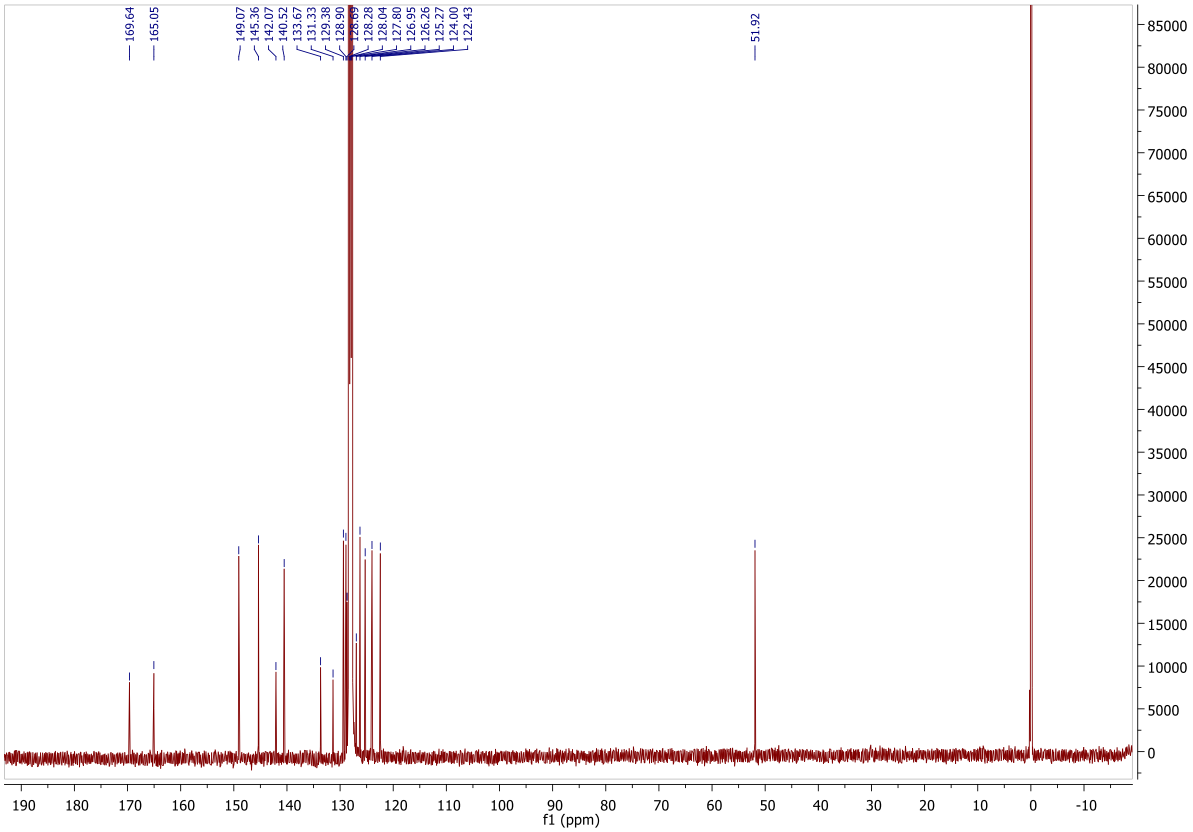

Methyl (*E*)-5-[(4-hydroxy-3,5-dimethoxyphenyl)diazenyl]nicotinate (**6d**)

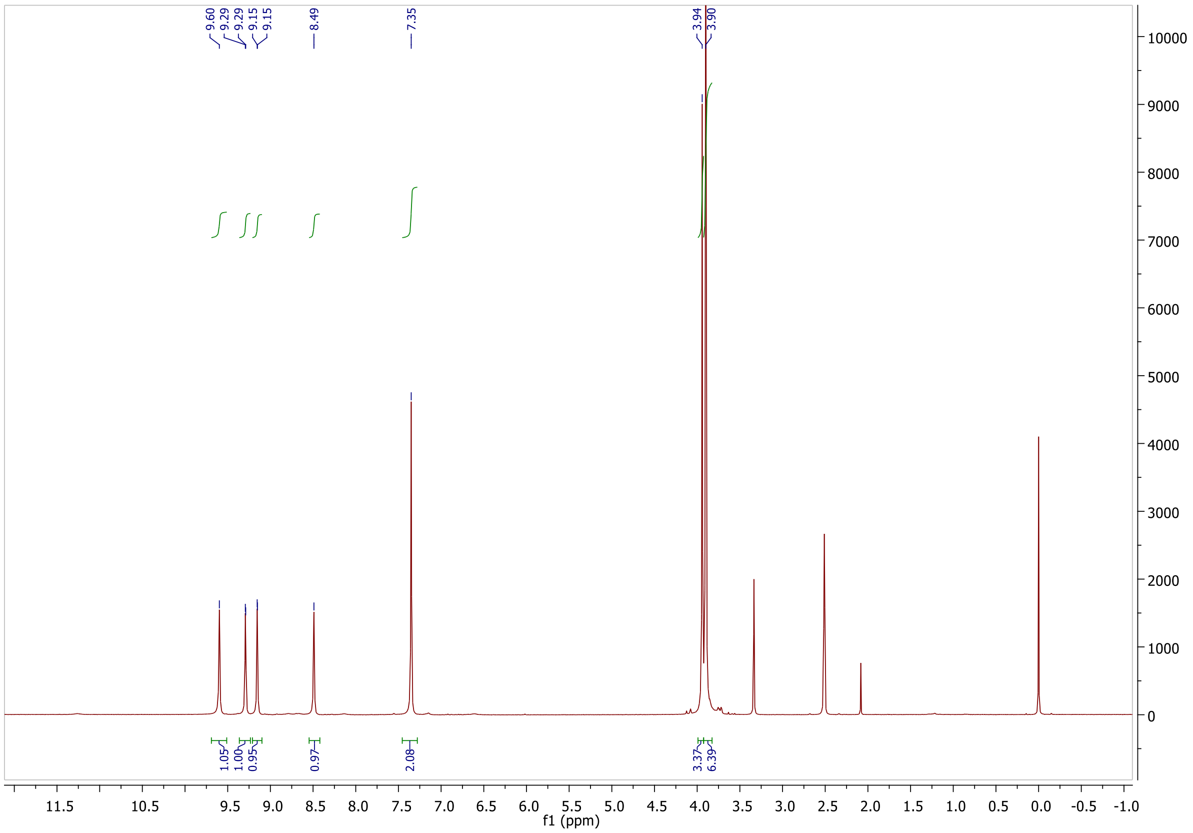

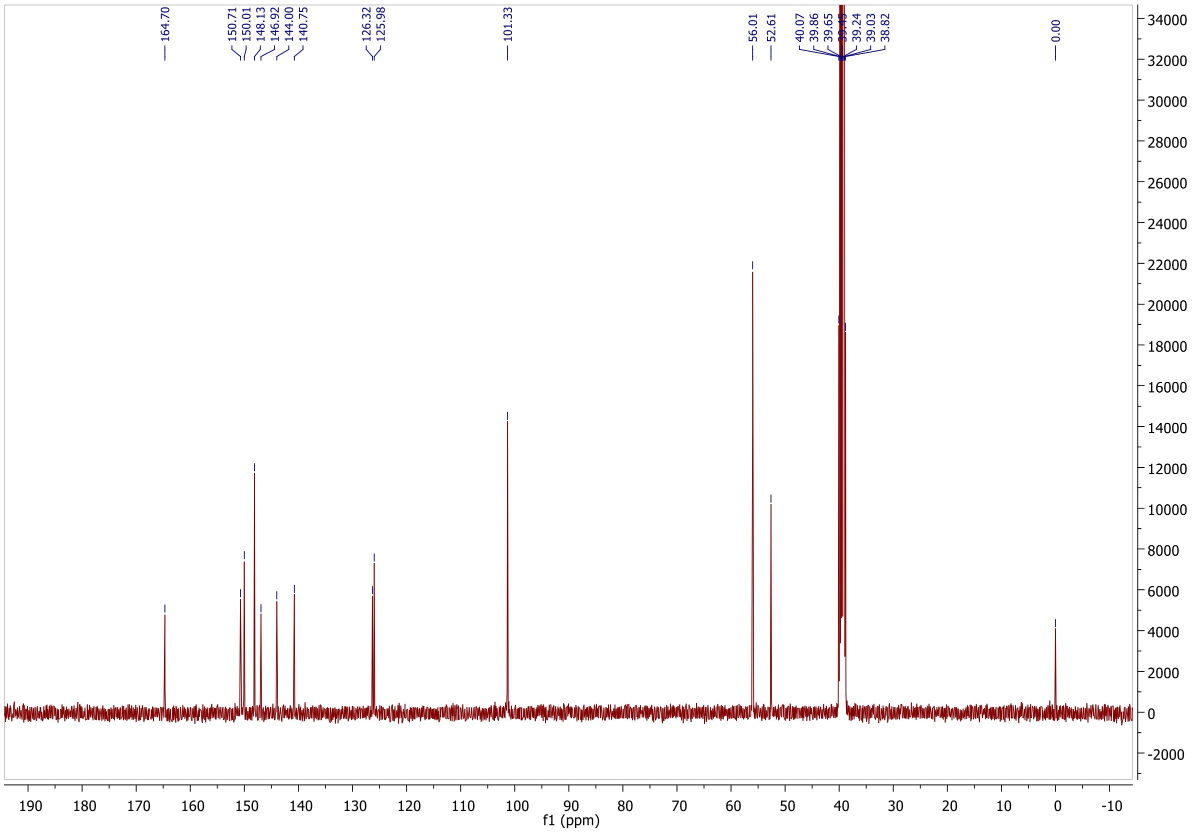

Methyl (*E*)-5-{[4-(dimethylamino)phenyl]diazenyl}nicotinate (**6e**)

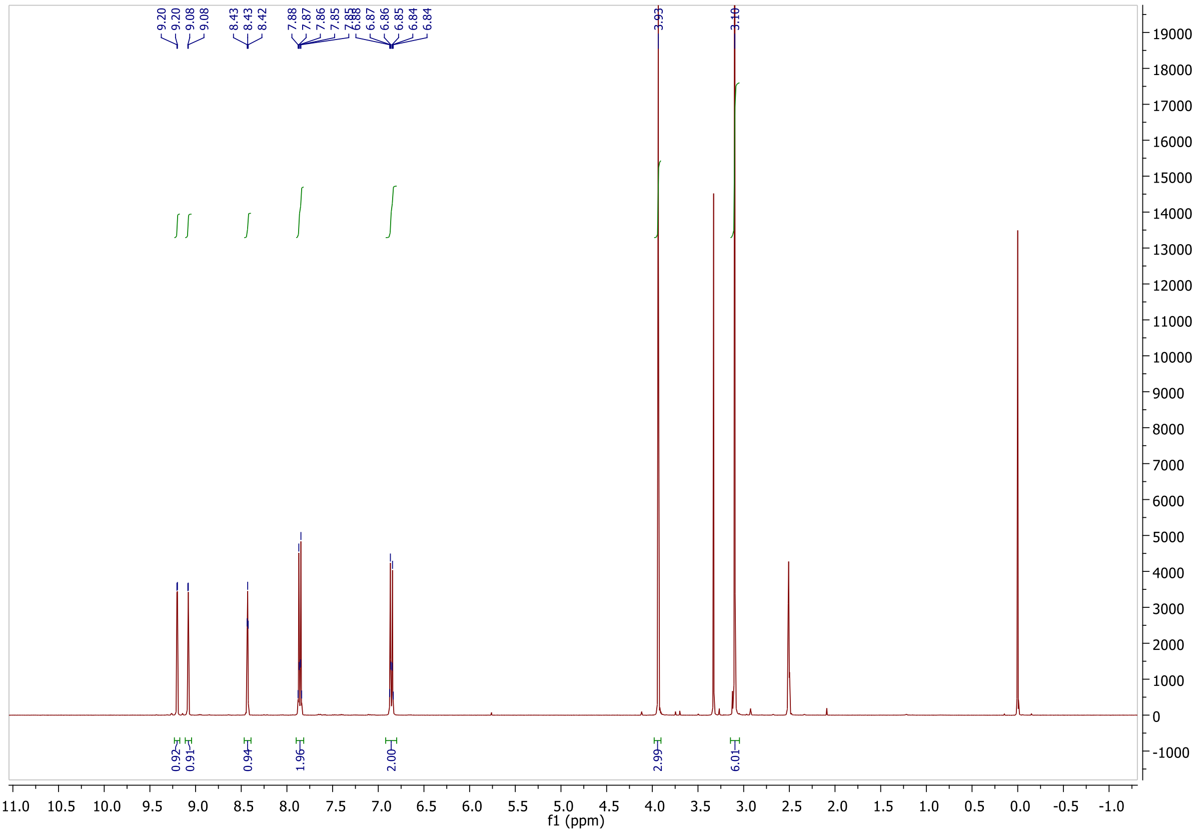

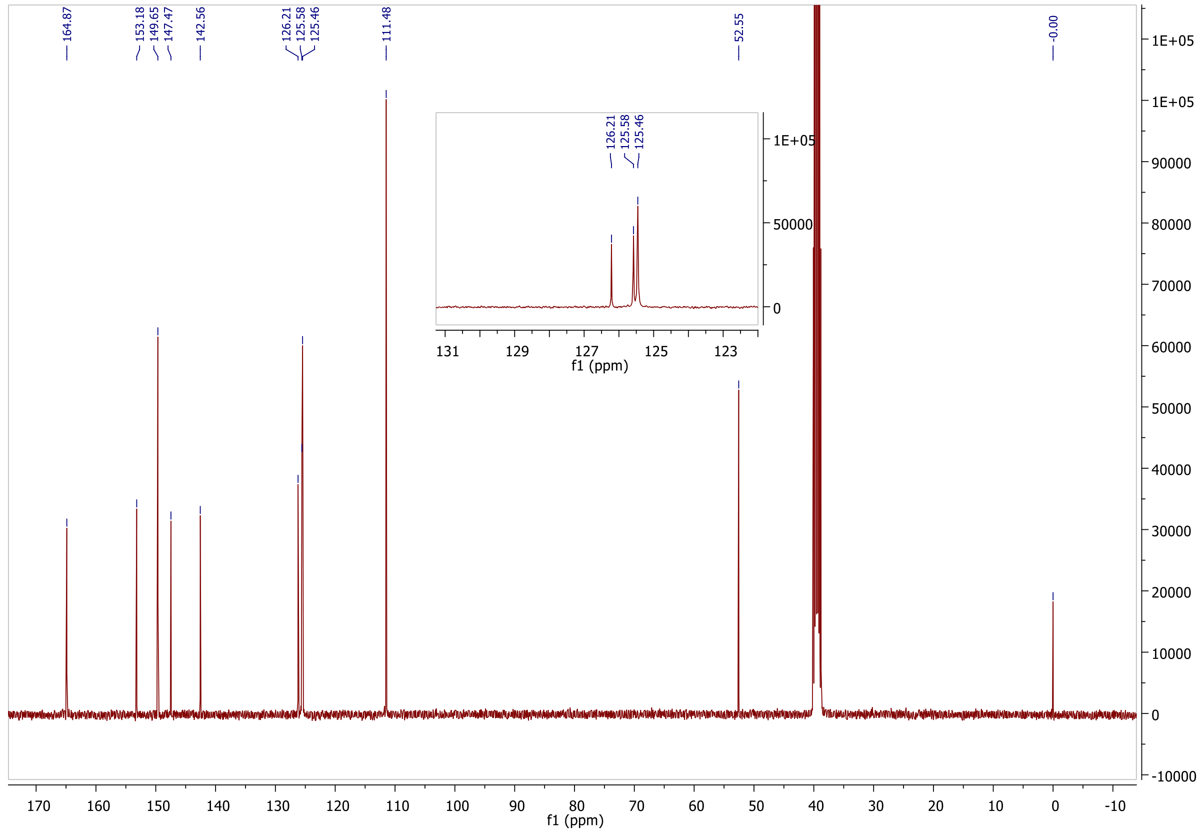

Methyl (*E*)-5-[(4-amino-3,5-dimethylphenyl)diazenyl]nicotinate (**6f**)

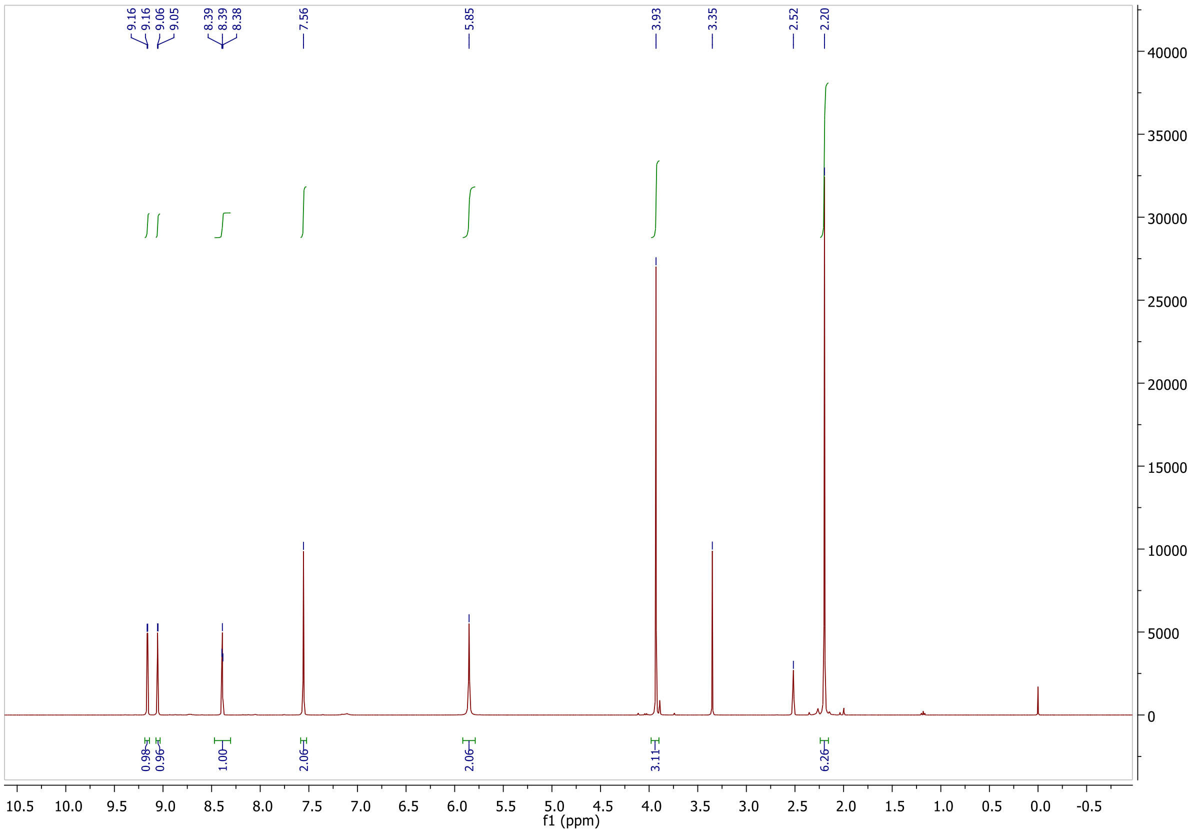

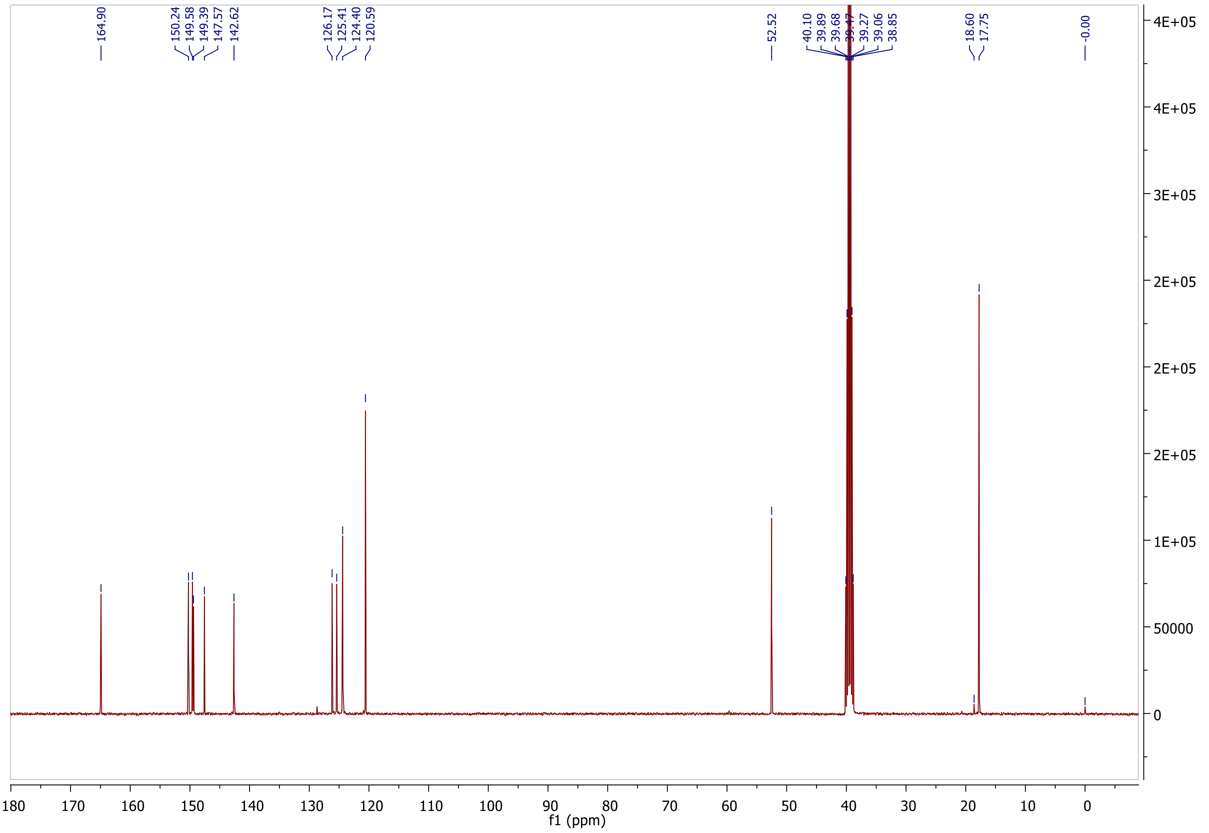

(*E*)-5-[(4-Hydroxy-2,6-dimethylphenyl)diazenyl]nicotinamide (**7a**)

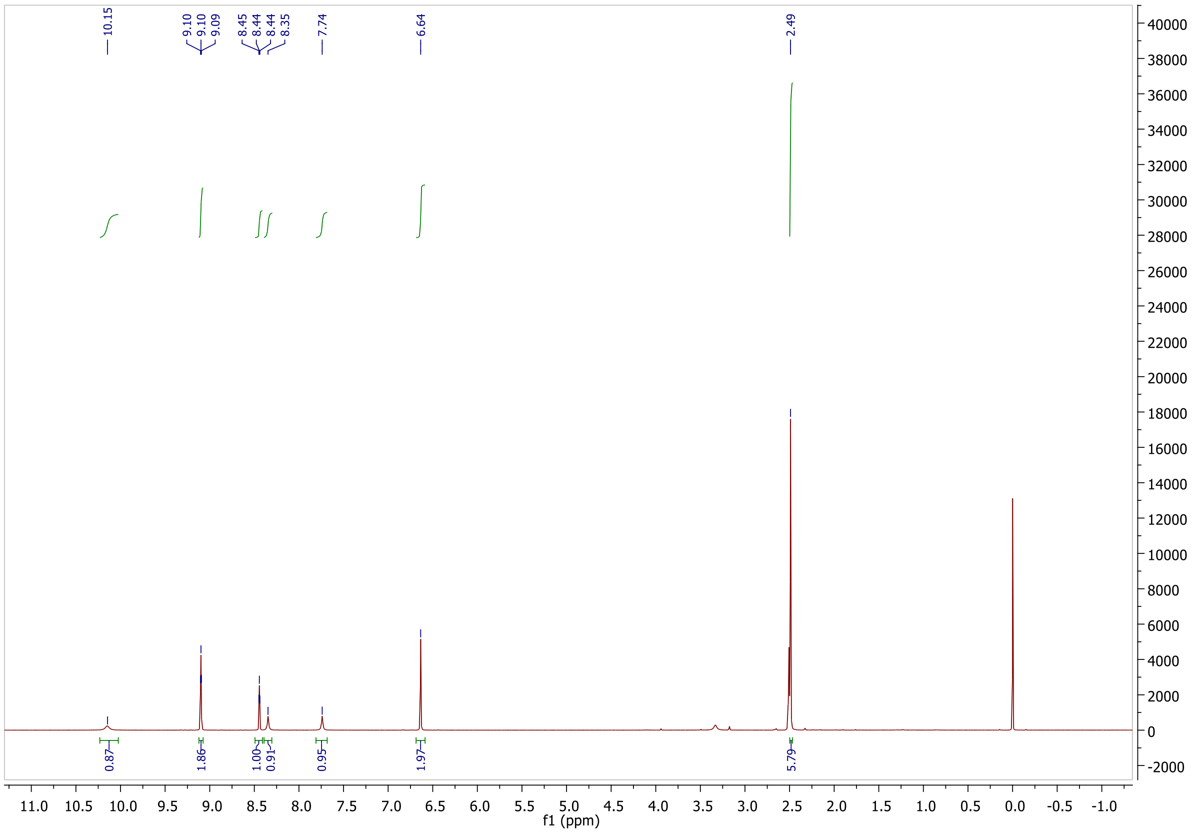

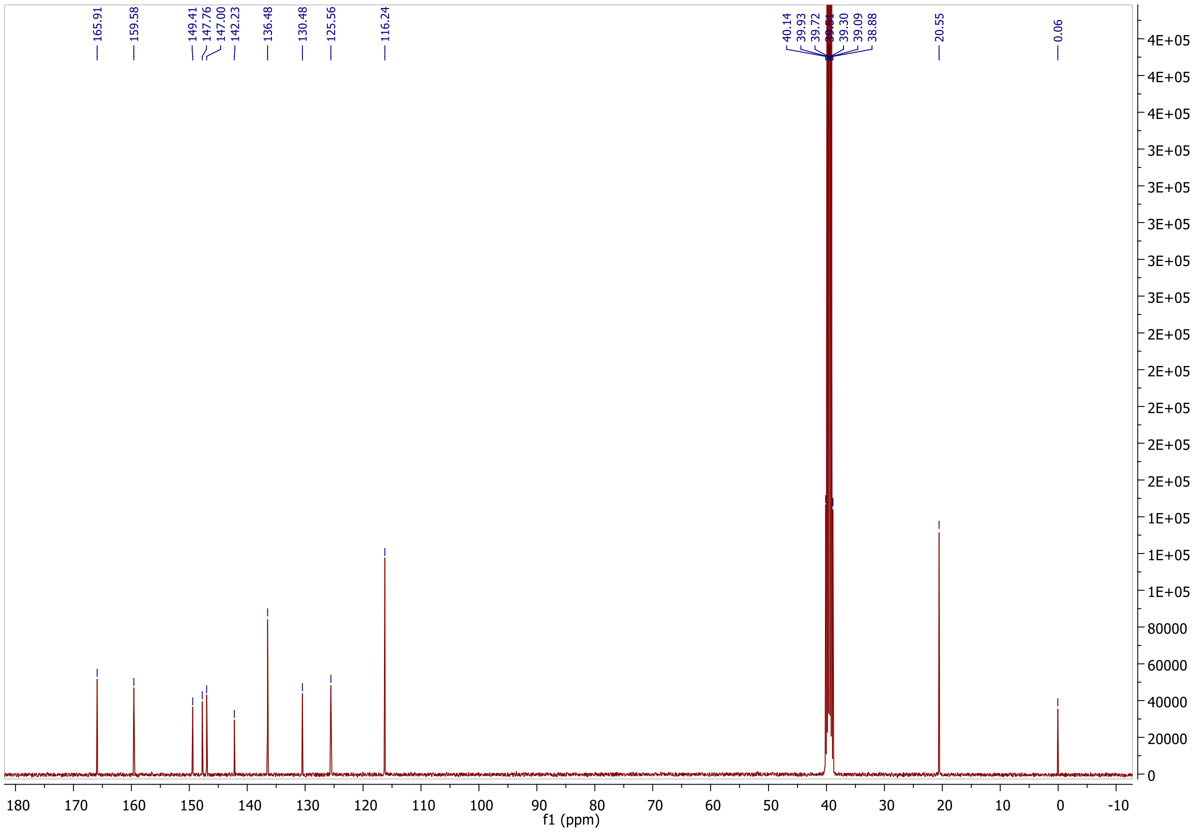

(*E*)-5-[(4-Hydroxy-3,5-dimethylphenyl)diazenyl]nicotinamide (**7b**)

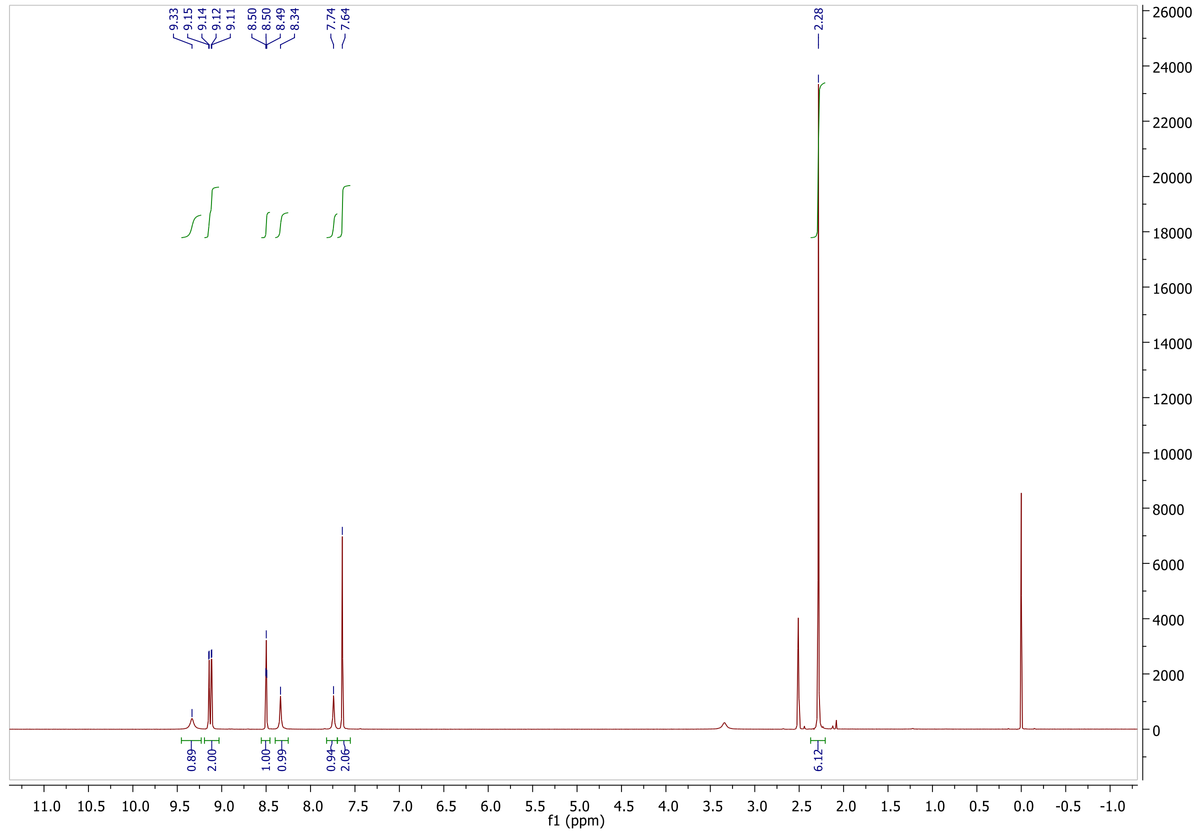

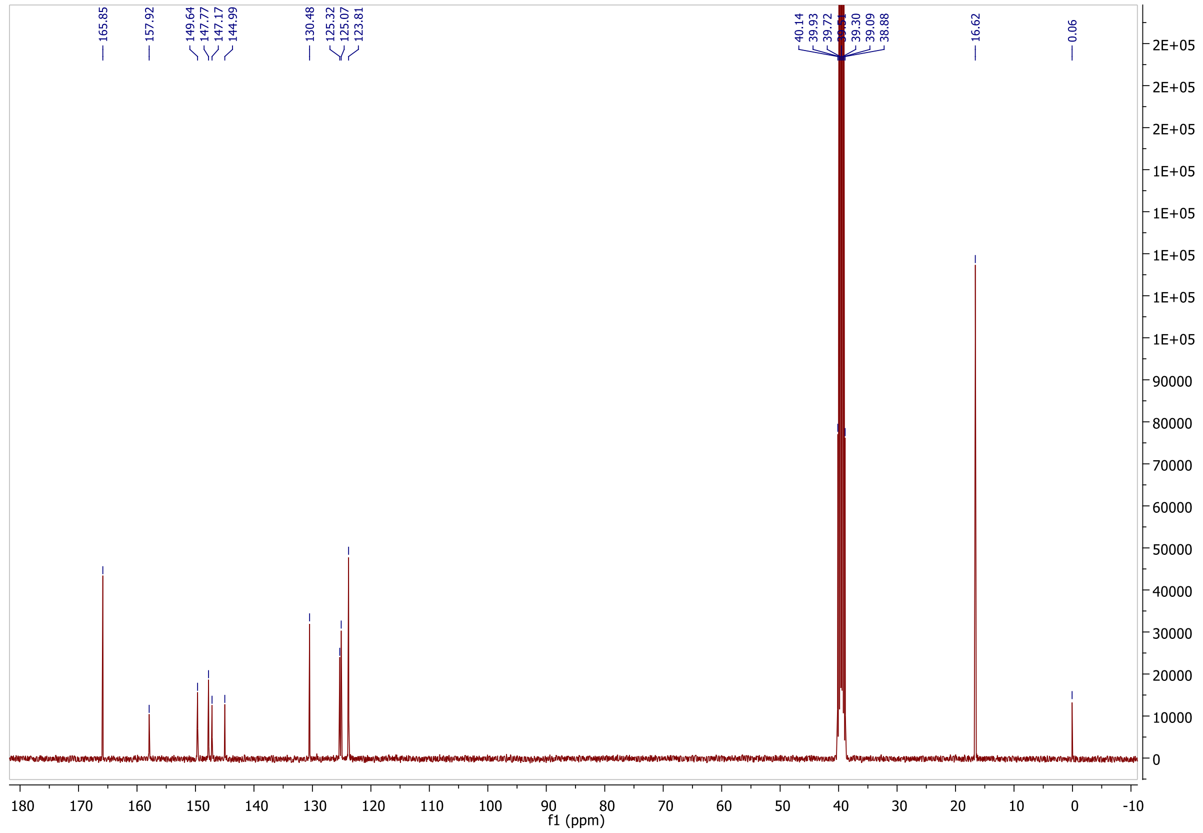

(*E*)-5-[(2-Hydroxynaphthalen-1-yl)diazenyl]nicotinamide (**7c**)

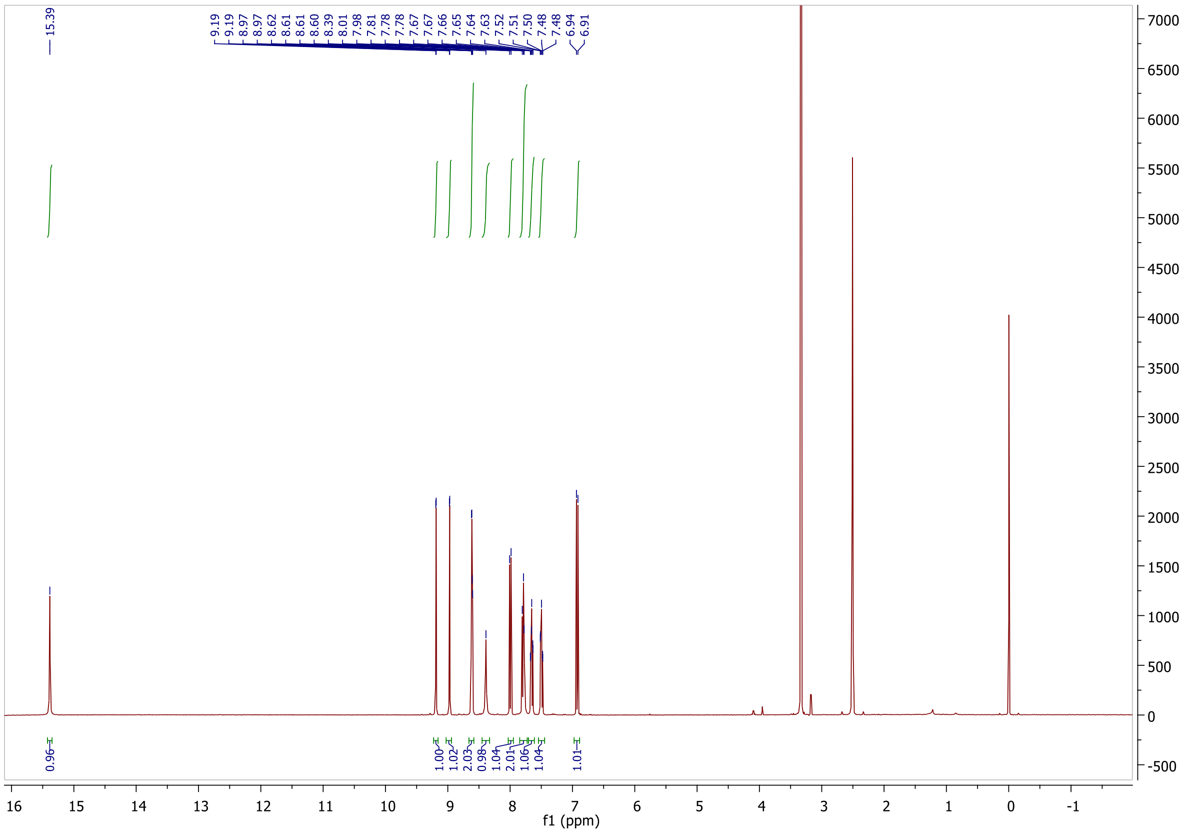

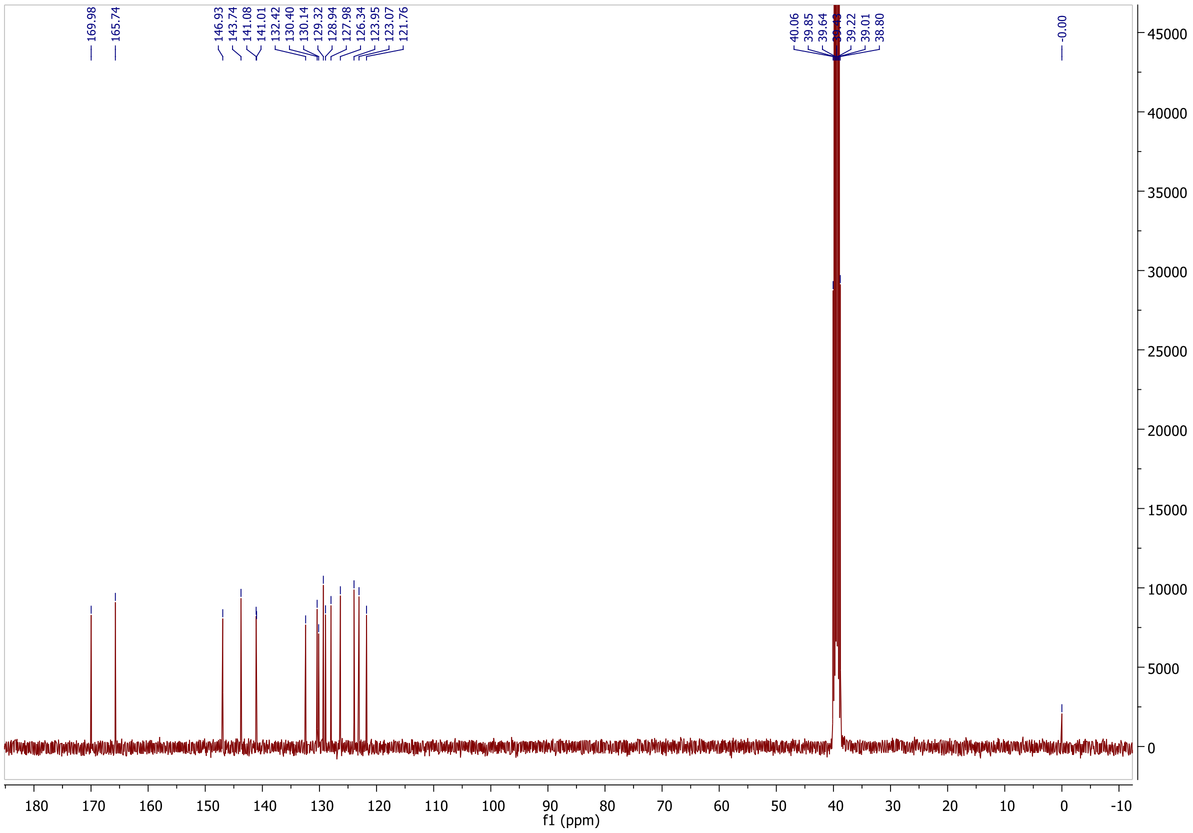

(*E*)-5-[(4-Hydroxy-3,5-dimethoxyphenyl)diazenyl]nicotinamide (**7d**)

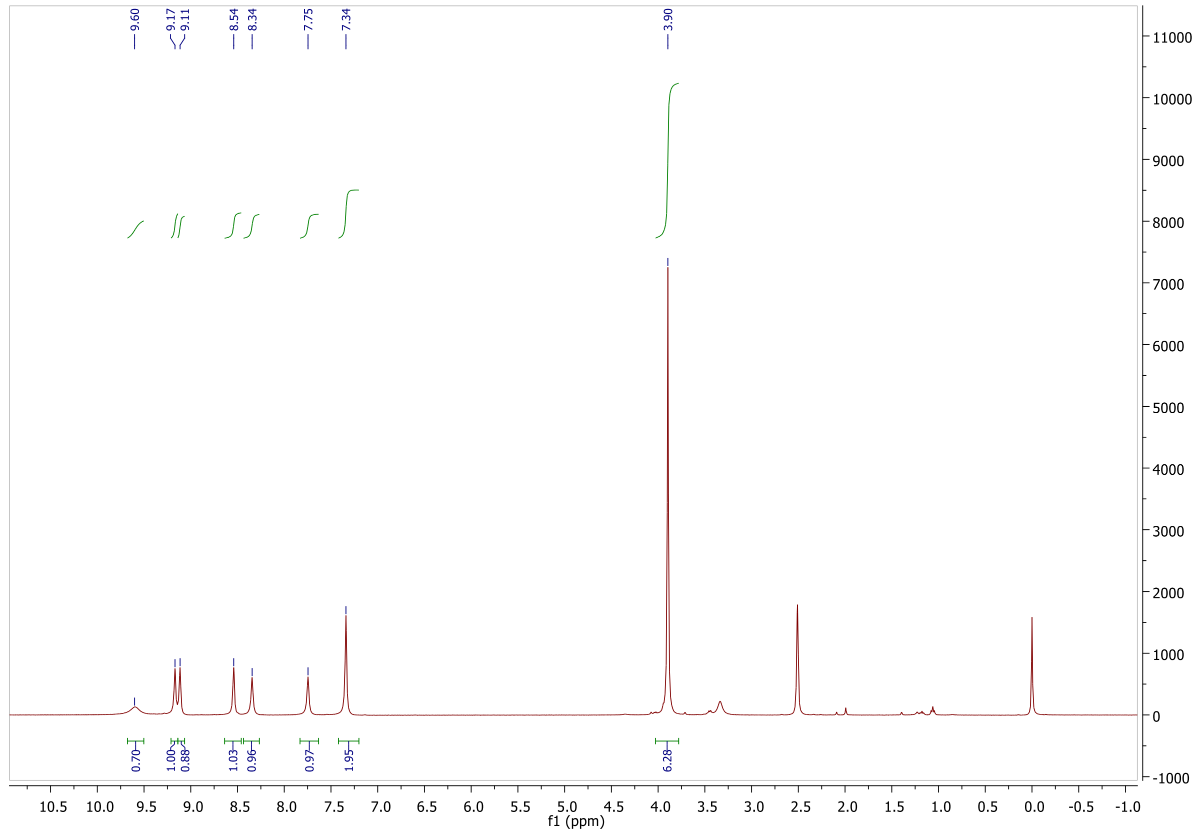

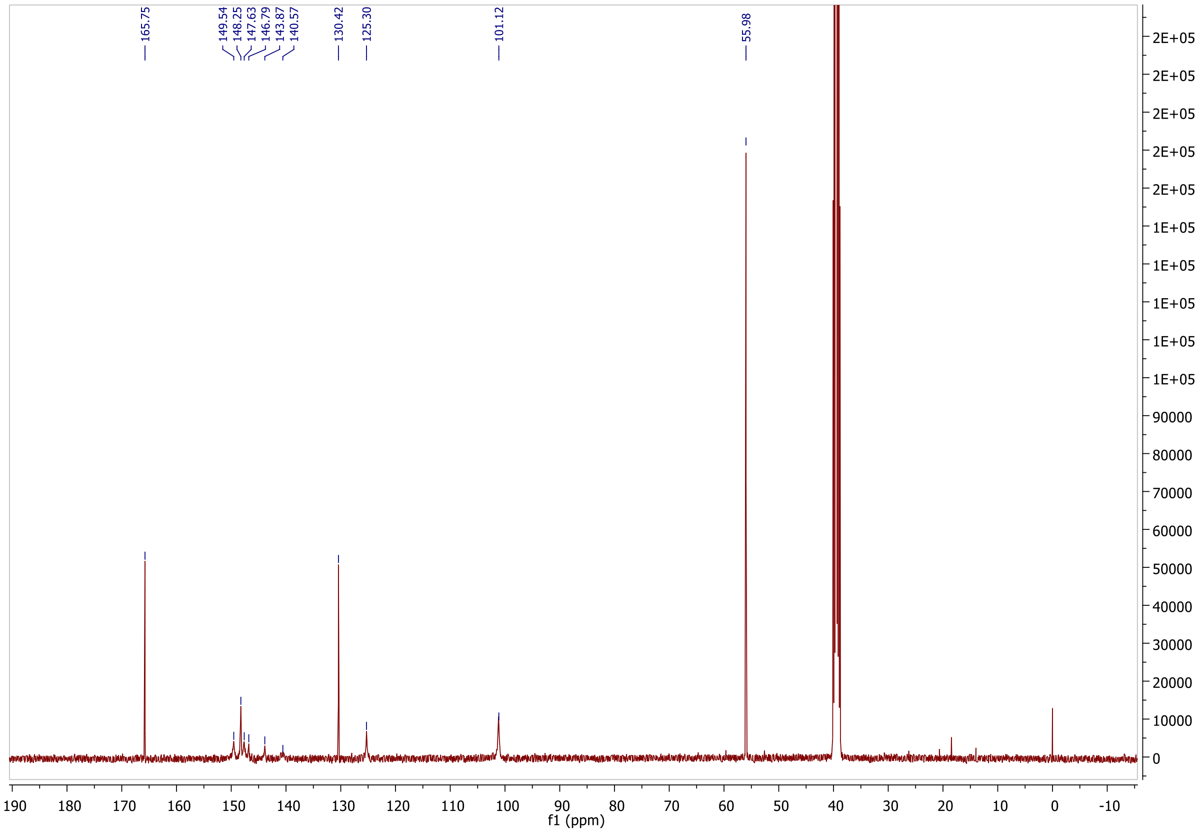

(*E*)-5-{[4-(Dimethylamino)phenyl]diazenyl}nicotinamide (**7e**)

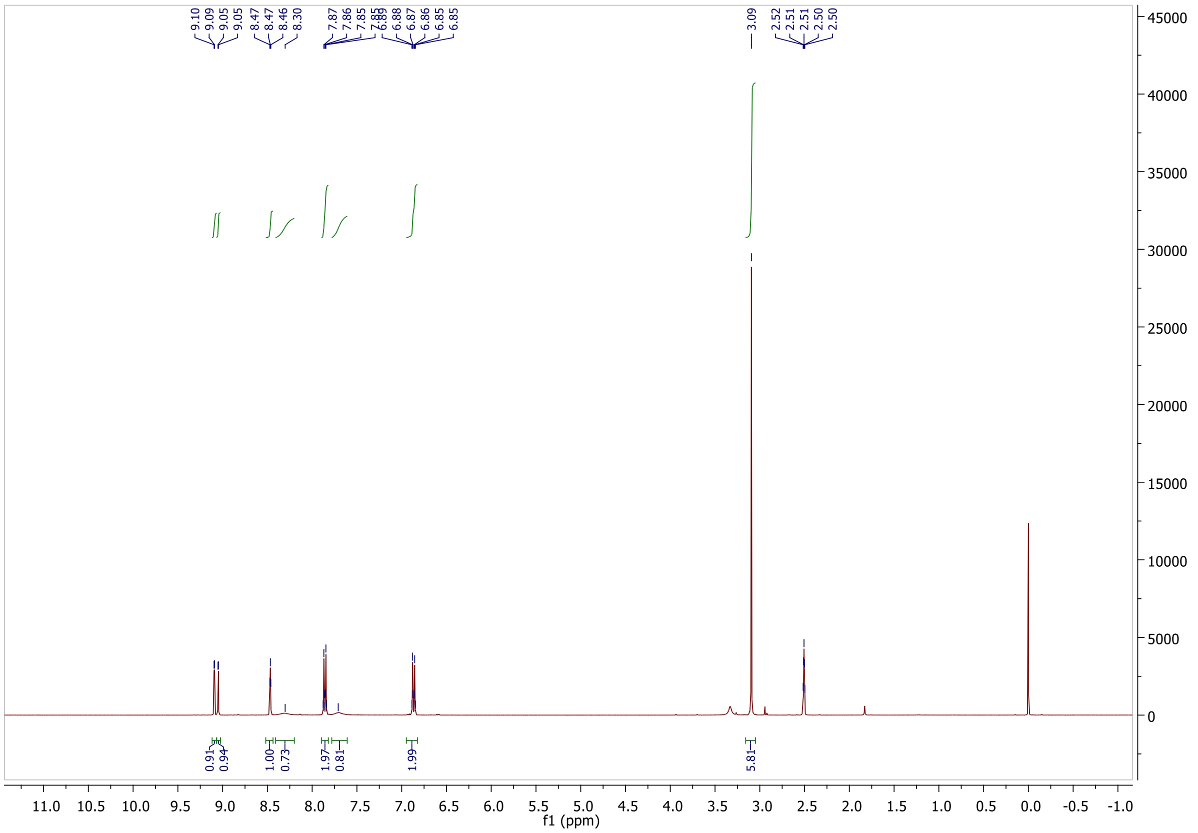

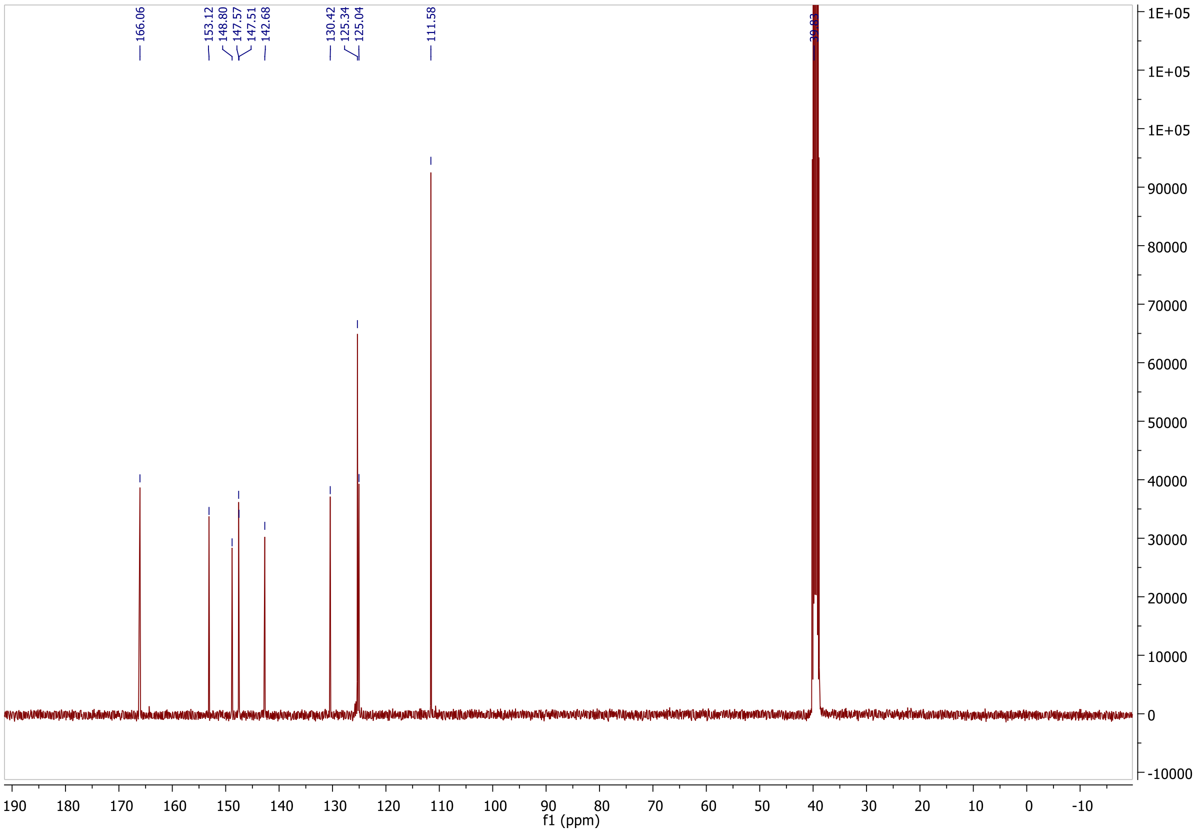

(*E*)-5-[(4-Amino-3,5-dimethylphenyl)diazenyl]nicotinamide (**7f**)

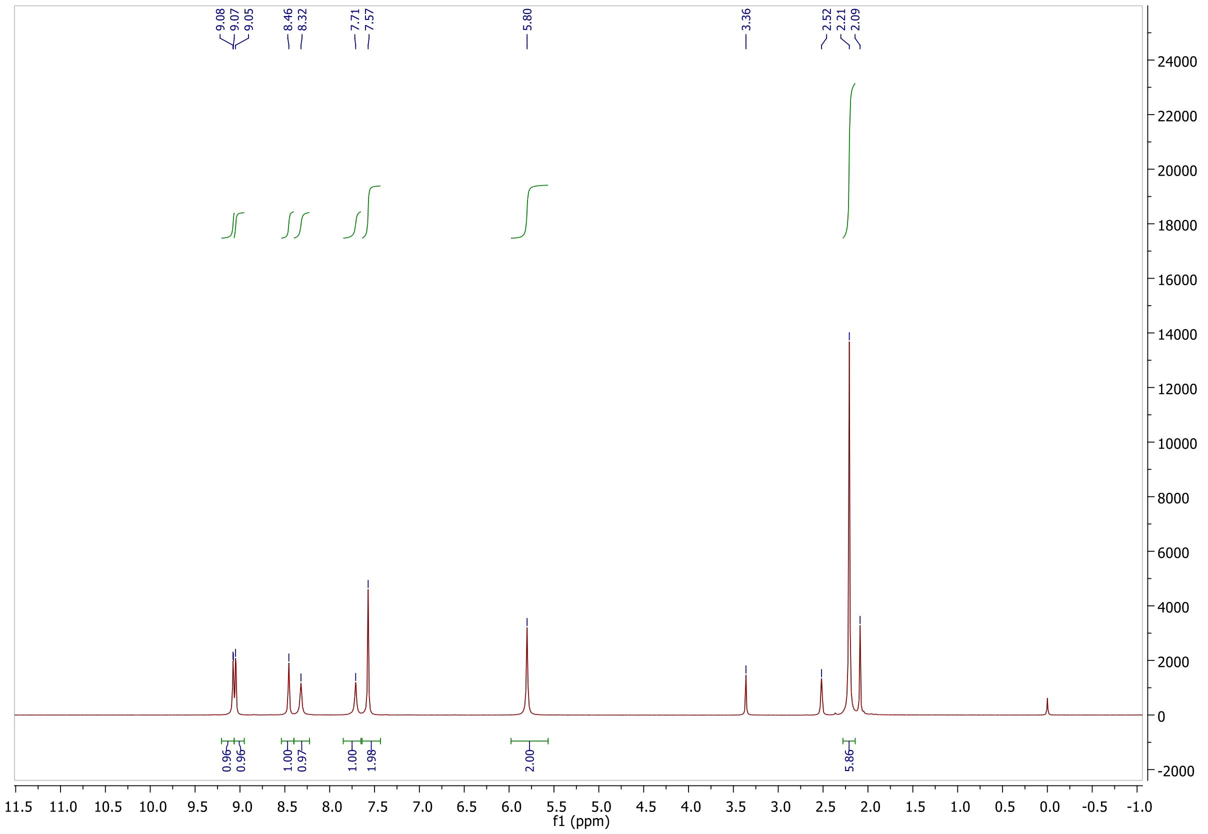

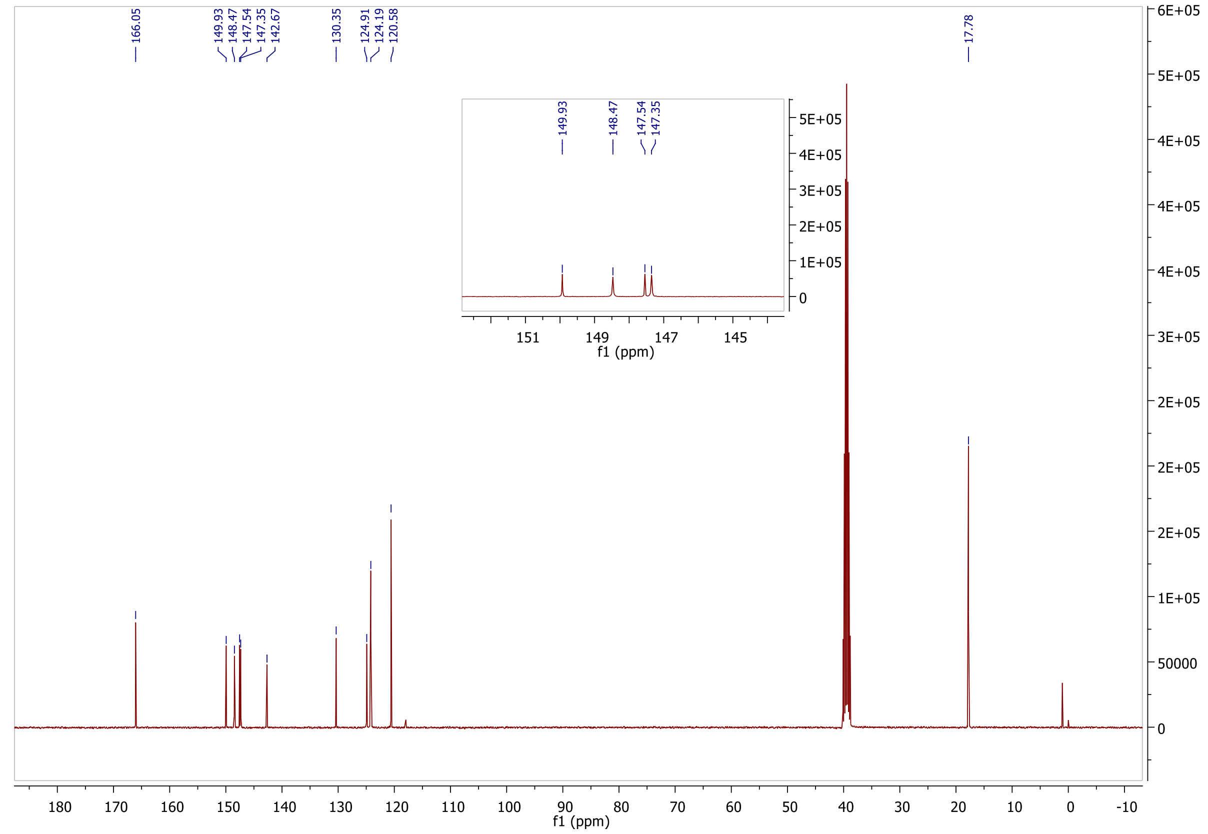

Methyl (*E*)-5-[(4-acetamido-3,5-dimethylphenyl)diazenyl]nicotinate (**8a**)

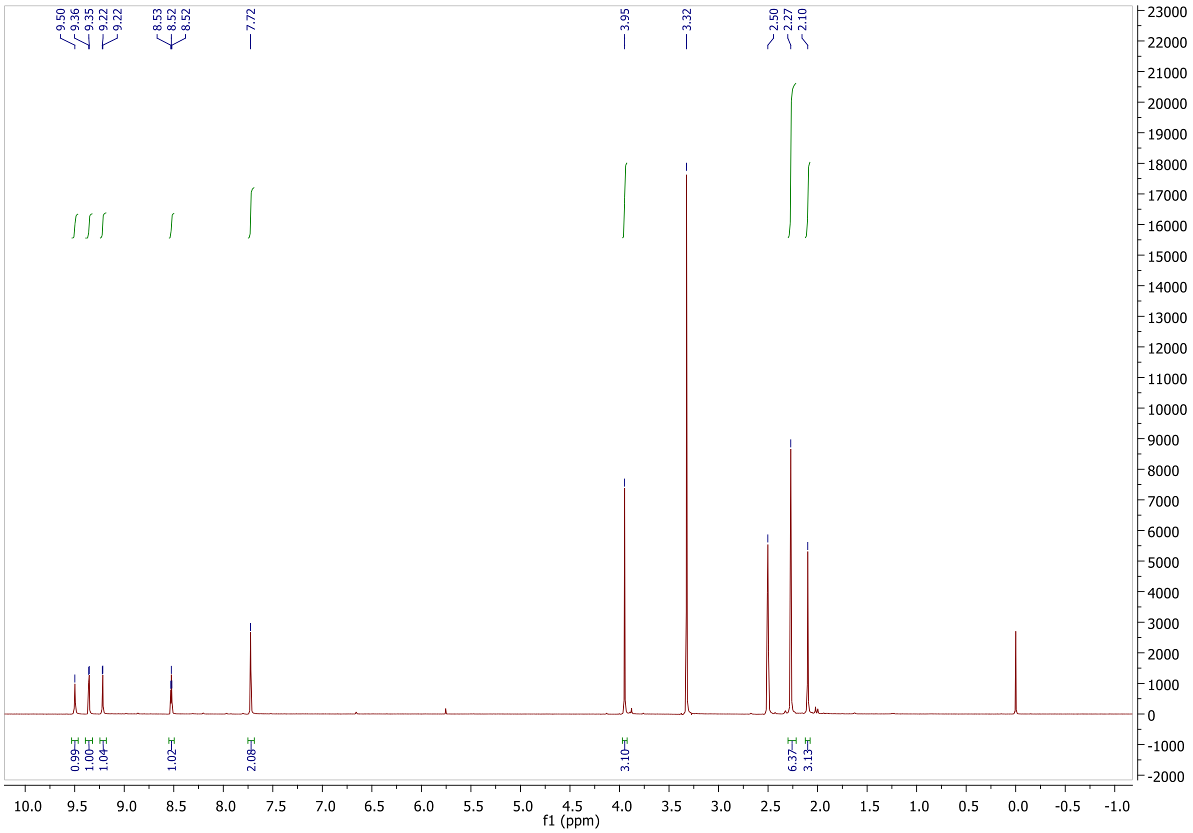

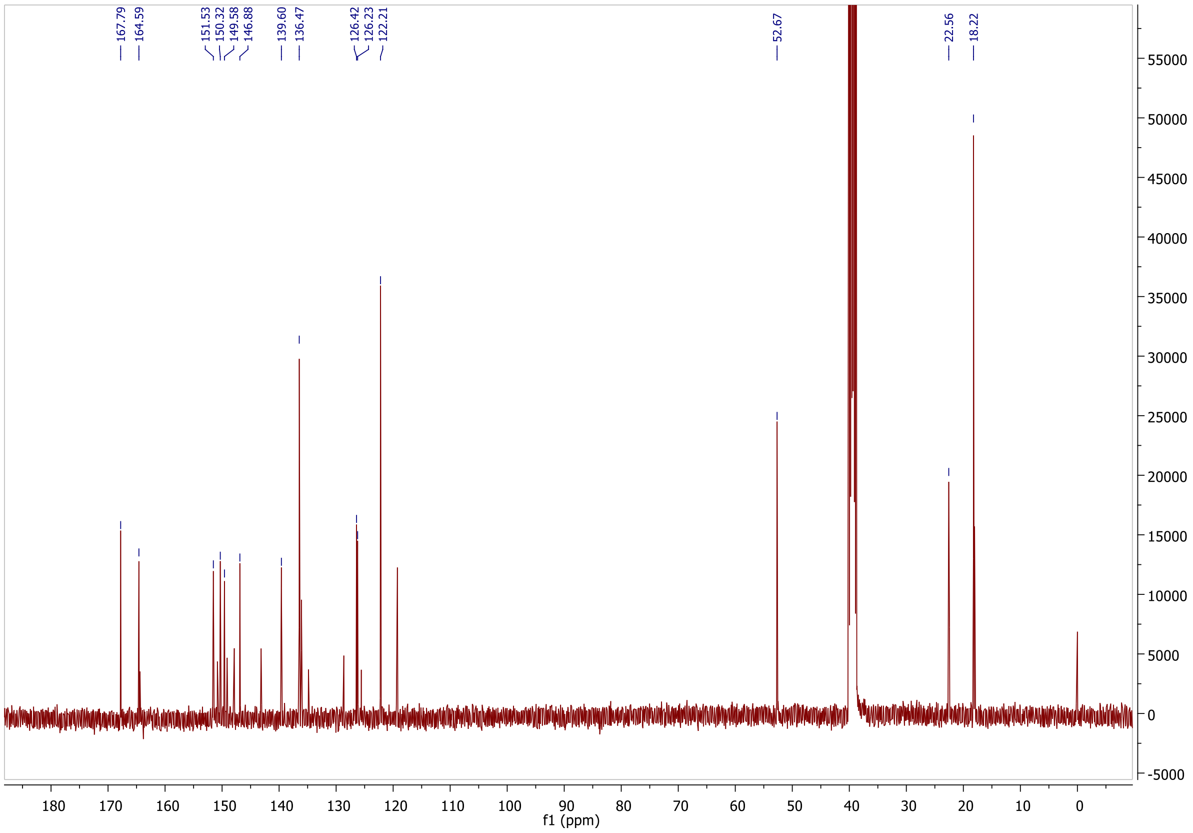

Additional (minor) signals derive from (*Z*)-photoisomer formed by daylight.

Methyl (*E*)-5-[(4-butyramido-3,5-dimethylphenyl)diazenyl]nicotinate (**8b**)

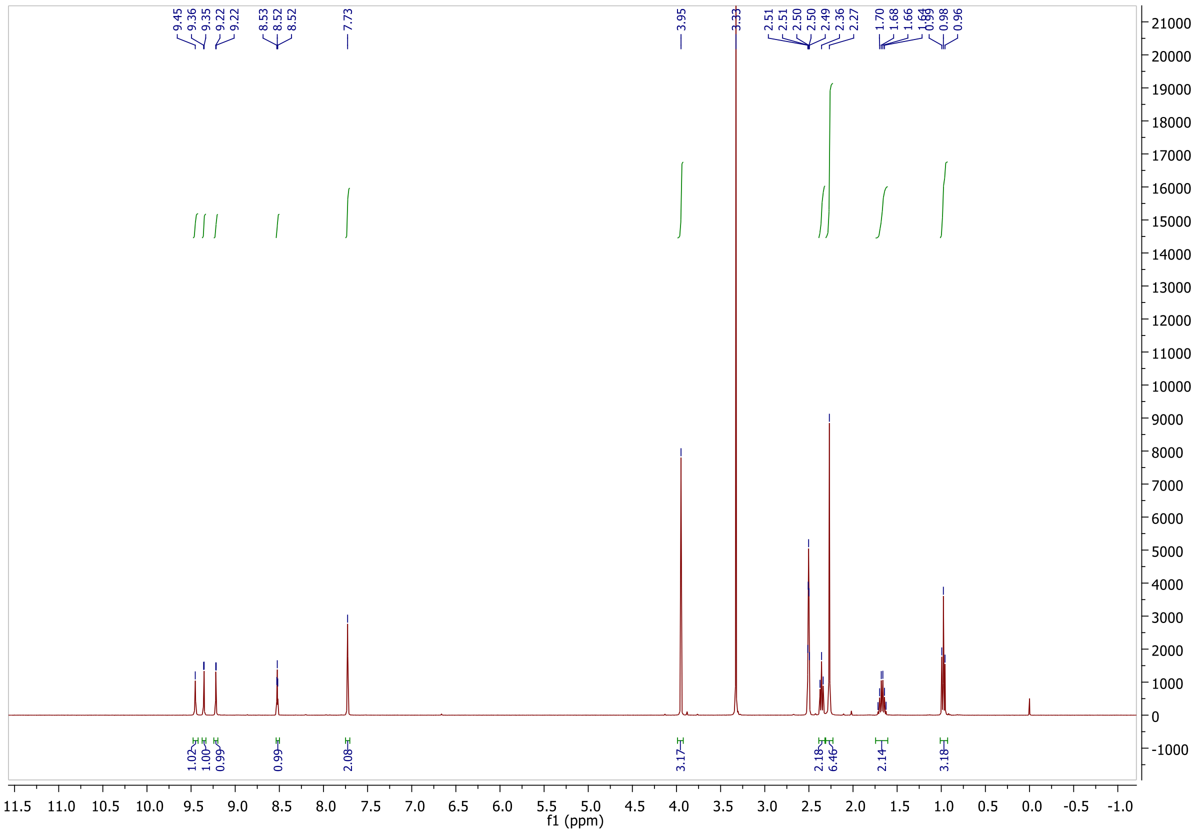

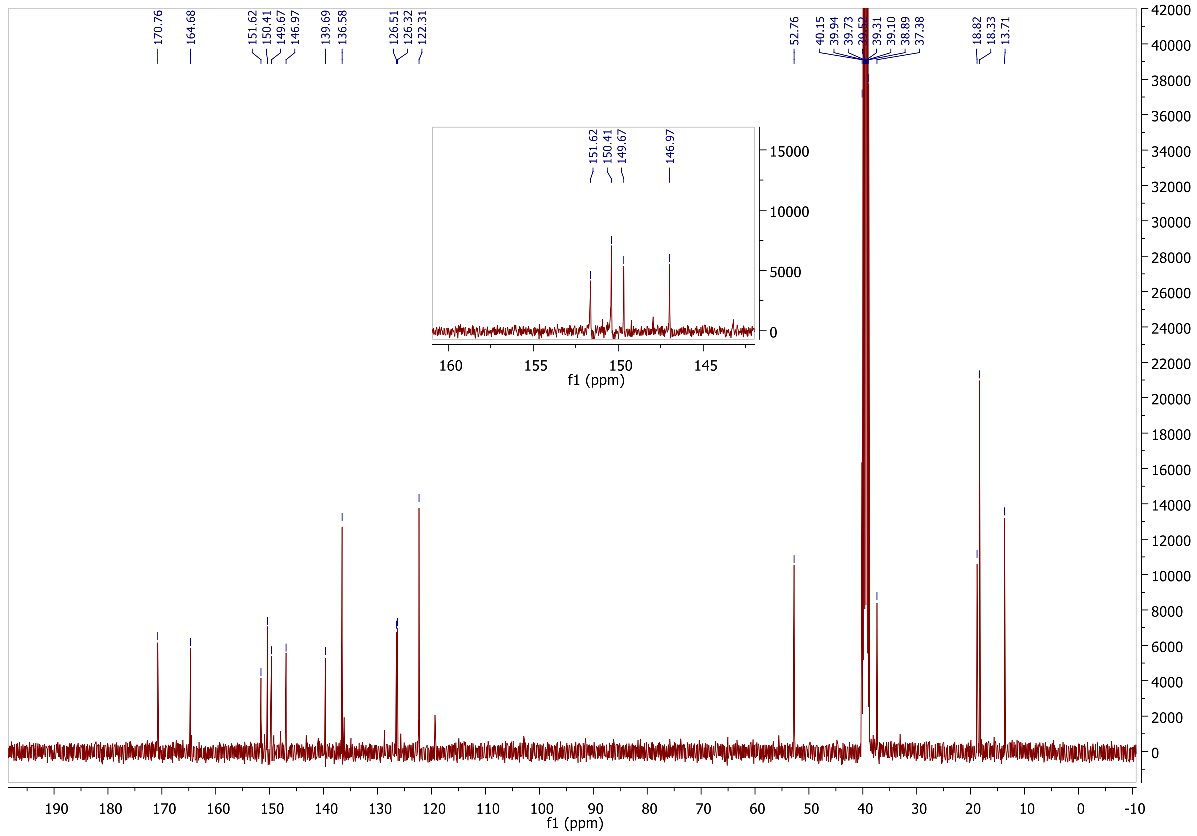

Additional (minor) signals derive from the (*Z*)-photoisomer formed by daylight.

Methyl (*E*)-5-[(4-hexanamido-3,5-dimethylphenyl)diazenyl]nicotinate (**8c**)

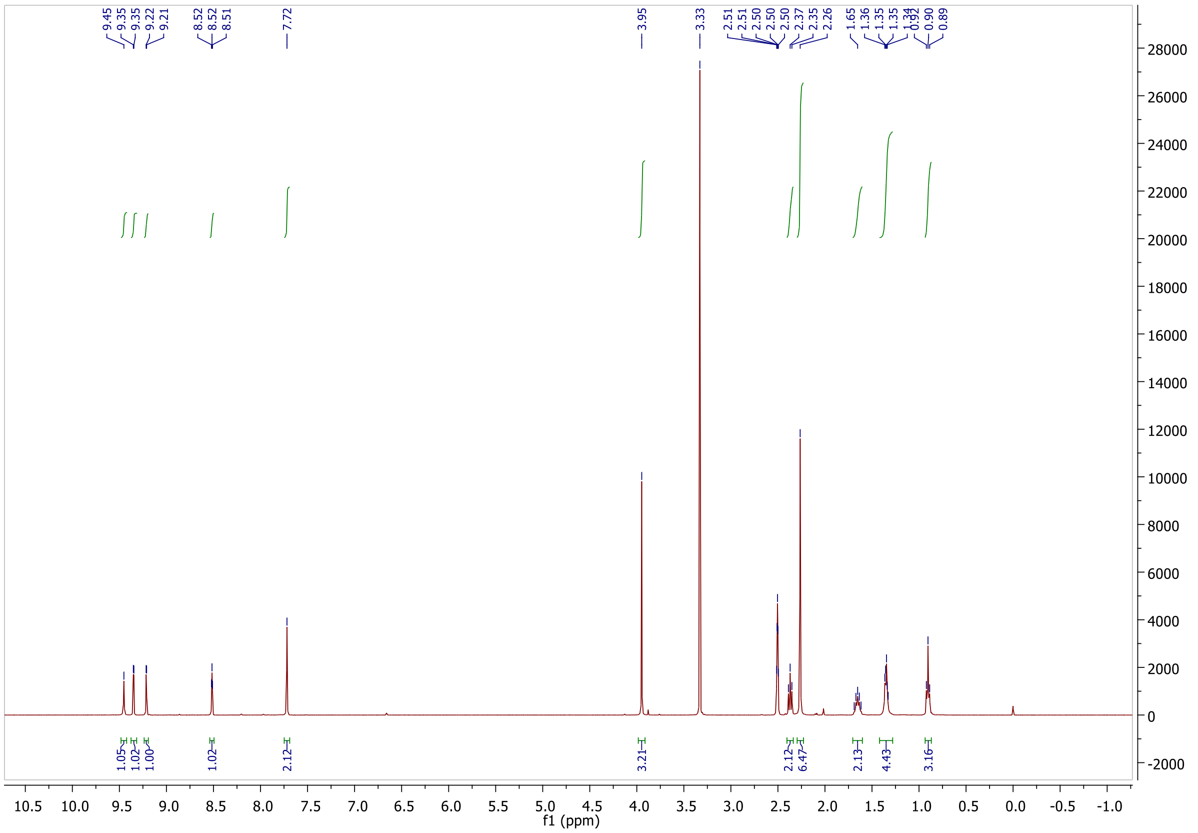

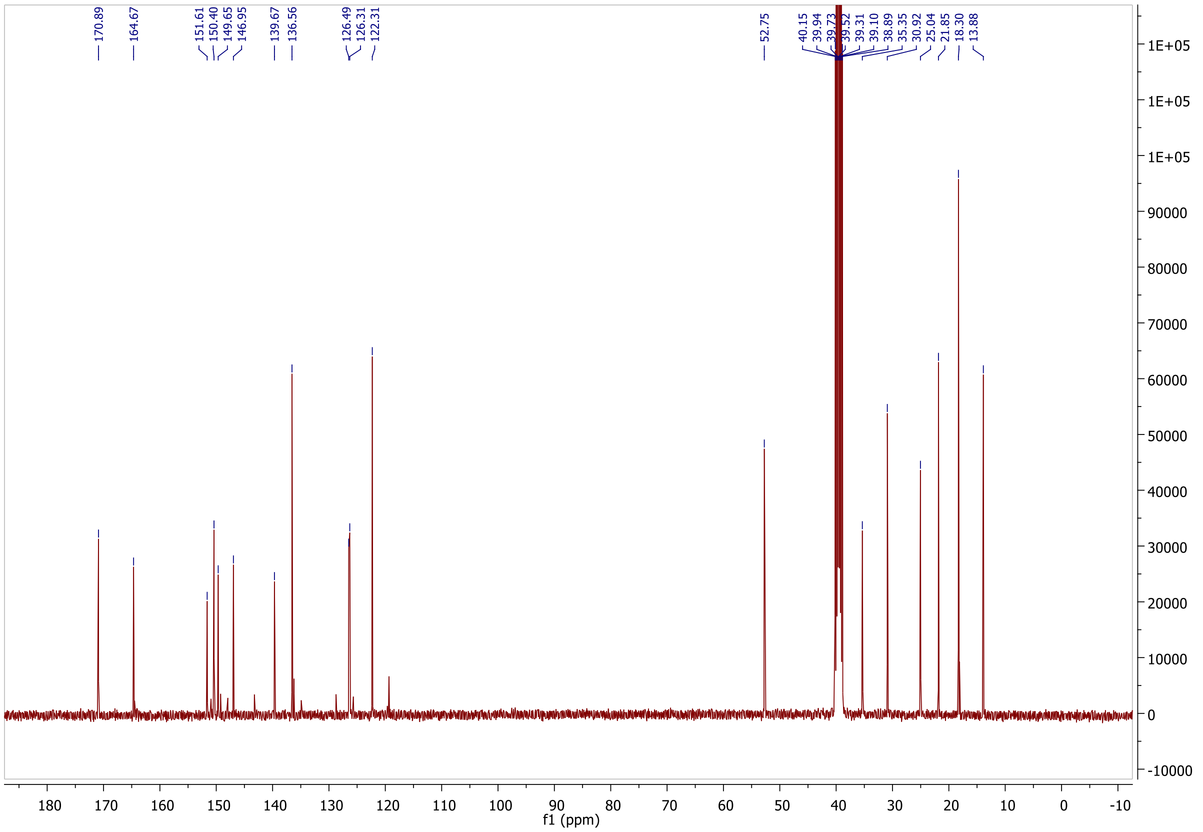

Additional (minor) signals derive from the (*Z*)-photoisomer formed by daylight.

Methyl (*E*)-5-[(3,5-dimethyl-4-octanamidophenyl)diazenyl]nicotinate (**8d**)

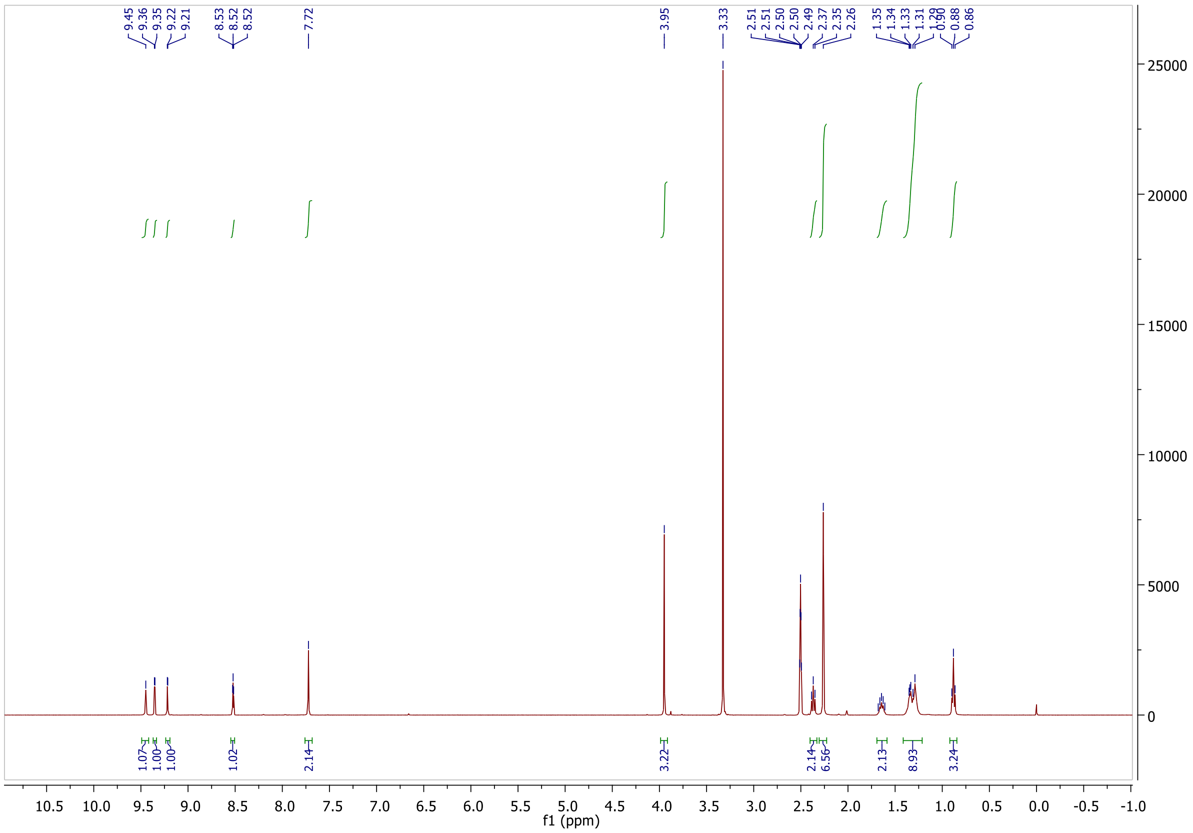

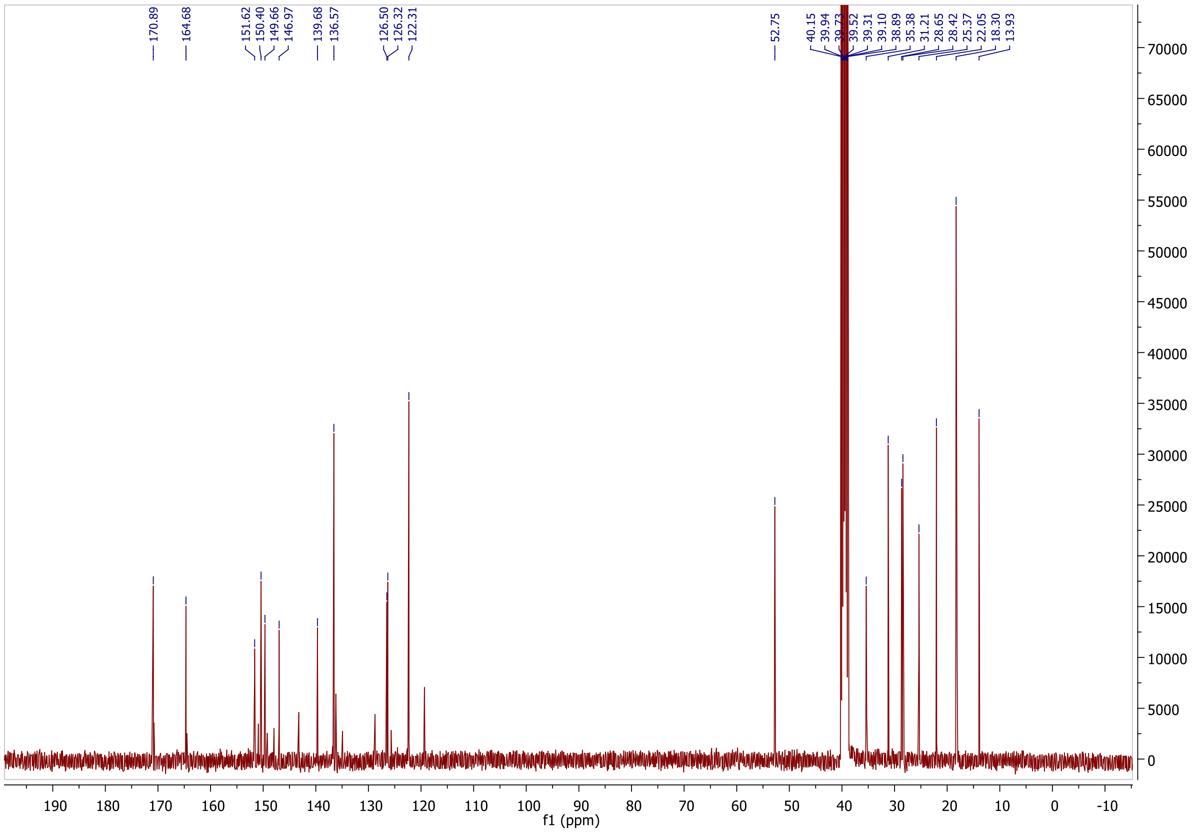

Additional (minor) signals derive from the (*Z*)-photoisomer formed by daylight.

Methyl (*E*)-5-[(3,5-dimethyl-4-decanamidophenyl)diazenyl]nicotinate (**8e**)

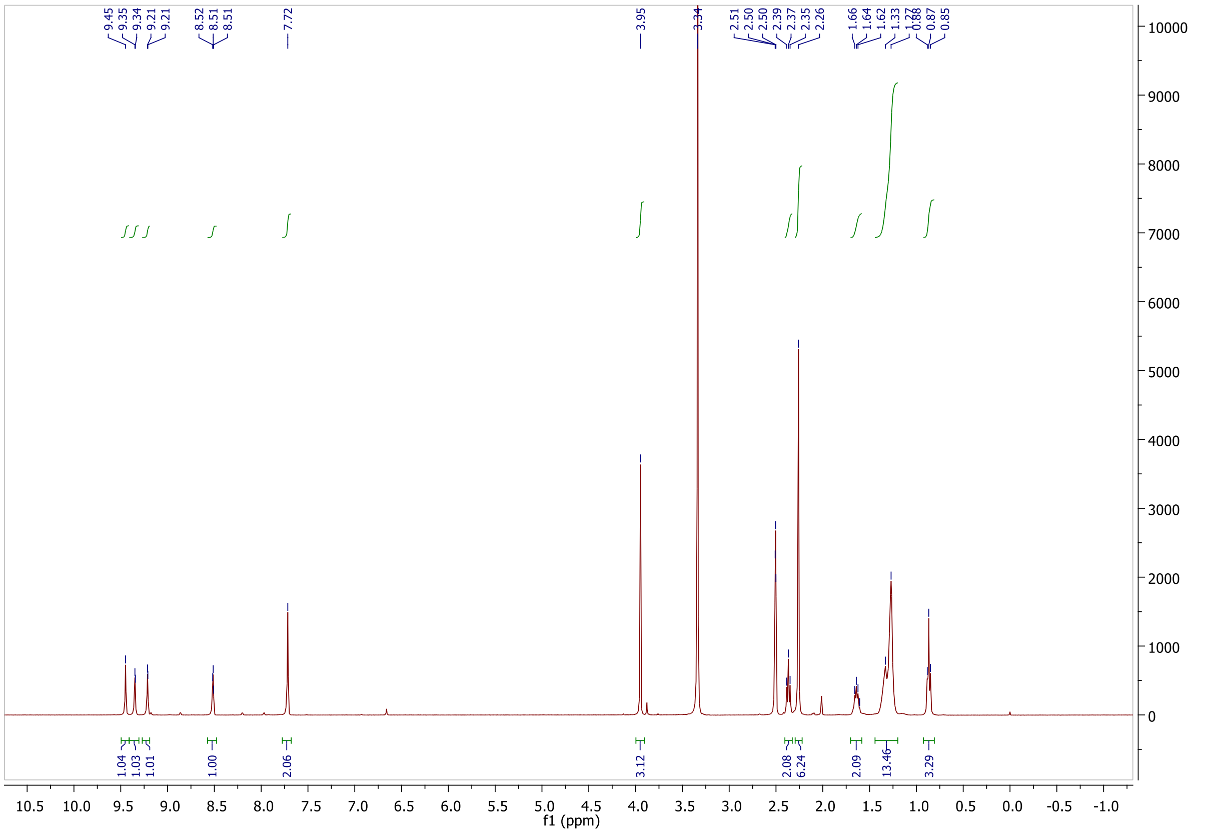

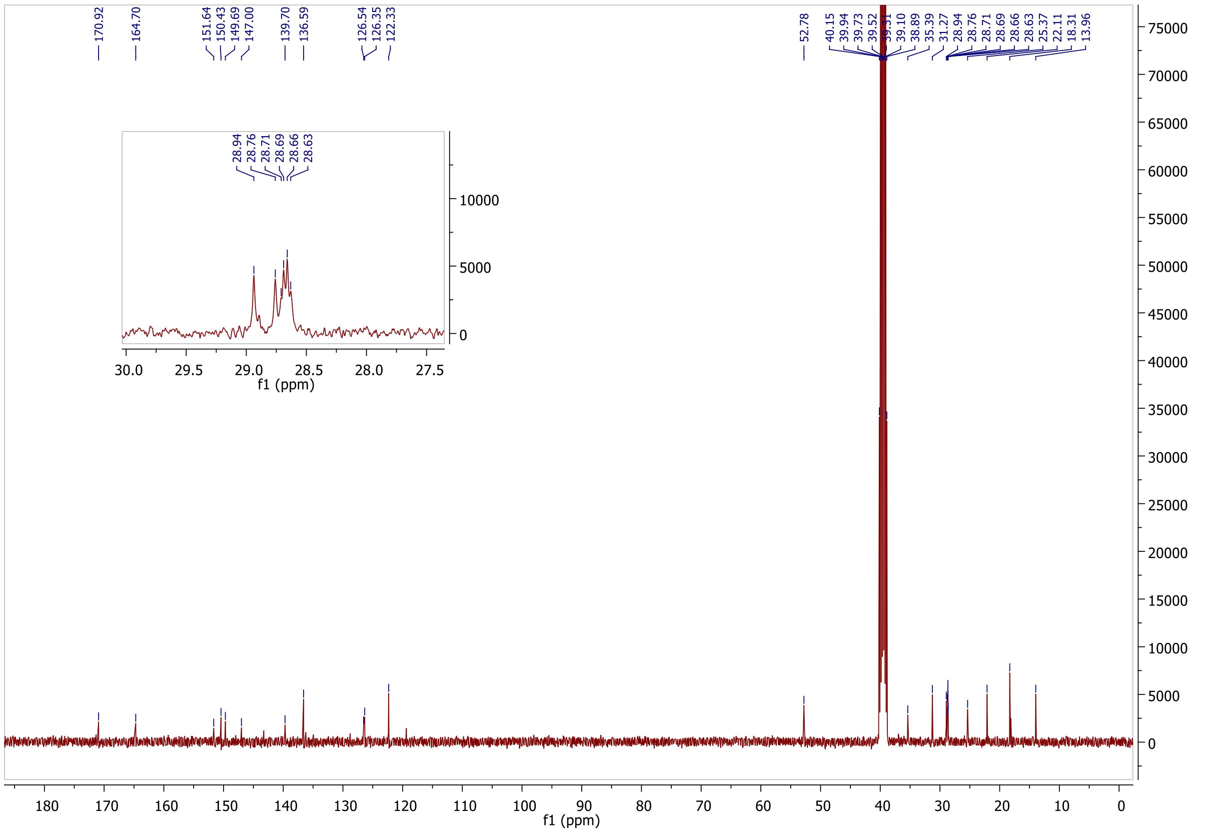

Additional (minor) signals derive from the (*Z*)-photoisomer formed by daylight.

Methyl (*E*)-5-[(4-dodecanamido-3,5-dimethylphenyl)diazenyl]nicotinate (**8f**)

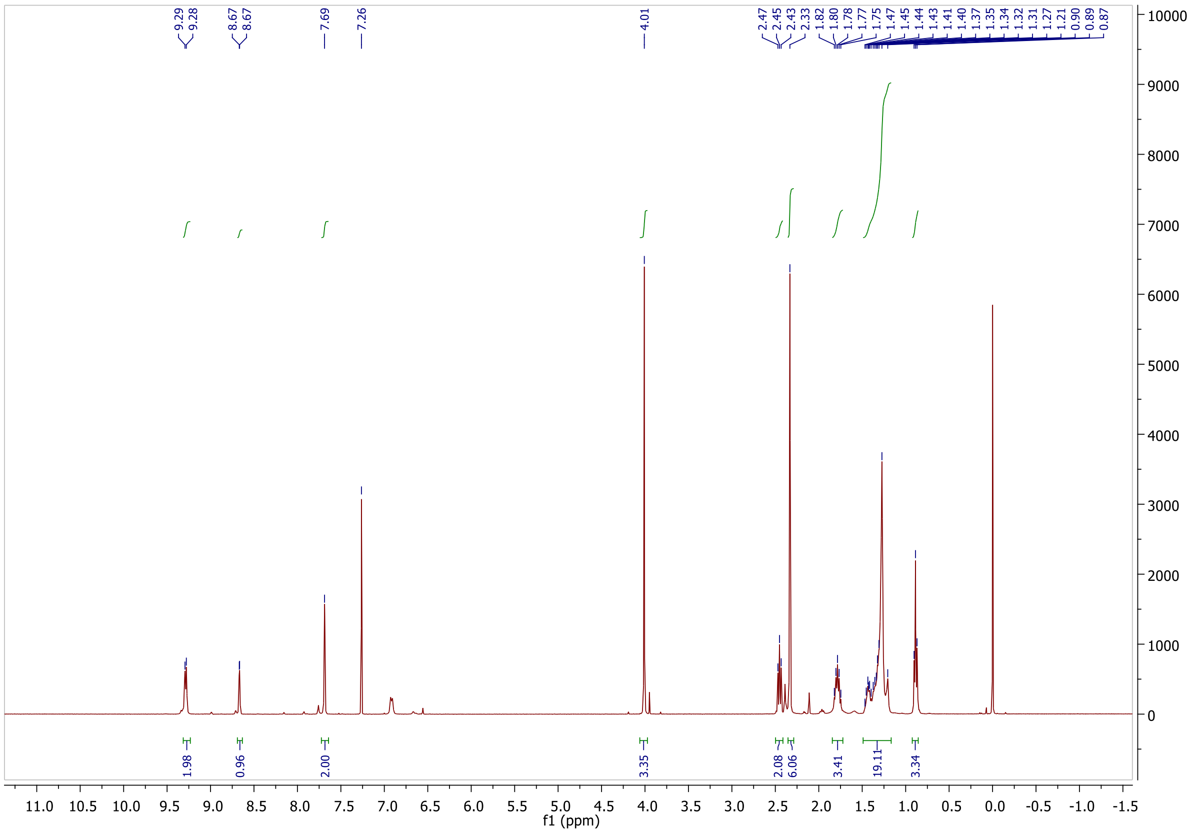

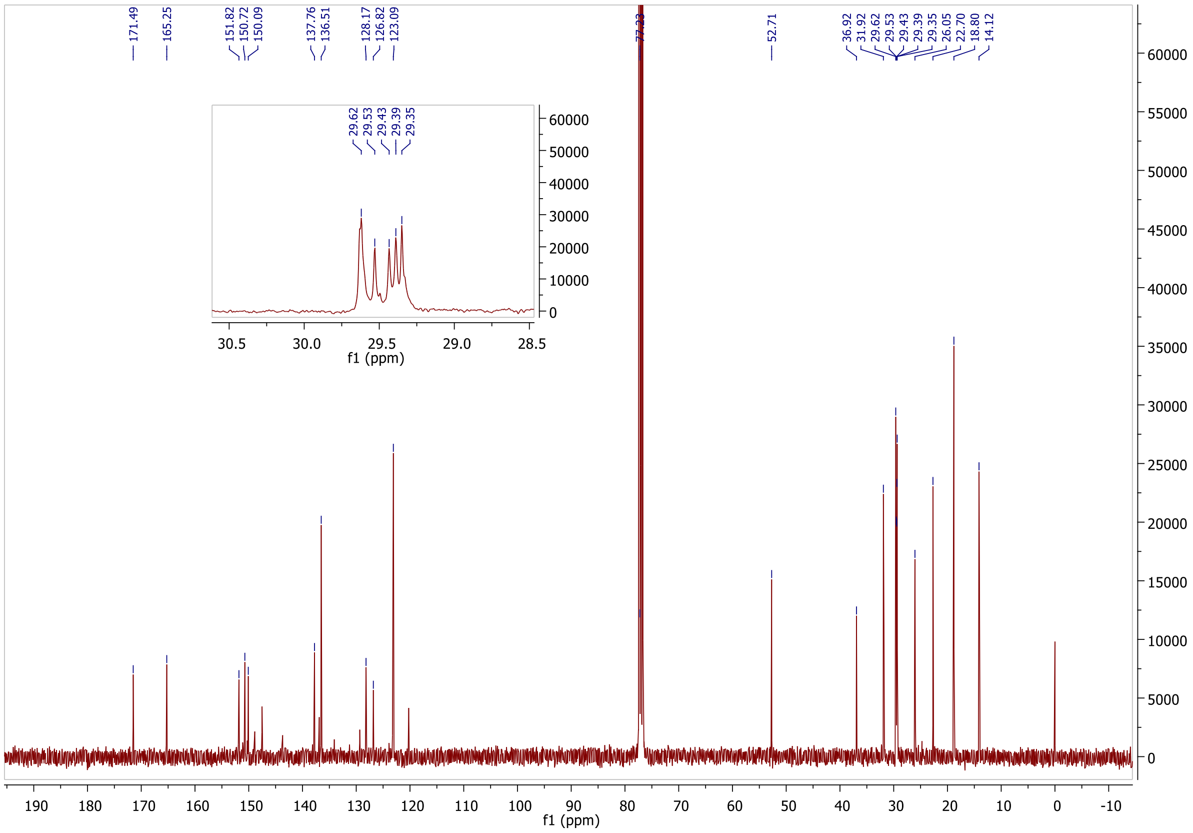

Additional (minor) signals derive from the (*Z*)-photoisomer formed by daylight.

Ethyl (*E*)-5-[(4-amino-3,5-dimethylphenyl)diazenyl]nicotinate (**11**)

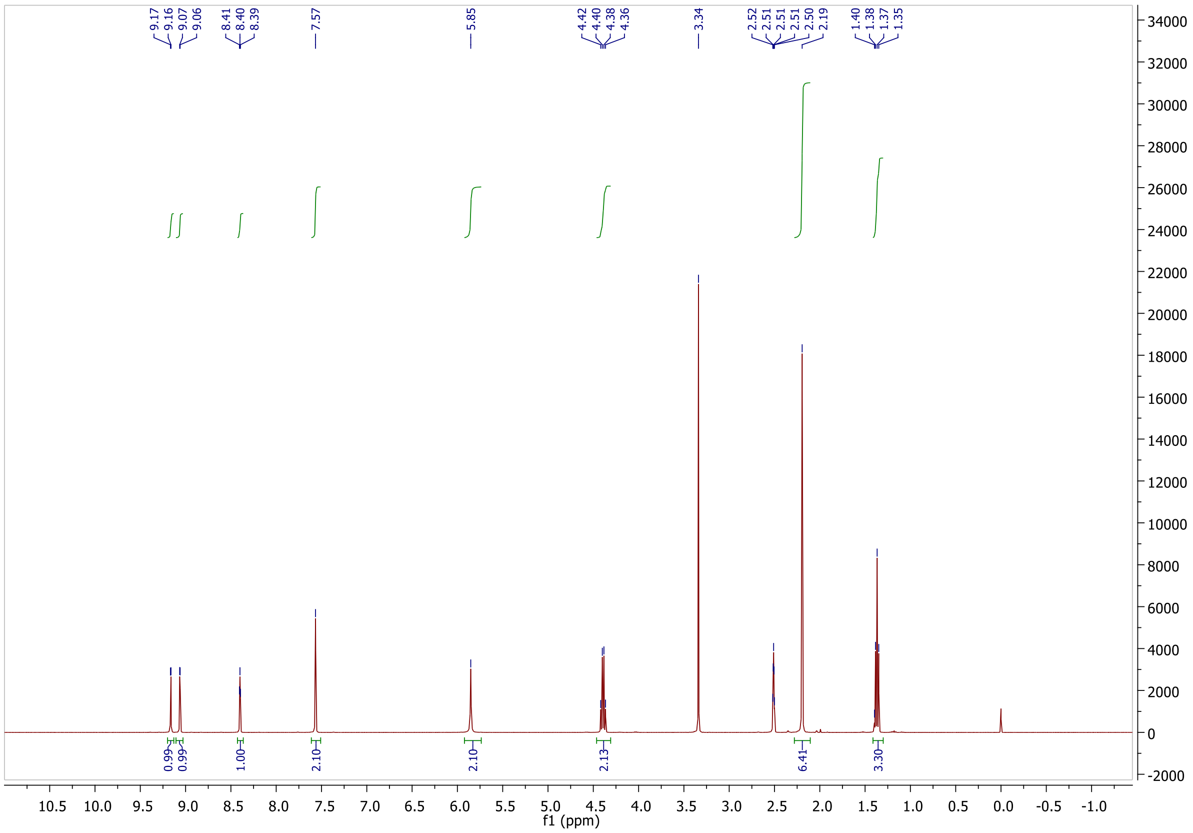

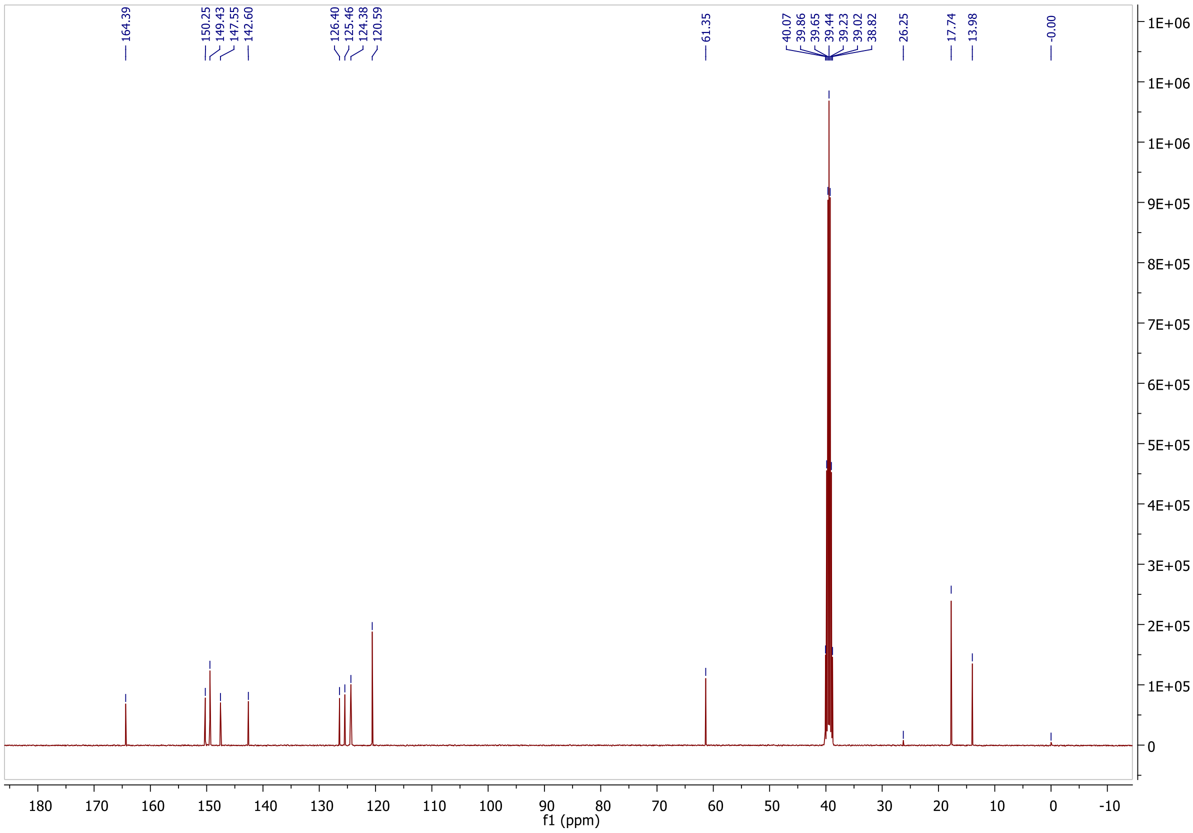

Ethyl (*E*)-5-[(3,5-dimethyl-4-tetradecanamidophenyl)diazenyl]nicotinate (**8g**)

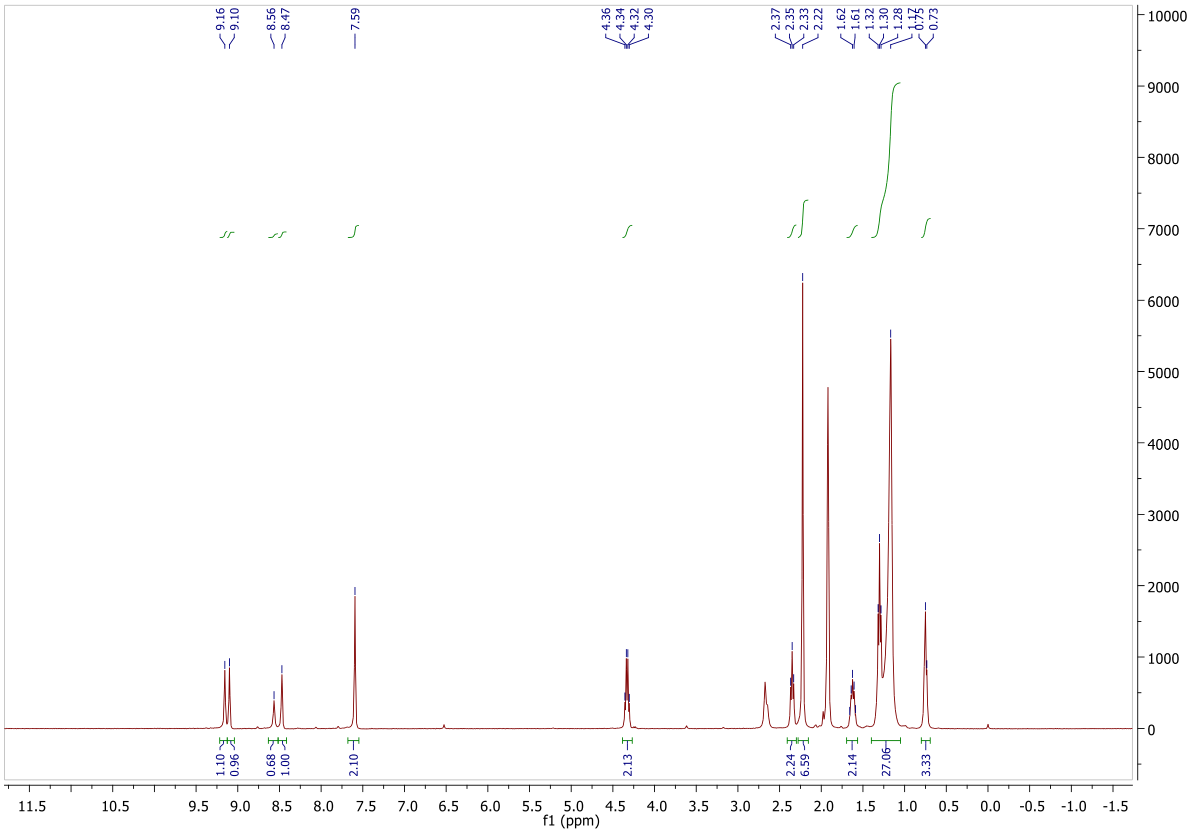

Methyl (*E*)-5-[(4-benzamido-3,5-dimethylphenyl)diazenyl]nicotinate (**8h**)

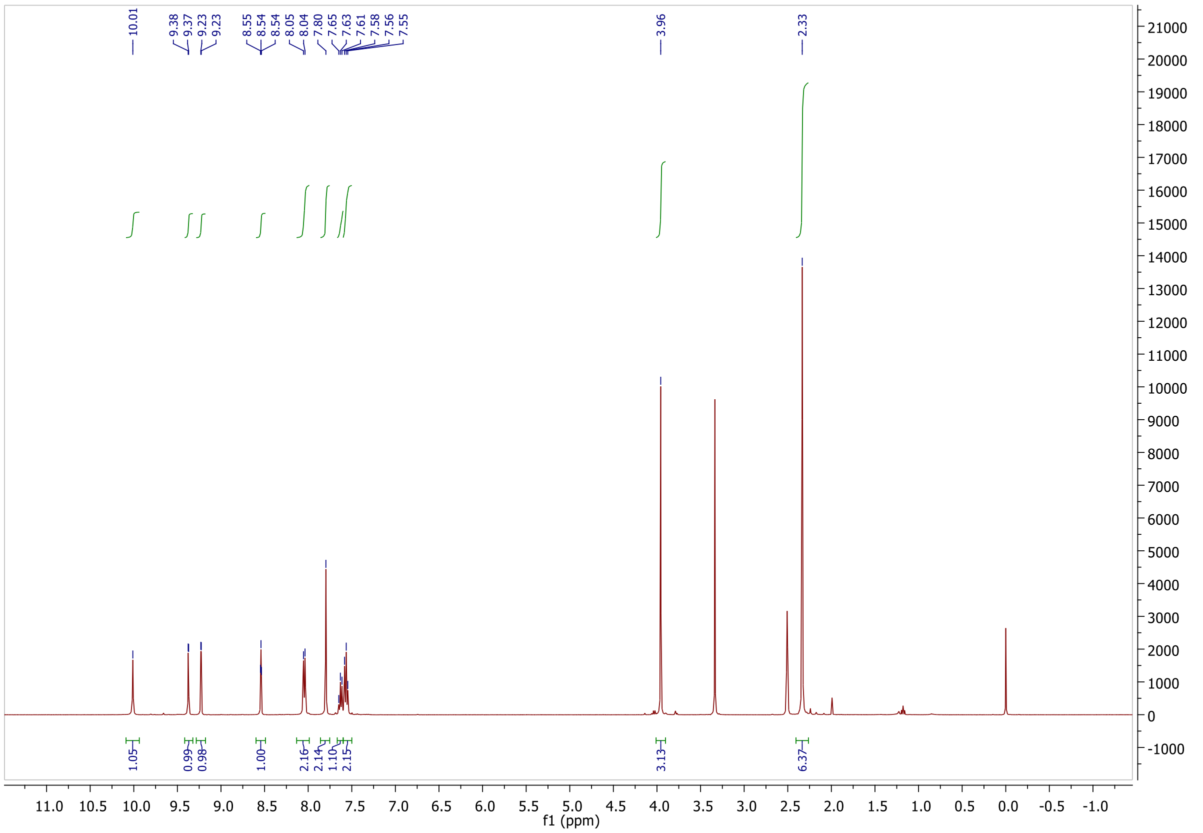

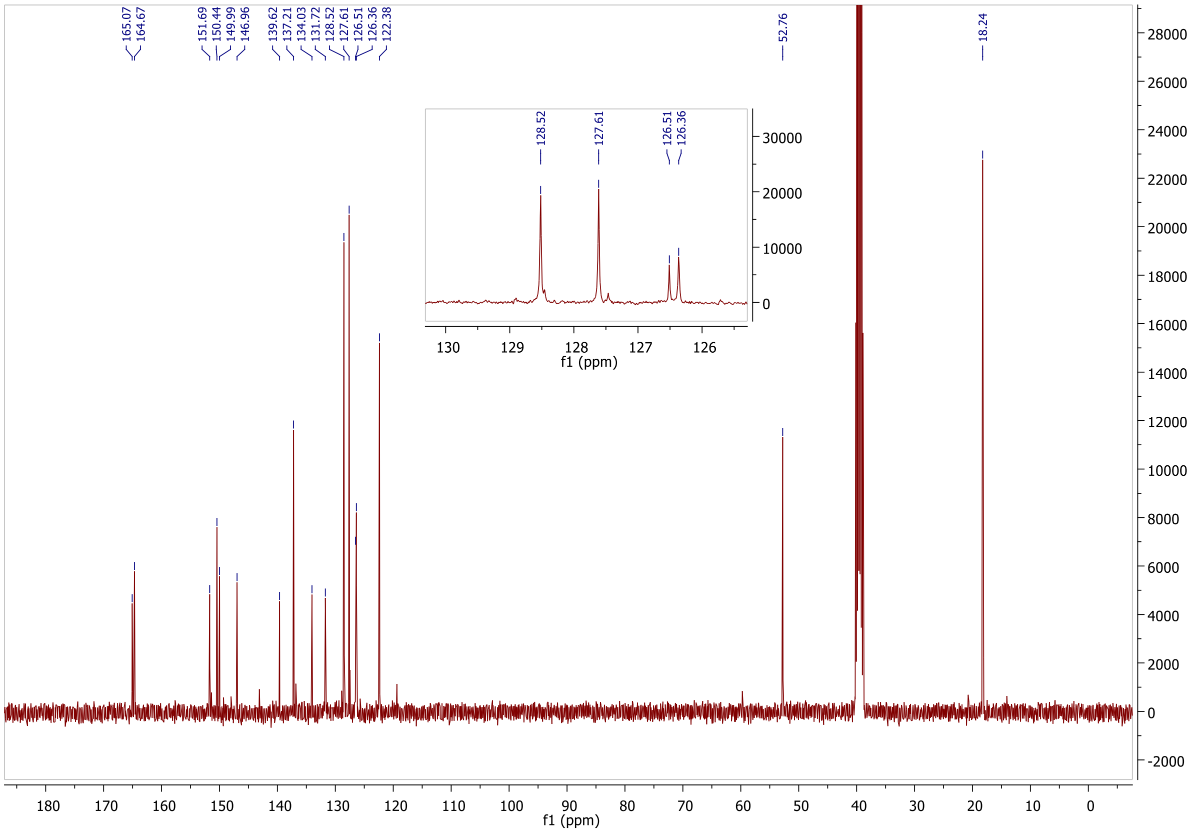

Additional (minor) signals derive from the (*Z*)-photoisomer formed by daylight.

Methyl (*E*)-5-{[4-(4-fluorobenzamido)-3,5-dimethylphenyl]diazenyl}nicotinate (**8i**)

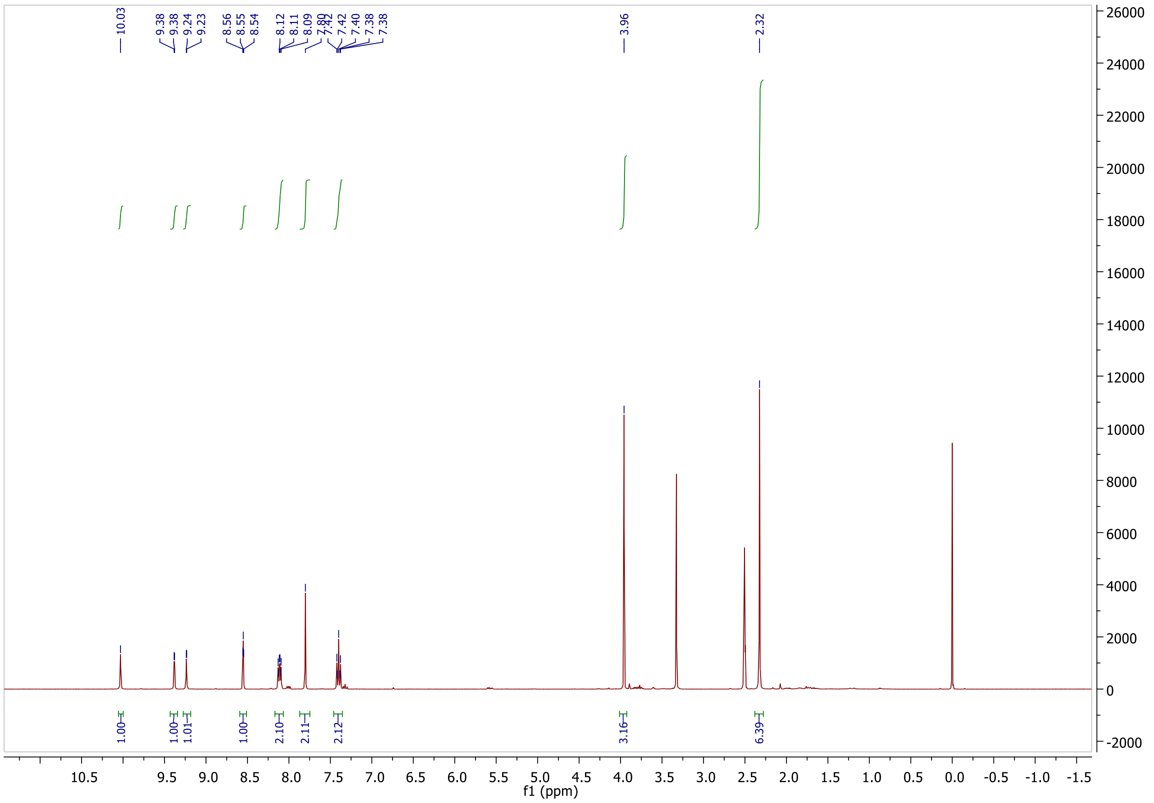

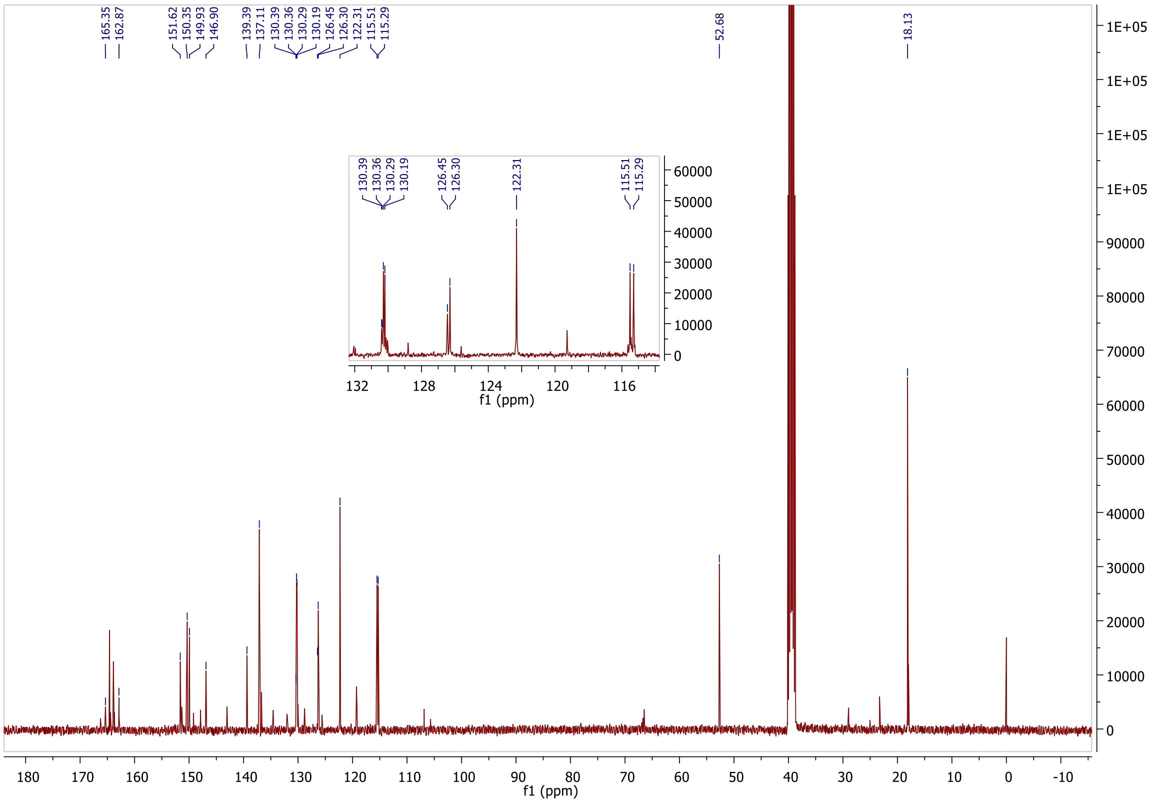

Additional (minor) signals derive from the (*Z*)-photoisomer formed by daylight.

Methyl (*E*)-5-({3,5-dimethyl-4-[4-(thiophen-2-yl)benzamido]phenyl}-diazenyl)nicotinate (**8j**)

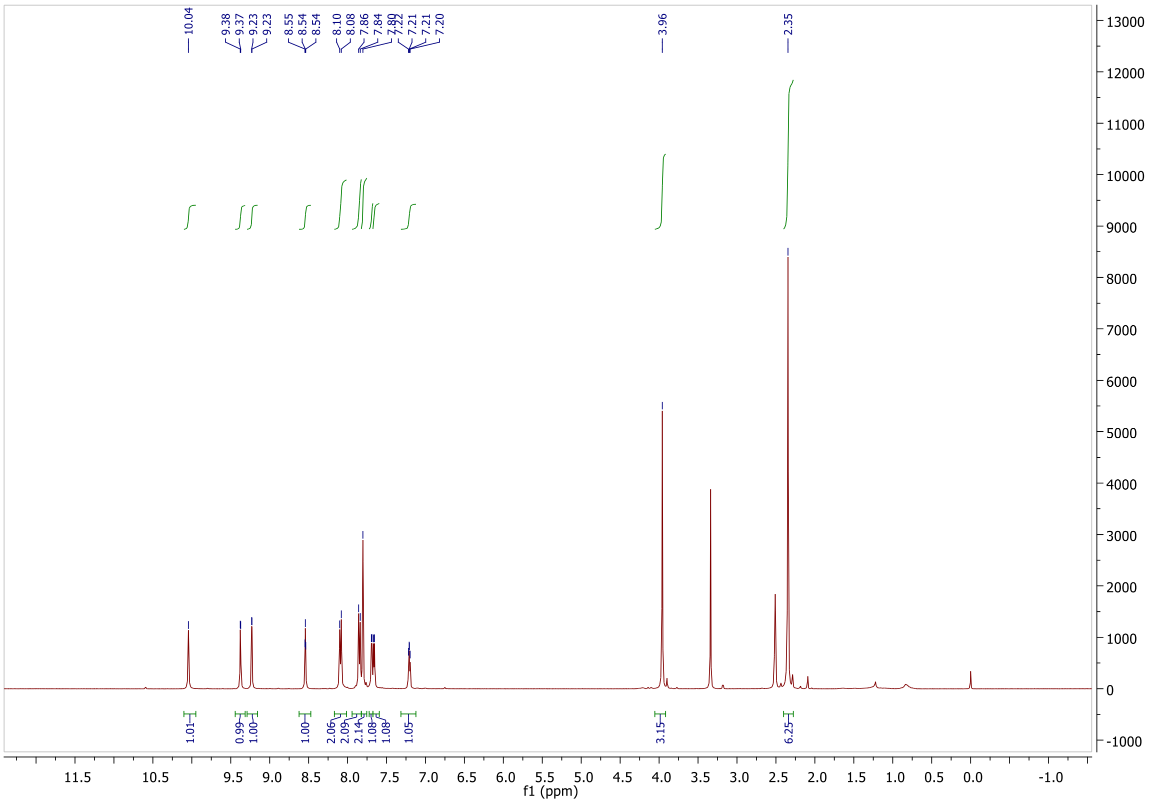

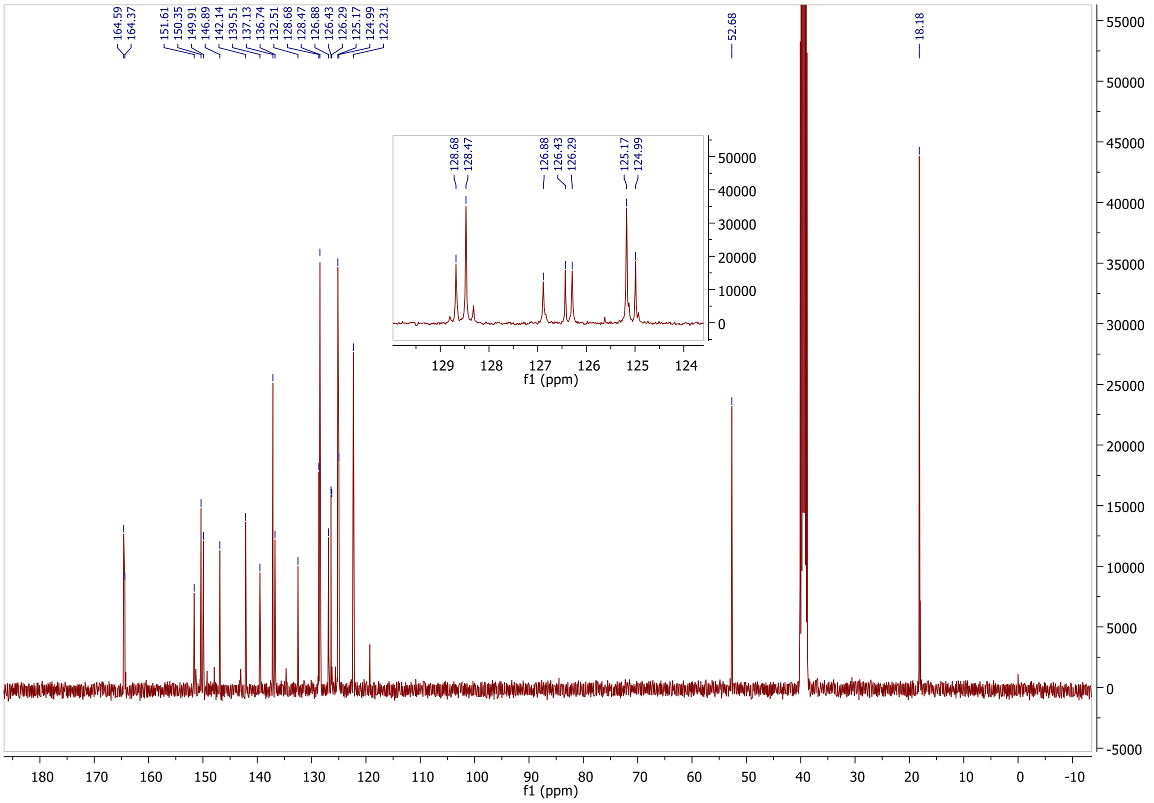

Additional (minor) signals derive from the (*Z*)-photoisomer formed by daylight.

Methyl (*E*)-5-{[4-(adamantane-1-carboxamido)-3,5-dimethylphenyl]diazenyl}nicotinate (**8k**)

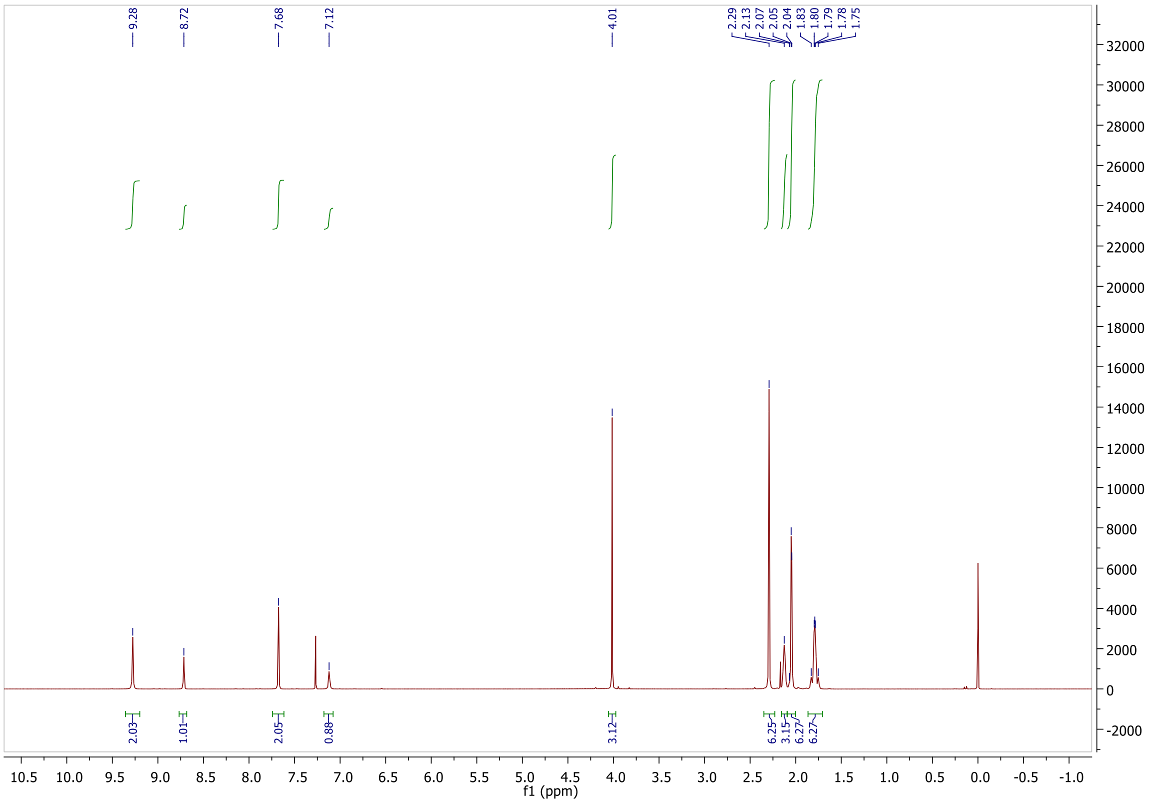

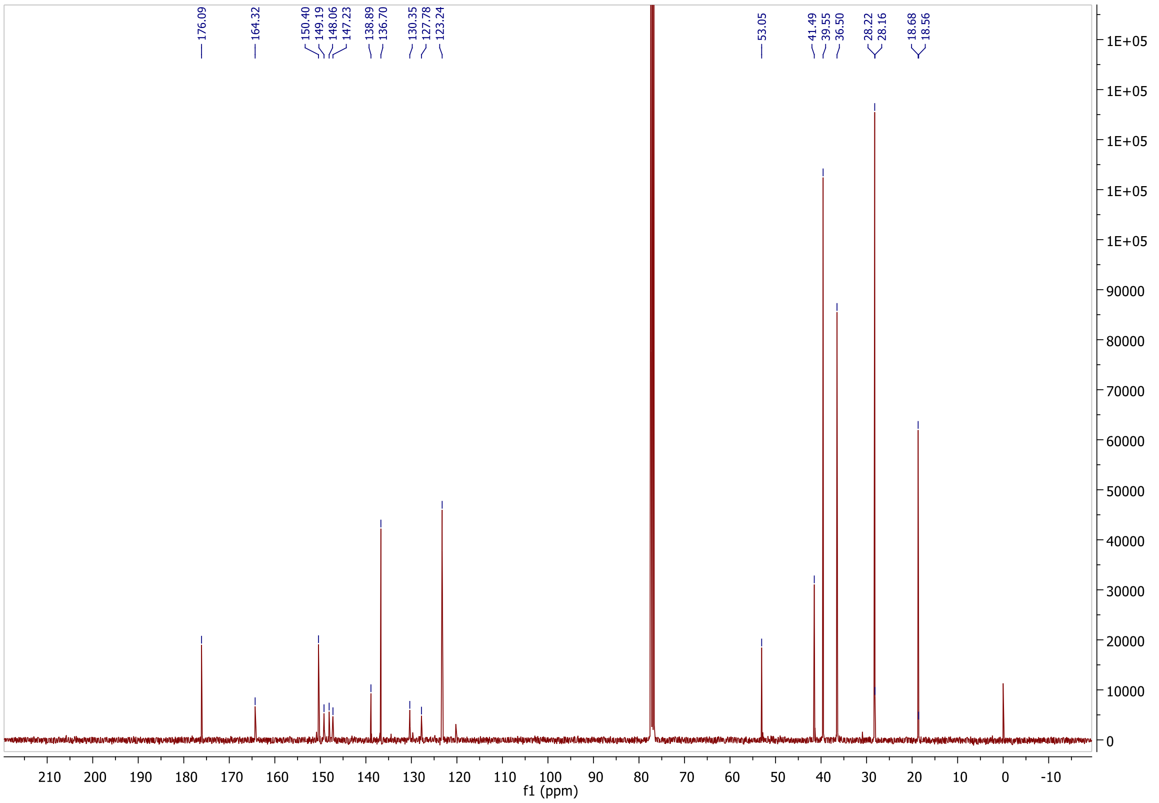

Additional (minor) signals derive from the (*Z*)-photoisomer formed by daylight.

(*E*)-5-[(4-Acetamido-3,5-dimethylphenyl)diazenyl]nicotinamide (**9a**)

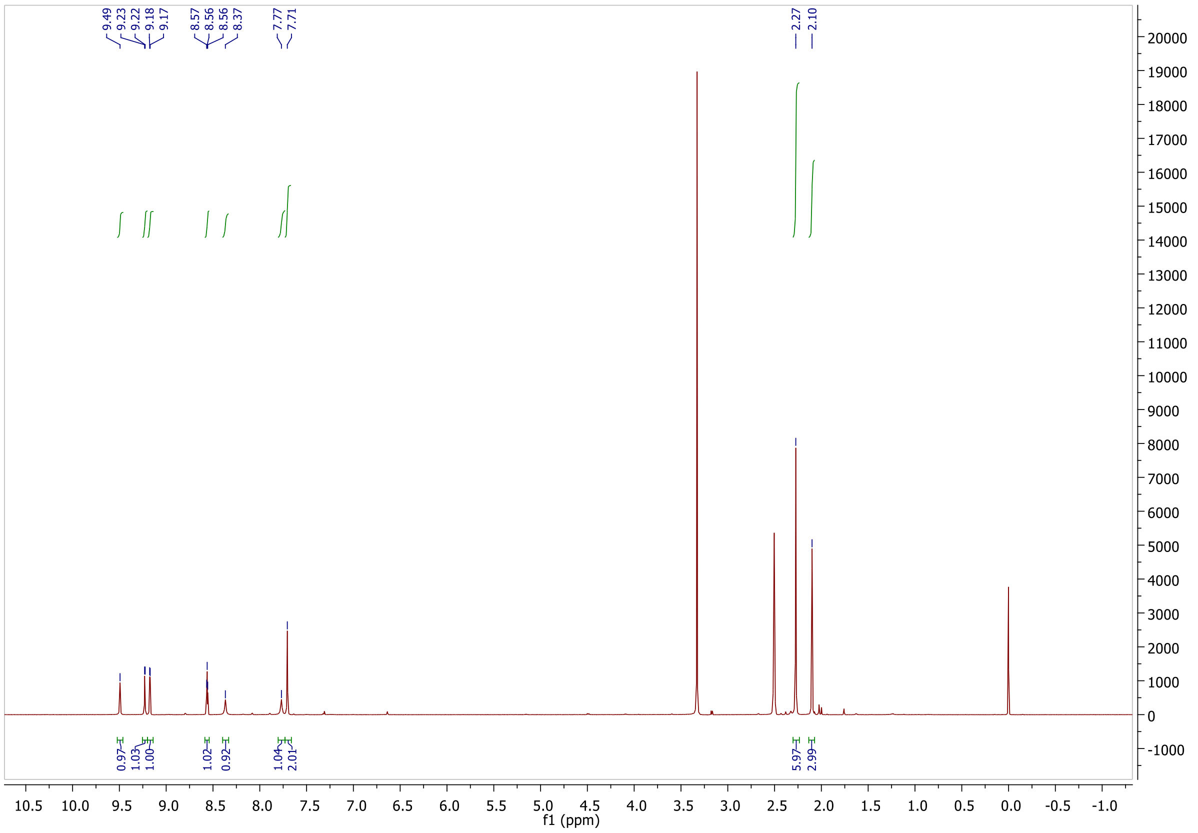

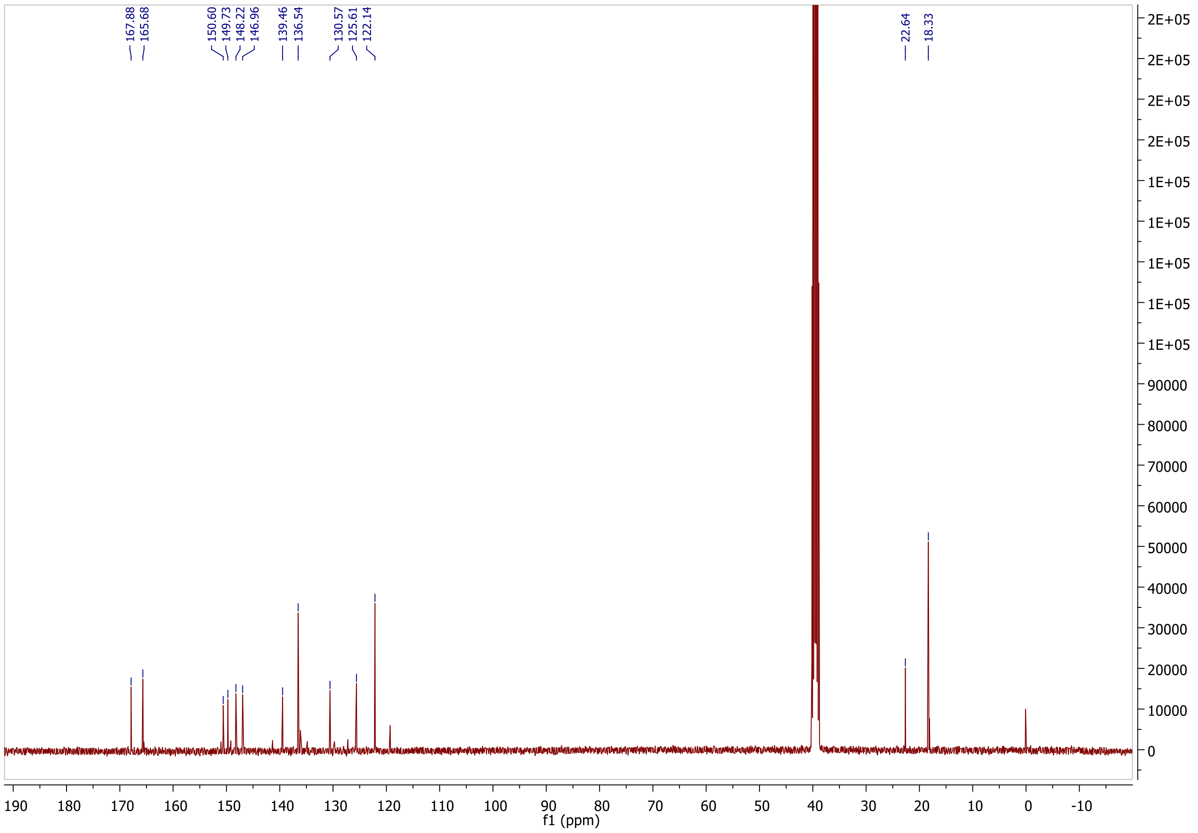

Additional (minor) signals derive from the (*Z*)-photoisomer formed by daylight.

(*E*)-5-[(4-Butyramido-3,5-dimethylphenyl)diazenyl]nicotinamide (**9b**)

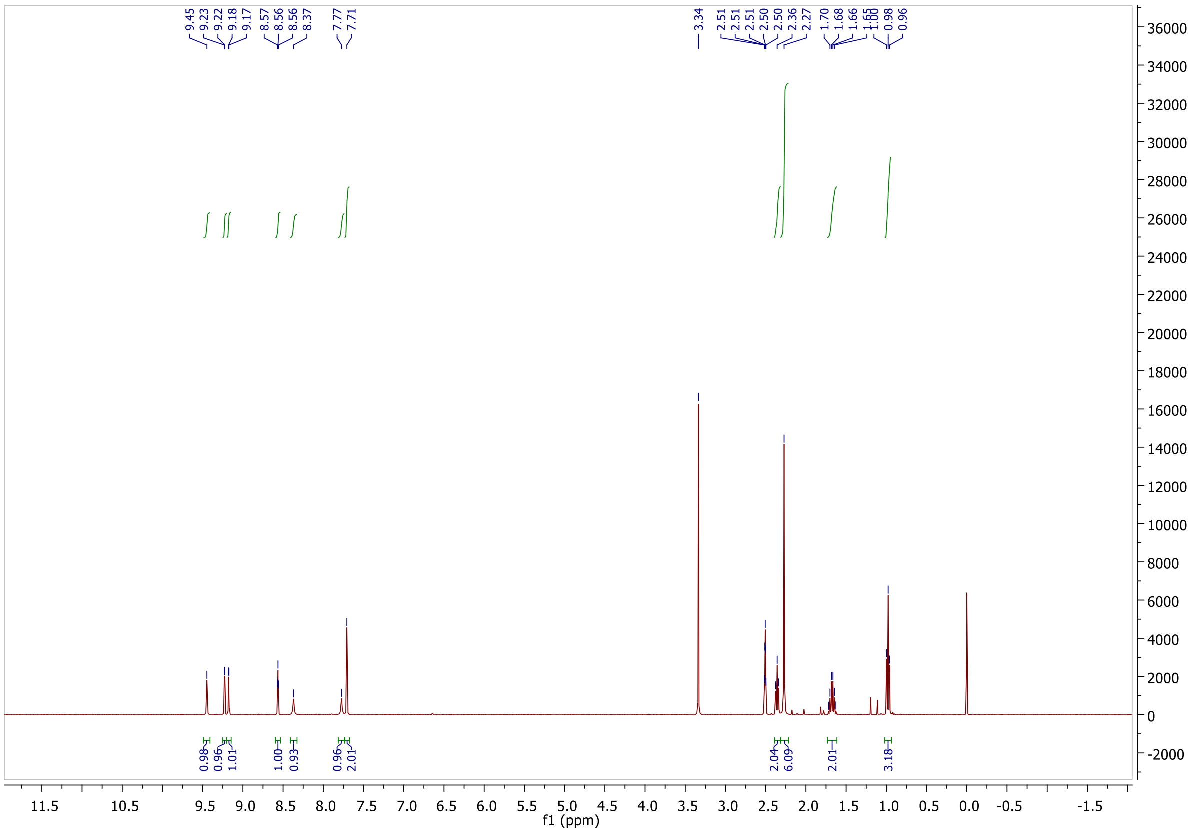

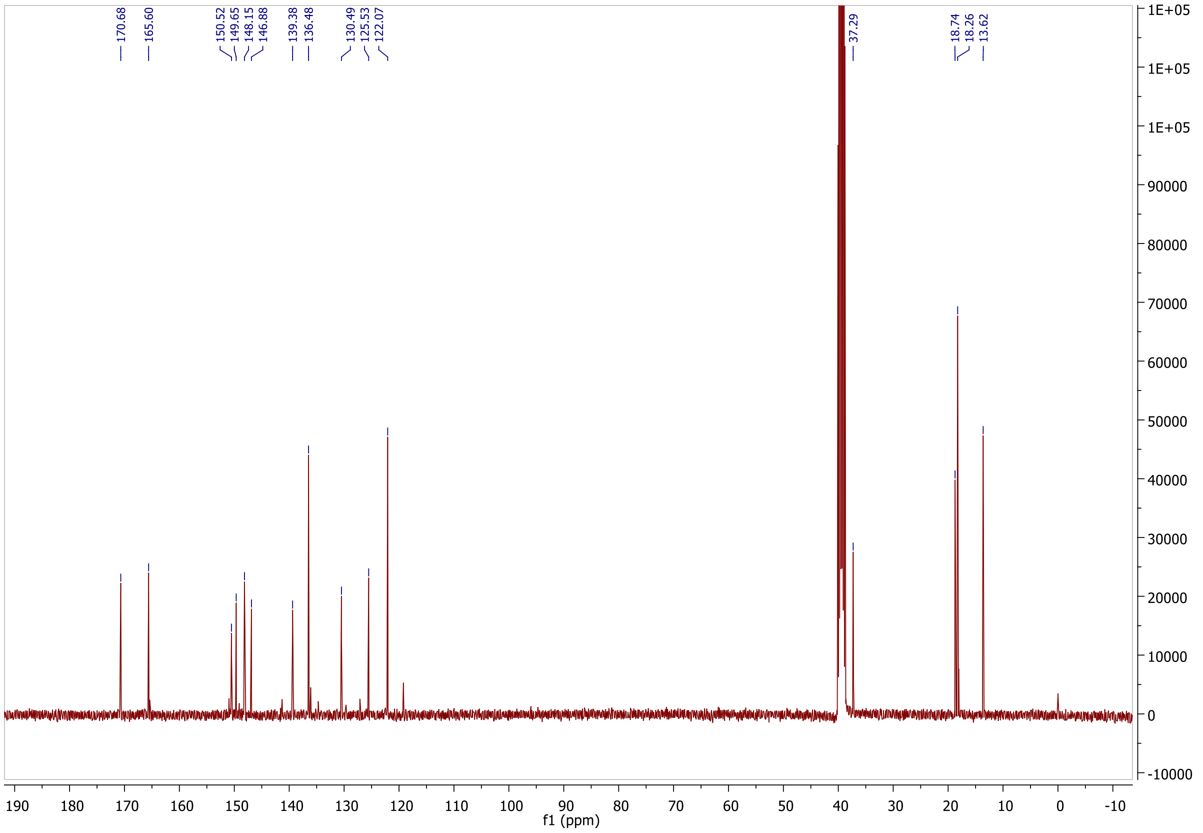

Additional (minor) signals derive from the (*Z*)-photoisomer formed by daylight.

(*E*)-5-[(4-Hexanamido-3,5-dimethylphenyl)diazenyl]nicotinamide (**9c**)

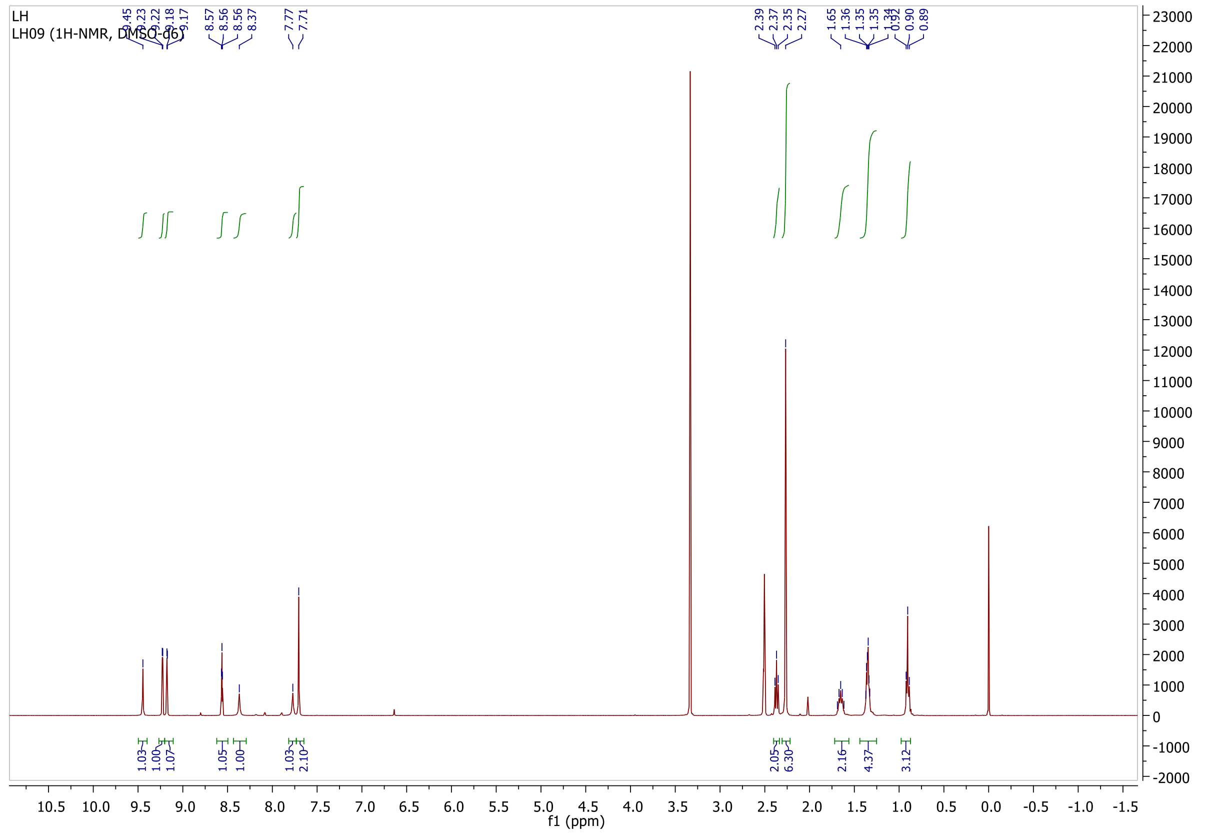

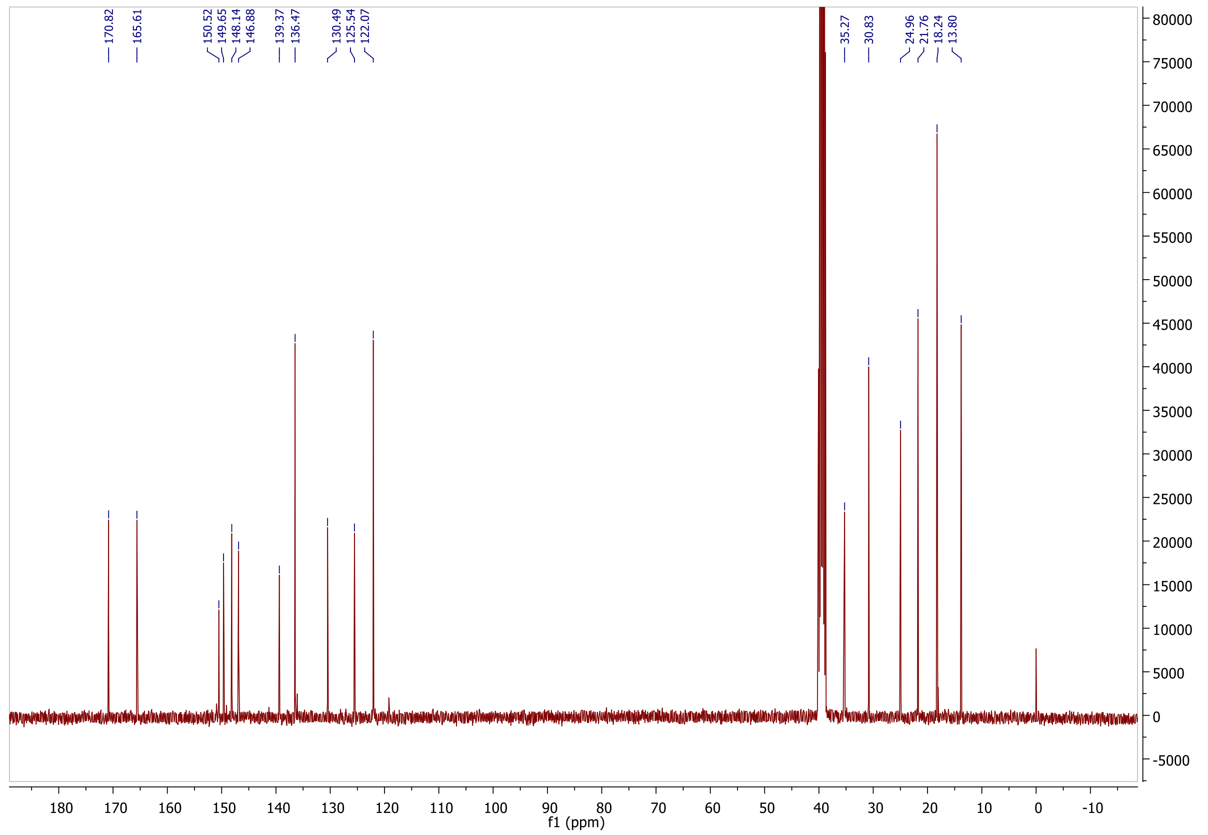

Additional (minor) signals derive from the (*Z*)-photoisomer formed by daylight.

(*E*)-5-[(3,5-Dimethyl-4-octanamidophenyl)diazenyl]nicotinamide (**9d**)

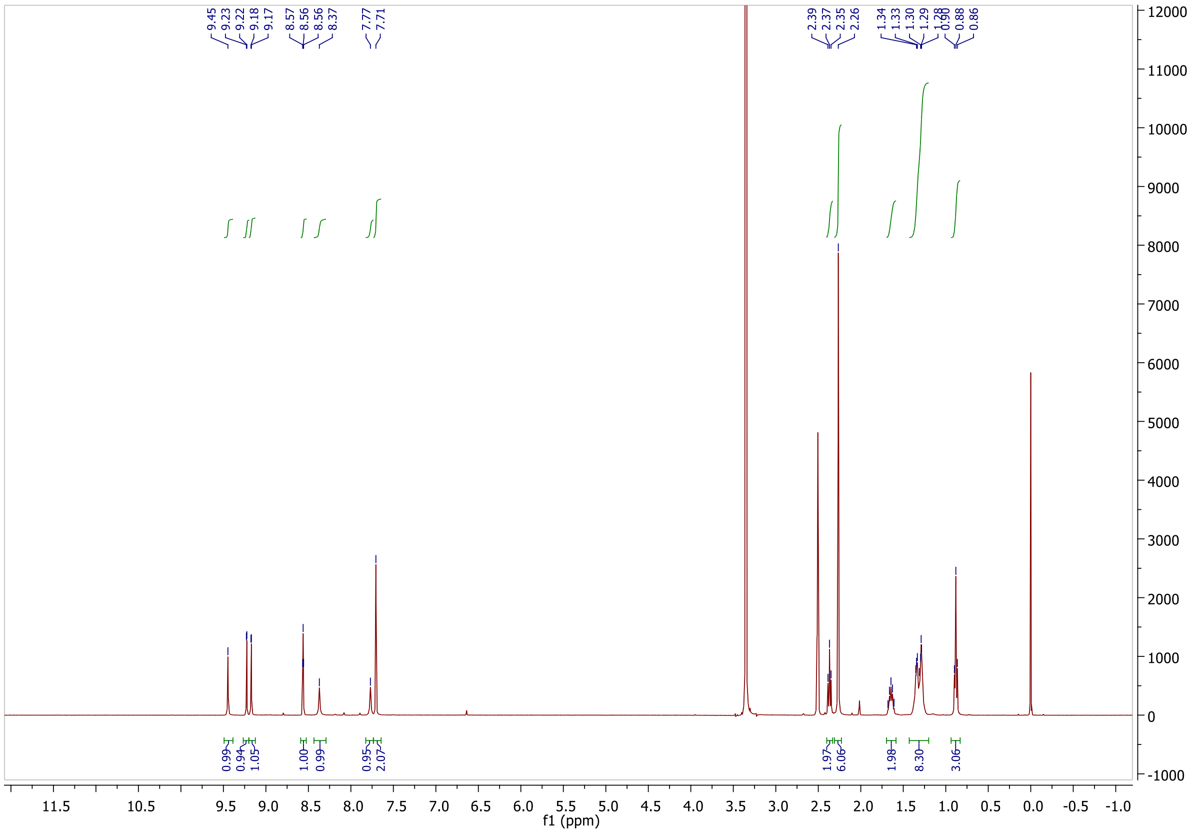

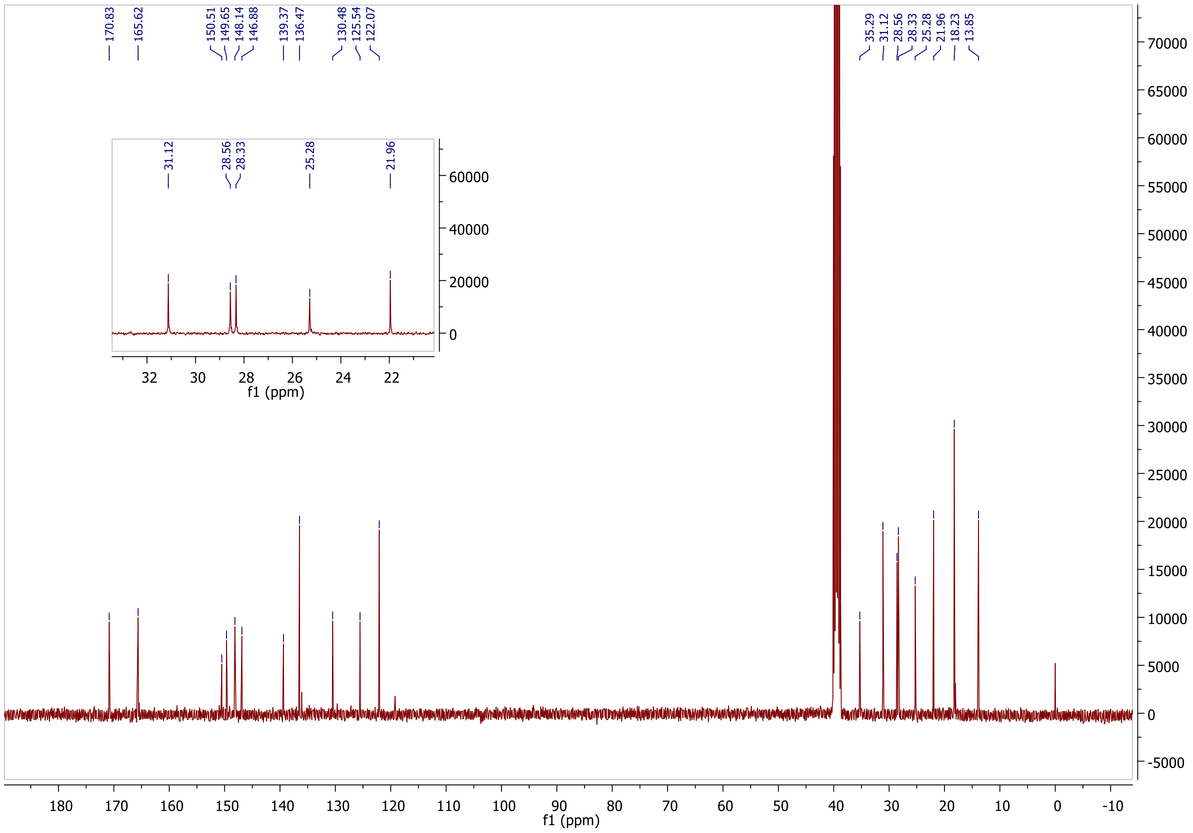

Additional (minor) signals derive from the (*Z*)-photoisomer formed by daylight.

(*E*)-5-[(4-Decanamido-3,5-dimethylphenyl)diazenyl]nicotinamide (**9e**)

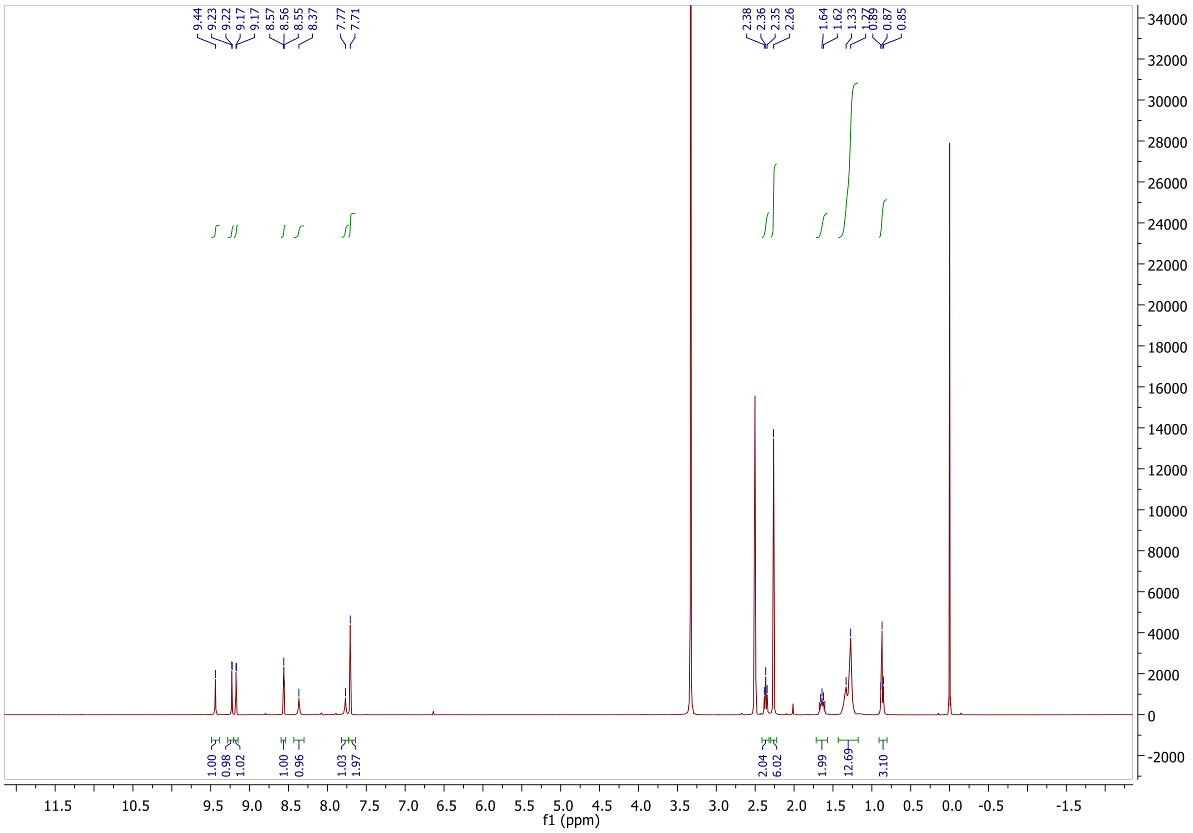

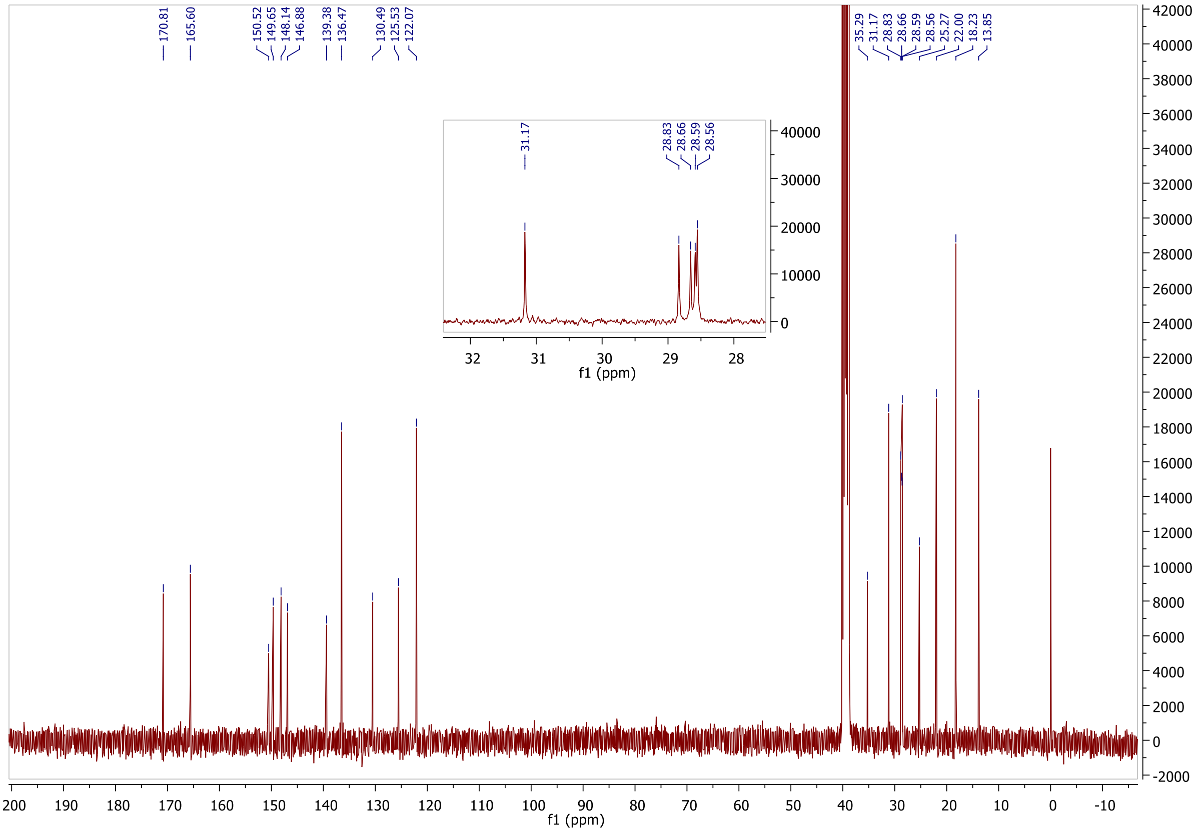

(*E*)-5-[(4-Dodecanamido-3,5-dimethylphenyl)diazenyl]nicotinamide (**9f**)

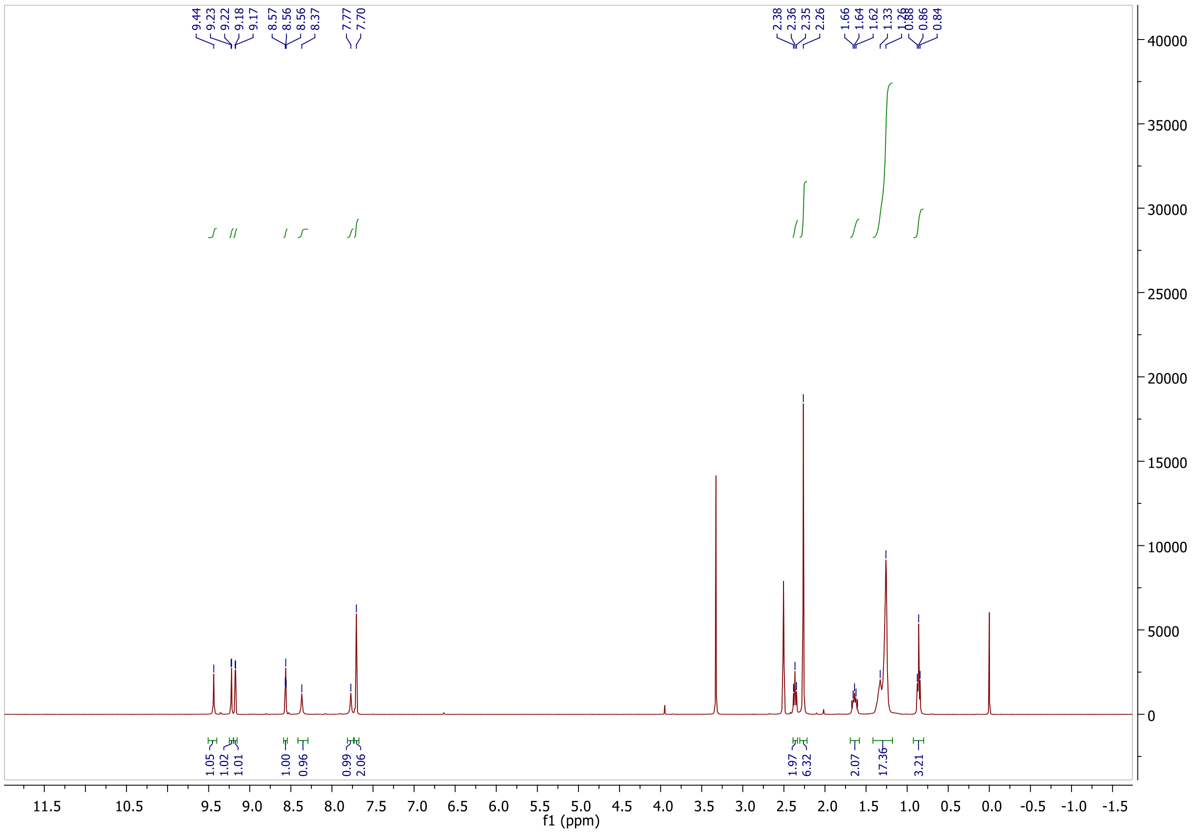

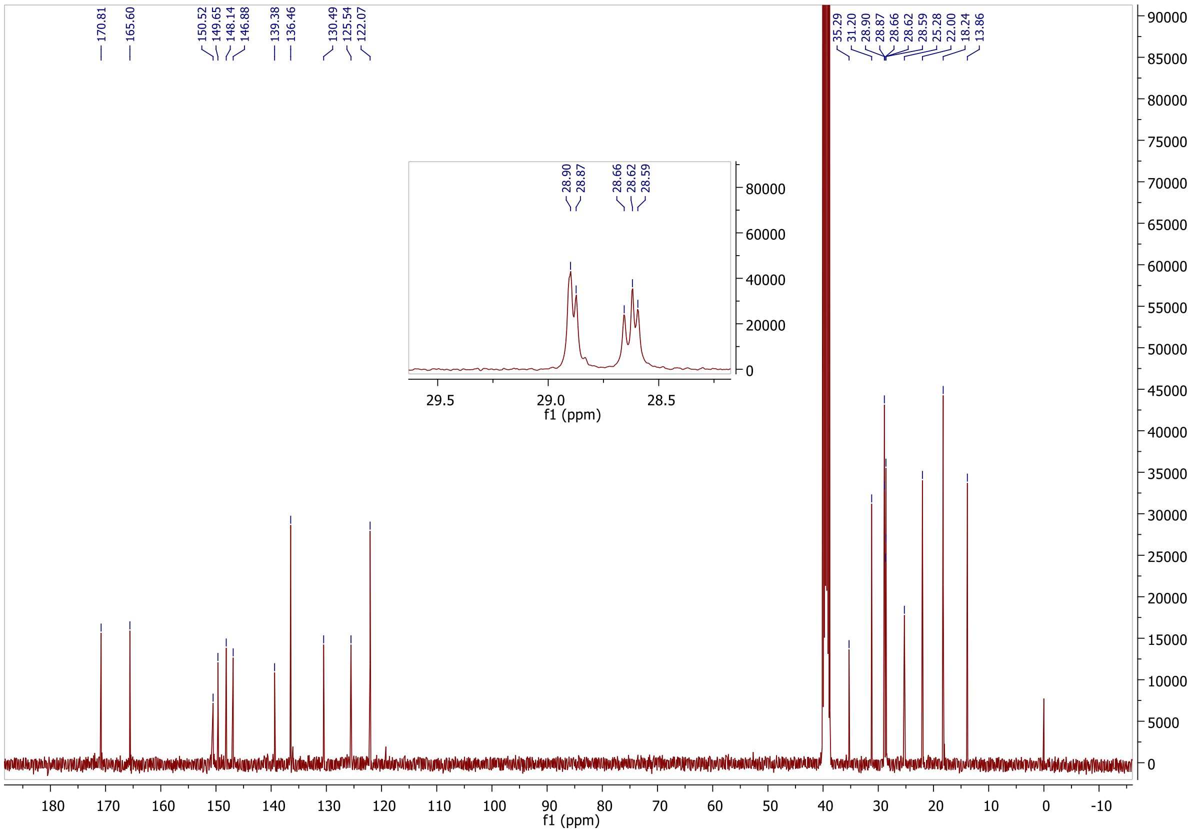

(*E*)-5-[(3,5-Dimethyl-4-tetradecanamidophenyl)diazenyl)]nicotinamide (**9g**)

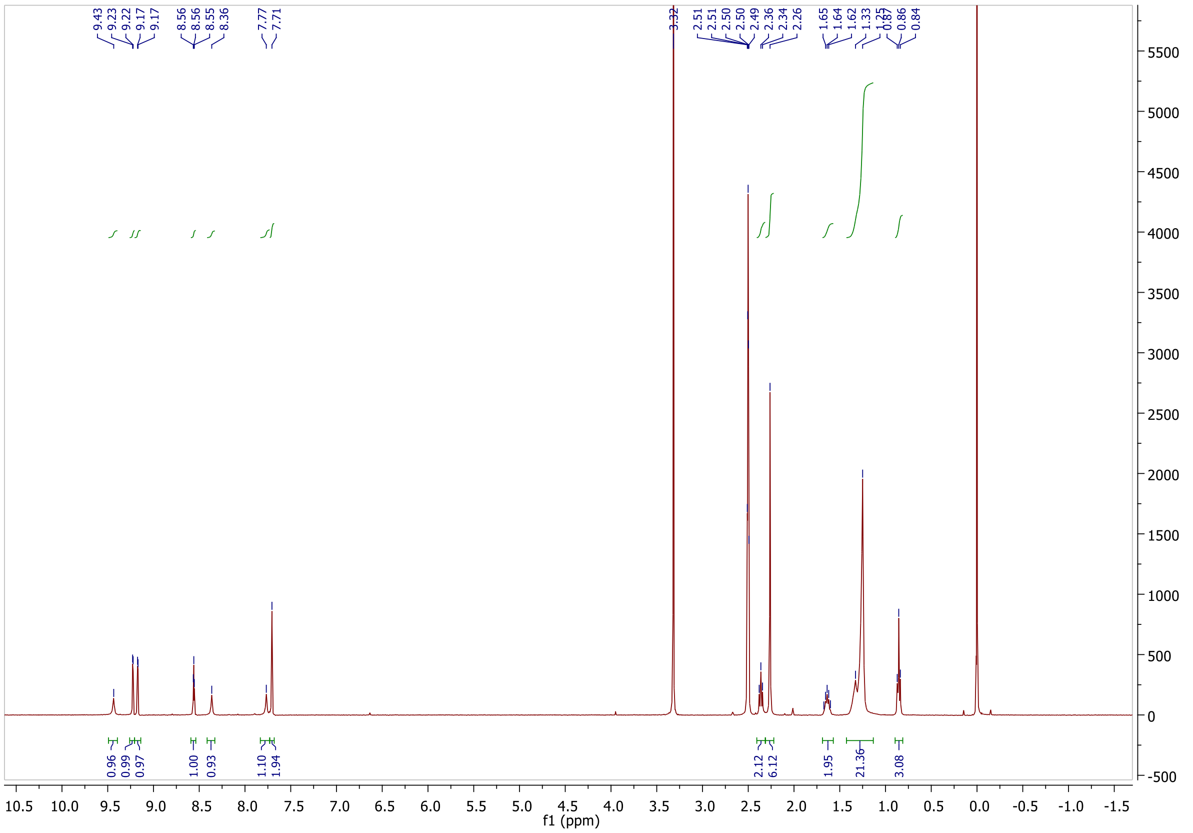

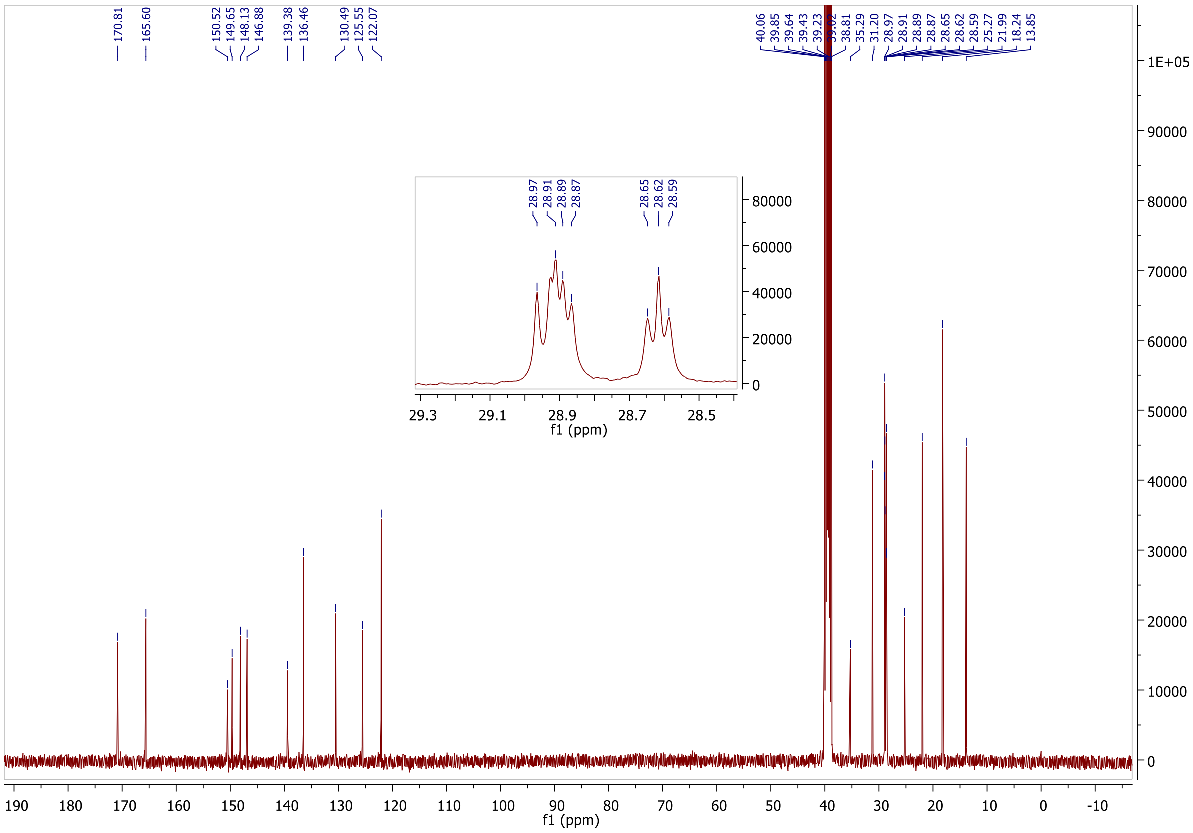

(*E*)-5-[(4-Benzamido-3,5-dimethylphenyl)diazenyl]nicotinamide (**9h**)

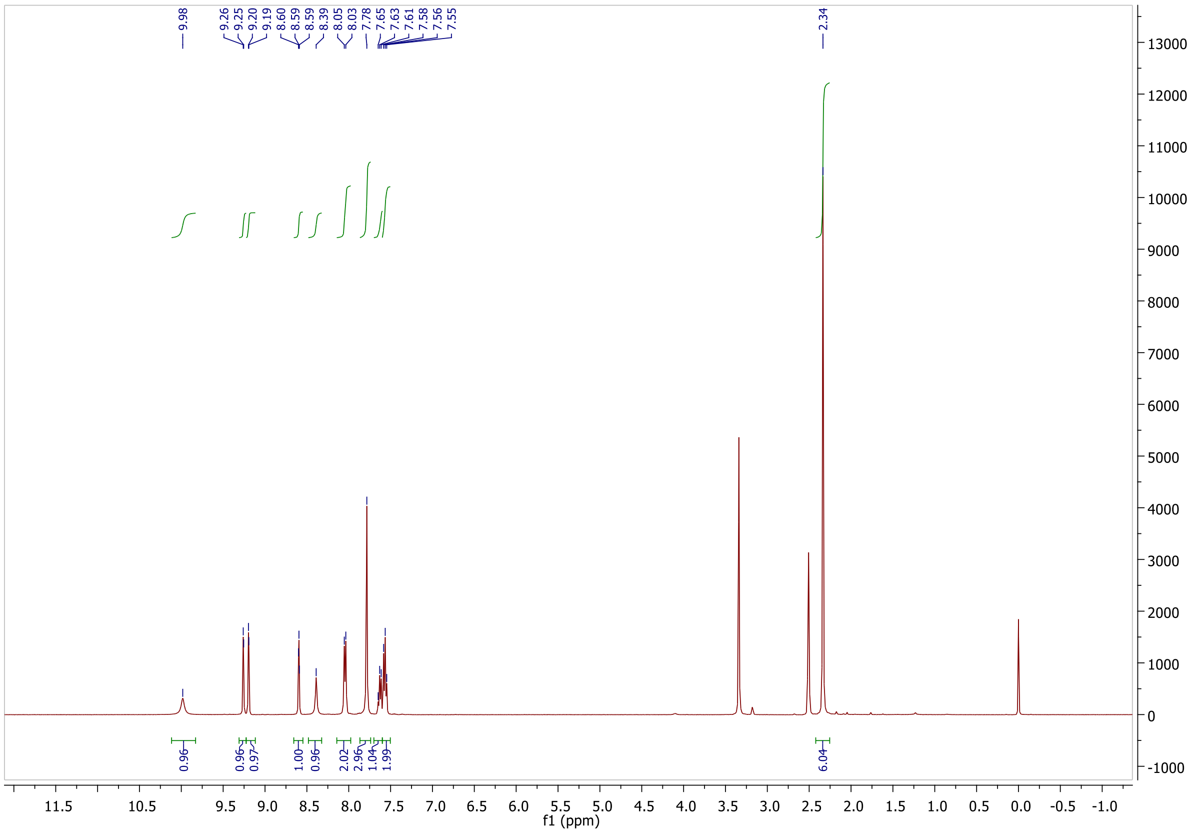

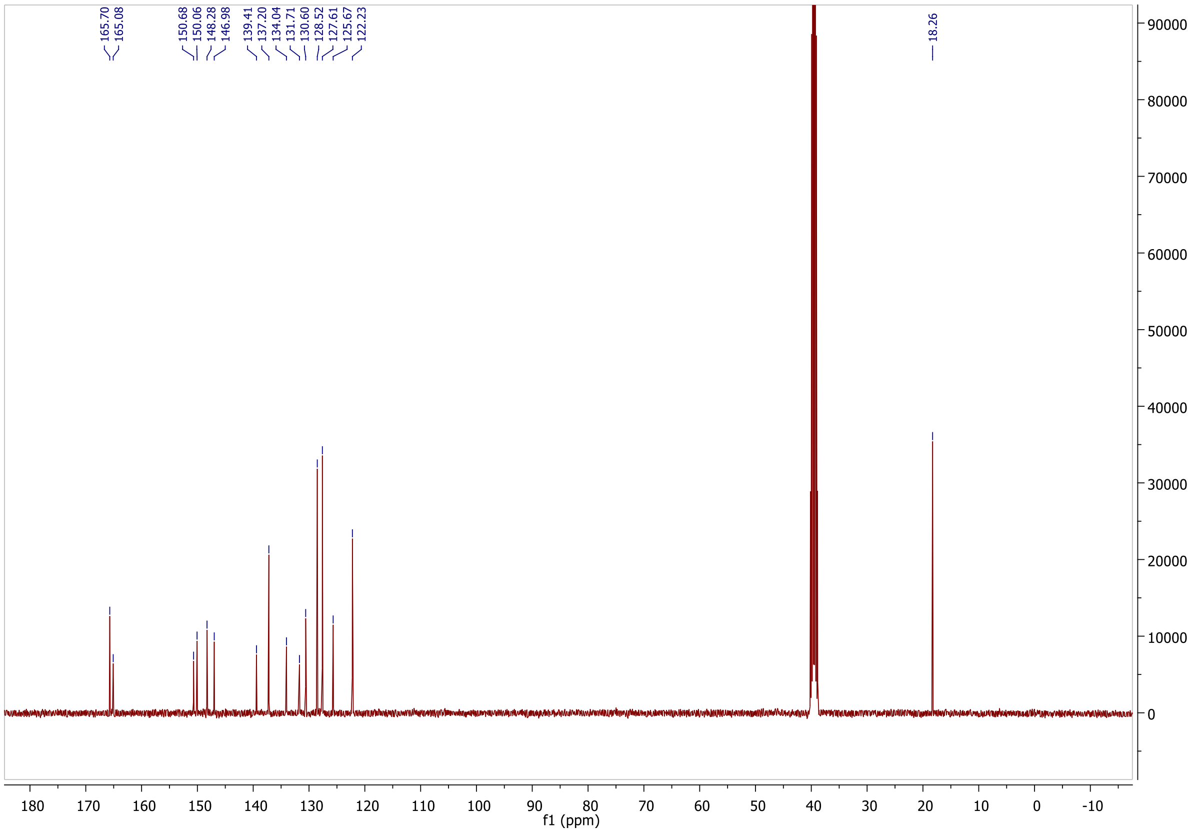

(*E*)-5-{[4-(4-Fluorobenzamido)-3,5-dimethylphenyl]diazenyl}nicotinamide (**9i**)

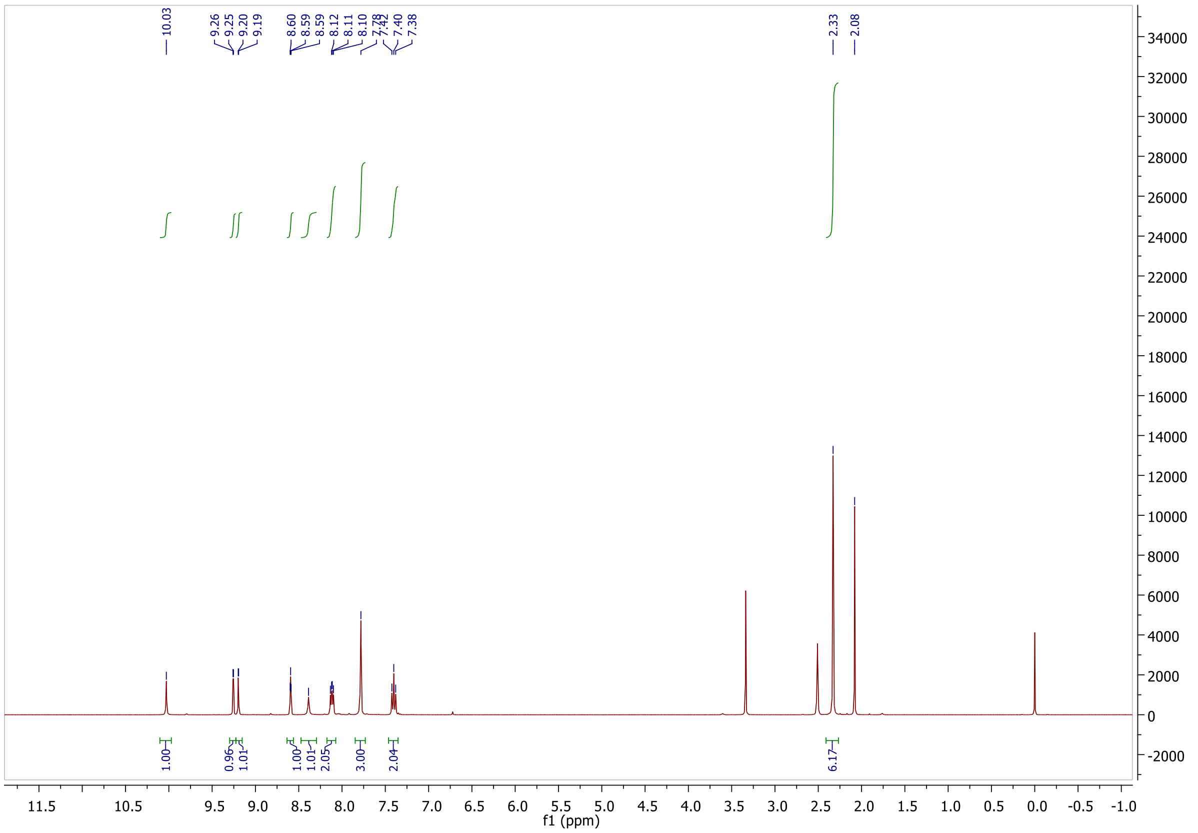

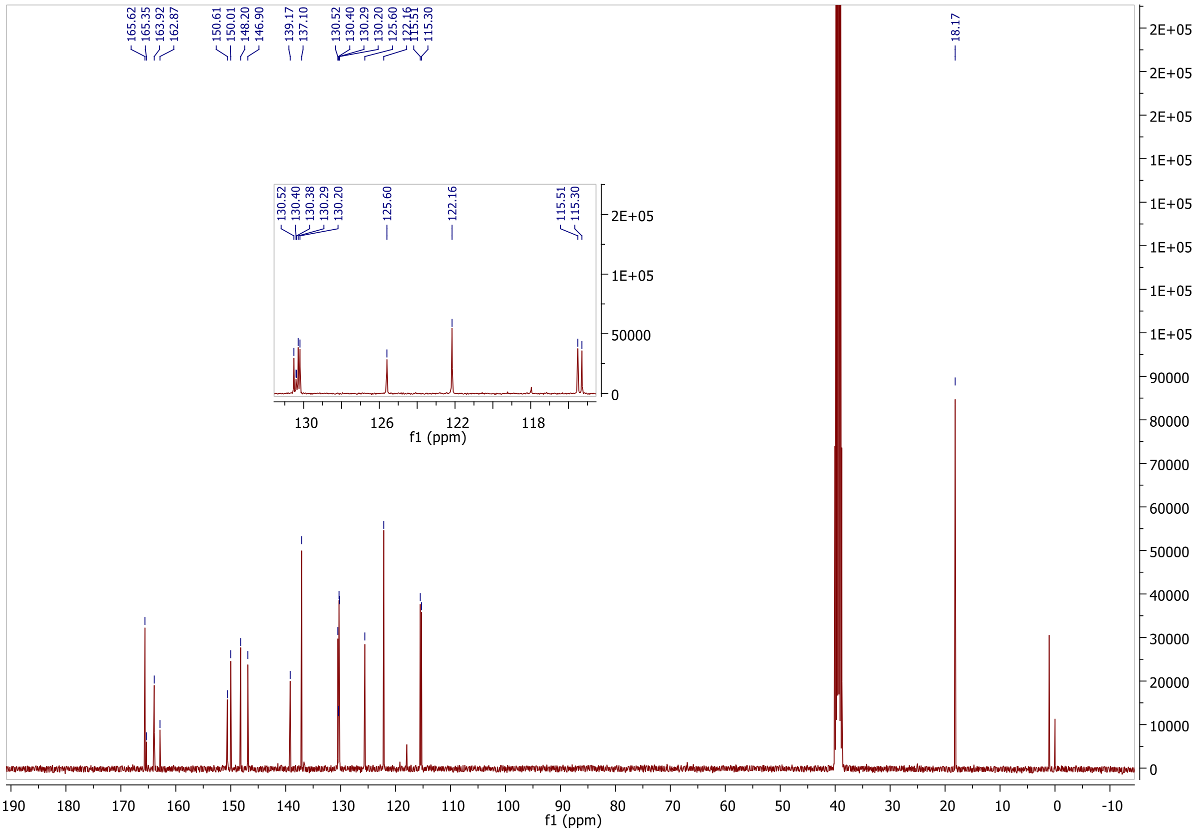

Additional (minor) signals derive from the (*Z*)-photoisomer formed by daylight.

(*E*)-5-({3,5-Dimethyl-4-[4-(thiophen-2-yl)benzamido]phenyl}diazenyl)nicotinamide (**9j**)

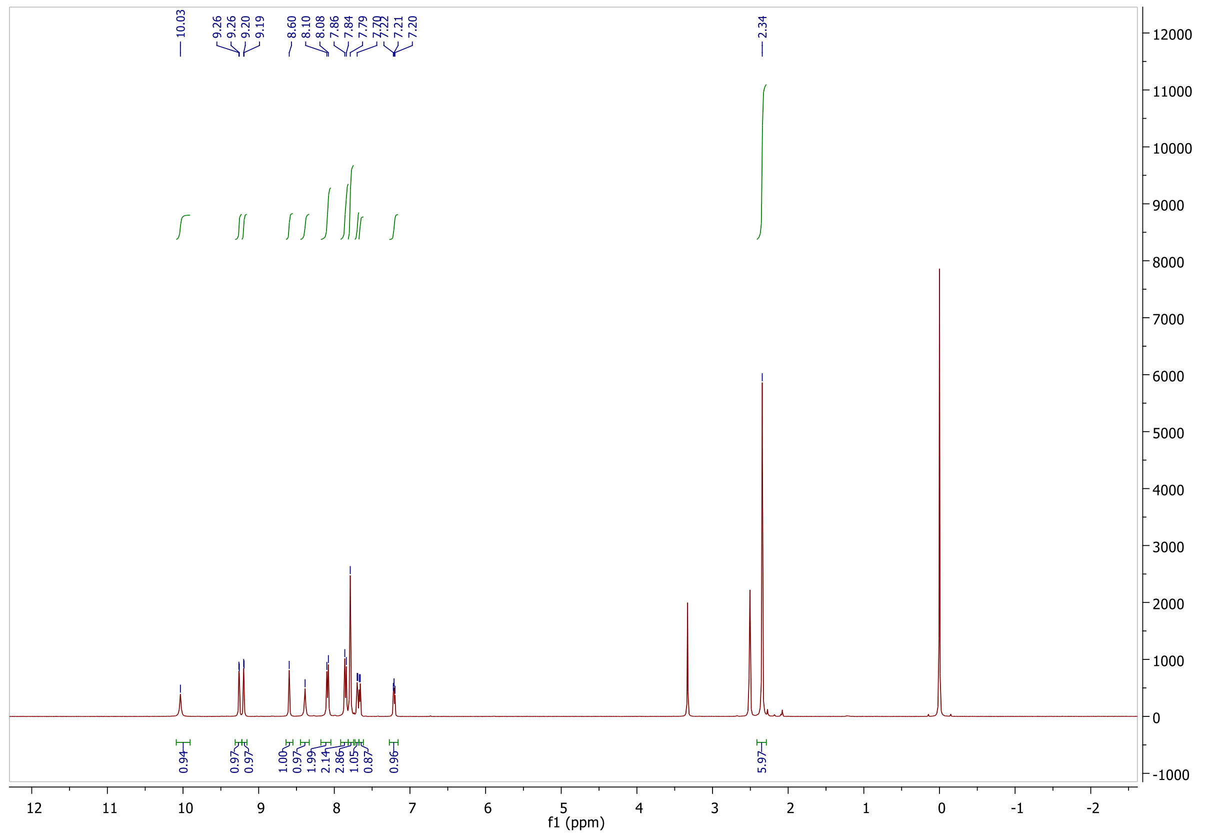

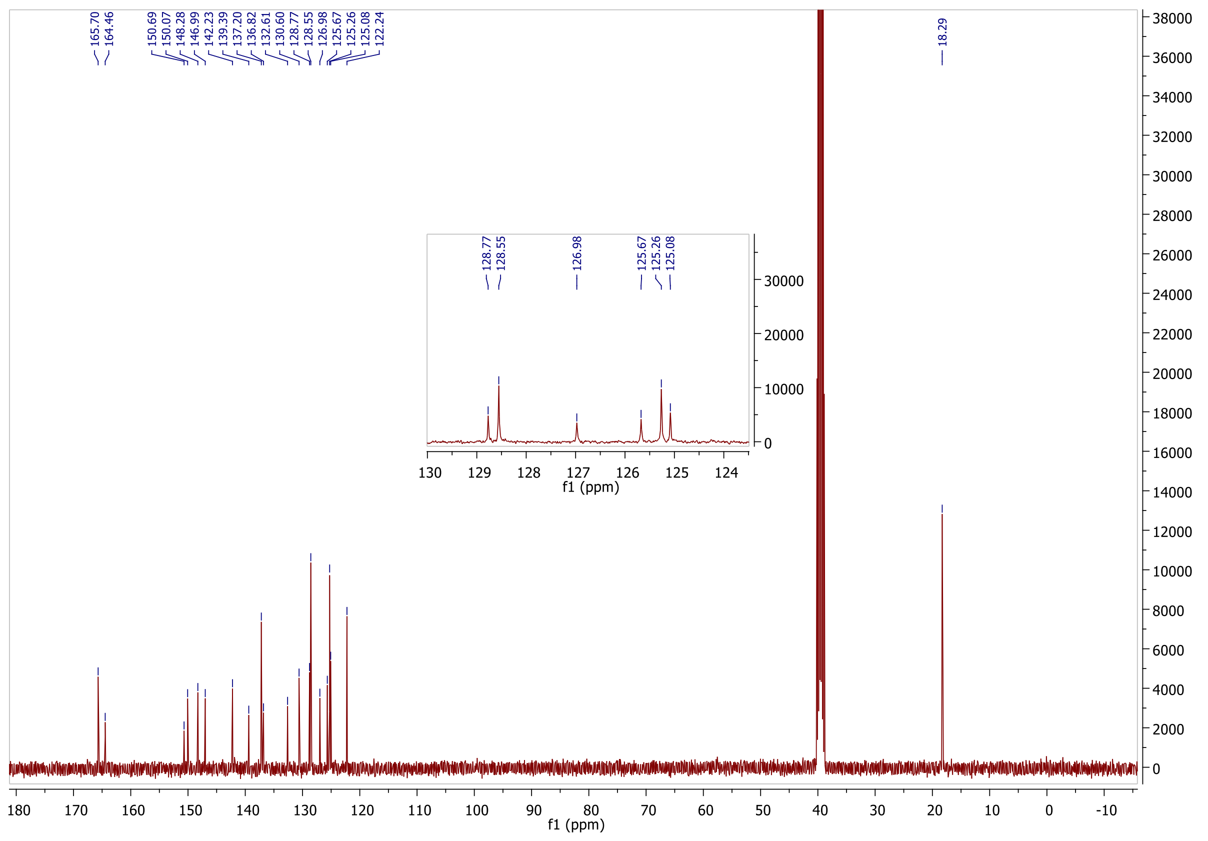

(*E*)-5-{[4-(Adamantane-1-carboxamido)-3,5-dimethylphenyl]diazenyl}nicotinamide (**9k**)

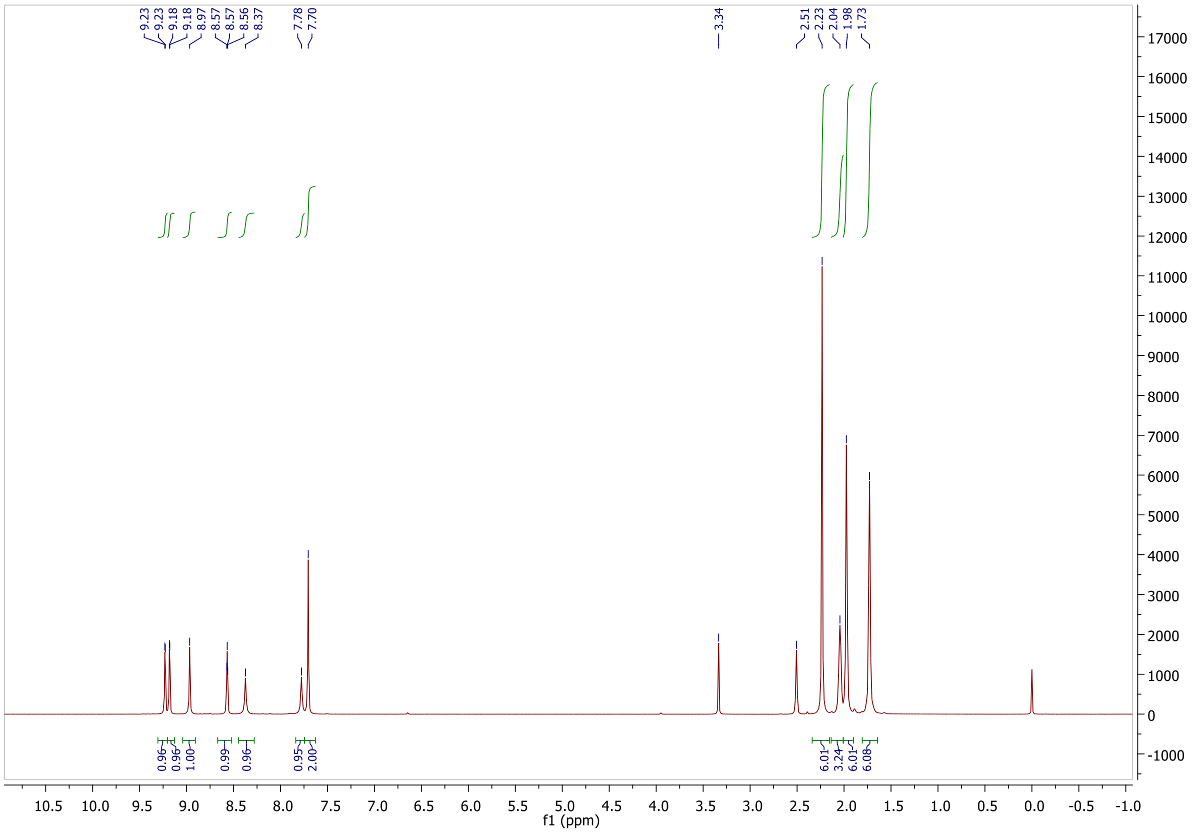

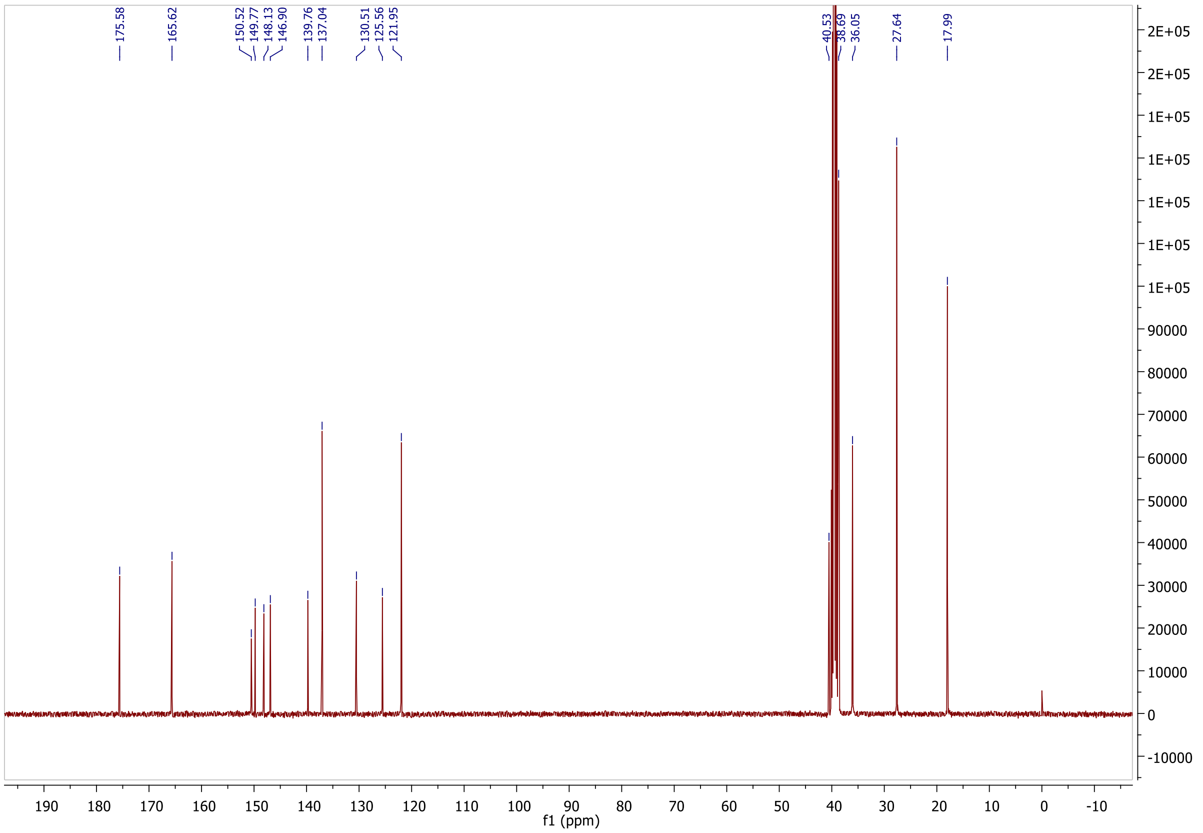

(*E*)-5-[(4-Amino-3,5-dimethylphenyl)diazenyl]-*N*-methylnicotinamide (**12a**)

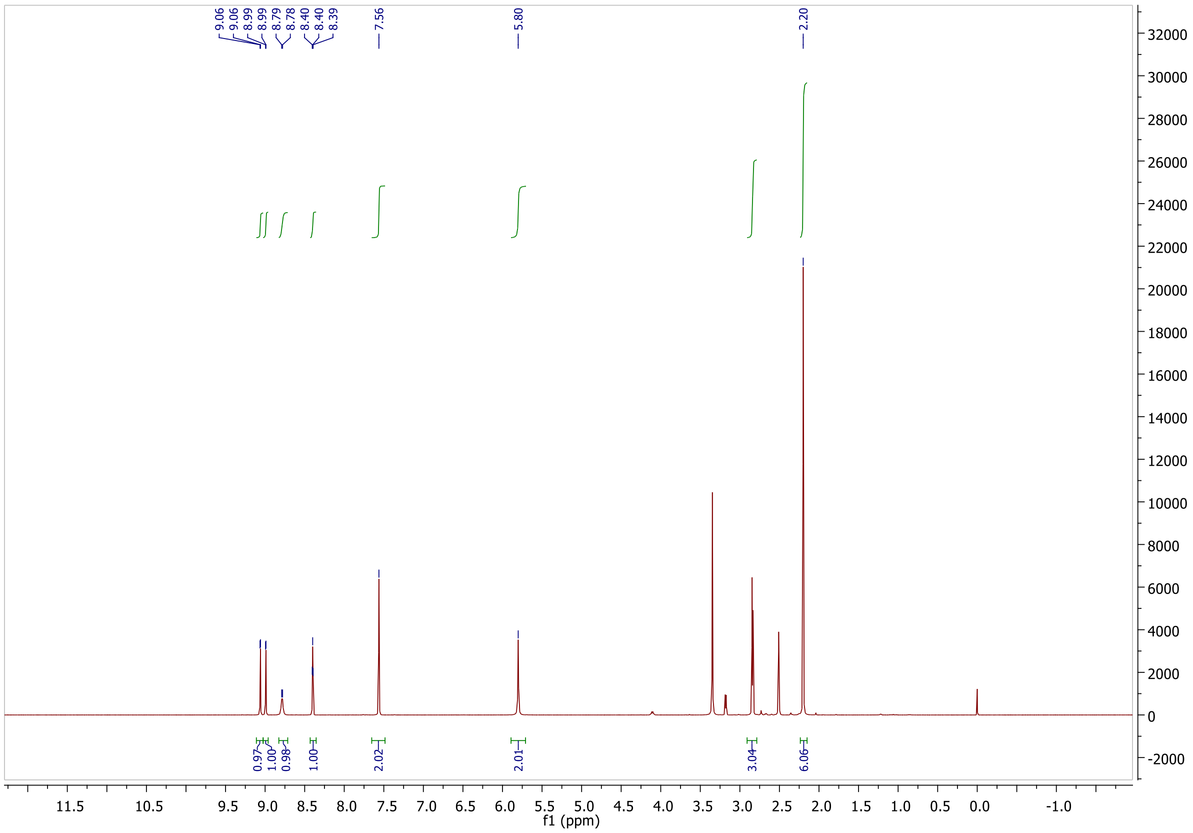

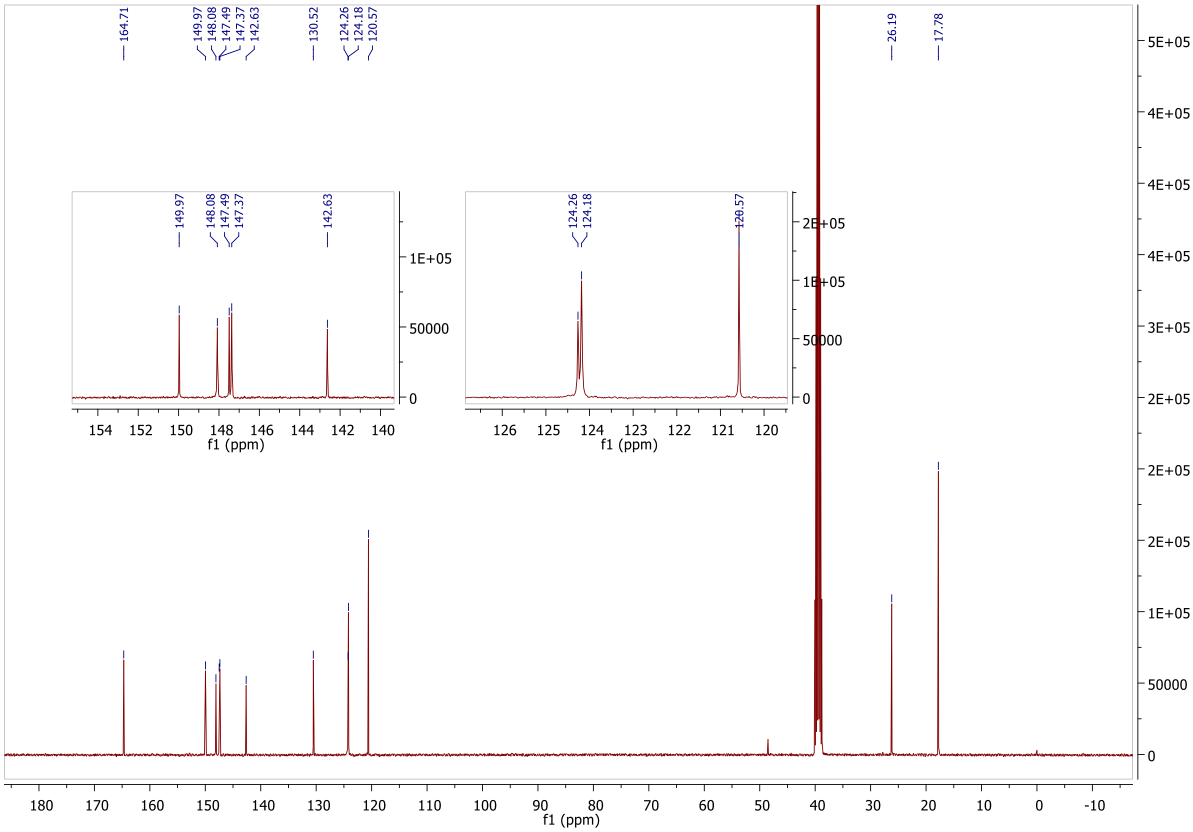

(*E*)-5-[(4-Amino-3,5-dimethylphenyl)diazenyl]-*N*-ethylnicotinamide (**12b**)

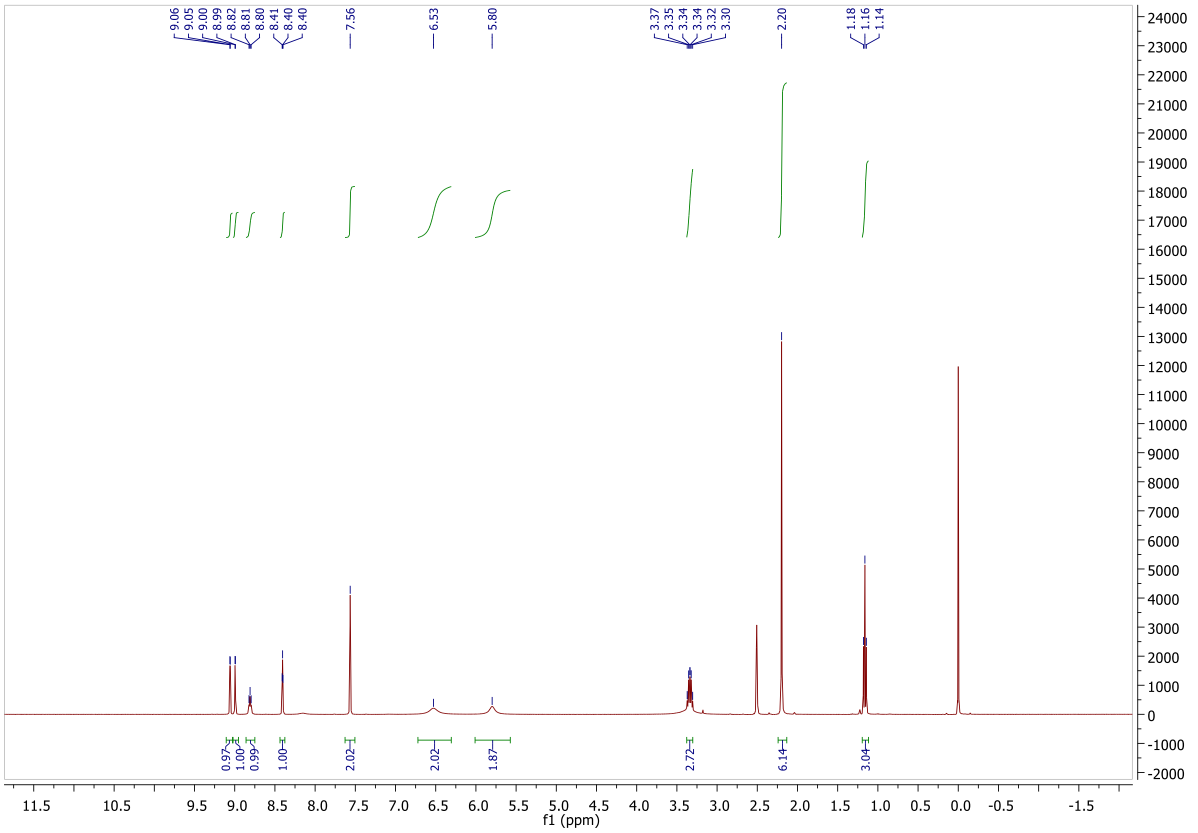

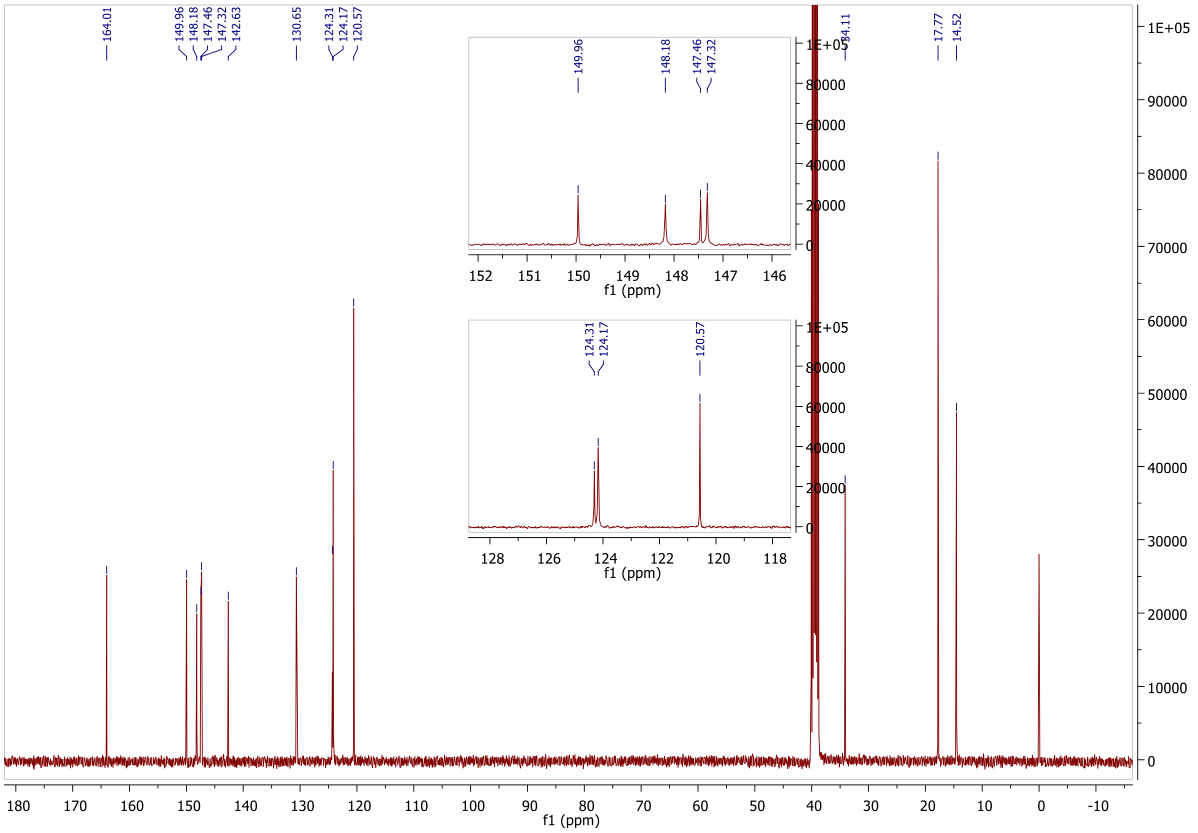

(*E*)-5-[(4-Amino-3,5-dimethylphenyl)diazenyl]-*N*-butylnicotinamide (**12c**)

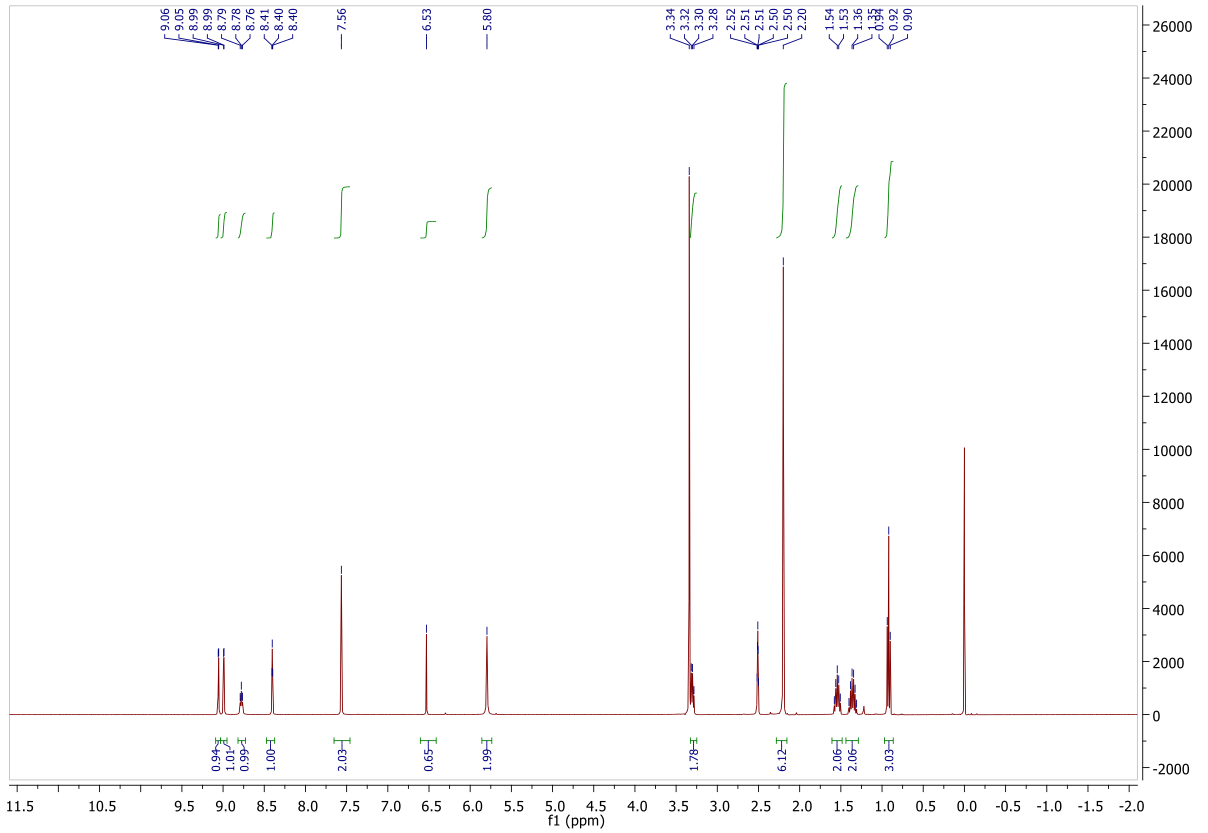

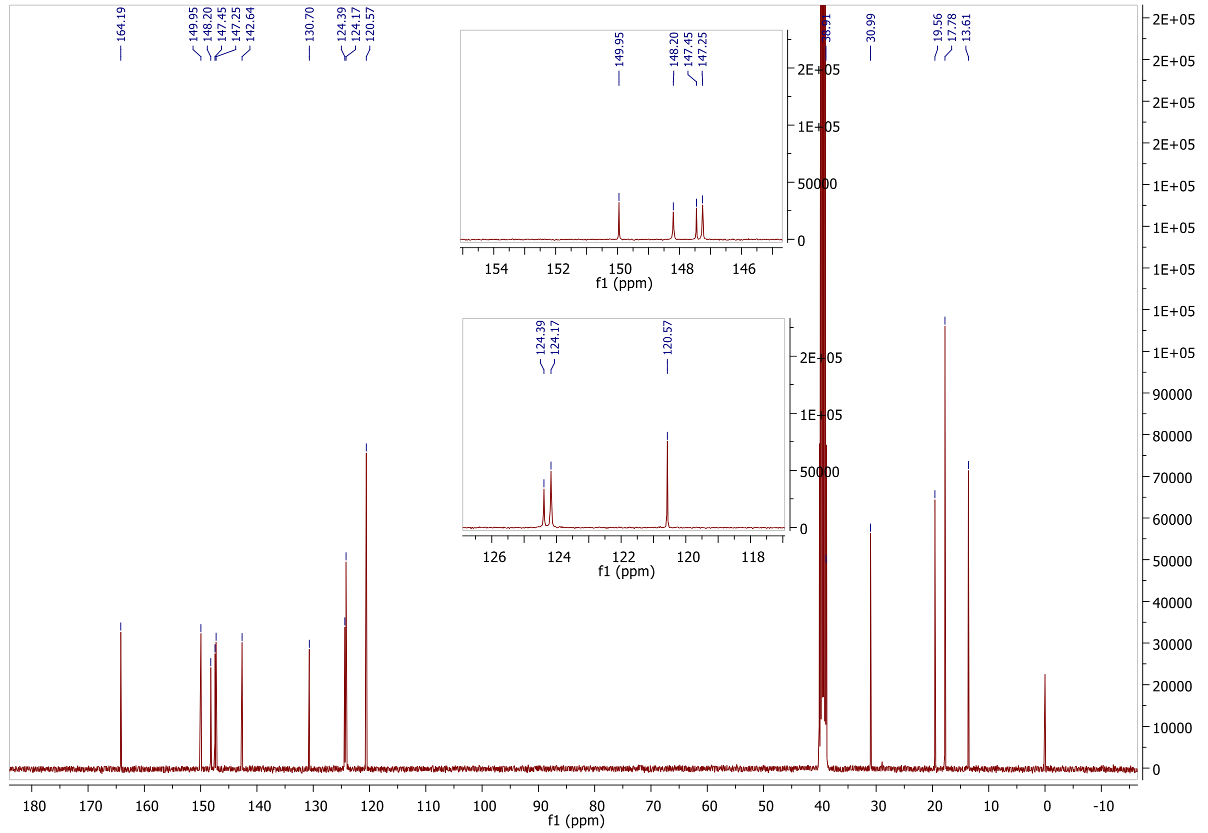

(*E*)-5-[(4-Amino-3,5-dimethylphenyl)diazenyl]-*N*-(2-hydroxyethyl)nicotinamide (**12d**)

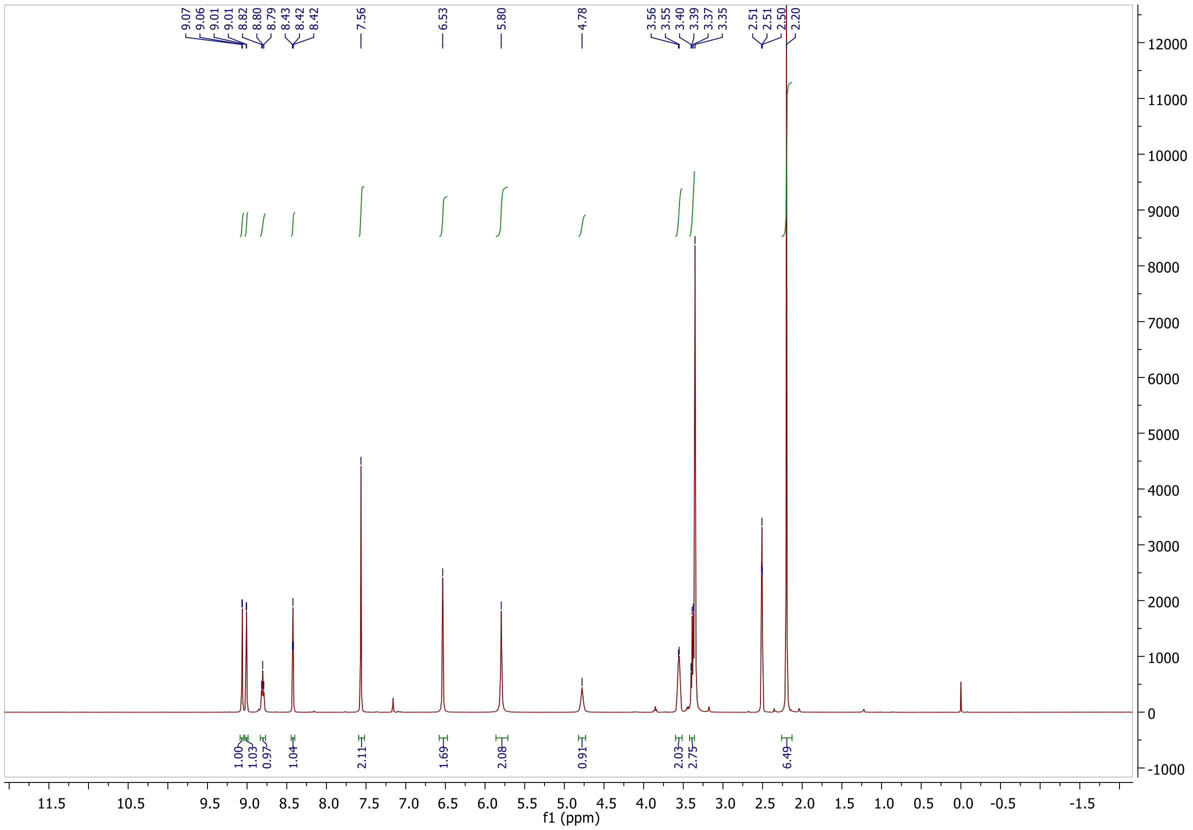

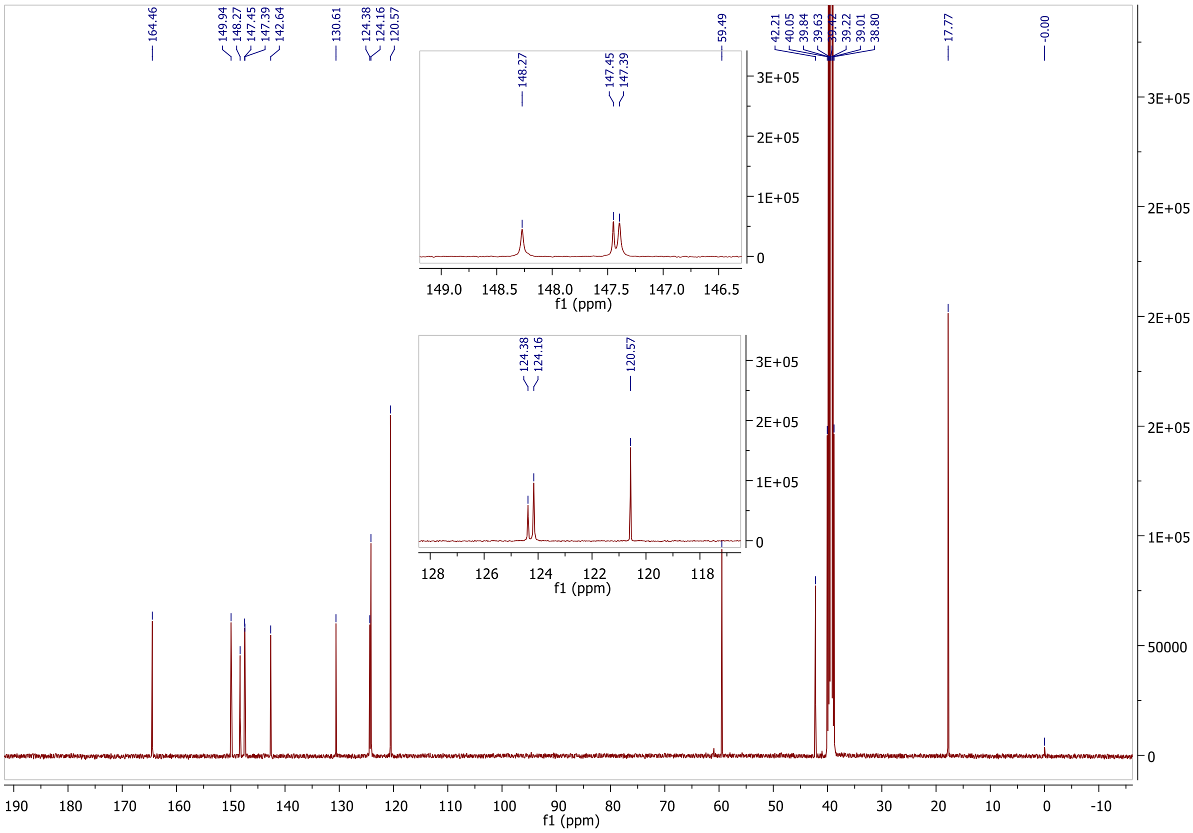

Methyl (*E*)-5-{[4-(tert-butoxycarbonyl)phenyl]diazenyl}nicotinate (**14a**)

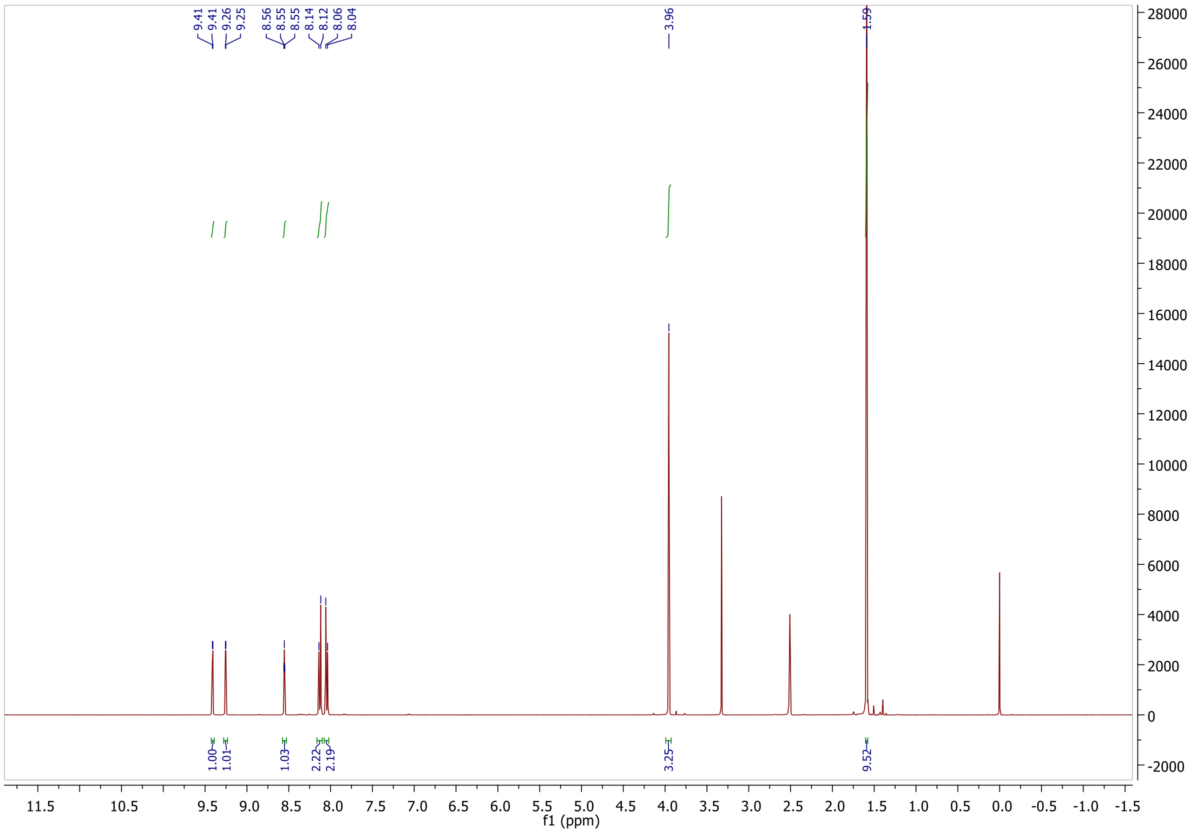

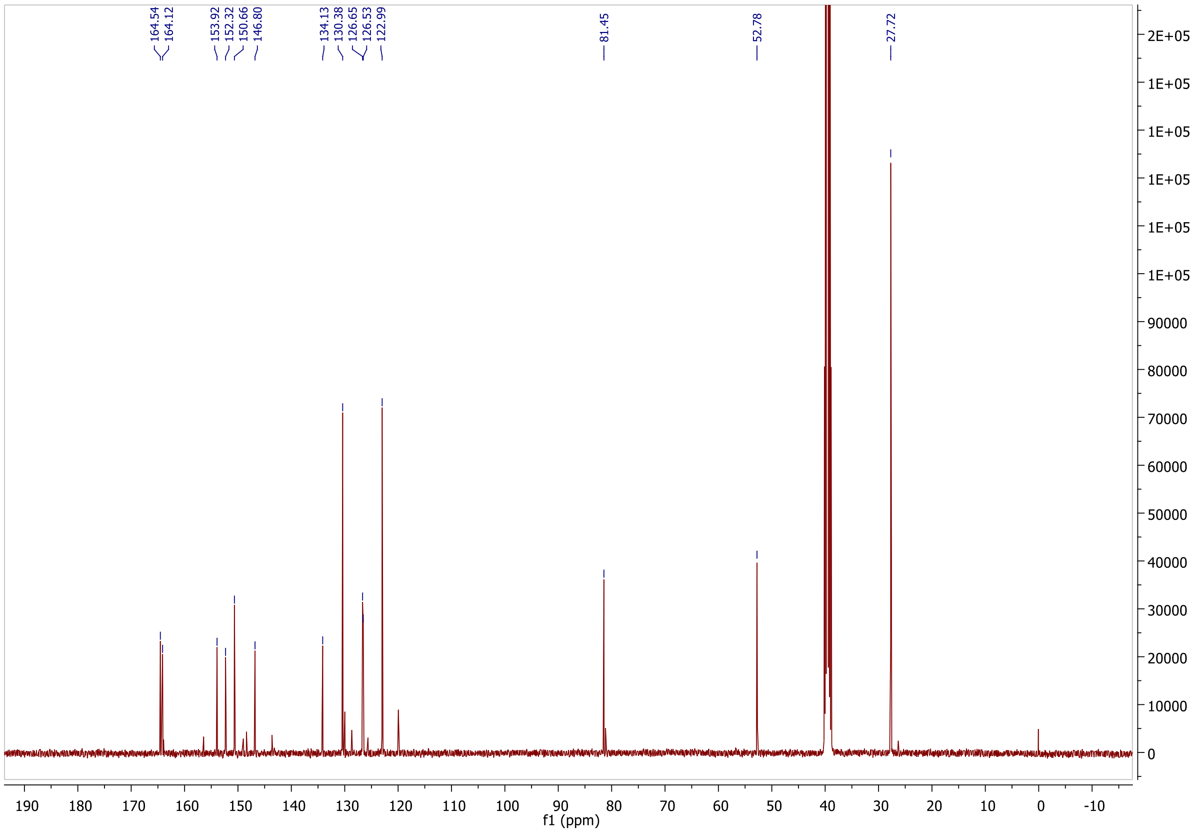

Additional (minor) signals derive from the (*Z*)-photoisomer formed by daylight.

Methyl (*E*)-5-({4-[2-(tert-butoxy)-2-oxoethyl]phenyl}diazenyl)nicotinate (**14b**)

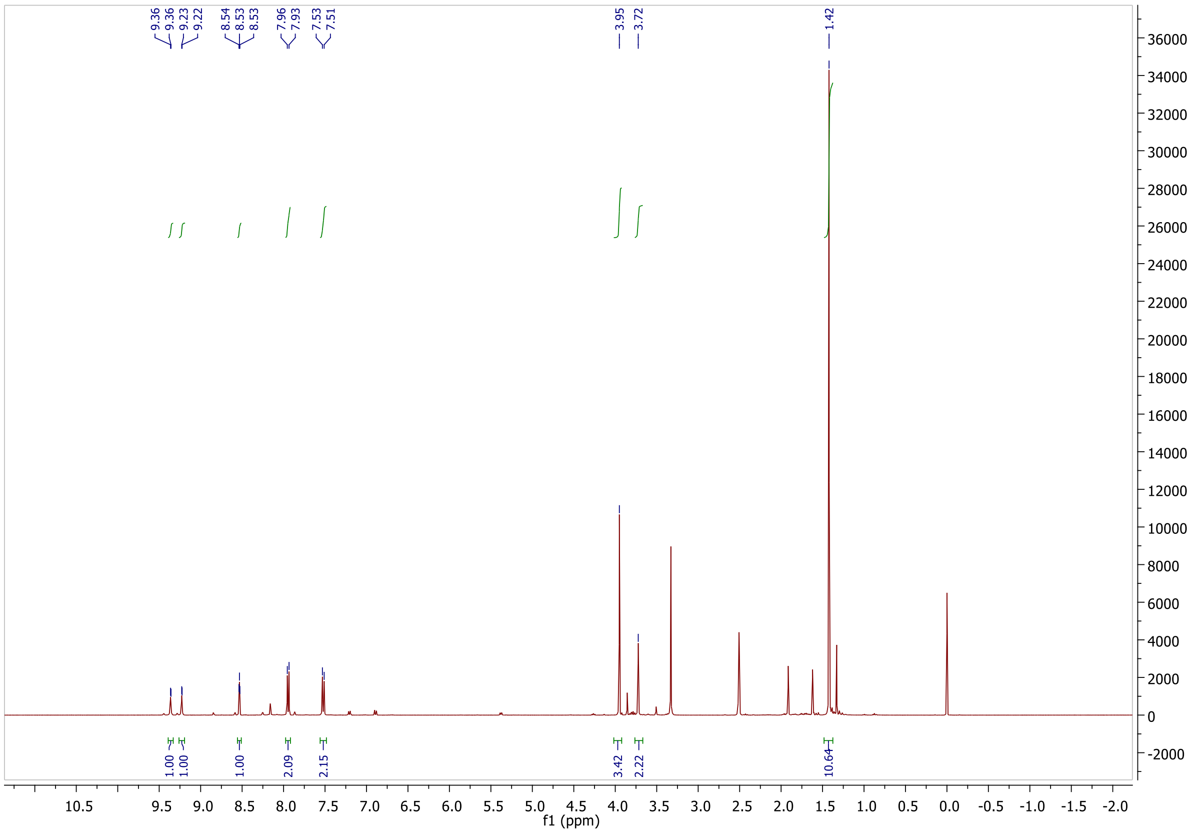

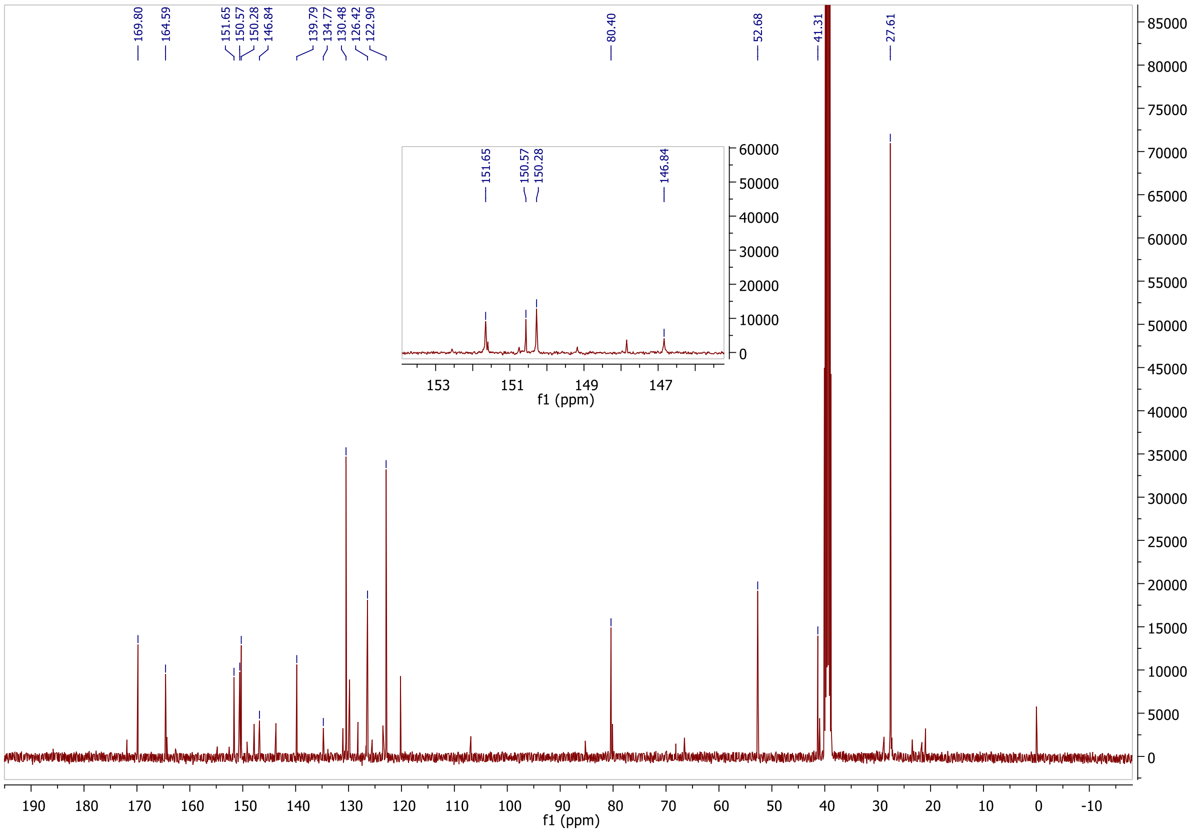

Additional (minor) signals derive from the (*Z*)-photoisomer formed by daylight.

Methyl (*E*)-5-[(4-{[(tert-butoxycarbonyl)amino]methyl}phenyl)diazenyl]-nicotinate (**14c**)

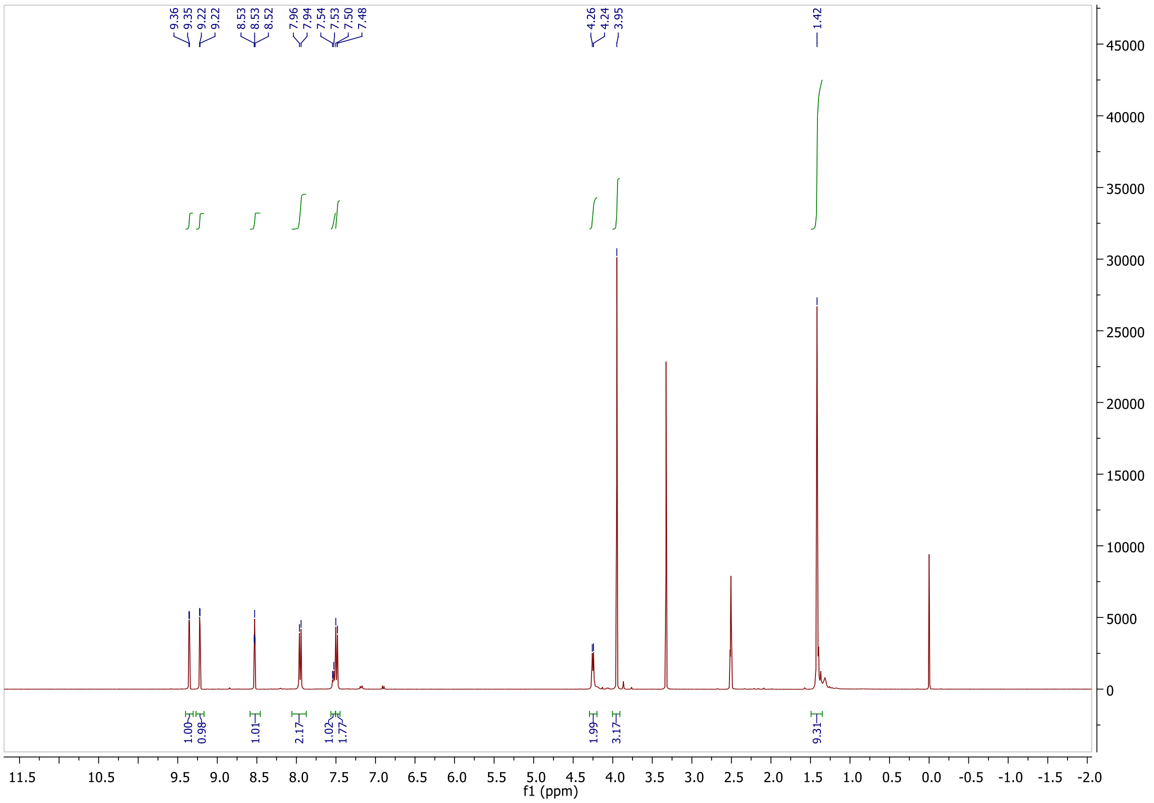

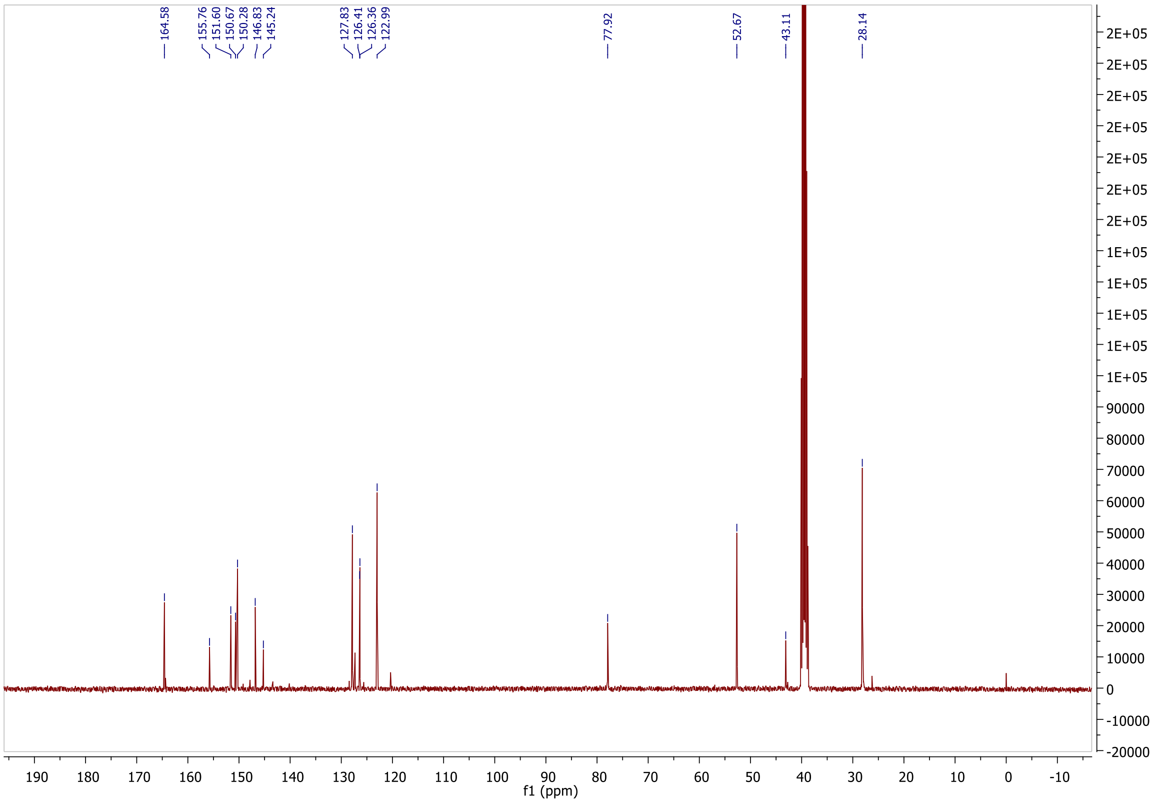

Additional (minor) signals derive from the (*Z*)-photoisomer formed by daylight.

Methyl (*E*)-5-[(4-bromophenyl)diazenyl]nicotinate (**14d**)

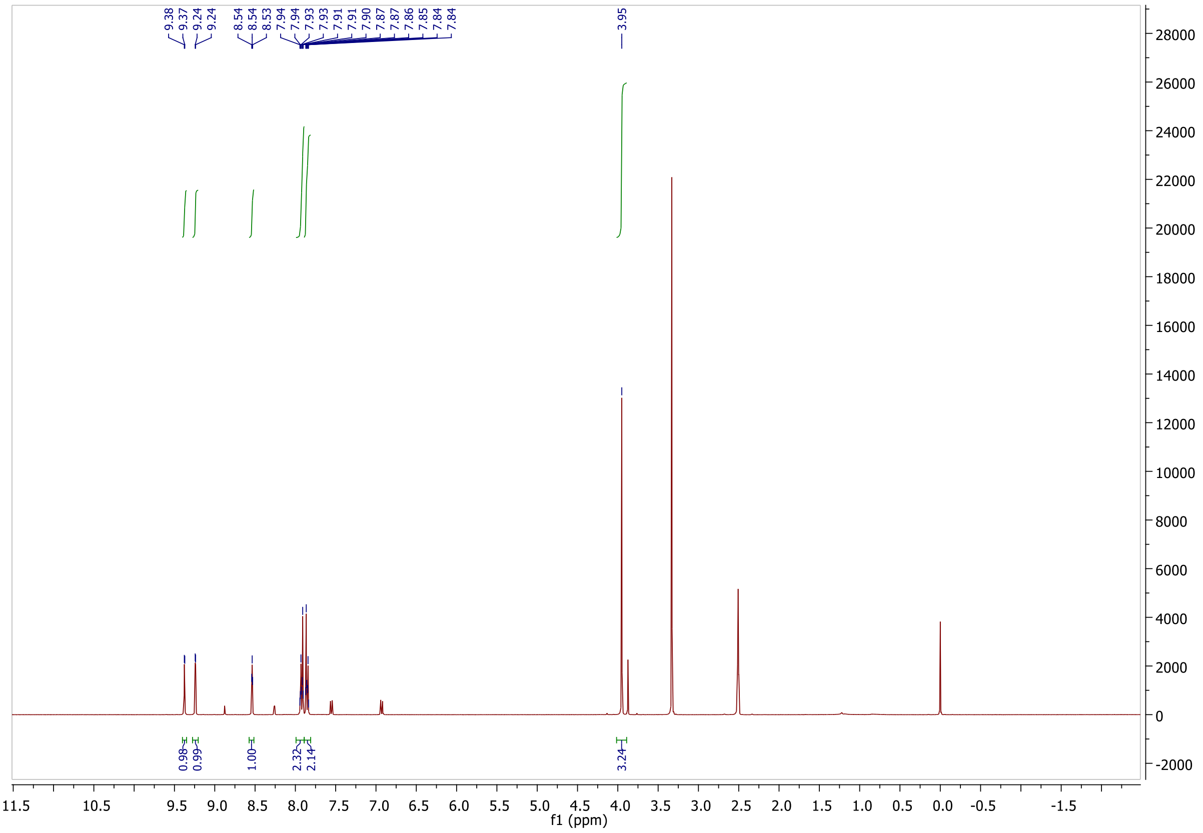

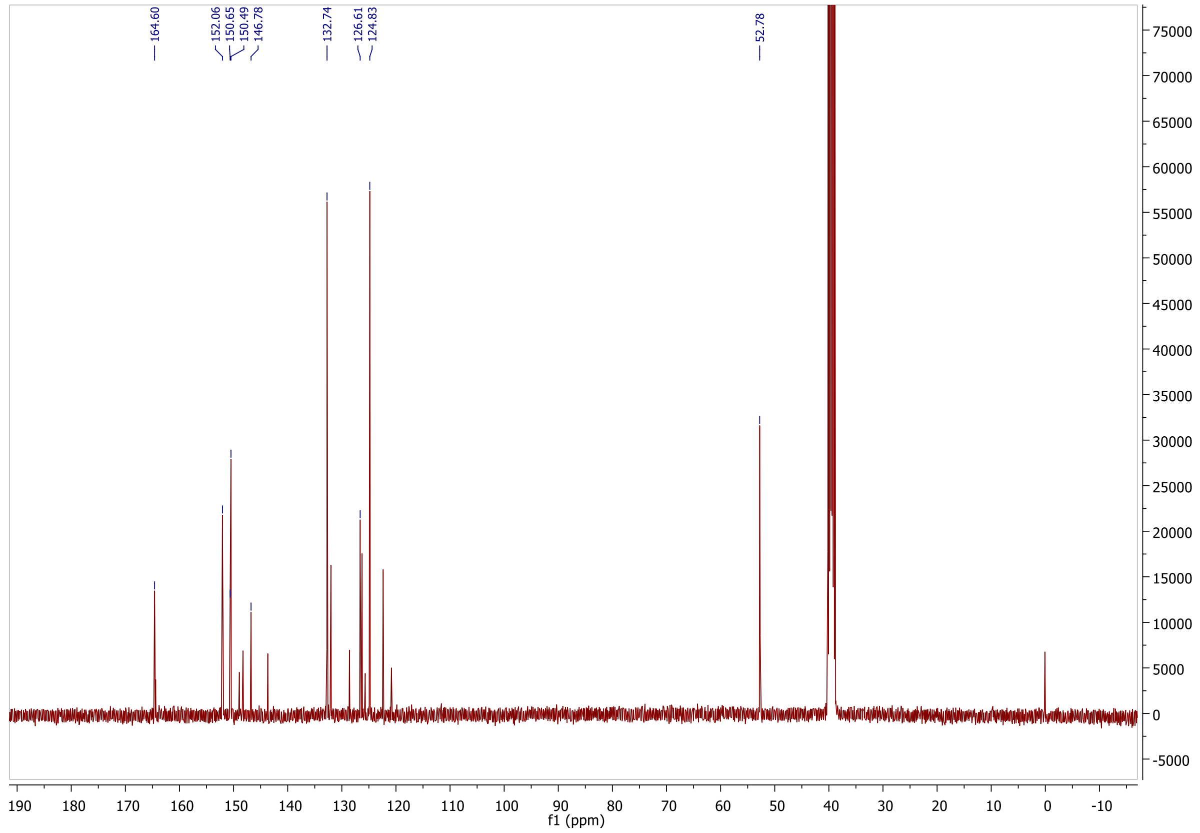

Additional (minor) signals derive from the (*Z*)-photoisomer formed by daylight.

Methyl (*E*)-5-{[4-(butylcarbamoyl)phenyl]diazenyl}nicotinate (**14e**)

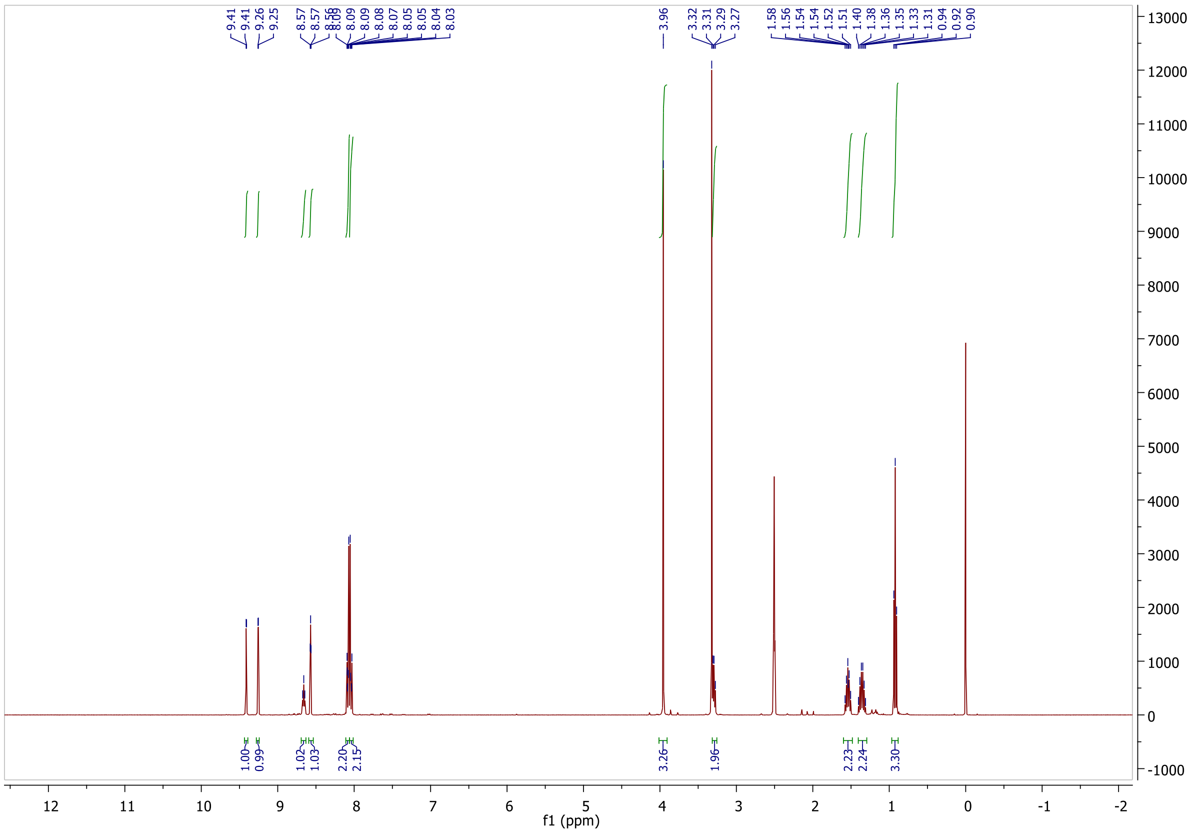

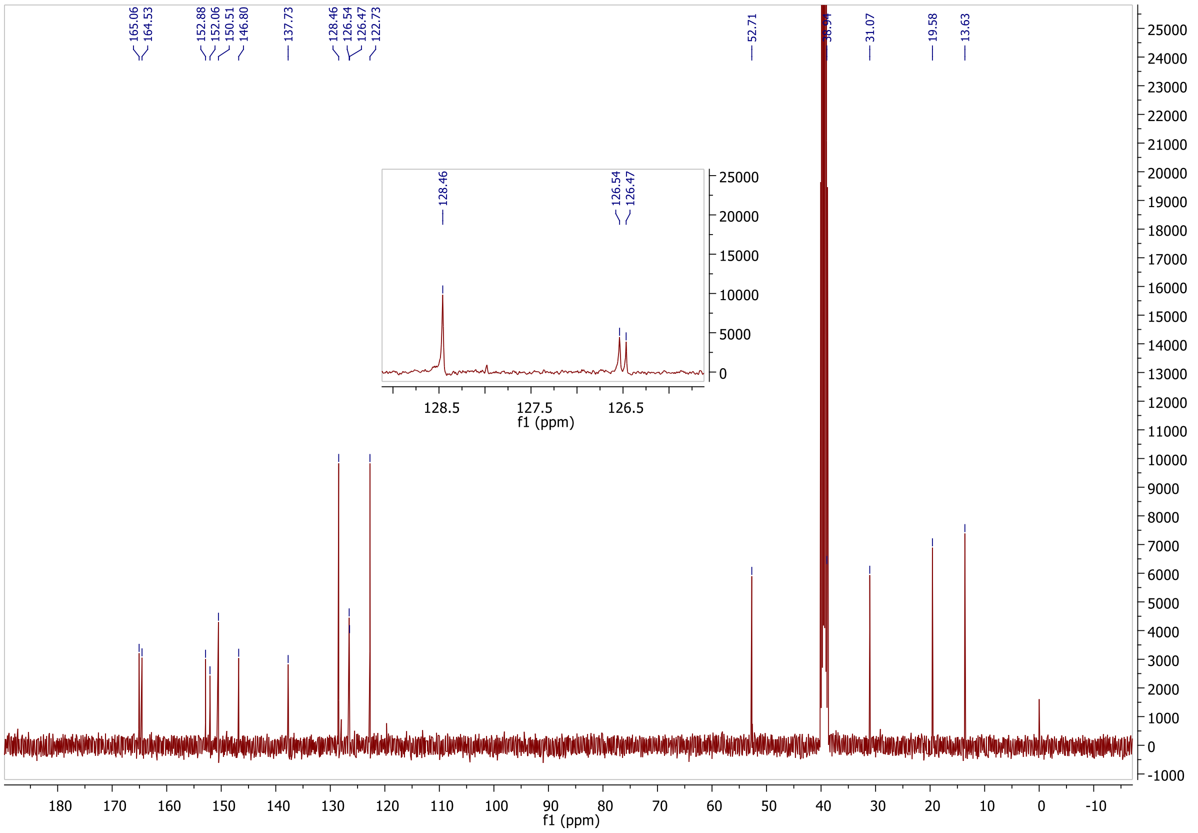

Additional (minor) signals derive from the (*Z*)-photoisomer formed by daylight.

Methyl (*E*)-5-{[4-(decanamidomethyl)phenyl]diazenyl}nicotinate (**14f**)

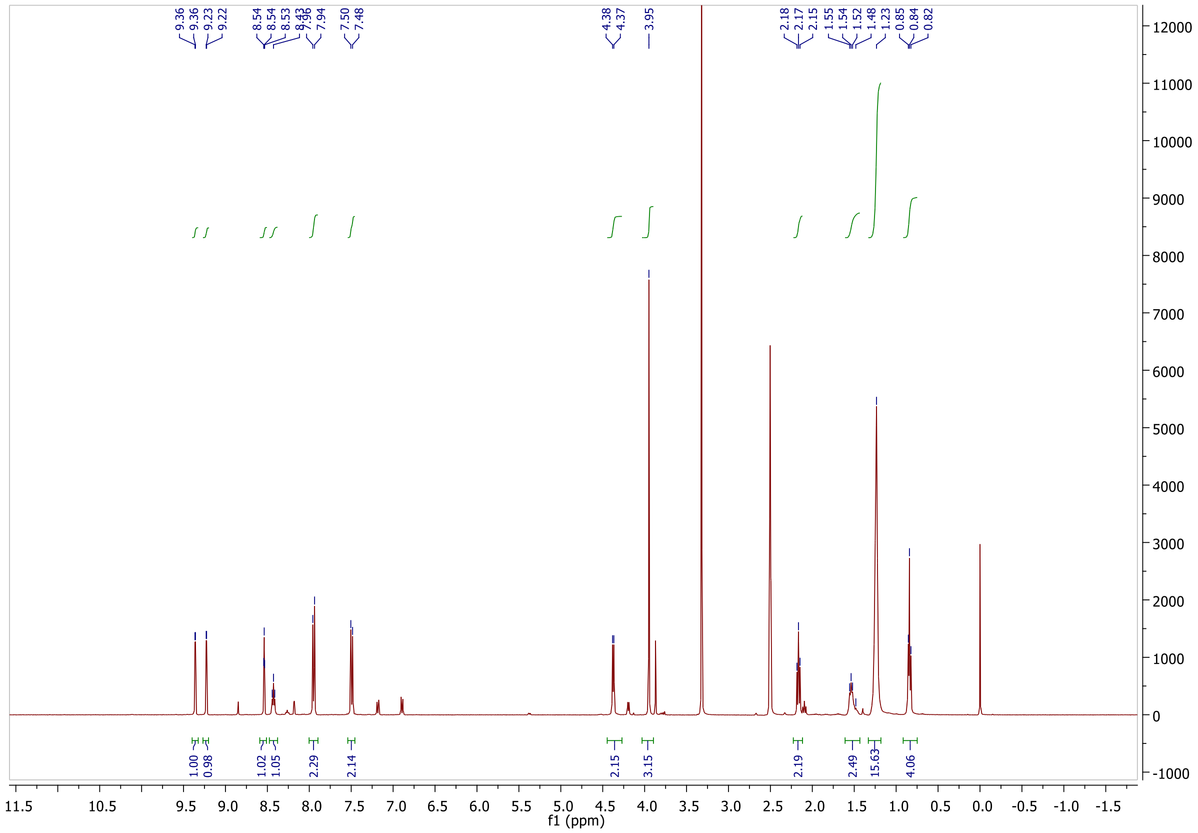

Methyl (*E*)-5-{[4-(6-hydroxyhex-1-yn-1-yl)phenyl]diazenyl}nicotinate (**14g**)

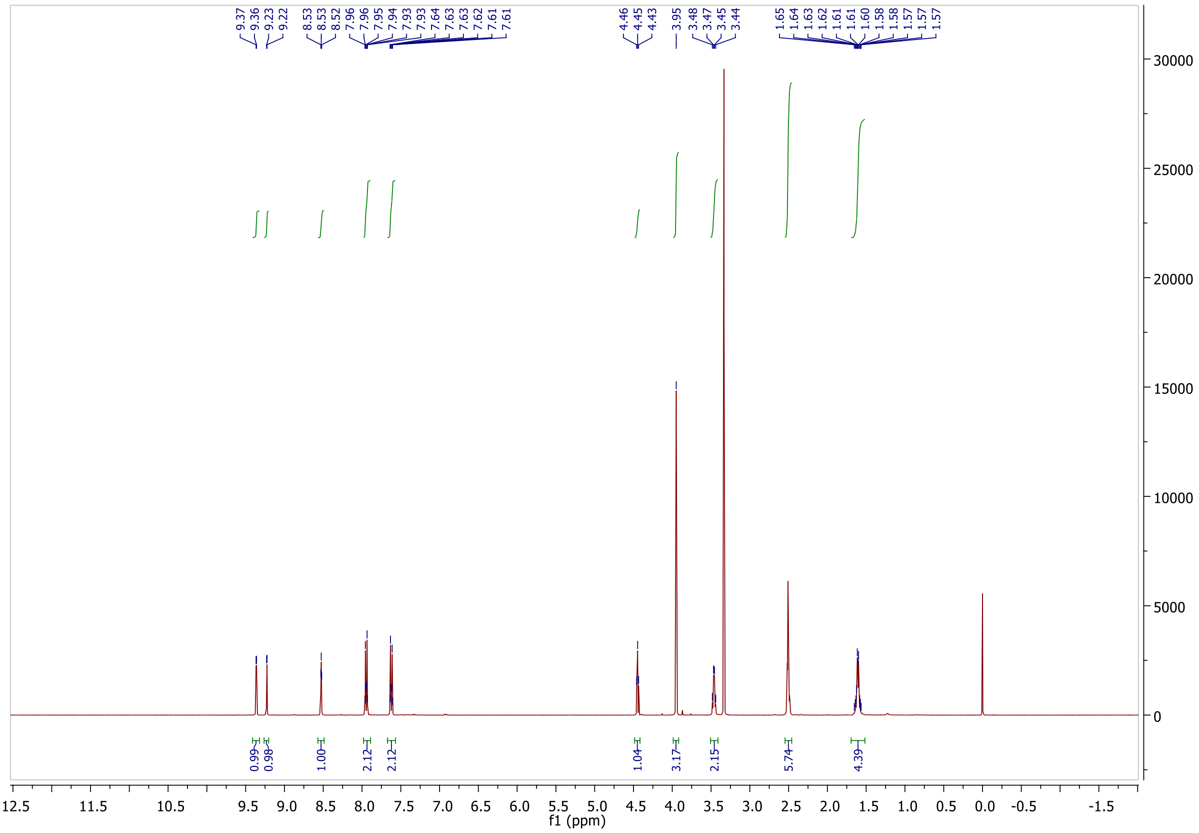

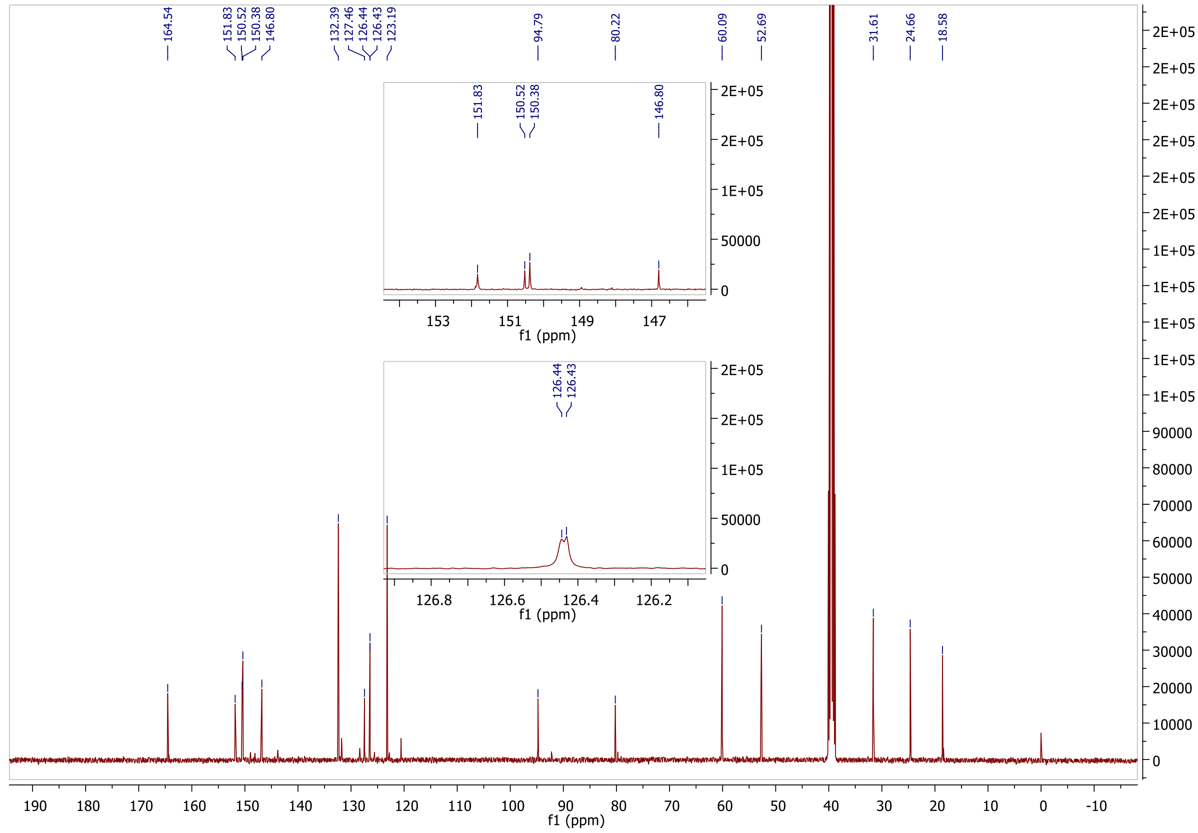

Additional (minor) signals derive from the (*Z*)-photoisomer formed by daylight.

Methyl (E)-5-{[4-(3-hydroxy-3-methylbut-1-yn-1-yl)phenyl]diazenyl}nicotinate (**14h**)

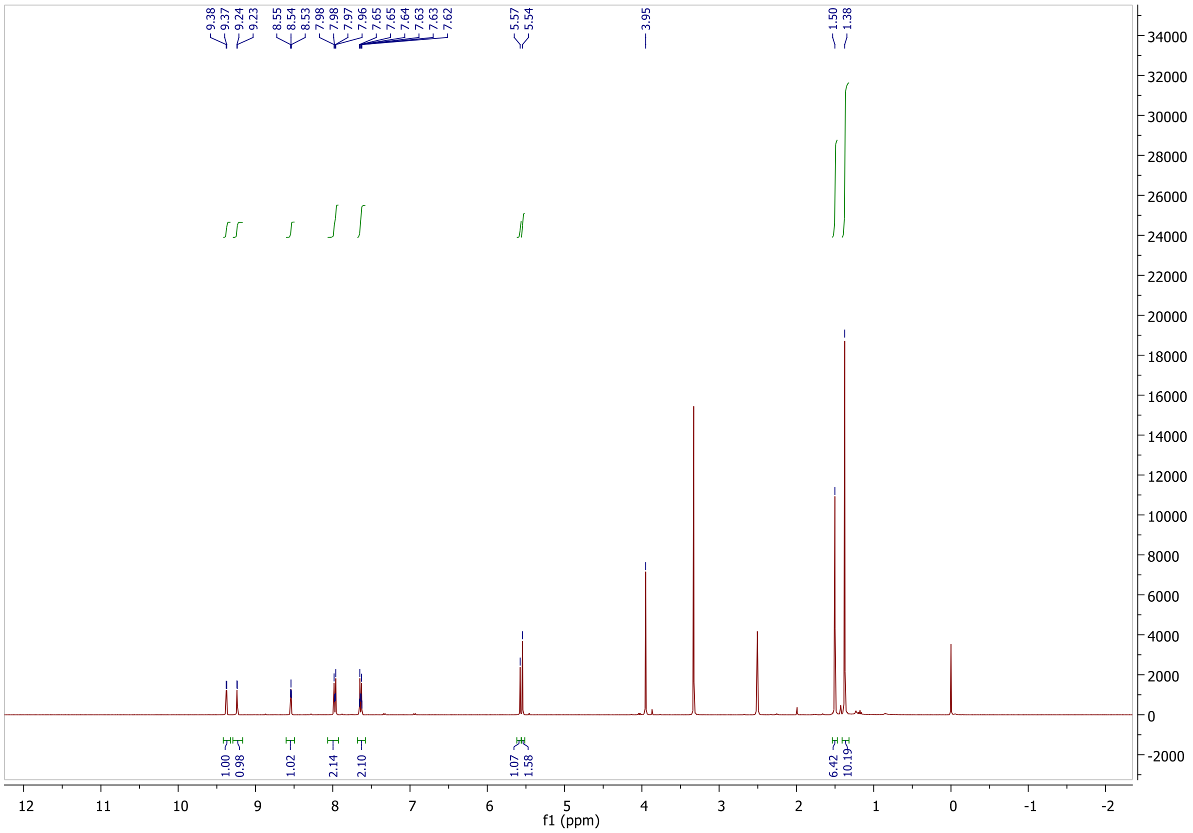

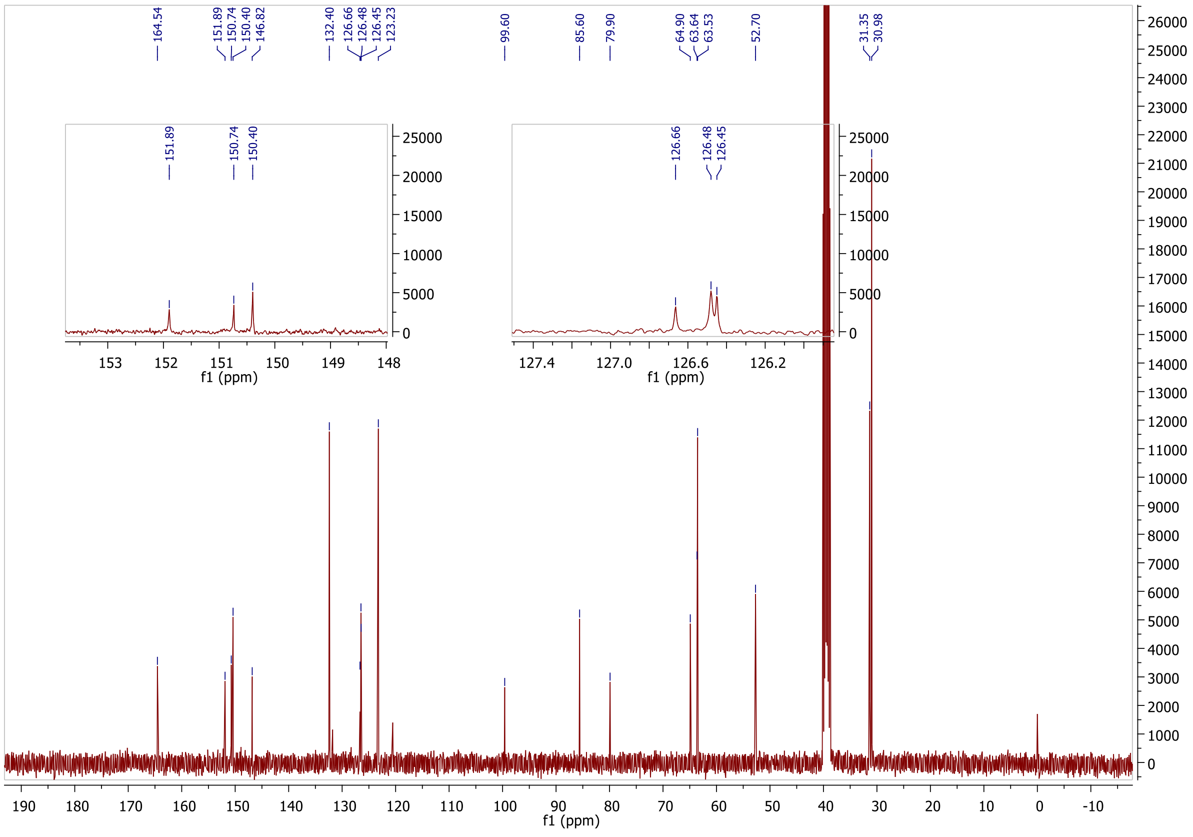

(*E*)-5-{[4-(*tert*-Butoxycarbonyl)phenyl]diazenyl}nicotinamide (**15a**)

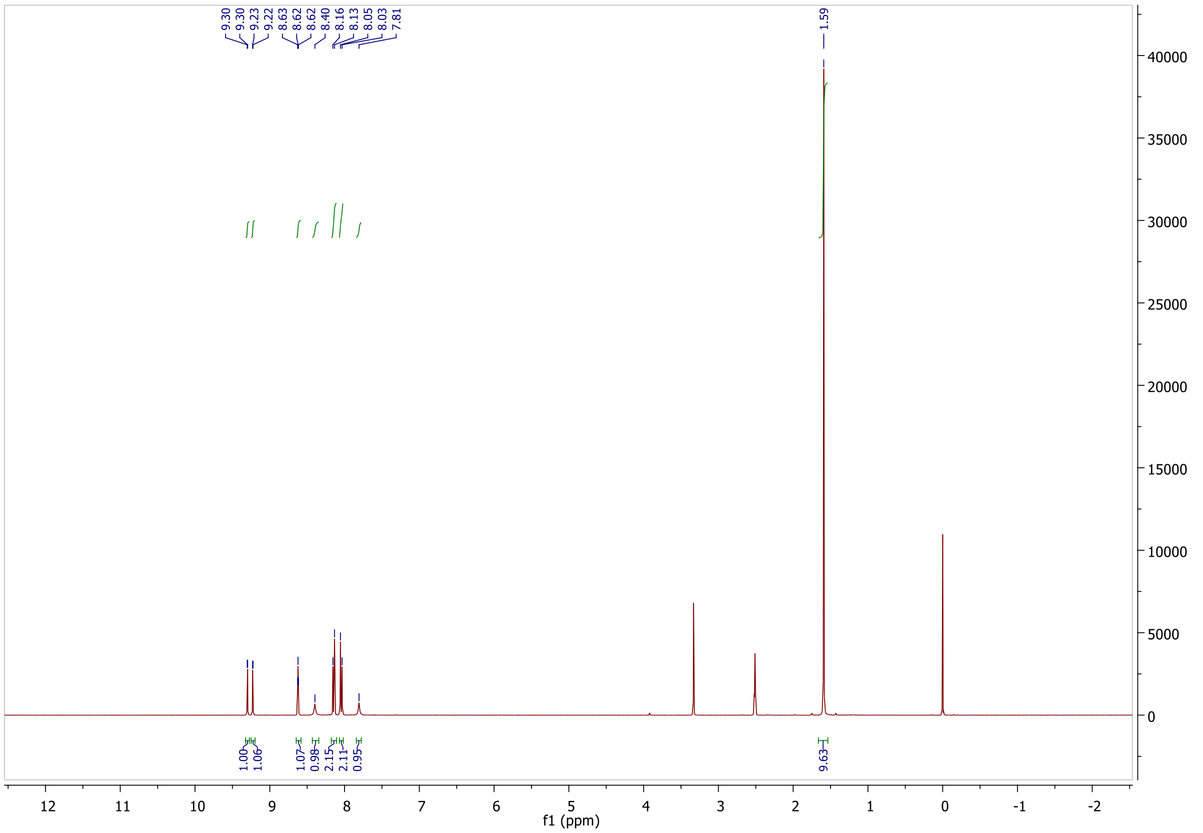

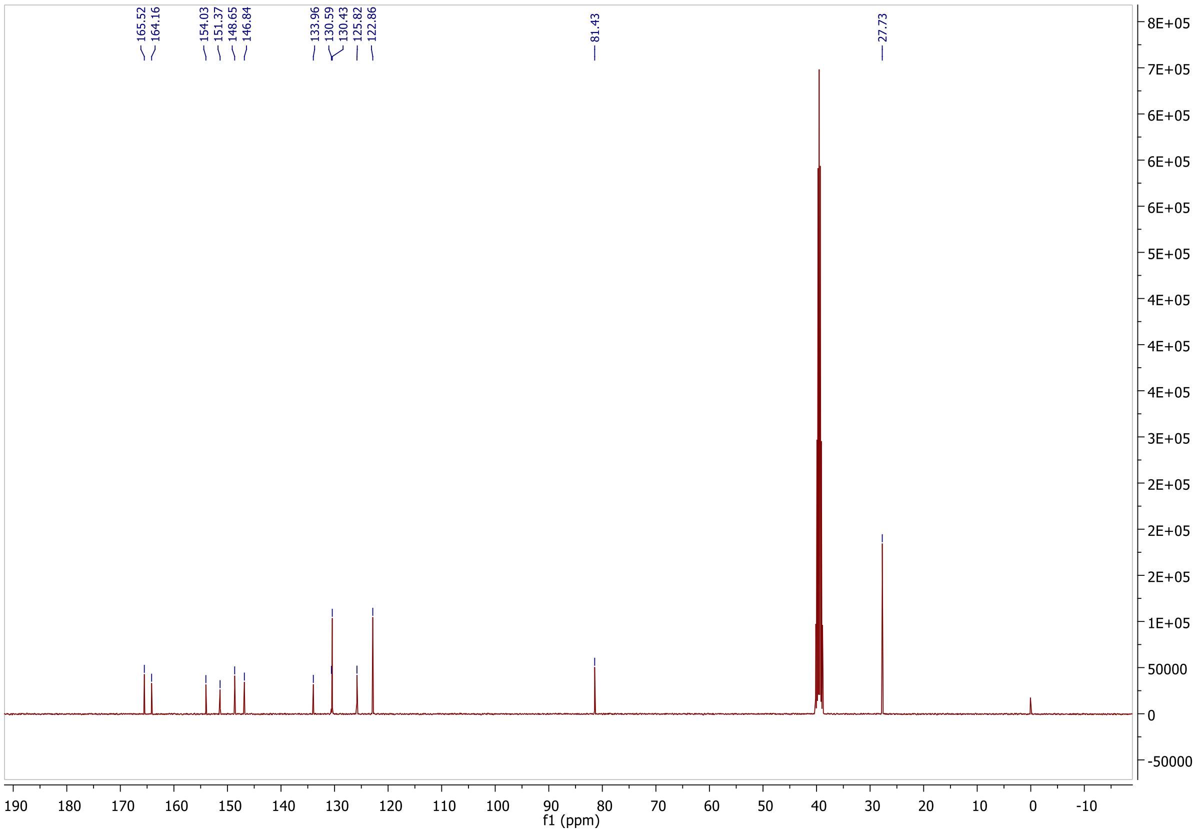

(*E*)-5-({4-[2-(*tert*-Butoxy)-2-oxoethyl]phenyl}diazenyl)nicotinamide (**15b**)

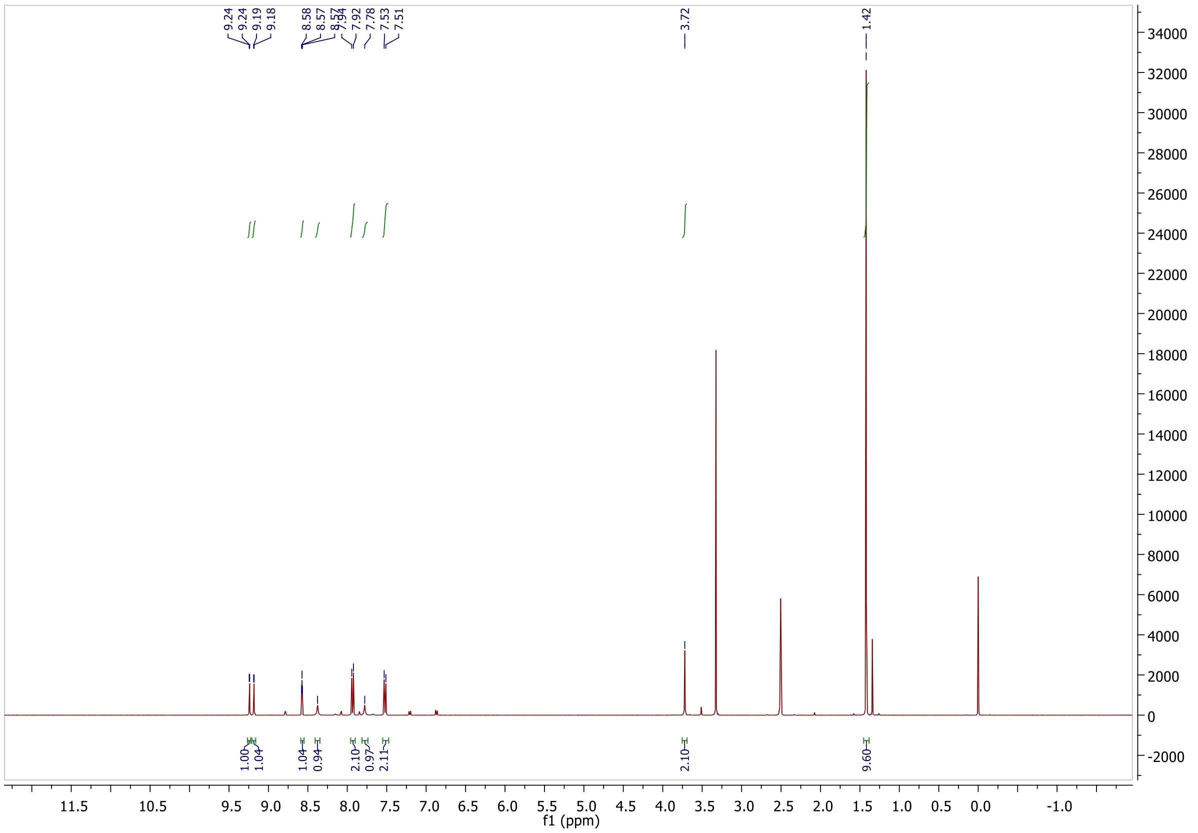

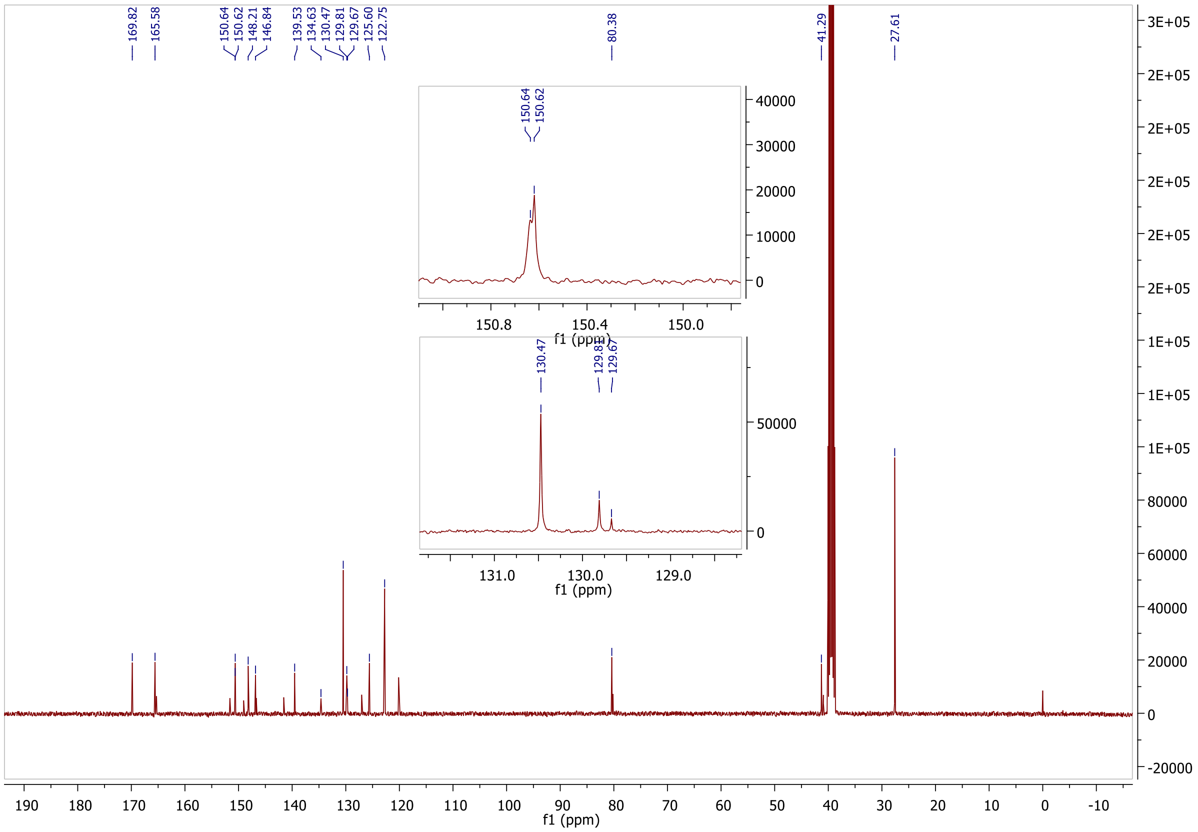

Additional (minor) signals derive from the (*Z*)-photoisomer formed by daylight.

(*E*)-5-[(4-{[(*tert*-Butoxycarbonyl)amino]methyl}phenyl)diazenyl]nicotinamide (**15c**)

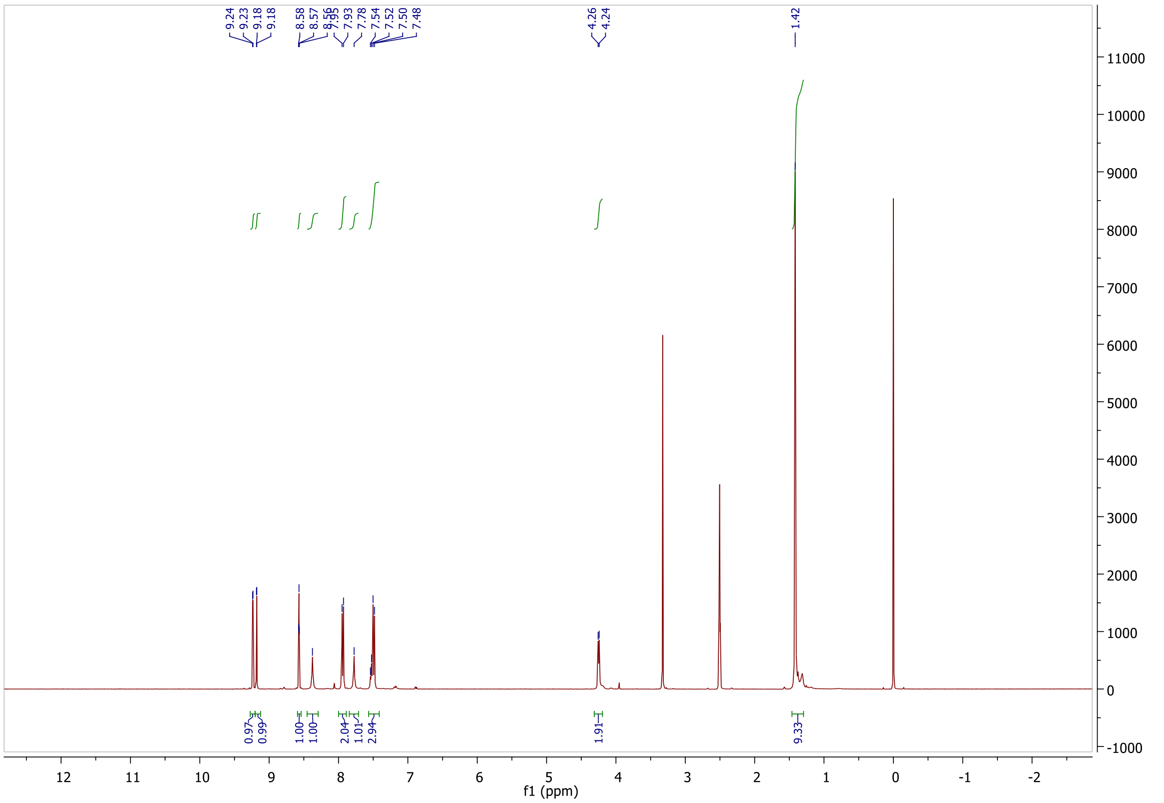

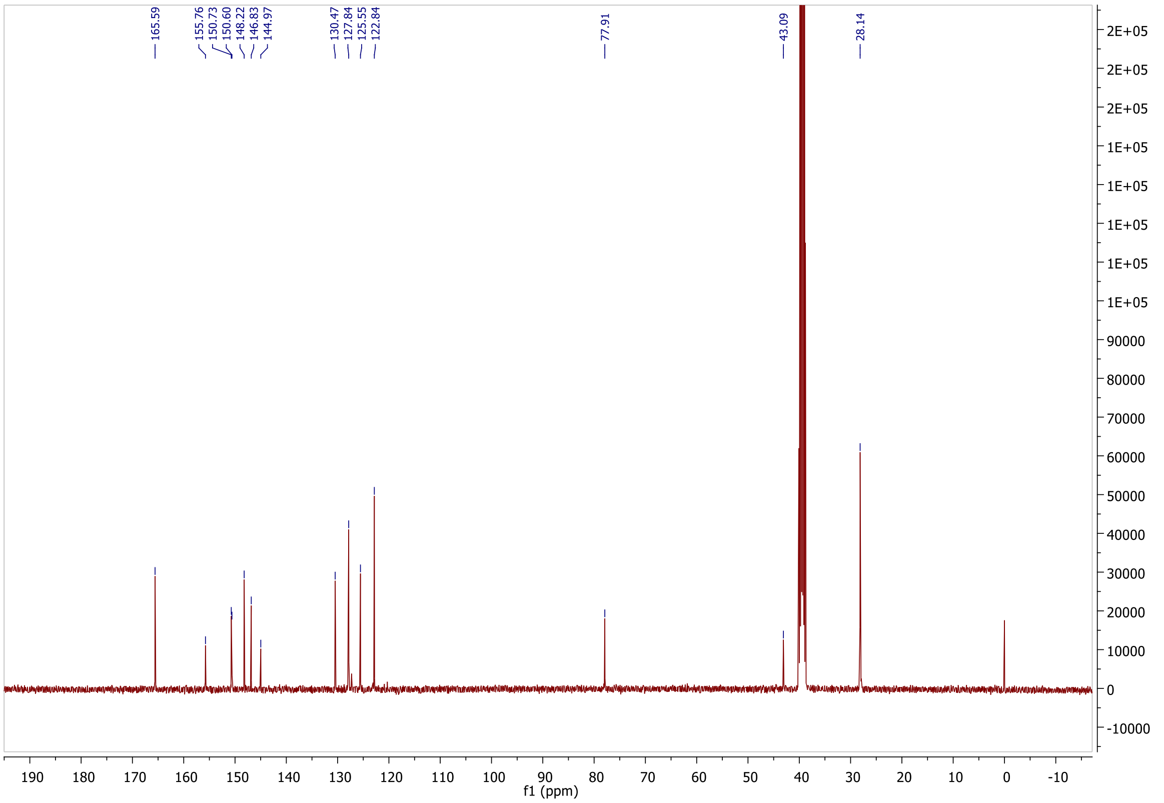

Additional (minor) signals derive from the (*Z*)-photoisomer formed by daylight.

(*E*)-5-[(4-Bromophenyl)diazenyl]nicotinamide (**15d**)

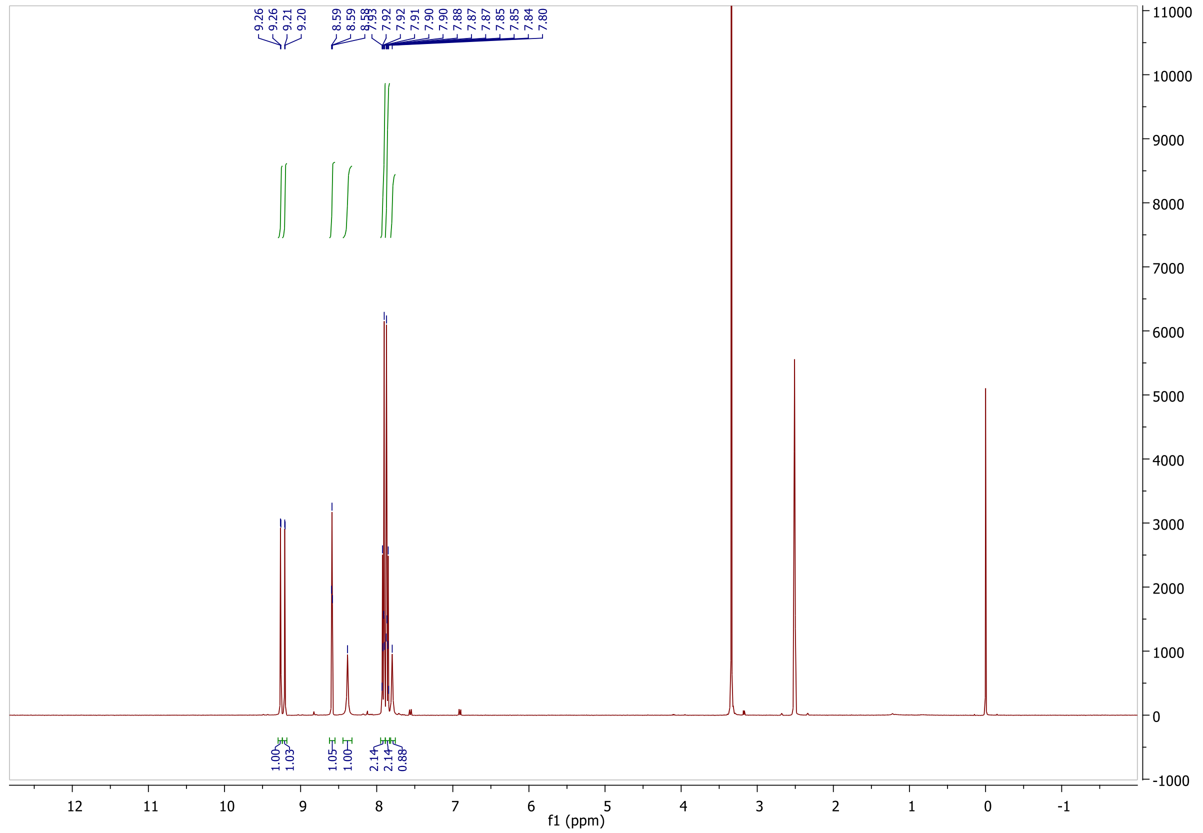

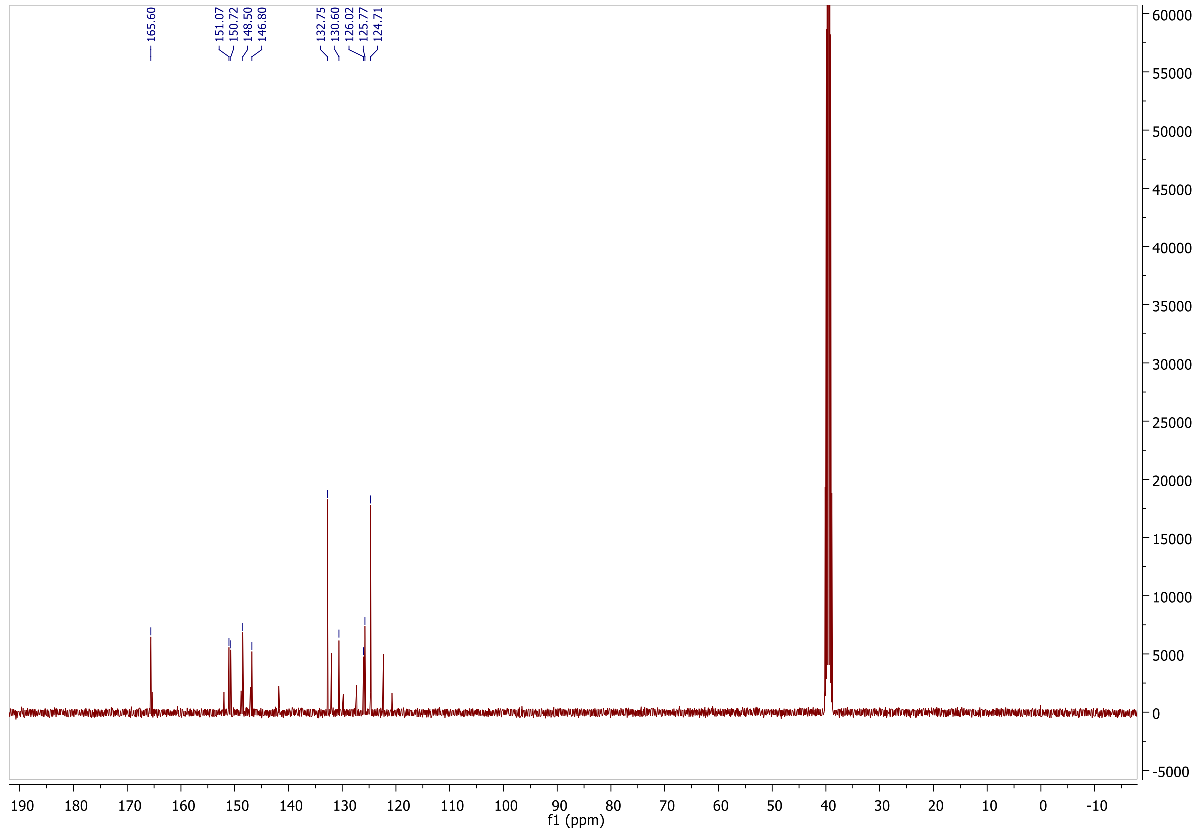

Additional (minor) signals derive from the (*Z*)-photoisomer formed by daylight.

(*E*)-5-{[4-(Butylcarbamoyl)phenyl]diazenyl}nicotinamide (**15e**)

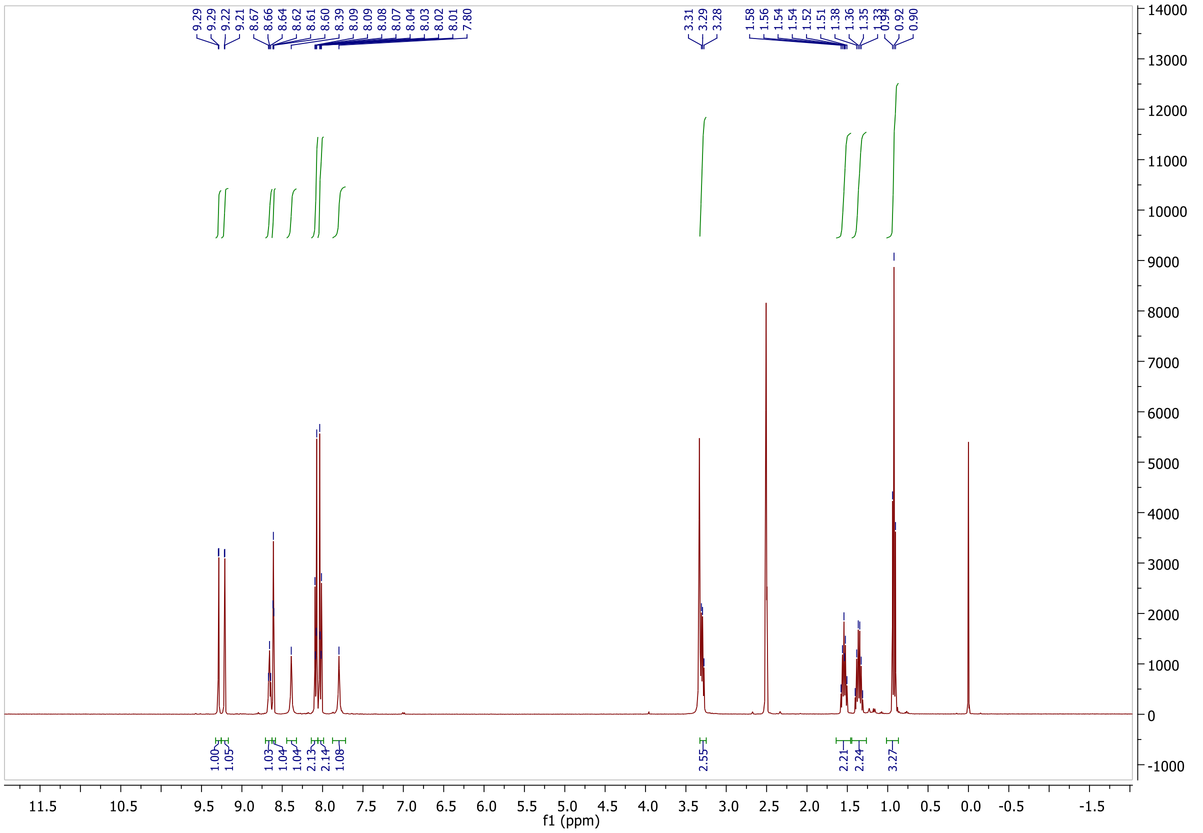

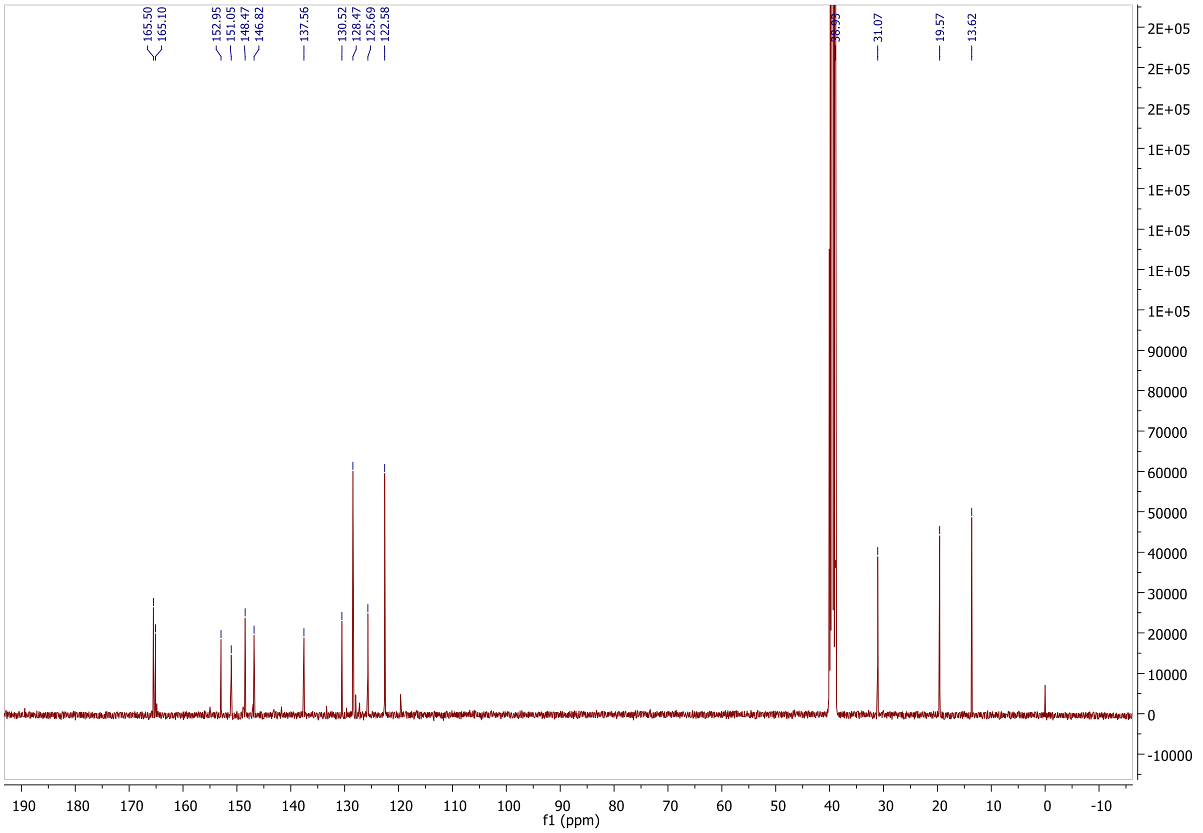

Additional (minor) signals derive from the (*Z*)-photoisomer formed by daylight.

(*E*)-5-{[4-(Decanamidomethyl)phenyl]diazenyl}nicotinamide (**15f**)

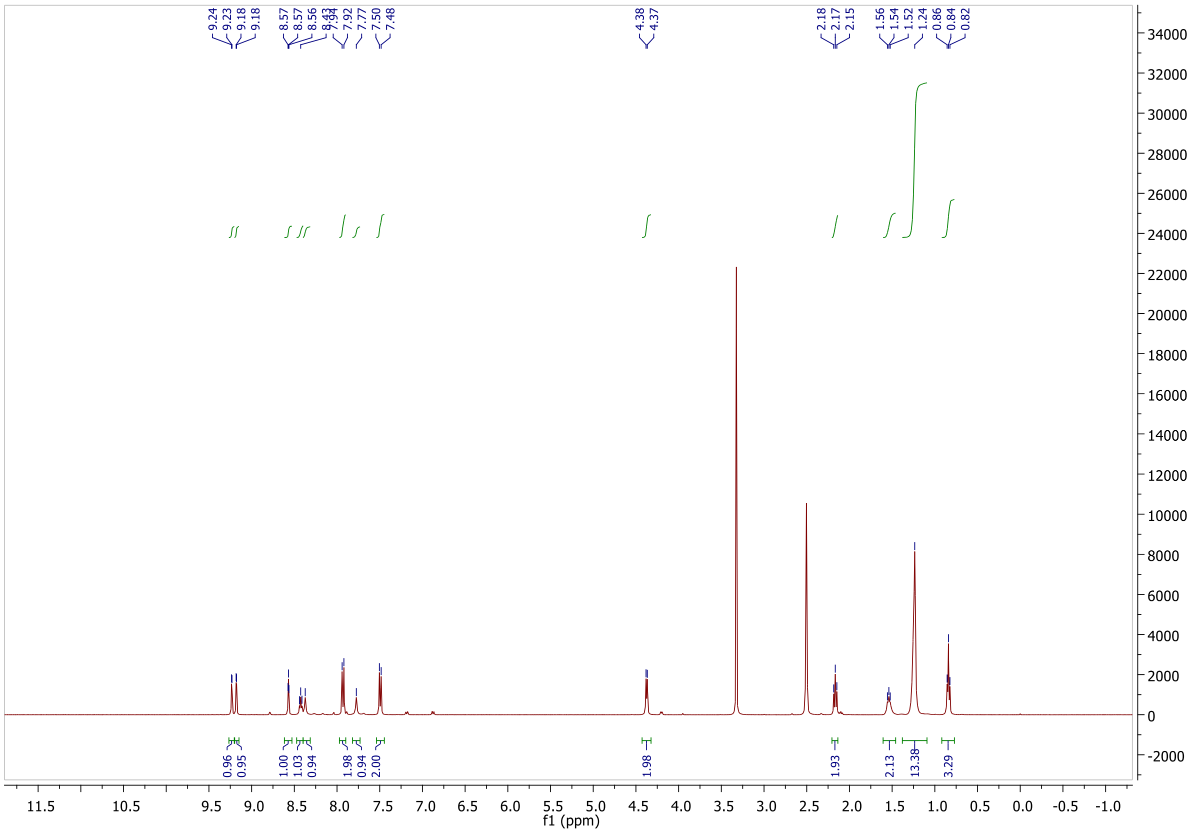

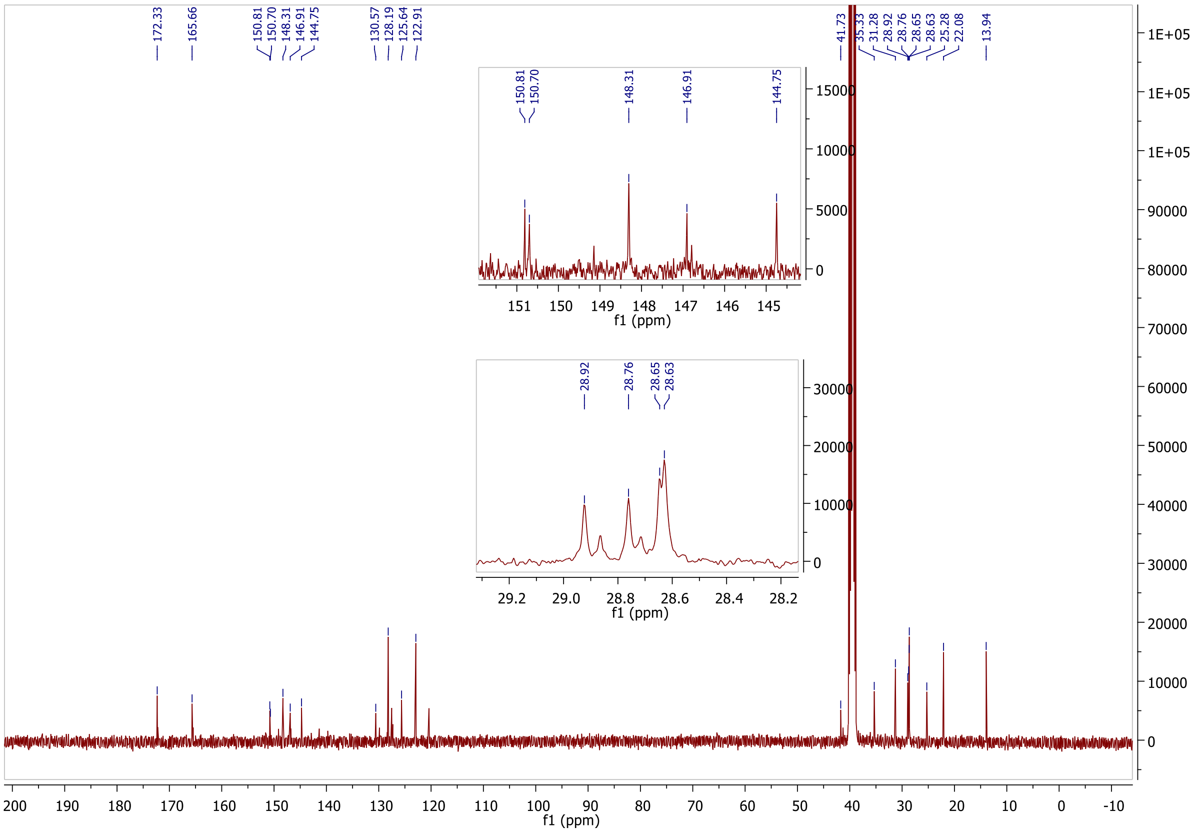

Additional (minor) signals derive from the (*Z*)-photoisomer formed by daylight.

(*E*)-5-{[4-(6-Hydroxyhex-1-yn-1-yl)phenyl]diazenyl}nicotinamide (**15g**)

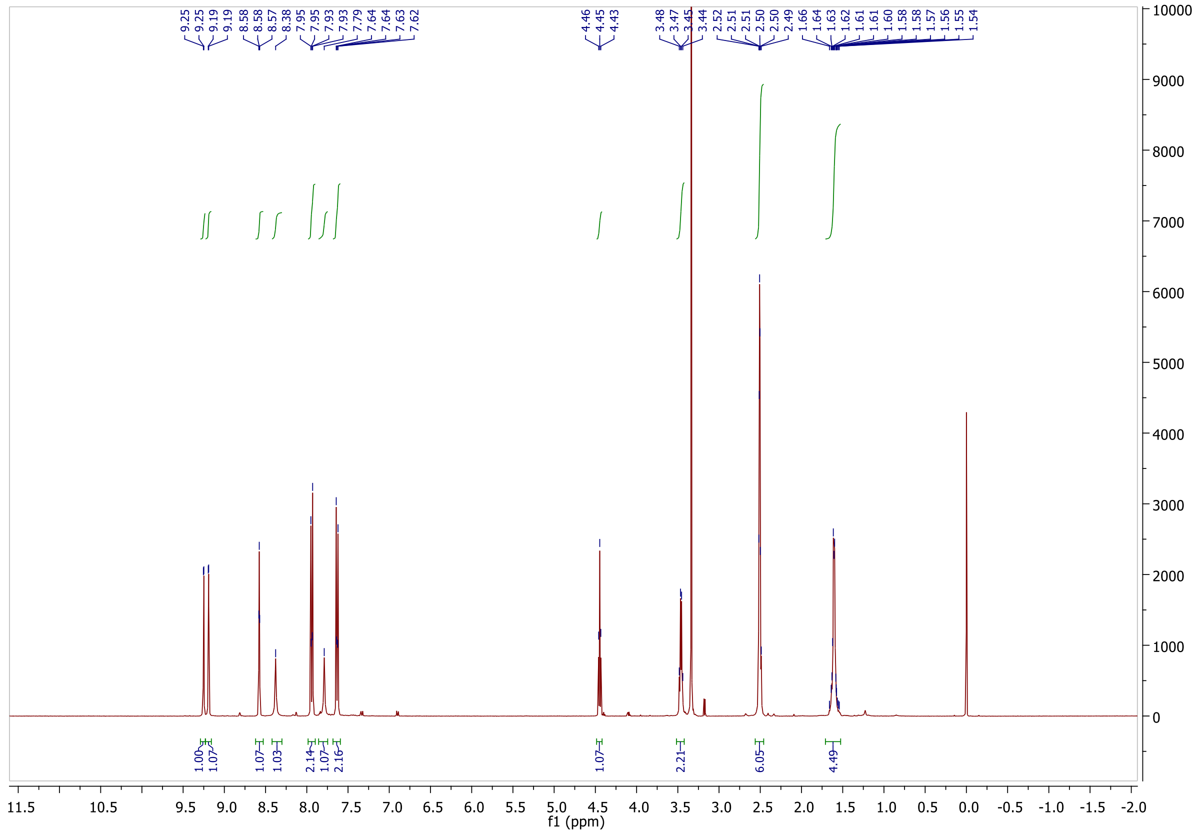

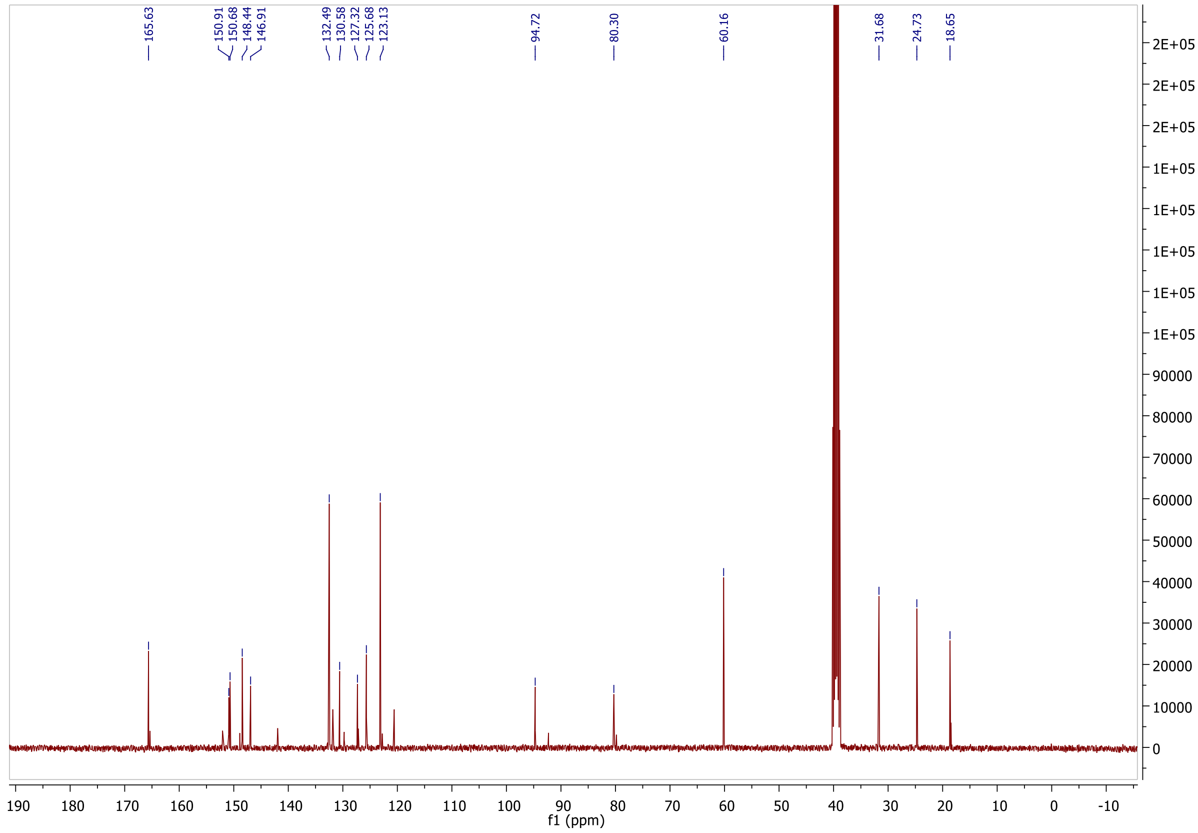

Additional (minor) signals derive from the (*Z*)-photoisomer formed by daylight.

(*E*)-5-{[4-(3-Hydroxy-3-methylbut-1-yn-1-yl)phenyl]diazenyl}nicotinamide (**15h**)

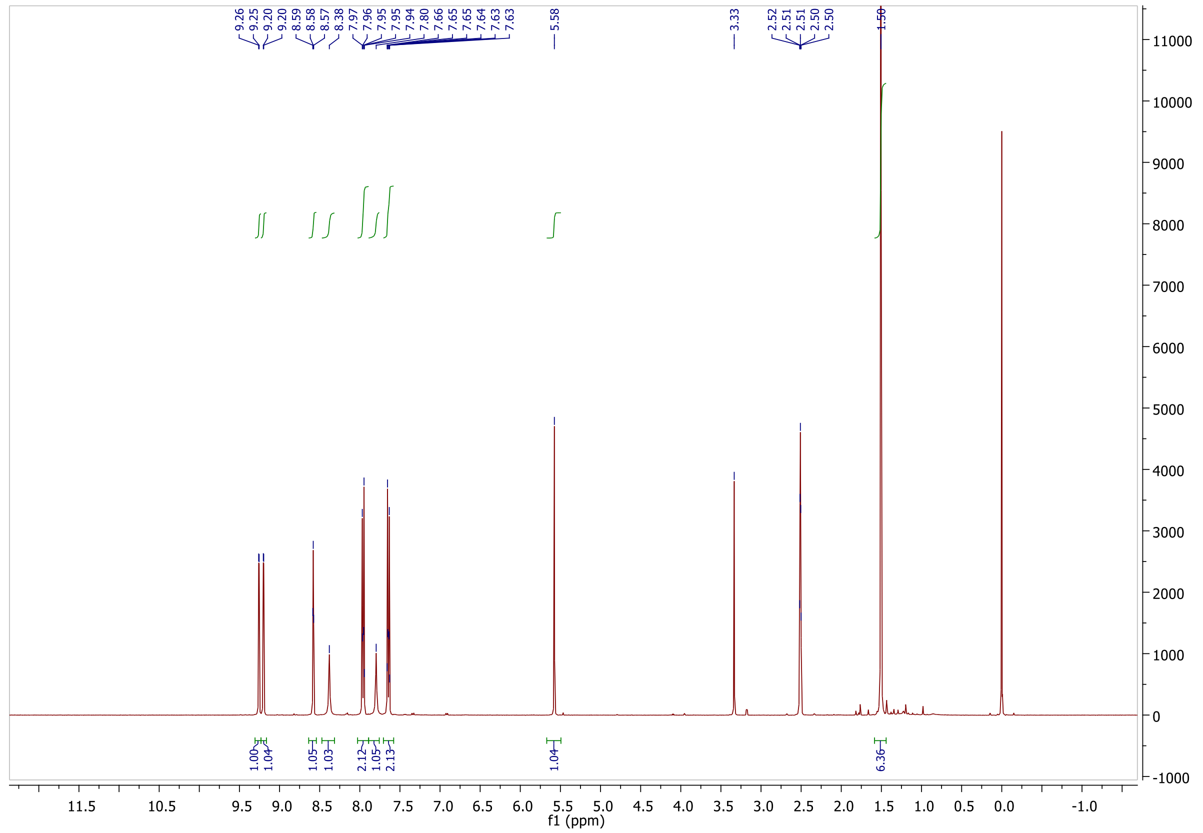

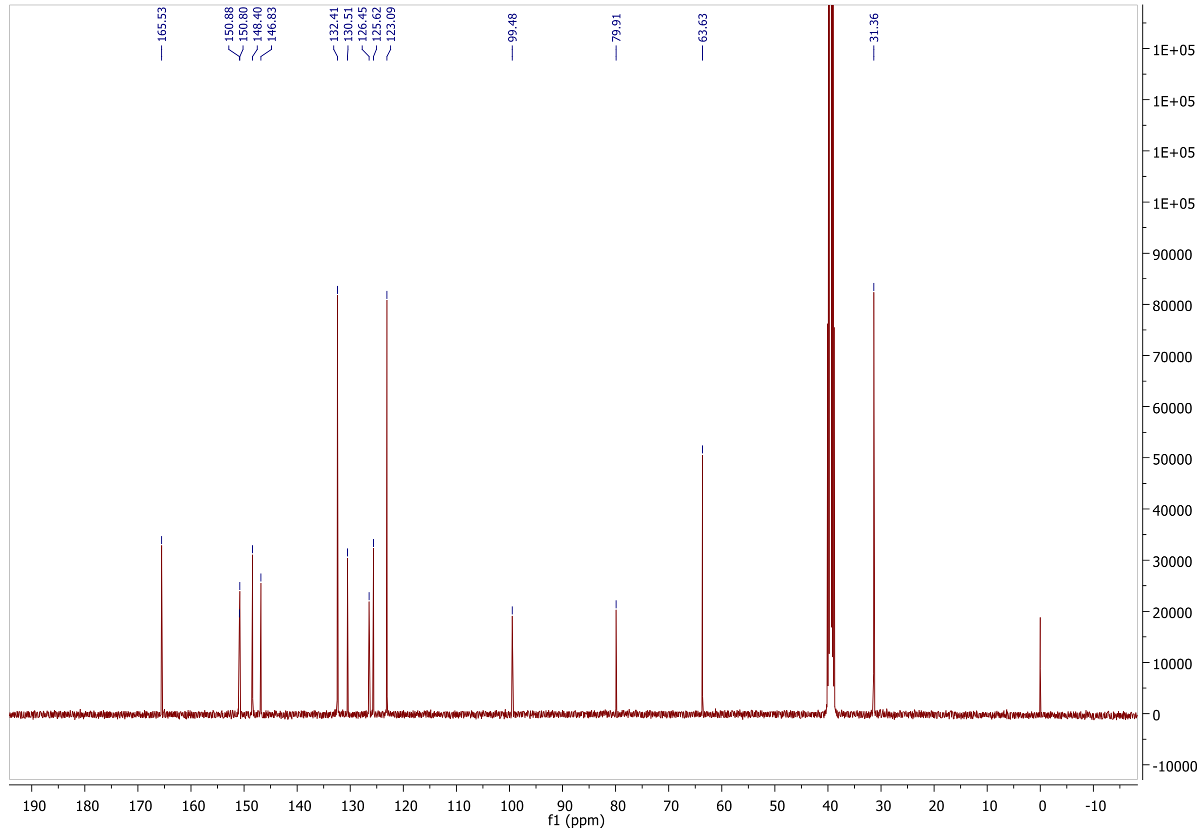

Methyl 5-[(3-nitrobenzyl)oxy]nicotinate (**17**)

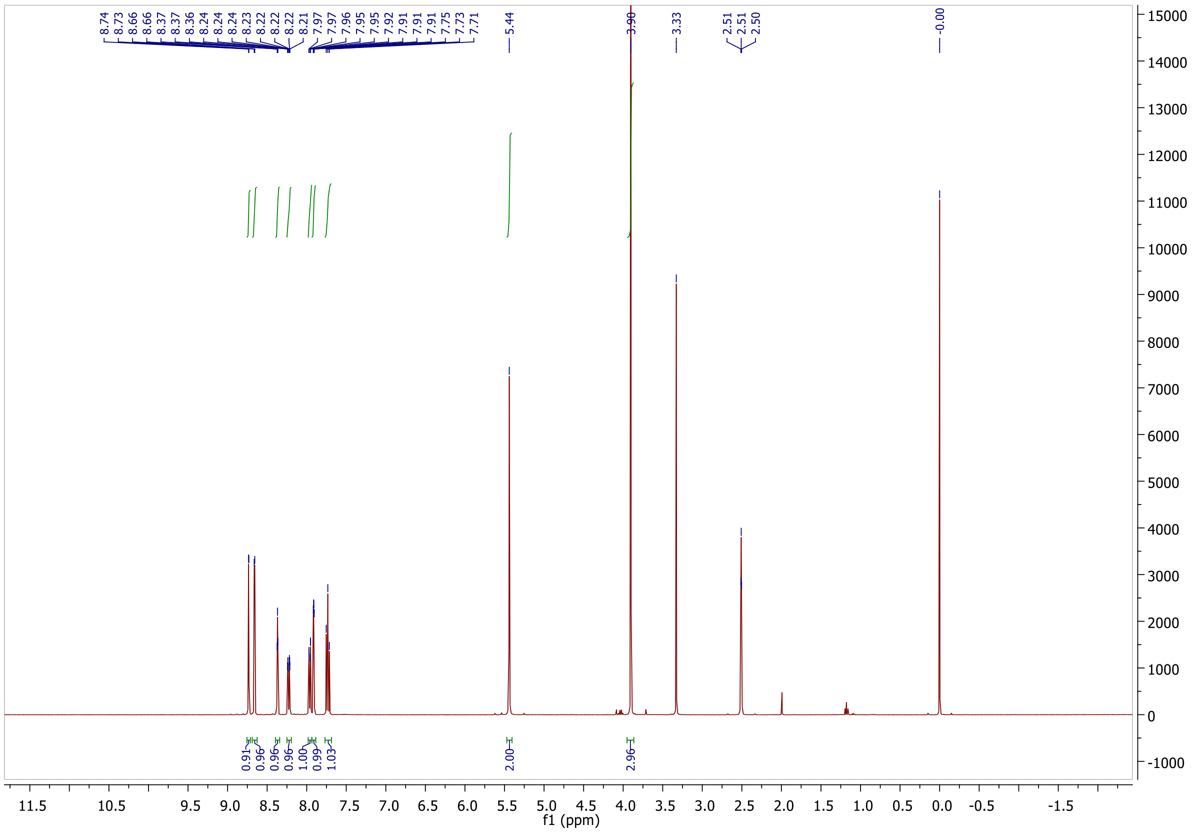

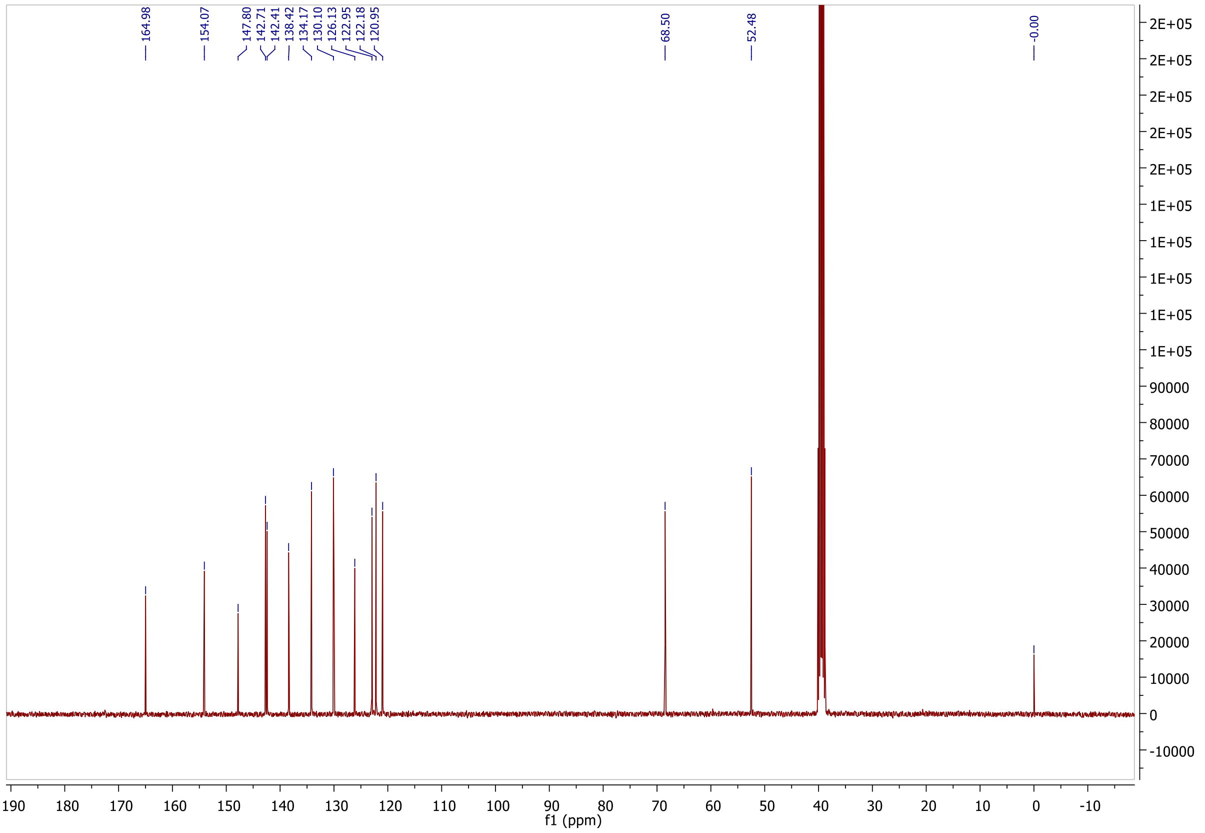

Methyl 5-[(3-aminobenzyl)oxy]nicotinate (**18**)

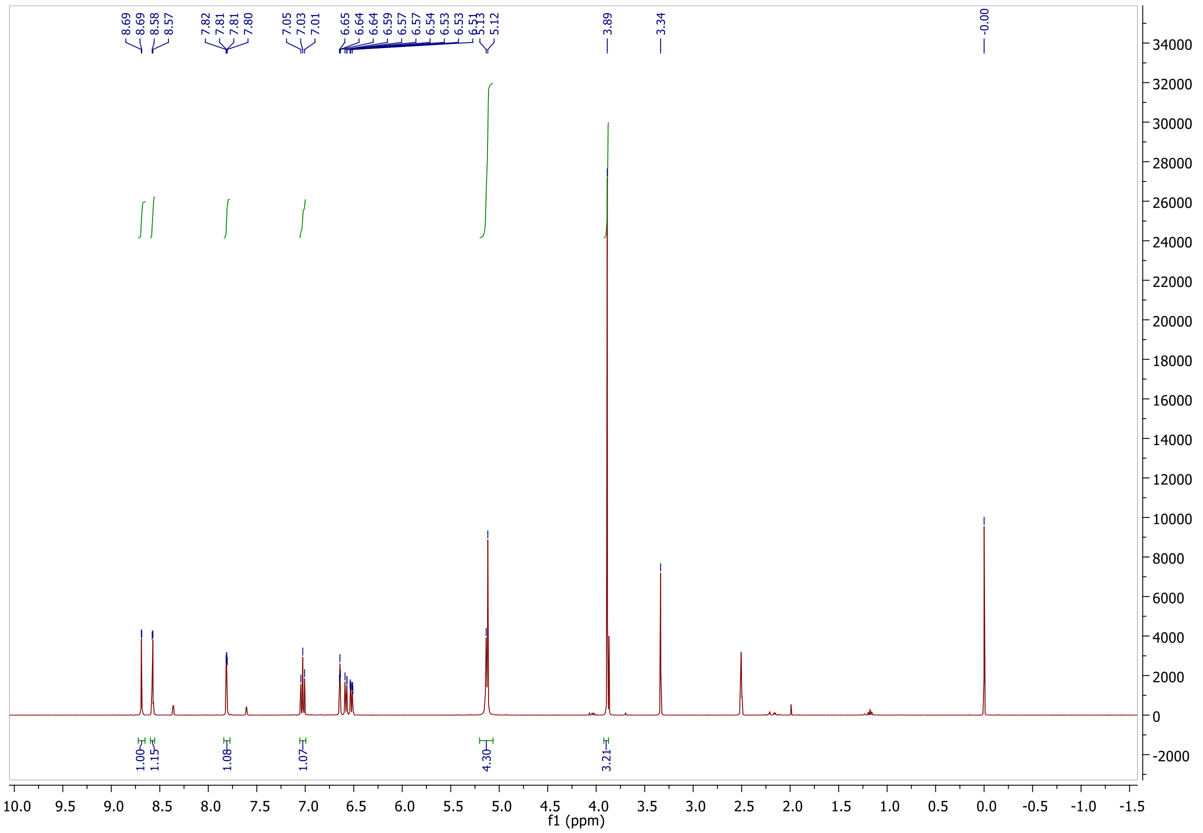

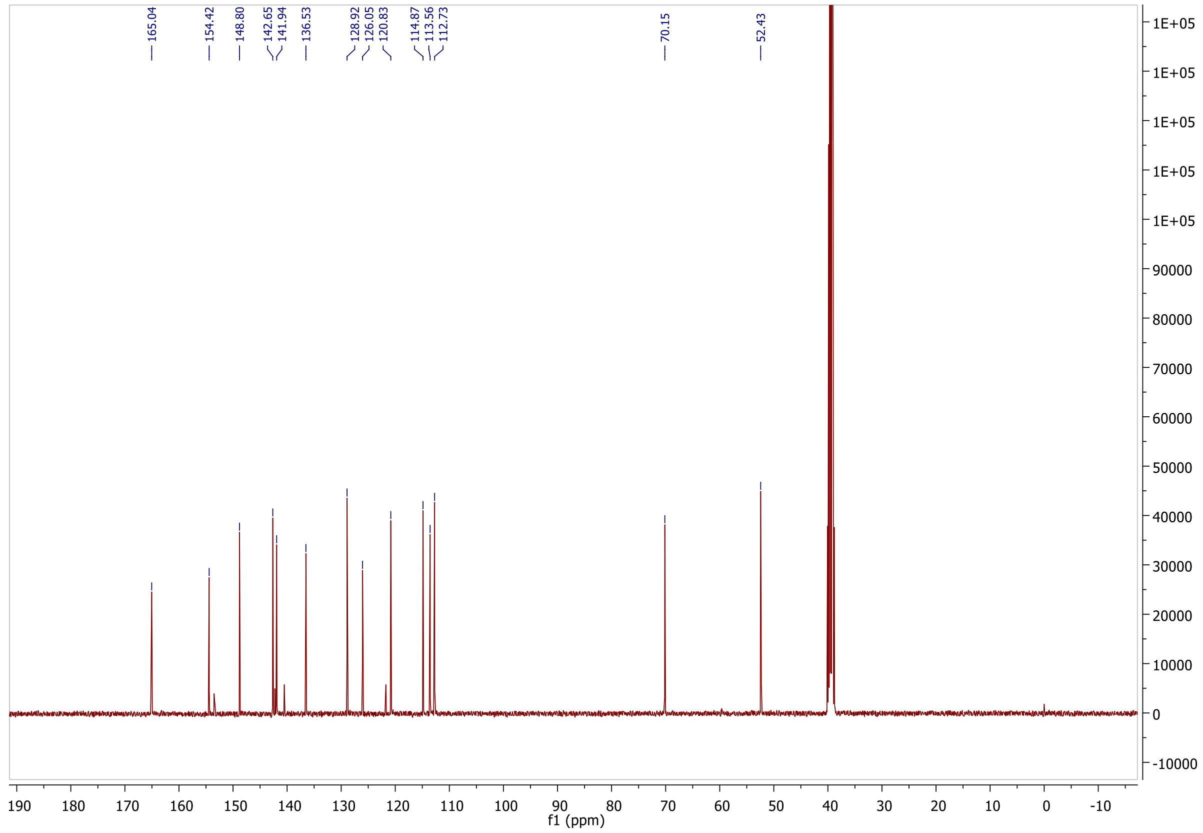

Methyl (*E*)-5-[(3-{[4-(benzyloxy)phenyl]diazenyl}benzyl)oxy]nicotinate (**19a**)

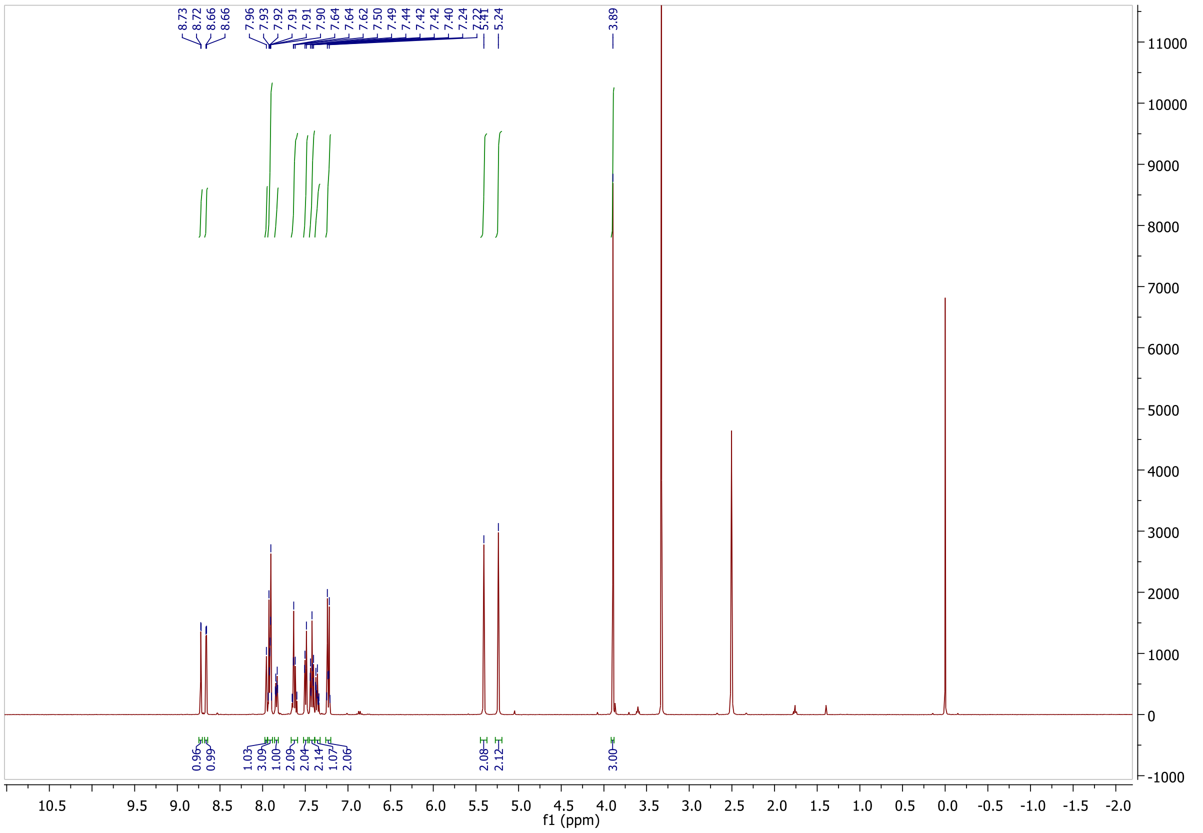

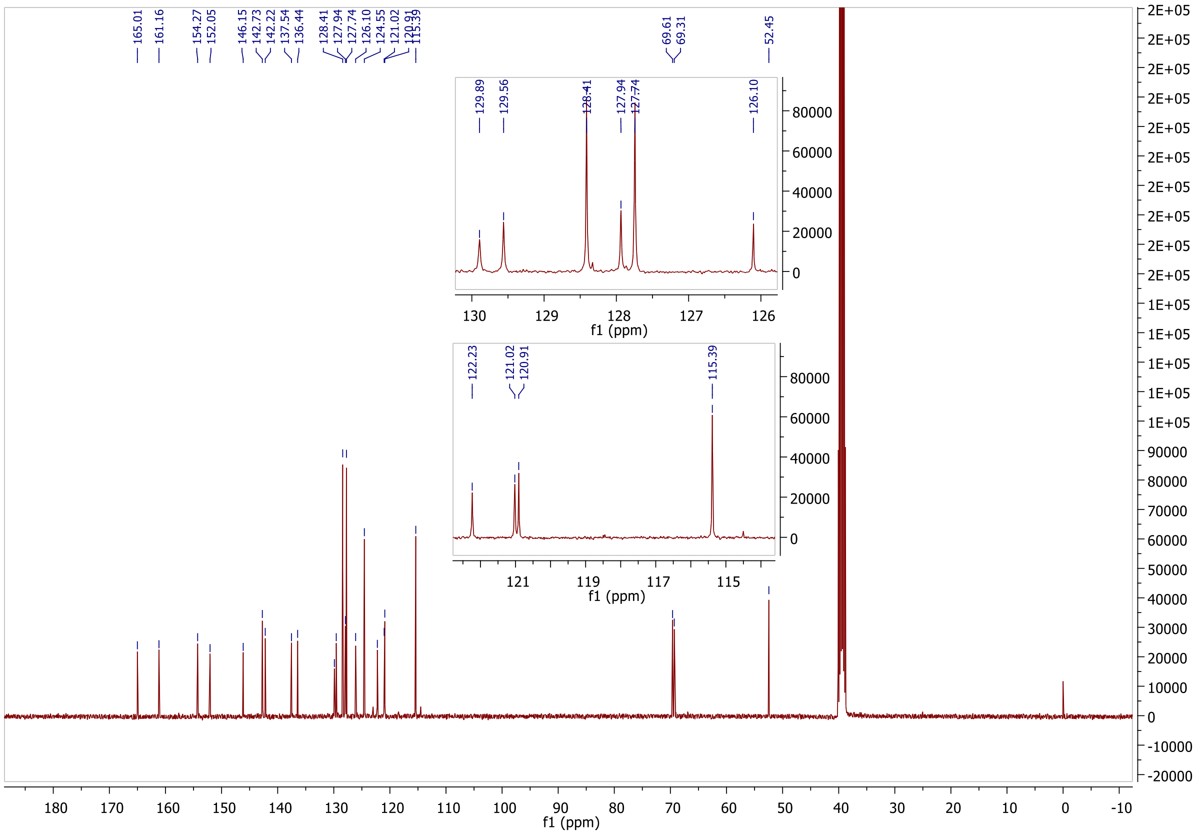

Additional (minor) signals derive from the (*Z*)-photoisomer formed by daylight.

Methyl (*E*)-5-({3-[(4-fluorophenyl)diazenyl]benzyl}oxy)nicotinate (**19b**)

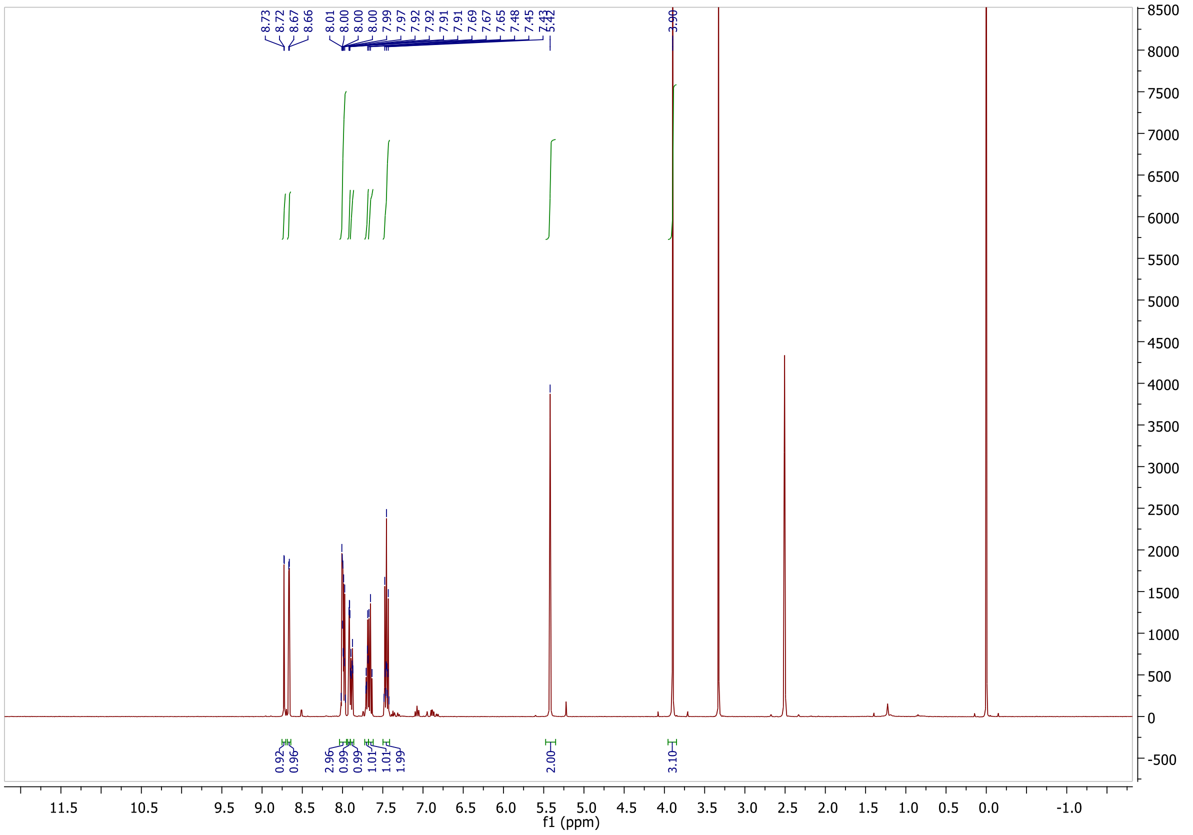

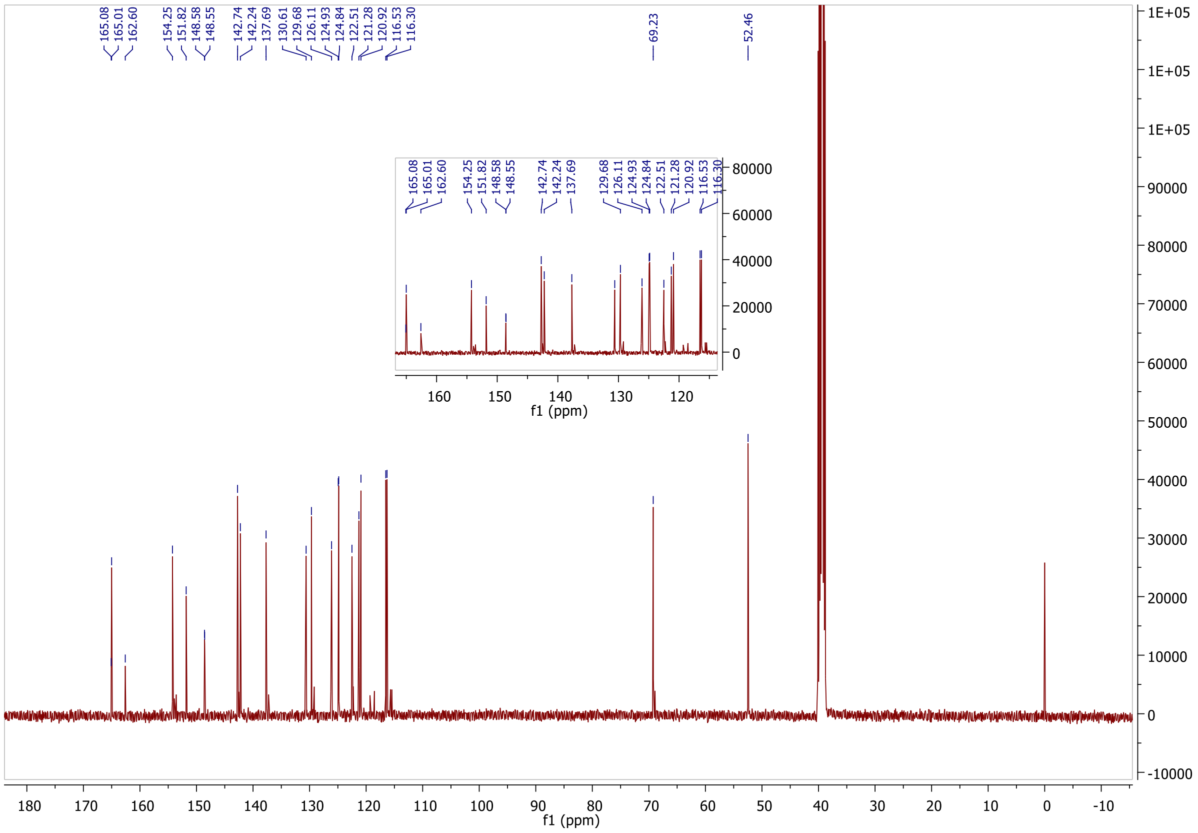

Additional (minor) signals derive from the (*Z*)-photoisomer formed by daylight.

Methyl (*E*)-5-({3-[(4-ethynylphenyl)diazenyl]benzyl}oxy)nicotinate (**19c**)

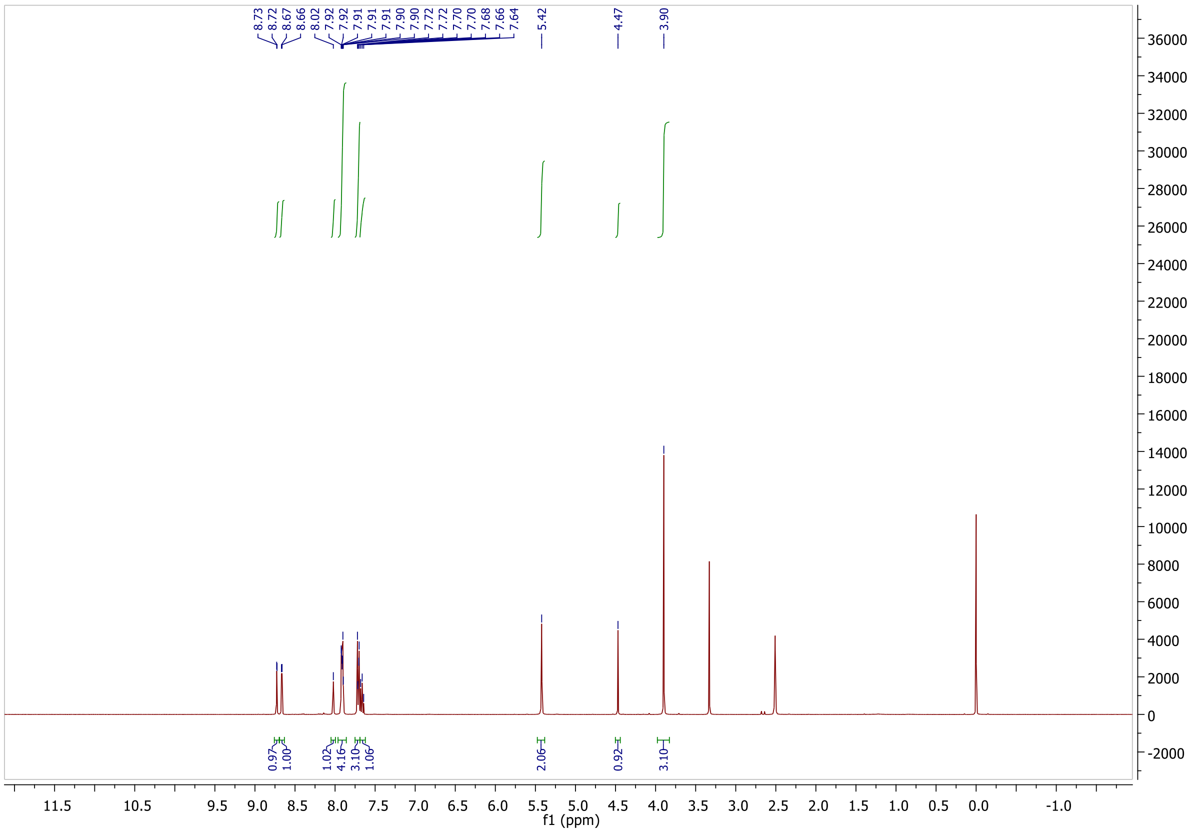

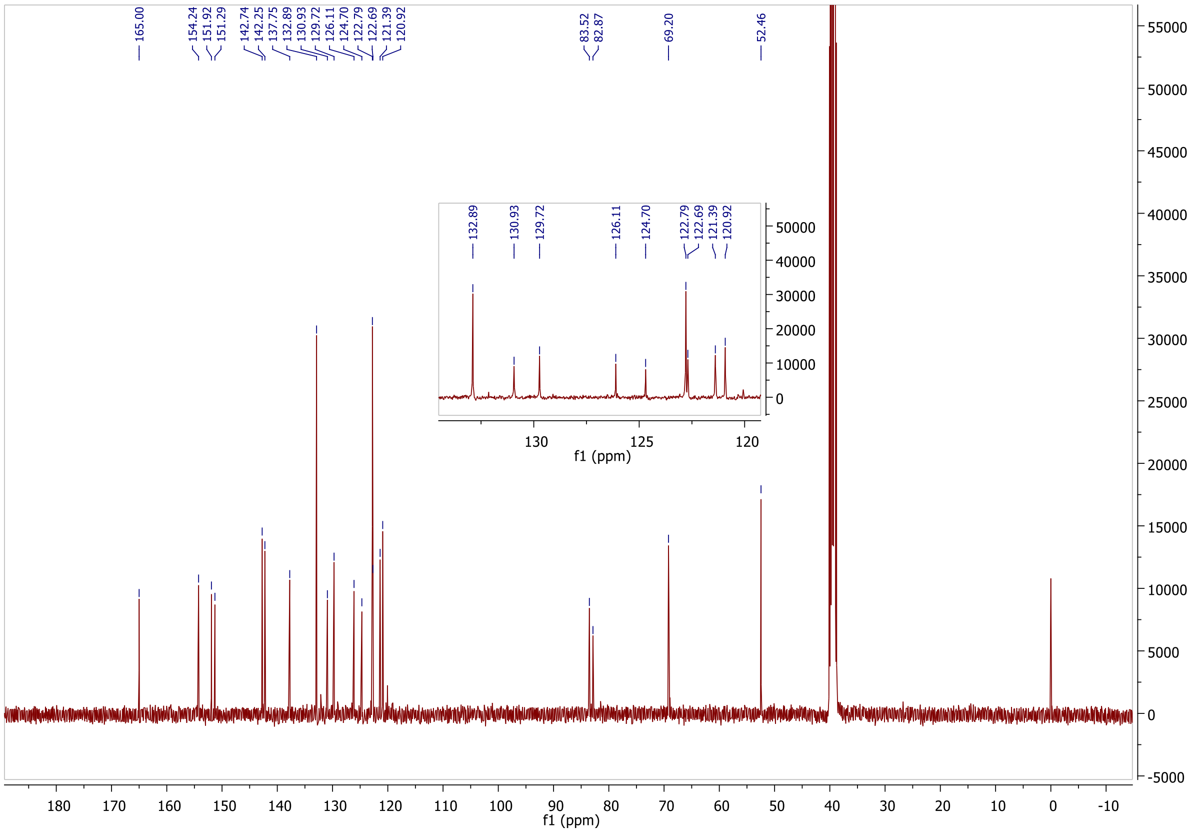

Additional (minor) signals derive from the (*Z*)-photoisomer formed by daylight.

Methyl (*E*)-5-({3-[(4-morpholinophenyl)diazenyl]benzyl}oxy)nicotinate (**19d**)

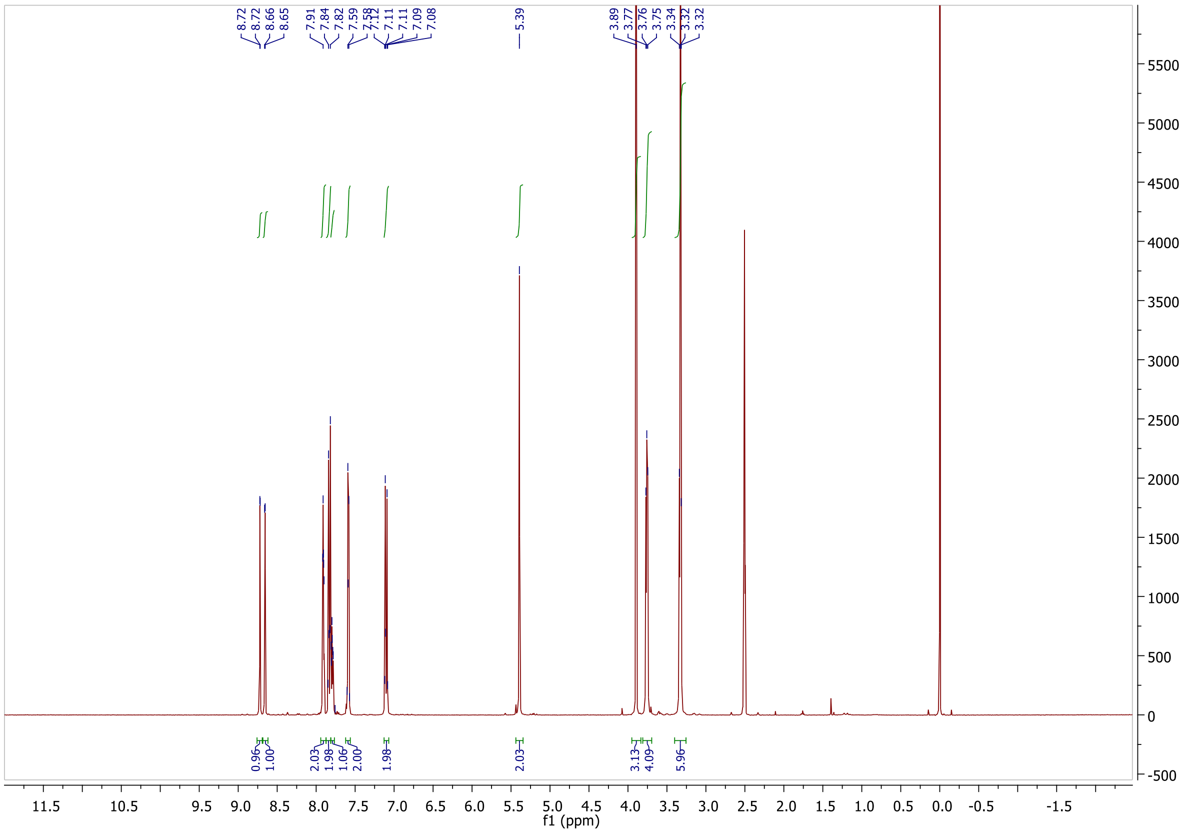

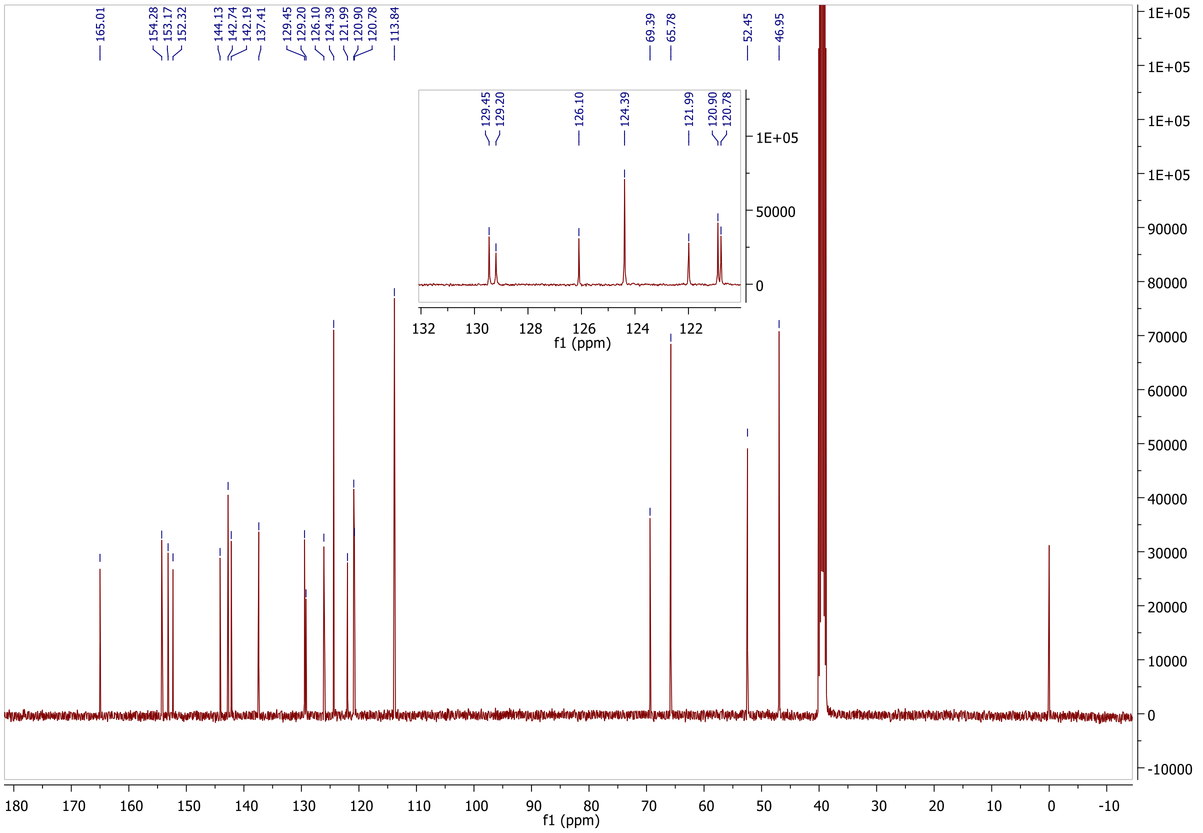

(*E*)-5-[(3-{[4-(benzyloxy)phenyl]diazenyl}benzyl)oxy]nicotinamide (**20a**)

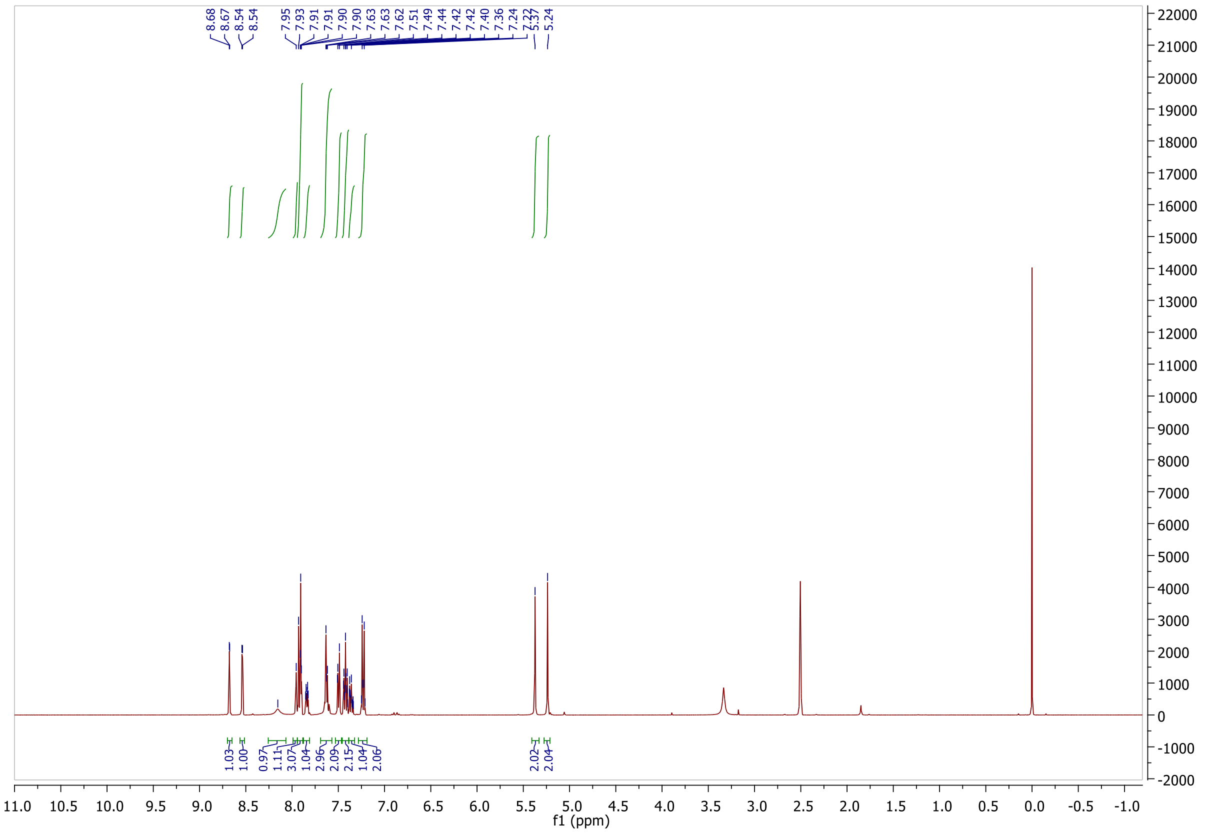

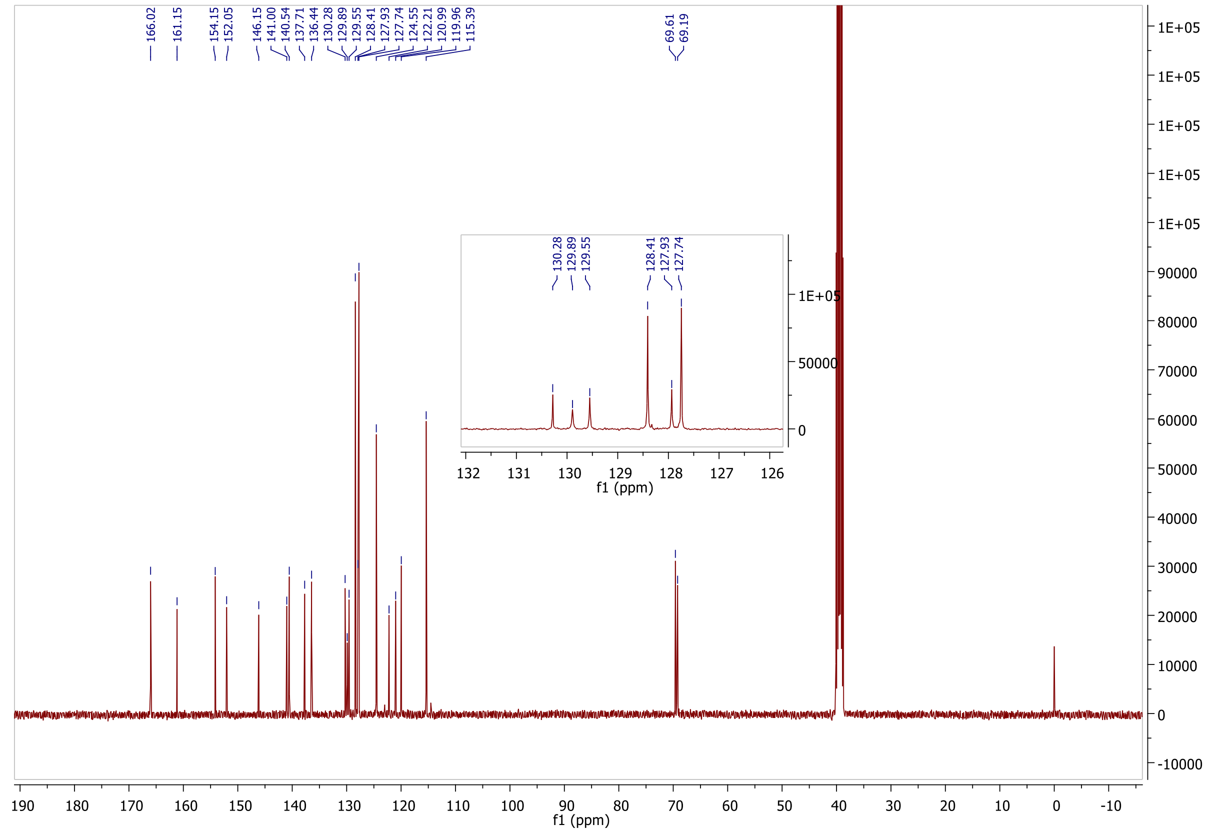

(*E*)-5-({3-[(4-Fluorophenyl)diazenyl]benzyl}oxy)nicotinamide (**20b**)

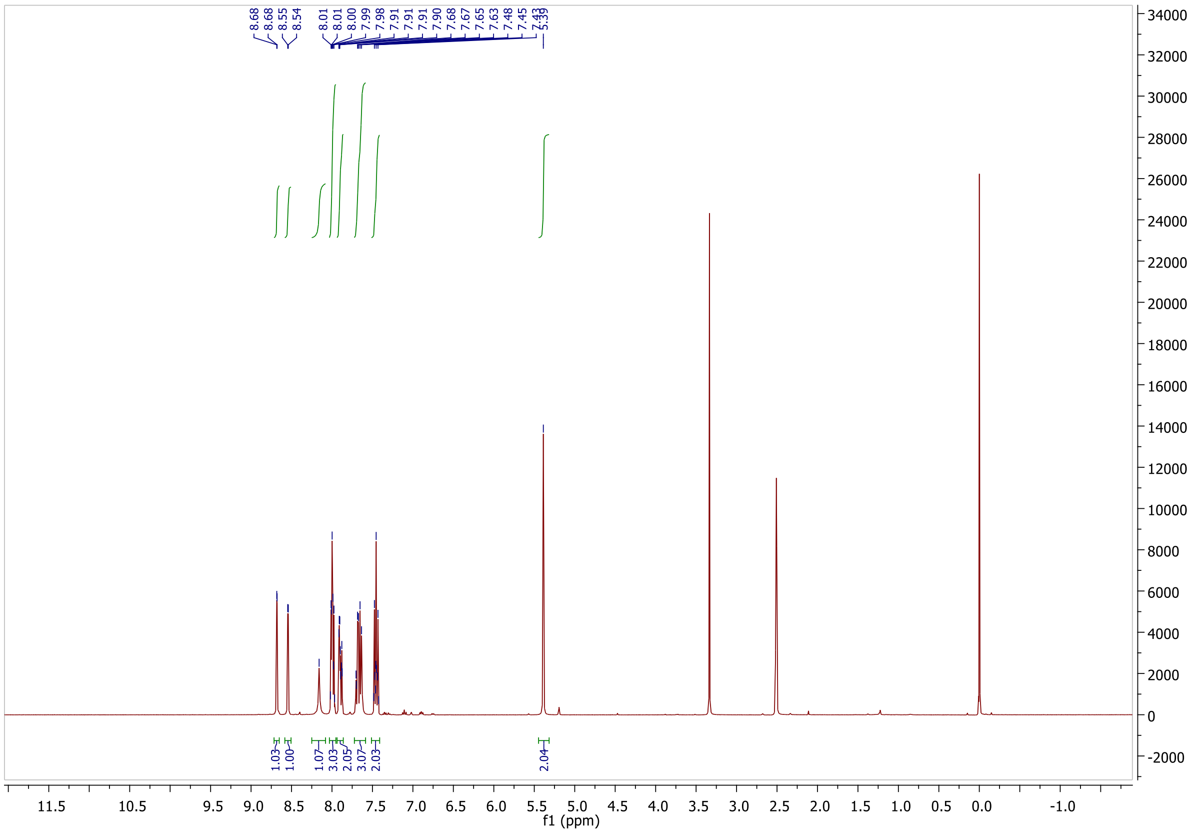

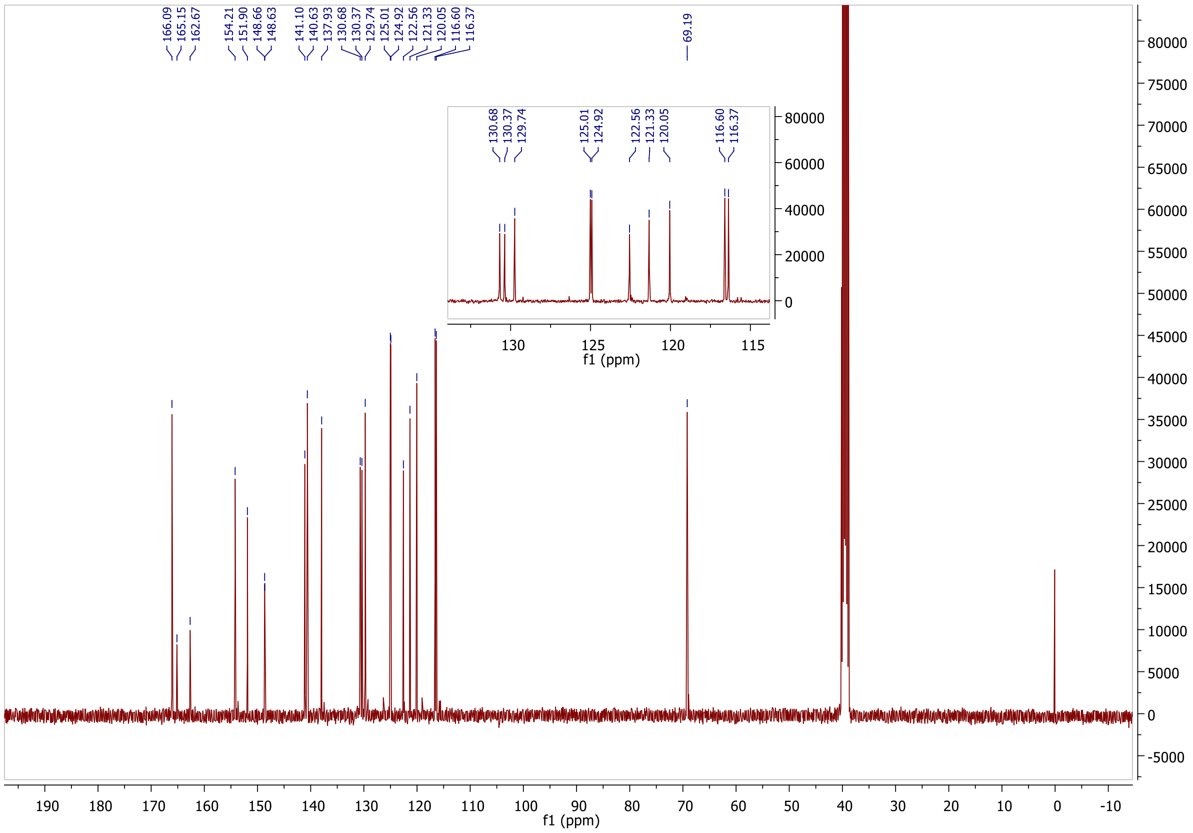

Additional (minor) signals derive from the (*Z*)-photoisomer formed by daylight.

(*E*)-5-({3-[(4-Ethynylphenyl)diazenyl]benzyl}oxy)nicotinamide (**20c**)

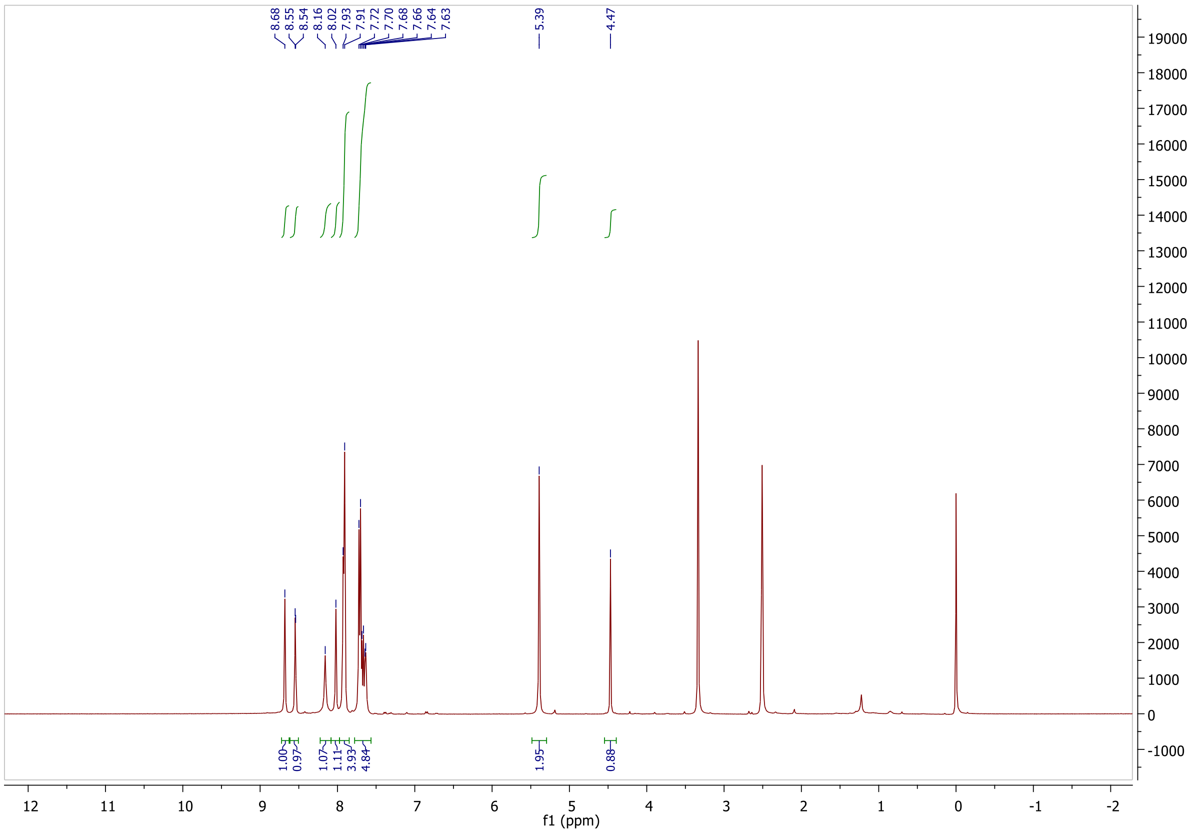

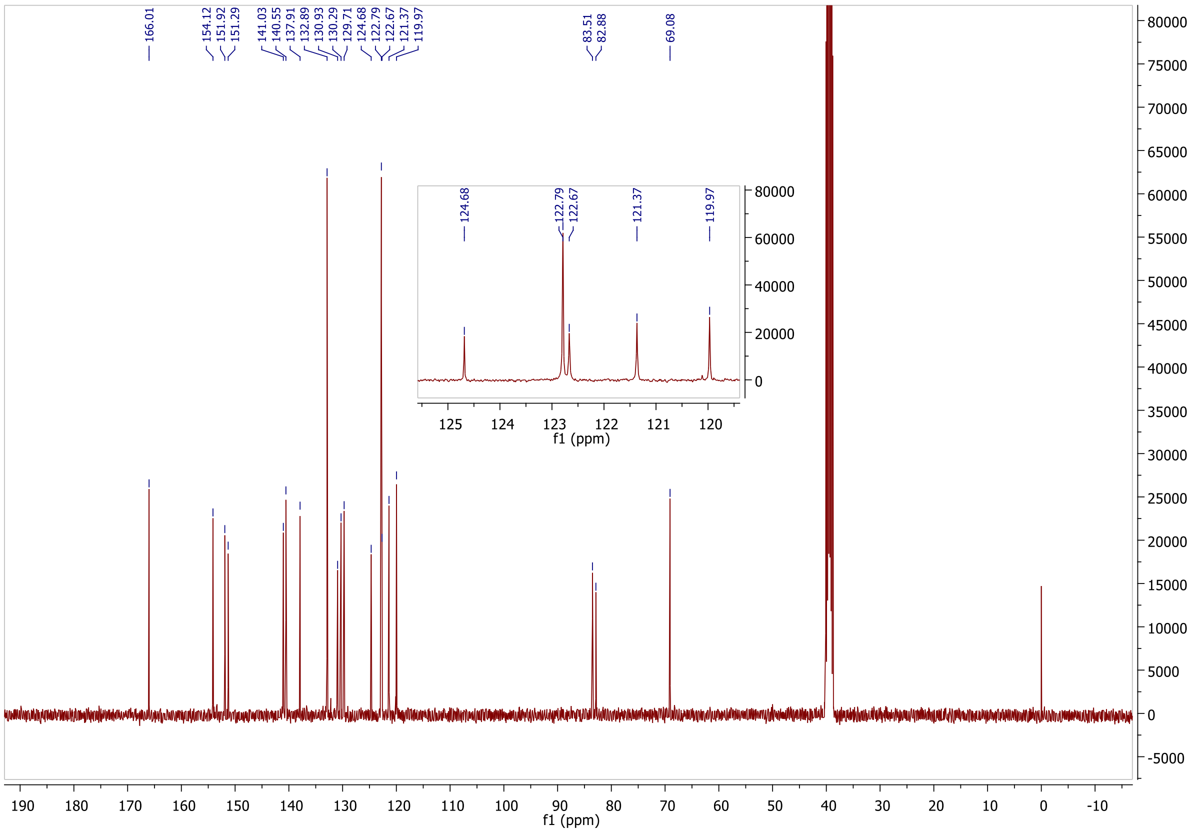

(*E*)-5-({3-[(4-Morpholinophenyl)diazenyl]benzyl}oxy)nicotinamide (**20d**)

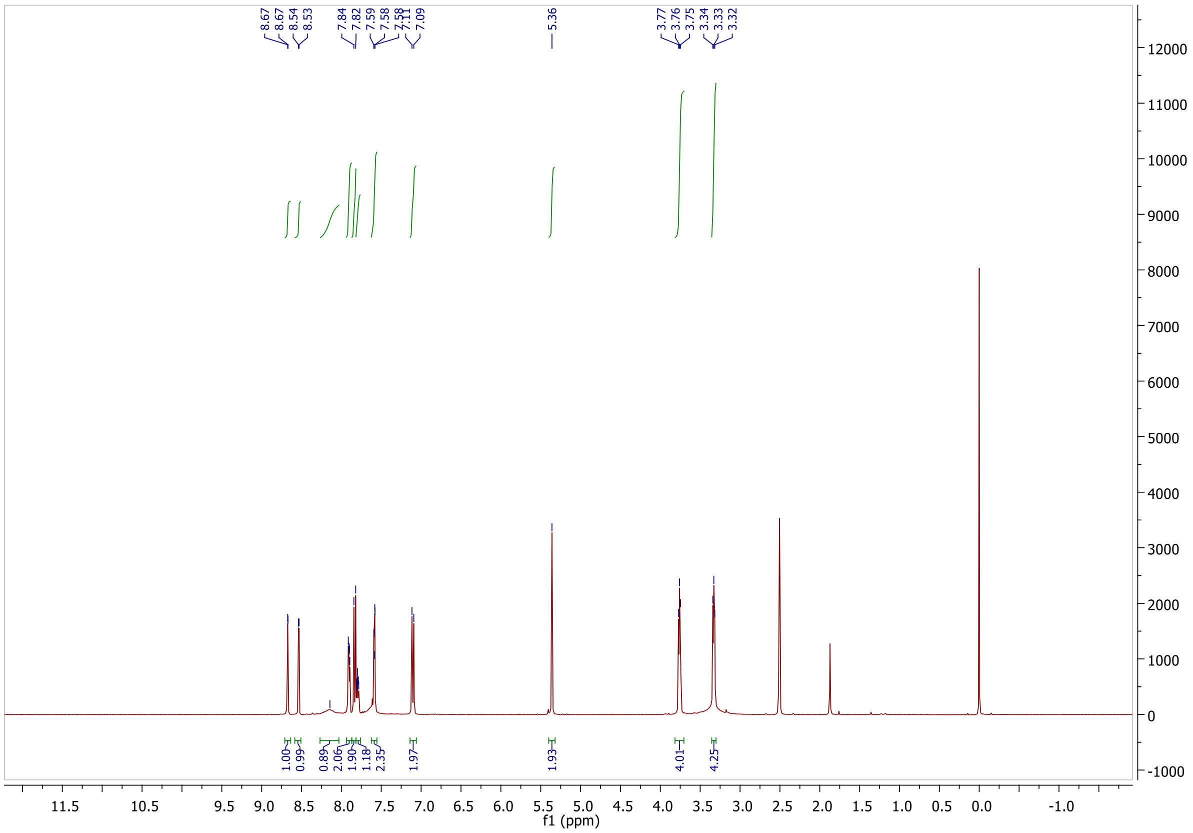

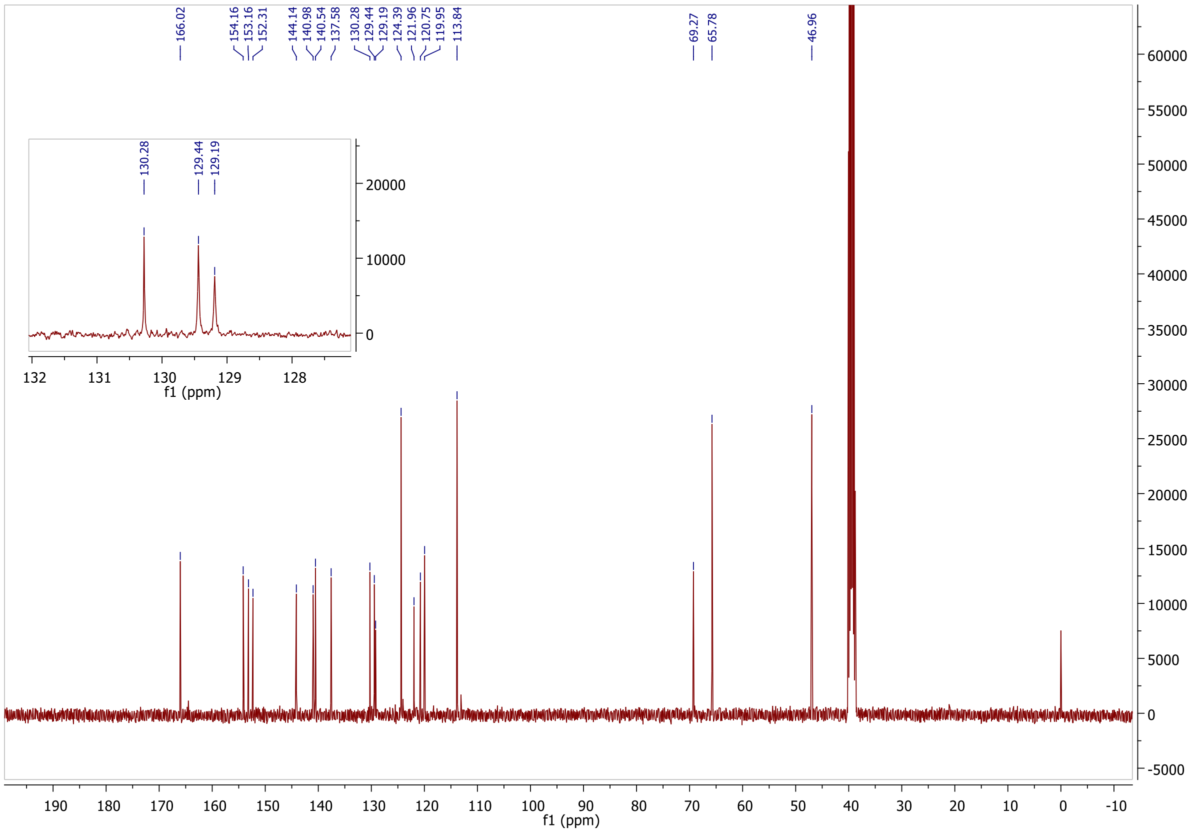

Supplement: Supplementary file 1 — Supplementary [file CMDC-15-1480-s001.pdf]
